# A novel approach to finding conserved features in low-variability gene alignments characterises RNA motifs in SARS-CoV and SARS-CoV-2

**DOI:** 10.1038/s41598-023-39207-1

**Published:** 2023-07-26

**Authors:** Jordan P. Skittrall, Nerea Irigoyen, Ian Brierley, Julia R. Gog

**Affiliations:** 1 Department of Pathology, Division of Virology, Addenbrooke’s Hospital, University of Cambridge, Hills Road, Cambridge, CB2 0QQ UK; 2 Department of Applied Mathematics and Theoretical Physics, Centre for Mathematical Sciences, University of Cambridge, Wilberforce Road, Cambridge, CB3 0WA UK

**Keywords:** Computational models, Data mining, SARS-CoV-2, SARS virus, Viral evolution, Virus structures, Viral genetics

## Abstract

Collections of genetic sequences belonging to related organisms contain information on the evolutionary constraints to which the organisms have been subjected. Heavily constrained regions can be investigated to understand their roles in an organism’s life cycle, and drugs can be sought to disrupt these roles. In organisms with low genetic diversity, such as newly-emerged pathogens, it is key to obtain this information early to develop new treatments. Here, we present methods that ensure we can leverage all the information available in a low-signal, low-noise set of sequences, to find contiguous regions of relatively conserved nucleic acid. We demonstrate the application of these methods by analysing over 5 million genome sequences of the recently-emerged RNA virus SARS-CoV-2 and correlating these results with an analysis of 119 genome sequences of SARS-CoV. We propose the precise location of a previously described packaging signal, and discuss explanations for other regions of high conservation.

## Introduction

A key challenge in the development of antivirals is the relative lack of targets that are sufficiently conserved across multiple families of viruses for drugs to exhibit a broad spectrum of activity. Coupled with the need to avoid host toxicity from drug cross-reactivity with host molecules, the identification of drug targets that control key lifecycle features of viruses, but that do not have close analogues in the host or in unrelated viruses, becomes an important goal. The identification and functional characterisation of such targets is a difficult informatic and biological problem, precisely because of the lack of analogues for comparison.

One class of approaches to this problem seeks to find regions of high conservation in viral nucleic acid that cannot be explained by conservation in resultant amino acids. The idea of these approaches is that such nucleic acid conservation may correlate with previously uncharacterised lifecycle features. Such approaches have, for example, found packaging signals and previously undescribed proteins in influenza A virus^1–3^, noted a previously undescribed open reading frame in enteroviruses^4^, and identified conserved structural elements in group A rotaviruses^5^. Many such approaches have used a sliding window technique to find conserved regions in sequences: such a technique is often adequate, as demonstrated by its successes, and is particularly appropriate when the length scale of the expected regions is known, but otherwise it imposes a length scale on the problem that may make it more difficult to discover regions differing from this scale. The difficulty may be overcome by repeating analyses with different window sizes, at a cost of increased computational and analytical time and increased risk of false-positive signals.

We have previously described a scale-agnostic approach to this problem^6^, showing that this approach could find previously discovered conserved regions of nucleic acid in influenza A virus and group A rotaviruses, and successfully applied this approach to a new analysis of HIV-1^7^. Such an approach is more efficient at finding conserved regions of unexpected length scales, potentially at the cost of being less efficient at finding conserved regions at tuned length scales.

All the approaches described so far work in RNA virus genome datasets in which relatively high variability is seen between different sequenced samples. This means that both “signal” (conserved) and “background” (variable) regions are seen to exhibit variability, but the lower variability in signal regions can be detected. However, even amongst RNA virus genome datasets there may be found examples where the overall variability between samples is low and so it is more challenging to distinguish signal from background. There are two important classes of such datasets. The first class contains datasets of truly newly emerged viruses, such as SARS-CoV-2, where the dataset available represents well the evolutionary space the human-tropic virus has explored but variability is still low because limited time means the overall explored region is small. The second class contains datasets of highly adapted viruses, such as the flaviviruses, which have likely co-evolved with more than one host species for timescales around 100 million years^8^. In this example it is likely that viruses have explored accessible evolutionary space in the past, reaching a high reproductive fitness niche from which little deviation is observed in recently sequenced samples. In both cases, we need to use all the information available in a low noise, low signal context to discover conserved regions: in the first case, because we wish to develop therapeutics for an emerging disease as early as possible; in the second case, because waiting will not yield substantially more information and so any attempts to develop therapeutics can only use the information already available.

Here, we present three key ideas, aimed at improving detection of conserved regions in viral genome data that has low variability. We benchmark these ideas where appropriate. We then apply the ideas to the example of SARS-CoV-2. The first key idea, of weighting analysed sequence by the information it yields, is only possible following development of our previous scale-agnostic approach. The second key idea, of ranking loci by variability rather than using a raw variability measure when variability is low, is the natural extension to this scenario of a number of non-parametric tests in basic statistical theory. Whilst it does not rely upon the first idea of weighting, the second idea functions much more robustly in its presence. The third key idea, of attempting to remove interfering signal, functions independently from the first two ideas, but is of particular importance when there is low variability in all observed signals.

## Results

### Improving the signal-to-noise ratio: weighting data by information content, ranking skewed data, and accounting for interfering signals

When investigating sequences at the nucleic acid level, a major cause for sequence conservation in coding regions is the need for amino acid conservation. To detect other causes of nucleic acid conservation, any method used must account for amino acid conservation. Our method is based upon such a method^1^. That underlying method starts with an alignment of viral sequences, and for each codon within a gene allocates a score equal to the sum of the Hamming distances at that codon between every possible pairing of sequences in the alignment. To take amino acid conservation into account, that score is then normalised by dividing by the score that would have been given for a set of codons encoding the same amino acids, but with codon usage equal to the overall codon usage of the genome. To evaluate for regions of sequence conservation, this original method simply considered moving averages across a sliding window of contiguous codon loci. We previously improved upon the use of moving averages, by instead taking the normalised distance sums, further normalising them to have mean zero and standard deviation one, and then considering the problem as equivalent to finding the “steepest” descent in a random walk, where “steepest” means the most extreme *Z*-statistic, and tends towards finding contiguous regions of codons with higher-than-average conservation^6^.

**Figure 1:**
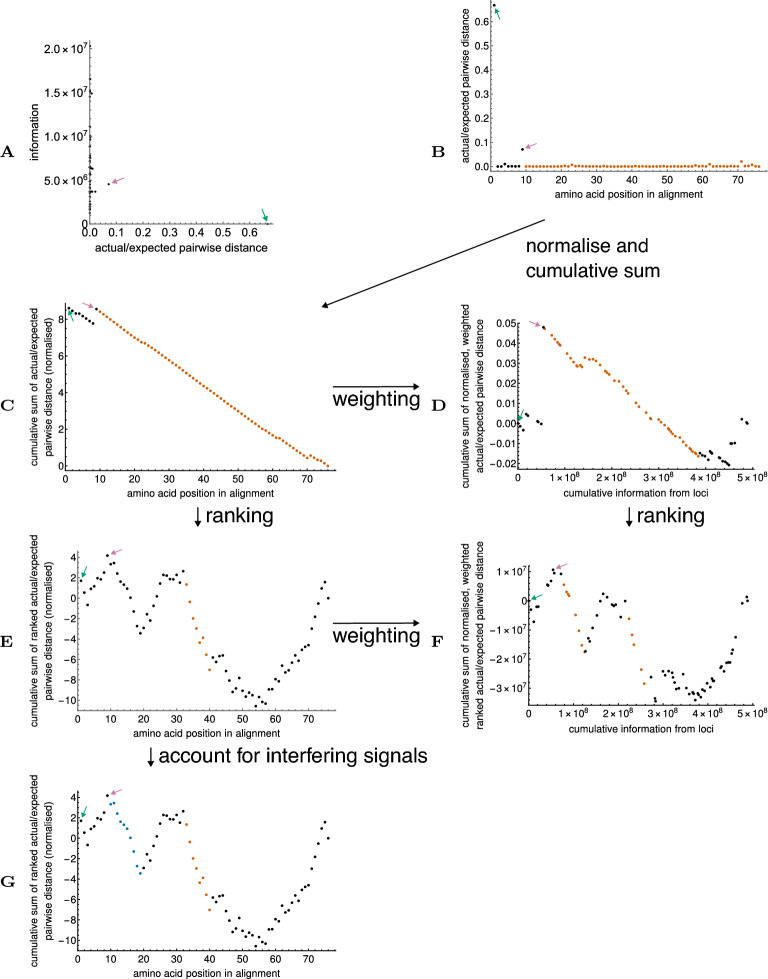
Example of how our gene alignment analysis works, applied to the E gene of SARS-CoV-2. (**A**) Information on nucleotide conservation (separately from amino acid conservation) obtained per codon in the alignment, versus the apparent nucleotide-level codon conservation measure, actual/expected pairwise distance (low is more conserved: see text for details). Each dot contains the results for one codon in the alignment. In this case, one locus with apparently low nucleotide conservation contains low information (green arrow), because all the information on conservation is explained by amino acid conservation (in fact this is the initiation codon for the gene). Another locus (pink arrow) has relatively low apparent nucleotide conservation, and moderate information content. (**B**) Apparent nucleotide-level codon conservation measure versus position of codon in the gene alignment. The dots corresponding to the codons discussed in panel (**A**) are marked with corresponding colour arrows. The orange highlighted region is deemed significantly conserved by the algorithm. (**C**) As (**B**), but with results normalised to have mean 0 and standard deviation 1, and a cumulative sum taken. This allows evaluation of the data using a random walk model^6^, where the steepest descent by *Z*-statistic corresponds to the most conserved region in the gene. Significance of the steepest descent can be evaluated by bootstrap resampling; any region with a bootstrap *p*-value exceeding 0.05 is highlighted in orange. However, the artefact created by having two outlier values means there is a single steepest descent covering almost the entire gene. (**D**) Effect of weighting the contributions from individual codons by information content. The first codon in the gene now has weight zero, and its artefactual contribution to the appearance of conservation in the rest of the gene is removed. The second locus with low conservation still has an artefactual contribution that makes the run of loci after it appear conserved. (**E**) Effect of ranking the conservation measures so that the descent statistic approaches a normal statistic faster. The artefact from the two outlier values is removed, and a more accurately delineated conserved region is identified. (**F**) Effect of combining weighting and ranking of conservation measures. A second conserved region, visible in (**E**) but not deemed significant there, is identified. (**G**) Effect of the procedure that accounts for potentially interfering signals in the data (see text), applied to the ranked (but not weighted) data. The second region identified in (**F**) can be identified at low confidence. For more information on the biological relevance of the identified regions, see “Application to SARS-CoV-2, to SARS-CoV, and biological implications”.

These previous methods, plus related methods, still all suffer from the implicit assumption that each codon yields the same amount of information on nucleic acid conservation (as opposed to amino acid conservation). This assumption is untrue: for example, where amino acid conservation requires that a single codon be used at a locus (as is seen at methionine loci, for example), all the conservation can be explained at the amino acid level, and so no extra RNA conservation independently of the amino acid conservation can be distinguished. Varying levels of information on nucleic acid conservation may be obtained from other loci, depending upon the degree of amino acid constraint, the number of codons that generate the amino acid(s) seen, and bias in codon usage. An example of the extent to which the information content of individual loci may vary is given for the SARS-CoV-2 E gene, in Fig. 1A.

The first of the key methodological improvements that allow us to find regions of nucleic acid conservation is to apply a weighting to each locus, with weights proportional to the information that locus gives us on nucleotide conservation beyond what is required for amino acid conservation (Fig. 1D,F; see “Methods” for details of approach). For organisms with high inter-sequence variability, useful results can be gained without this weighting, because there is sufficient overall information in the data. However, in organisms where our data have low overall inter-sequence variability, such as SARS-CoV and SARS-CoV-2, this step is key to minimising artefact from loci where amino acid conservation accounts for all possible nucleic acid conservation and so the apparent additional nucleic acid conservation is reported as zero.

The second of the key methodological improvements arises from recognising that although the steepest descent in our random walk framework tends towards being normally distributed, this central limit is not always closely approached in data from emerging or highly adapted organisms. The data from such organisms appears highly skewed, because at most loci there is high nucleotide conservation, making the few loci where variation has occurred (by chance or by selection for individual locus variability) into outliers (Fig. 1A,B). These outliers consequently disproportionately affect any analysis.

The solution to this issue of skewness is to move from a parametric test to the appropriate non-parametric equivalent, that is, by using ranked data (see “Methods” for details of approach). The effects of applying such a ranking scheme can be seen in Fig. 1E,F.

The third of the key methodological improvements deals with the situation where a gene contains more than one significantly conserved region. Our analysis implicitly uses a hypothesis testing framework, comparing a null hypothesis that the most conserved region observed occurs by random variation, versus an alternative hypothesis that the most conserved region observed is significantly more conserved than the background. The potential issue with this framework is that in a gene with more than one significantly conserved region, the “background” to the first significantly conserved region includes the signal from subsequent conserved regions. This means that where there is more than one signal, there is a slight reduction in ability to find those signals^6^.

To deal with this potential issue, we implement an addition to our search algorithm that, when the most conserved region in a sequence is determined not to be significantly conserved, re-runs the analysis with the next most conserved region removed, in case the next region is causing interference (see “Methods” for details of approach). Regions found to be significant after such a re-analysis are marked as such, as a caution that the false positive rate in such regions may be slightly higher. An example of the output from this procedure can be seen in Fig. 1G.

We have benchmarked the weighting and ranking processes using well-characterised housekeeping genes from *Escherichia coli*^9^. We have benchmarked the procedure for dealing with interfering signals using simulated data, and by applying the procedure to the same *E. coli* housekeeping genes. All results from benchmarking may be found in the Supplementary Information S1.

### Application to SARS-CoV-2, to SARS-CoV, and biological implications

The advantages of the refinements we describe above are especially visible in the very low genetic variability setting seen in the still-emerging virus SARS-CoV-2. Using our newly developed techniques, we undertook an analysis of 5,121,523 SARS-CoV-2 genomes downloaded from the GISAID database^10^. Because our method analyses sequence variability at individual loci rather than e.g. attempting to work with the phylogenetic structure, it is ideally suited to analyses of the full variability in such a large dataset. We analysed each of the main open reading frames, plus the sequences encoding the individual protein products of the 1a/1ab frames (Figs. S21–S28). We were motivated to examine the individual 1a/1ab protein product sequences because the relative non-conservation within the first 4100 nucleotides of the large open reading frames (corresponding to the nsp1 and nsp2 regions plus part of the nsp3 region) led to much of the remainder of those frames being marked as significantly conserved by the algorithm (a true, but unhelpful, positive result). In order to see smaller regions of relative conservation within other regions, it was therefore more informative to consider the individual protein sequences separately (Figs. S25–S28).

After analysing SARS-CoV-2 sequences, we then proceeded to analyse 119 sequences of SARS-CoV, as a cross-check in a related virus to find analogous conserved regions. Analysing SARS-CoV alone represents an extremely challenging scenario in terms of information, because a small number of sequences from human infections were obtained over a short evolutionary time, before the SARS outbreak was terminated. Although SARS-CoV sequences can be aligned and analysed alongside SARS-CoV-2 sequences (and such analysis has been performed successfully^11^), the risk of such an approach is that conserved features unique to one virus, or found in different genomic locations between the viruses, may be obscured.

A summary of the characteristics of regions found to be significantly conserved in SARS-CoV-2 may be found in Tables 1 and 2, and a summary of the characteristics of regions found to be significantly conserved in SARS-CoV may be found in Tables S2 and S3. We discuss salient findings and issues below; additional findings are discussed in the Supplementary text S1.

**Table 1:** Summary of regions of significant conservation found in SARS-CoV-2 genes.

Gene	Order found	NC_045512.2 nt location	$\varvec{Z}$	$\varvec{p}$	Comment
1a	2	4355–6247	2.92	0.0001	See main text
	1	7127–13,480	3.53	$<0$.0001	See main text
1ab	3	4355–6247	2.80	$<0$.0001	See main text
	2	7127–21,546	3.25	$<0$.0001	Nests around 1st identified; see main text
	1	7493–17,984	4.21	$<0$.0001	See main text
S	2	23,087–23,395	2.54	0.0037	Adjacent to receptor-binding motif; conserved structure possibly required to stabilise variable adjacent region
	3	23,735–23,866	2.20	0.0394	Region overlaps with various ARTIC primers
	4	24,242–24,313	2.24	0.0191	Analogous region amongst conserved regions identified in SARS-CoV. $5^{\prime }$ region overlaps with v4/4.1 ARTIC primer
	1	24,446–24,690	3.74	$<0$.0001	Analogous region amongst conserved regions identified in SARS-CoV. Region overlaps with v4/4.1 ARTIC primer
3a	1	25,732–26,052	2.38	0.0013	Reason for conservation unclear
	2	26,119–26,148	1.79	0.0065	Reason for conservation unclear
3b	1	26,234–26,254	1.95	0.028	Likely TRS-B for forming E sgRNA. Likely TRS-L dependent noncanonical for a high-efficiency non-canonical sgRNA^12^. Region overlaps with v4.1 ARTIC primer
E	2	26,278–26,301	1.54	0.0253	Likely TRS-B for a high-efficiency non-canonical sgRNA. Possible TRS-B for forming alternate M sgRNA^12^. Incomplete overlap at $3^{\prime }$ end with a conserved region identified in SARS-CoV. Region overlaps with v1/2 ARTIC primer
	1	26,341–26,355	1.68	0.0272	Region overlaps with v4/4.1 ARTIC primer
M	1	27,159–27,191	2.18	0.0059	Out-of-frame internal open reading frame previously identified by ribosomal profiling^13^. Region overlaps with v4/4.1 ARTIC primer
7b	2	27,771–27,788	1.40	0.0191	Possible TRS-B for forming alternate 7b sgRNA^12^. Overlap with regions identified in SARS-CoV but see note in SARS-CoV table
	1	27,813–27,830	1.47	0.0492	Region contains first AUG for alternate 7b sgRNA after possible TRS-B above. Overlap with regions identified in SARS-CoV but see note in SARS-CoV table
N	1	28,463–28,594	1.65	$<0$.0001	Overlaps region found in 9a; region overlaps with v4.1 ARTIC primer
9a	2$^{*}$	28,410–28,457	1.63	0.0121	Reason for conservation unclear
	1$^{*}$	28,488–28,577	1.83 (1.57)	0.0018 (0.1304)	Overlaps region found in N; region overlaps with v4.1 ARTIC primer. Reason for conservation unclear
9b	1$^{*}$	28,917–28,955	1.52 (1.24)	0.0316 (0.5678)	Reason for conservation unclear

The list is presented in gene order, then $5^{\prime }$ to $3^{\prime }$ within the gene. Within each gene, the order in which the algorithm identifies regions (starting with most highly conserved by our criteria) is listed in the table. Genes not listed do not contain regions found by our algorithm to have significant conservation.

*sgRNA* subgenomic RNA, *TRS* transcription-regulatory sequence (-B=body).

$^{*}$ Denotes a region only found by excluding a potentially interfering signal. *Z*- and *p*-values in parentheses denote values prior to removal of the next most significant signal. If parenthetical values are absent, then such a signal was removed in an earlier step only.

**Table 2:** Summary of regions of significant conservation found in individual proteins of SARS-CoV-2 1a/1ab.

**Region**	**Order found**	**NC**_**045512.2** **nt location**	$\varvec{Z}$	$\varvec{p}$	**Comment**
NSP2	1	974–1042	2.36	0.0099	$3^{\prime }$ region overlaps with ARTIC primers
	2	1688–1720	2.19	0.0306	Reason for conservation unclear
NSP3	3$^{*}$	4598–4750	2.26 (2.24)	0.0446 (0.0589)	$3^{\prime }$ region of SARS-unique domain (SUD). Region overlaps with ARTIC primers
	2	7127–7726	2.42	0.0237	Region contains transmembrane domain 2. Reason for conservation unclear
	1	8396–8545	3.12	0.0002	Near C-terminus of Y domain. Reason for conservation unclear
NSP4	1$^{*}$	9812–9847	2.06 (2.02)	0.0412 (0.0778)	Region overlaps with v1–3 ARTIC primer
NSP6	2	11,174–11,194	2.06	0.0234	N-terminal region of 3rd transmembrane domain. Analogous region amongst conserved regions identified in SARS-CoV
	1	11,675–11,740	2.11	0.0271	C-terminal region of protein. Region overlaps with ARTIC primers
NSP7	1$^{*}$	11,843–11,914	1.76 (1.60)	0.0098 (0.0796)	Putative negative-sense ORF; see Supplementary text S1. $3^{\prime }$ end analogous to part of a conserved region identified in SARS-CoV. Region overlaps with v1–3 ARTIC primer
	2$^{*}$	11,987–12,019	1.37 (1.33)	0.0487 (0.1185)	Stem-loop structure predicted, more stable in negative-sense via non-canonical GU pairs. Analogous to part of a conserved region identified in SARS-CoV.
NSP9	1$^{*}$	12,977–13,003	1.76 (1.70)	0.0203 (0.1061)	Possible pseudoknot with this region and region in nsp10; frameshift candidate; see Supplementary text S1
NSP14	2$^{*}$	18,190–18,237	2.01 (1.99)	0.0456 (0.0762)	Reason for conservation unclear.
	1	19,189–19,485	2.56	0.0014	Reason for conservation unclear
NSP16	2	20,845–20,928	1.94	0.0404	Putative packaging signal^14^; see main text. Region overlaps with v1–3 ARTIC primer
	1	21,469–21,507	2.16	0.0146	Reason for conservation unclear

The list is presented $5^{\prime }$ to $3^{\prime }$. Within each protein, the order in which the algorithm identifies regions (starting with most highly conserved by our criteria) is listed in the table. Proteins not listed do not contain regions found by our algorithm to have significant conservation.

$^{*}$ Denotes a region only found by excluding a potentially interfering signal. *Z*- and *p*-values in parentheses denote values prior to removal of the next most significant signal. If parenthetical values are absent, then such a signal was removed in an earlier step only.

#### Characterisation of previously described putative packaging signals in SARS-CoV-2 nsp16 and SARS-CoV nsp15 regions

**Figure 2:**
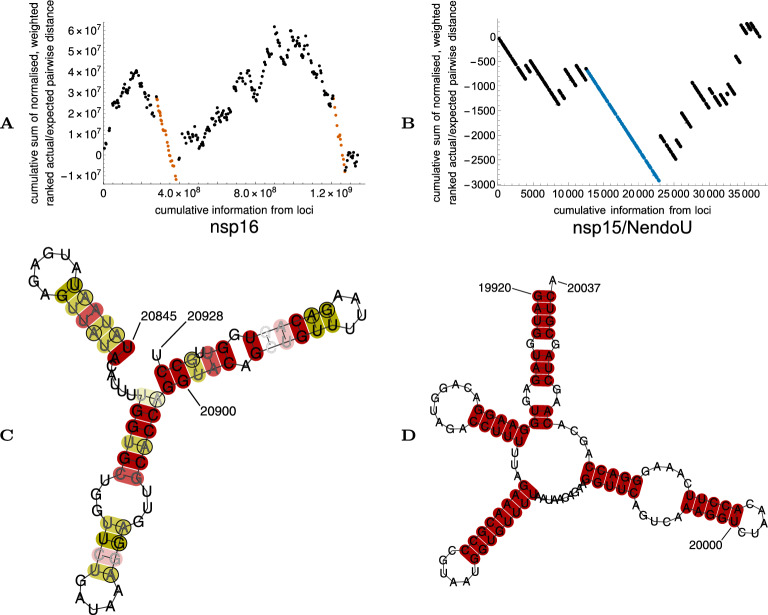
(**A**) Output from analysis of SARS-CoV-2 nsp16 region, with regions deemed significant highlighted in orange: the region coincident with the packaging-associated region of reference^14^ is the left-hand highlighted region. More detail may be found in Table 2. (**B**) Output from analysis of SARS-CoV nsp15 region, with region deemed significant highlighted in blue. Note how, because there is substantially less information from sequence data available for SARS-CoV versus SARS-CoV-2, that despite the use of weighting and ranking pairwise distance ratios, there is much less granularity in delineating the extent of the conserved region. (**C**) Output of RNAalifold analysis of the conserved region of SARS-CoV-2 coincident with the packaging-associated region of reference^14^. As the memory requirements for including every sequence as input to the folding algorithm are too great, just one representative of each sequence seen in the region is included. Base pairs are highlighted in deep/mid/light red as all/all but one/all but two sequences are capable of forming the pairs shown. Base pairs are highlighted in deep/mid/light yellow as all/all but one/all but two sequences are capable of forming the pair shown or one other base pair (including GU pairs). A set of conserved stem-loops is visible. RNAalifold was used with input options disallowing lonely pairs, allowing G-quadruplexes, and with the ribosum scoring matrix enabled. Nucleotide numbering follows the reference genome (NC_045512.2). (**D**) Output of RNAalifold analysis of the conserved region of SARS-CoV coincident with the packaging-associated region of reference^17^. All available sequences are used as input to the folding algorithm, although all are identical in the given region. The folded region includes all of a stabilising basal stem predicted from folding the entirety of the conserved region predicted by our algorithm (see Fig. S95 for detail). Nucleotide numbering follows the reference genome (AY274119).

The second region our algorithm identifies as significantly conserved within the nsp16 sequence of SARS-CoV-2 (NC_045512.2 nucleotide reference 20,845–20,928) lies within a region previously shown to be associated with packaging RNAs into virus-like particles^14^. When we folded representative sequences from our region with RNAalifold^15,16^, we found a set of conserved stem-loops (Fig. 2).

We note that regions identified by our algorithm can be slightly longer or slightly shorter than regions with biological significance owing to low information content at extreme loci and/or random variability. Manual inspection of the reference genome (NC_045512.2) close to the region identified demonstrates a set of nine complementary base pairs (nucleotides 20,835–20,843 and 20,899–20,907) with some similarity to the base of the stem-loop previously reported as associated with a packaging signal in Murine Hepatitis Virus (MHV)^18,19^. Motivated by this, we produced folds of a series of regions with slightly different lengths from our predicted region, as a sensitivity analysis of the predicted structures. Similarly to a previously reported discrepancy for minimum free energy folding of the MHV region^20^, most of the predictions for a larger region have more than one stem-loop; one stem-loop in the middle of the region remains consistently predicted (Fig. S58). We postulate that this stem-loop (or possibly one of the two adjacent stem-loops) is a candidate for the packaging signal thought to exist in the region.

The region our algorithm identifies as significantly conserved in the SARS-CoV nsp15 sequence (AY291451 nucleotide reference 19,920–20,195) also overlaps with a region shown to be associated with viral RNA packaging^17,21^. The more recent work^17^ provides experimental evidence to constrain the key packaging signal to the region containing nucleotides 19,881–20,031. Our fold of our predicted conserved region (Fig. S95) predicts a series of conserved stem-loops, and the predicted fold of the region 19,920–20,031 within this larger fold again contains a three stem-loop structure. This prediction provides mutual corroboration between SARS-CoV and SARS-CoV-2 that we have improved the characterisation of these viruses’ packaging signals and their structural basis. However, when as a sensitivity analysis a fold is attempted of a more truncated region, the three stem-loop structure in SARS-CoV is lost and only one of the stem-loops is consistent (Fig. S96). The resulting lower confidence in the structural prediction arises both from the lower confidence in the predicted boundaries of the region to fold, and because the lack of covariation in the few available SARS-CoV sequences makes it harder for the folding algorithm to select which base pairs occur in nature.

A larger region containing the conserved region identified in SARS-CoV-2 by our algorithm has been postulated as a negative-sense open reading frame^22^. Such a possibility would be a plausible alternative explanation for the conservation we find, although the boundaries of the conserved region we find combined with the biological corroboration above seem more consistent with a packaging signal.

#### Transcription-regulatory sequences leading to alternate subgenomic RNAs

Some of the smaller regions identified by our algorithm with high confidence in SARS-CoV-2 have been identified as forming the $5^{\prime }$ regions of “body” sequences, or as forming the $3^{\prime }$ regions of non-canonical “leader” sequences, in subgenomic RNAs (sgRNAs)^12^. These regions that are identified as conserved are therefore correctly placed to function, respectively, as the transcription-regulatory sequences-body (TRS-B) leading to RdRp pausing and switching to the $5^{\prime }$ TRS-leader during negative-strand synthesis, or as non-canonical mid-genome leader sites (TRS-L-dependent noncanonical).

The region we identify within 3b, nucleotides 26,234–26,254, is likely the canonical TRS-B for forming E sgRNA (Kim et al.^12^ recorded 149,744 sgRNAs corresponding to this TRS-B in their experiments). However, it is also likely to be the TRS-L-dependent noncanonical for a non-canonical sgRNA produced with higher efficiency than the E sgRNA (Kim et al.^12^ recorded 800,532 sgRNAs missing genomic nucleotides 26,257–26,282). This non-canonical sgRNA would result in a frameshift in the E protein, leading to a truncation. The functions of both sgRNAs may be important.

The region identified second within E, nucleotides 26278–26301, is likely the TRS-B for the non-canonical sgRNA mentioned above. In addition, it is a candidate low-efficiency TRS-B for M sgRNA (Kim et al.^12^ recorded just over 900 sgRNAs corresponding to this TRS-B), and an analogous conserved region is identified within SARS-CoV.

The two regions we identify within 7b, nucleotides 27,771–27,788 and 27,813–27,830, reflect a low-efficiency TRS-B that appears to yield a truncated form of 7b (Kim et al.^12^ recorded around 15,000 sgRNAs corresponding to this TRS-B) and the region containing the corresponding initiation codon, respectively.

False-positive results from our algorithm tend to be seen more commonly in shorter sequences in our benchmarking (consistent with what one would expect from random variation in the null setting), and so care must be taken when making inferences about conserved functions of the short sequences identified above. However, the number of sgRNAs observed corresponding to the likely canonical E, non-canonical E, and truncated-7b TRSs noted above give strong corroborative evidence for our observed conservation and for the conservation at the additional truncated-7b initiation site.

#### Overlapping alternate open reading frame

The region we identify within M, nucleotides 27,159–27,191, has been identified as an overlapping alternate open reading frame in ribosomal profiling experiments by Finkel *et al.*^13^. Where there are overlapping alternate open reading frames, the higher conservation in the wobble positions in the frame being analysed can lead to the region being identified as conserved. The previously identified alternate open reading frame terminates $3^{\prime }$ of the M termination codon; the region we identify terminates with M because our analysis is limited to the region within the gene. We do not identify the other alternate open reading frames identified by Finkel et al. The region we do identify is the one with the greatest number of reads in these ribosomal profiling experiments, and it would be reasonable to infer that it is therefore the region under greatest conservation pressure and hence easiest to find.

#### Primers commonly used in sequencing

Our algorithm can identify any conservation seen in a dataset, even when that conservation is artefactual. One of the potential risks in the SARS-CoV-2 dataset is inadvertent identification of binding regions for primers used in common sequencing workflows (that is, conservation is artefactual because less conserved sequences do not make it through the sequencing pipeline). We have highlighted in our summary where identified conserved regions overlap with primer binding sites in the commonly-used ARTIC sequencing protocol^23^. As the protocol uses overlapping amplicons of around 400 nucleotides, any conserved region greater than 400 nucleotides long, and many just shorter than this, will contain a primer site, so we only highlight the presence of primer sites fully contained within regions shorter than 250 nucleotides that are identified as conserved. The frequency of such primer-binding sites is high and not all such sites are identified as conserved: furthermore, primers are designed to avoid variable regions, so are more likely to be found in regions that are truly conserved, especially in later versions of the ARTIC protocol. In consequence, where a conserved region is identified as overlapping a primer binding site, this should be viewed as a possible contribution to observed conservation, but not as the only explanation.

## Discussion

We have presented, and demonstrated the application to biological sequences of, a new technique customised towards maximising the signal-to-noise ratio when analysing sequences with low signal and low noise. This technique can complement other techniques to investigate newly-emerged pathogens, as well as pathogens where the amount of information on sequence diversity will remain forever-limited. The application we have demonstrated focusses towards RNA conservation in coding regions; however, by altering the distance and weight measures used, it would be straightforward to amend the technique to apply to non-coding nucleic acid or to amino acid sequences. The packaging signal characterisation seen in SARS-CoV-2 demonstrates how this technique can assist in isolating key lifecycle features in RNA viruses; isolating such features is a precursor to developing drugs targeted against them.

Through our demonstration of our technique with both SARS-CoV and SARS-CoV-2, we have shown how our algorithm can work to analyse around a hundred sequences or several million sequences. Because the main input to the algorithm is a list of codon frequencies, the number of sequences involved does not substantially affect the performance of the algorithm. In fact, with more sequences the measure of conservation used varies more between loci. Greater variability means the algorithm can more easily limit which pairs of loci need to be analysed as possible start and end points for conserved regions^6^. As a result, in practice the algorithm runs slightly faster when analysing data from more sequences. The main part of the algorithm itself takes a few hours to run on a desktop computer, and the main factor affecting complexity is the nucleotide length of the individual genes analysed. Pre-processing to generate codon frequencies of course takes longer in the case of larger datasets. In the case of very large datasets, such as SARS-CoV-2, careful memory management is required, and data must be stored in a way that minimises floating point errors. Overall, our algorithm has substantial speed and memory advantages over exhaustive tree-based approaches on large datasets, at the expense of removing the ability to investigate evolutionary history. Its main advantage over many approaches designed for use on desktop computers is that it still uses all of a large dataset for the main analysis, rather than attempting to select “representative” sequences to save memory and processor time.

Our algorithm retains the advantage of its predecessor^6^ of being scale-agnostic in determining regions of conservation. This increases its ability to find truly unknown features of interest, which may lack analogues in host organisms and therefore have reduced risk of toxicity as drug targets. However, two limitations ensue. The first is that if the length scale of a feature of interest being sought is known, then this algorithm has reduced sensitivity in comparison with algorithms tuned to the length of the feature of interest. Secondly and more importantly, where the difference in conservation between signal and background is relatively small, random variation may lead to the inclusion or exclusion of some codons at the end of a feature of interest. This artefact most likely explains why the conserved features we identify in SARS-CoV are in general longer than those identified in SARS-CoV-2: because of the extremely low variability seen in the SARS-CoV dataset, it is likely that the algorithm has called as conserved some non-critical regions contiguous with regions whose conservation is critical to the virus’s lifecycle. What is surprising, given the low variability even of the SARS-CoV-2 dataset and the extremely low variability of the SARS-CoV dataset, is that our algorithm identifies conserved regions in SARS-CoV at all and that some of them have clear analogues in SARS-CoV-2.

In general, our algorithm most easily finds longer contiguous regions of conservation, because of the statistical properties of the algorithm used. Other methods may therefore be more suitable for investigating point mutations. Our algorithm is particularly unsuited for detecting single amino acid variations that may be associated with fitness changes, as for example seen in SARS-CoV-2 variants, since in addition to being poorly suited for investigating nucleotide point mutations, our algorithm is implemented in a way that investigates nucleotide conservation/variation that cannot be explained by amino acid conservation/variation.

Our method retains the limitation seen in classical non-parametric tests that even using the non-parametric ranked test to overcome poor convergence to the central limit, our test statistics can still be far from normally distributed when ranks are tied. In most cases, presence of poor convergence can be diagnosed through inspection of graphical output, so that the method is useful notwithstanding this issue. To increase automation of the workflow and to give a more rigorous indication of whether the assumption of normality is valid, it will be necessary to develop a test for normality appropriate to this scenario. Such a test is likely to be analogous to existing tests for normality, in the same way that the non-parametric version of the analysis we have developed here is analogous to the non-parametric versions of classical statistical tests. The mathematics required to develop a test for normality should also allow formal demonstration of the rate at which the ranked test converges to a central limit.

What we have presented is a method for in silico analysis of biological sequences. In common with all such analysis techniques, its primary limitation for biological application is that it does not give the biological explanation for observed conservation, but highlights regions for further investigation. When the biological relevance of an identified region is clear, the technique facilitates further investigation. When the biological relevance is less clear, the technique only gives a very broad guide towards possible ways to proceed.

## Methods

### Information-scaled random walk

The idea underlying our work is that in an alignment of genetic sequences, the between-sequence diversity seen at a single locus *i* in the alignment can be represented by a number $x_i$. In this paradigm, as in previous work^1^ we consider as loci individual codons in coding regions of viral RNA, and at each locus generate a sum of all Hamming distances between pairs of codons for all possible pairings of sequences in the alignment (“pairwise distance”). This sum is normalised by dividing by the pairwise distance that would be observed if the observed codons were replaced so as to give the same amino acid frequency, but with the codon usage per amino acid matching the average usage across the entire gene or genome (“normalised pairwise distance”). In an earlier version of the method we present here^6^, that normalised pairwise distance was then taken to be the vertical deviation for that locus in a random walk where each codon was associated with unit horizontal deviation, following a method designed for assigning parentage to recombinant genes^24^. The overall walk was normalised to give mean vertical deviation of zero and variance of the vertical deviation of one, and an appeal to the Central Limit Theorem made to argue that the steepest descent by *Z*-statistic should be evaluated for statistical significance.

The key idea of this present work is that the earlier random walk paradigm can be refined by moving from the unit of horizontal movement in the random walk being the number of loci, with each locus counting equally, to the unit of horizontal movement being the information gained from each locus. For example, at a locus where the amino acid usage is constrained to be methionine, no further information is gained by considering codon usage, since every sequence is constrained to use the codon AUG. Note that since the observed codon usage exactly matches the expected codon usage, the normalised pairwise distance here is 1. In contrast, at a locus where the amino acid usage is constrained to be glutamine (possible codons CAA, CAG), substantial extra information is conveyed by observing that the codons actually seen match the overall usage in the genome (say e.g. 1:1 ratio of each), despite the normalised pairwise distance being 1 as in the methionine case, since in the presence of strong nucleic acid constraint (say all observed codons being CAA) a normalised pairwise distance of 0 would be observed, which is not possible in the methionine case. Including the methionine case, where no information is gained, in the random walk unnecessarily dilutes the information it is possible to obtain from analysing the walk. We note that our method does not depend upon the underlying biology explaining the overall codon usage in the genome (for example whether there are multiple tRNAs corresponding to one amino acid leading to abundance-related codon bias, avoidance of codons with CpGs), just the measured resultant usage.

The appropriate way to account for the variability in information gained from each locus is to construct a walk where the horizontal distance moved per locus is equal to the information gained from that locus, and the normalised pairwise distance now defines the gradient of the step, so that the vertical distance moved in the walk is (information gained $\times$ normalised pairwise distance). In our example, this would result in the uninformative methionine locus effectively being excluded from the walk, since it would result in moving distance zero in both horizontal and vertical directions.

More formally, for each locus in the gene being analysed, we calculate the Shannon information, $-\log p$, where *p* is the probability of observing the given codon frequencies at that locus. Each amino acid seen at a given locus gives a contribution to *p* of

$$\begin{aligned} \frac{s!}{y_1! \ldots y_k!}\, p_1^{y_1}\ldots p_k^{y_k}, \quad \textrm{ where } \quad \sum _{j=1}^k y_j = s. \end{aligned}$$

Here, *s* is the total number of sequences with the amino acid at this locus, *k* is the number of codons that translate to the amino acid, $y_j$ is the observed number of sequences using codon *j*, and $p_j$ is the proportion across the gene (or genome) amongst loci coding for this amino acid that use codon *j*. That is, for each amino acid seen, a multinomial distribution of codon usage at the locus is assumed, with the multinomial probabilities being the observed frequencies at which the codons are used to encode the amino acid across the gene (or genome). For each amino acid seen at a locus in an alignment, the calculated probabilities are multiplied together to obtain *p*.

Next, we use the calculated Shannon information at each locus (indexed *i*) as a weight, which acts as a coefficient of the diversity value $x_i$. If the $x_i$ were drawn from independent, identically distributed (i.i.d.) standard normal distributions, with the weights independent of the sampled values, then weights could be applied directly as coefficients without further modification. However, motivated by our sequence diversity value, we note that not only have we not required that the $x_i$ be i.i.d. samples, but worse, the weight $w_i$ ($-\log p$ from above) associated with $x_i$ may correlate with the value of $x_i$. (We can illustrate the existence of a correlation by noting that the highest values of $w_i$ occur only at loci with highly conserved unusual codons, which implies a low value of $x_i$.) It is obvious that uniformly translating the $x_i$ so that they have a different mean does not commute with applying weightings $w_i$, meaning care is necessary if one wishes to weight the $x_i$ and translate them to have mean 0 and standard deviation 1. The appropriate translation to obtain a mean of 0 is

$$\begin{aligned} x_i \rightarrow w_ix_i - w_i \left( \frac{\sum _{l=1}^{n} w_lx_l}{\sum _{l=1}^{n} w_l} \right) , \end{aligned}$$

where *n* is the total number of loci, and hence the appropriate normalisation factor to obtain a standard deviation of 1 (preserving orientation) is

$$\begin{aligned} \sqrt{\left[ \frac{\sum _{l=1}^n w_l}{\left( \sum _{l=1}^n w_l\right) ^2 - \left( \sum _{l=1}^n w_l^2\right) } \right] \left[ \sum _{l=1}^n w_l \left( x_l - \left( \frac{1}{n}\sum _{m=1}^{n}x_m \right) \right) ^2 \right] } \quad . \end{aligned}$$

We now construct a walk, in which for each increment *i* of the walk, the horizontal displacement is $w_i$, and the vertical increment is

$$\begin{aligned} {\hat{x}}_i = \frac{w_ix_i - w_i \left( \frac{\sum _{l=1}^{n} w_lx_l}{\sum _{l=1}^{n} w_l} \right) }{\sqrt{\left[ \frac{\sum _{l=1}^n w_l}{\left( \sum _{l=1}^n w_l\right) ^2 - \left( \sum _{l=1}^n w_l^2\right) } \right] \left[ \sum _{l=1}^n w_l \left( x_l - \left( \frac{1}{n}\sum _{m=1}^{n}x_m \right) \right) ^2 \right] }} \quad . \end{aligned}$$

As in our previous work^6^, we are now in a position to treat the ${\hat{x}}_i$ as step sizes in a random walk, but here with non-uniform step sizes in both dimensions, the weight of the step acting as a coefficient to the step size in each dimension. Let $c_0, \ldots , c_n$ and $v_o, \ldots , v_n$ be partial cumulative sums, formed as follows:

$$\begin{aligned} c_0= & {} 0, \quad c_k = \sum _{i=1}^k {\hat{x}}_i \quad \text { for } 1\le k \le n;\\ v_0= & {} 0, \quad v_k = \sum _{i=1}^k w_i \quad \text { for } 1\le k \le n. \end{aligned}$$

The difference between any two cumulative sums $c_i$ and $c_j$ gives (for $i<j$) the weighted sum ${\hat{x}}_{i+1} + \ldots + {\hat{x}}_j$, with sum of weights $v_j-v_i$. As long as the terms in the sums represent a random sample of the steps in the walk, then the central limit theorem guarantees that

$$\begin{aligned} Z_{ij} = \frac{c_i-c_j}{\sqrt{v_j-v_i}} \quad \text { for } 0\le i < j \le n \end{aligned}$$

will approach a normal statistic. We can then determine $\max _{i,j:i<j}Z_{ij}$, which in our original problem will correspond to the most highly conserved run of codons (compared to chance) in a coding sequence.

We have described algorithmic approaches to optimise finding $\max _{i,j:i<j}Z_{ij}$ in our previous work^6^. We note that in contrast to this previous work, the walk we have now generated is closer in its characteristics to that of the standard Brownian bridge, which is better characterised analytically in the literature than is the walk we generated in our previous work. However, we retain the relatively fast bootstrap approach to calculating significance we have described previously.

We note that the weights $w_i$ are only defined up to a multiplicative constant (corresponding to the base of the logarithm in the definition of Shannon information), but that this is taken into account in the normalisation and in any case, multiplicative scaling of the *Z*-statistics by the same amount would not change their ordering.

### Ranking of diversity measure

The weighting technique we have described above is especially important for samples of sequences that do not explore a large portion of evolutionary space, as it improves signal-to-noise ratio in sample sets with low amounts of signal. However, in such samples, the rate of convergence of the test statistic to a normal statistic will still be slow, and for short subsequences (statistics $Z_{ij}$ with *i* close to *j* in the notation above) this may be particularly problematic, with most loci apparently conserved and walks dominated by a small number of variable loci. Ultimately, in this scenario the statistic risks simply picking out the longest run between observed random variation. The problem is a breakdown of the parametric assumption in the low variability scenario.

In the situation of a collection of conserved sequences, it is therefore appropriate to seek an appropriate non-parametric test. One obvious approach to take to generate such a test is to rank the diversity measures, and perform testing on the ranks rather than the measures from which they were generated.

When using rankings, the question of information content becomes even more important than for raw measures of diversity, because now, adding a collection of uninformative loci with intermediate ranked diversity will affect the relative ranks of all other loci. The most appropriate extension to our previous idea of weighting by information content is that for each diversity measurement, rather than allocating a ranking that is one greater than the next lowest diversity measurement, we instead allocate a ranking that is greater by the corresponding weight.

Formally, we begin by defining a function $R^{-1}$, which maps a sequence position *i* to its position in the ordering of all the $x_j$. That is, $R^{-1}(i)$ is the integer rank of $x_i$ amongst $x_1,\ldots ,x_n$, in the usual sense of the word “rank”. We define the lowest value to have rank 1 and the highest value to have rank *n*. At this stage we assume that no two $x_j$ share a rank (i.e. $R^{-1}$ is bijective); we shall deal with relaxing this assumption later.

We have called the defined function $R^{-1}$ because we wish primarily to work with its inverse, which we shall call *R*. *R* maps a position in an ordering to its corresponding sequence position. This definition allows us to construct a series of weighted ranks $w_{R(1)},w_{R(1)}+w_{R(2)},\ldots ,\sum _{j=1}^k w_{R(j)},\ldots$ for each position in the ordering (that is, position *k* in the ordering, counting from the lowest, has weighted rank $\sum _{j=1}^k w_{R(j)}$). With this notation, sequence position *i* has weighted rank $\sum _{j=1}^{R^{-1}(i)} w_{R(j)}$, and corresponds to a walk increment of $w_i\sum _{j=1}^{R^{-1}(i)} w_{R(j)}$. This may be normalised to yield a vertical walk increment of

$$\begin{aligned} {\hat{x}}_i = \frac{w_i \left( \sum _{j=1}^{R^{-1}(i)}w_{R(j)} \right) - w_i \left( \frac{\sum _{j=1}^{n} \left[ w_j \sum _{k=1}^{R^{-1}(j)}w_{R(k)} \right] }{\sum _{j=1}^{n} w_j} \right) }{\sqrt{\left[ \frac{\sum _{j=1}^n w_j}{\left( \sum _{j=1}^n w_j\right) ^2 - \left( \sum _{j=1}^n w_j^2\right) } \right] \left[ \sum _{k=1}^n w_k \left( \left[ \sum _{j=1}^{R^{-1}(k)} w_{R(j)} \right] - \left[ \frac{1}{n} \sum _{l=1}^n \sum _{j=1}^{R^{-1}(l)} w_{R(j)} \right] \right) ^2 \right] }} \quad . \end{aligned}$$

To this point, we have assumed that *R* represents a bijection, i.e. that no two sequence positions share the same position in the ordering of diversity measures $x_j$. Where there is such a tie, we allocate all tied positions to have the same weighted rank, which is defined by setting the increment from the preceding weighted rank in the order to be the mean of the weights of the tied positions. The image of the tied sequence positions in the ordering can be arbitrary within the ties, but so that it is well-defined, we may for example set $R^{-1}(i)<R^{-1}(j)$ when $i<j$ and $x_i=x_j$. Formally, when we have a degeneracy of the form $x_{i_1}=x_{i_2}=\ldots =x_{i_k}$, the weighted rank for each of sequence positions $i_1, \ldots , i_k$ is defined to be

$$\begin{aligned} \left( \sum _{j=1}^{R^{-1}(i_1)-1}w_{R(j)}\right) +\frac{1}{k}\left( \sum _{j=1}^k w_{R(i_j)}\right), \end{aligned}$$

and the corresponding walk increment for position $i_l$ is

$$\begin{aligned} w_{i_l}\left[ \left( \sum _{j=1}^{R^{-1}(i_1)-1}w_{R(j)}\right) +\frac{1}{k}\left( \sum _{j=1}^k w_{R(i_j)}\right) \right]. \end{aligned}$$

We note that, aside from replacing each diversity measurement $x_i$ with a rank $R^{-1}(i)$, the algorithmic handling of sequences using this modification is identical to previously (in essence, the diversity measure $x_i$ is simply replaced by a new measure $x_i^{\prime }=R^{-1}(i)$).

Where a very large number of tied ranks is present, we caution that the test statistic may not converge rapidly to its central limit. Similar limitations are seen in classical non-parametric tests, for example the Mann-Whitney U test, which assume that ranks are not tied to derive their limits^25^.

### Handling potentially interfering signals

We showed in our previous work^6^ that when a sequence of real numbers contains two “signal” subsequences with positions that have similar deviations from the mean values of the background positions, then the two signals interfere with each other and less of each signal is found, on average. This is an unsurprising consequence of significance testing comparing a putative signal with background, such that if the background also contains signal, the signal-to-noise ratio will be lower and the chances of finding the signal will be lower.

A straightforward way of increasing the sensitivity of any algorithm for picking up signal regions in such circumstances is to attempt to re-run the analysis after identifying and removing a potentially interfering second signal region. Such an approach clearly has a specificity cost. However, one might expect the specificity cost to be relatively small for a number of reasons. Firstly, removing the next-identified putative signal region is more likely to have a large effect on a significance calculation for the first region when the second region is a large, contiguous region, as would be more expected from a second signal and less from a piece of randomly varied background. Secondly, it is possible to mark regions that have been identified following this procedure, meaning this can be taken into account when further analysis is performed.

Our algorithm modification to implement this procedure is that when the most extreme descent in a dataset has been identified but found not to be significant, then its location is recorded, but it is excised and the analysis continued to determine the location of the next most extreme descent. That descent is then removed and the analysis repeated with the first region present. If the analysis finds the same descent as in the first step and it now crosses a significance threshold, it is noted, and the analysis is continued with the sequence remaining after the most extreme descent has been removed, in the usual way (and with the next most extreme sequence replaced).

We note that there are two ways analysis can cease immediately following this procedure. The first, straightforward, way is if the originally identified most extreme descent still fails to cross a significance threshold. The second way is if the most extreme descent identified following excision of the second most extreme descent is no longer identical to the original. This can occur if, for example, excision of the second region leads to a new, more extreme region formed by the regions adjacent to the second region. In this case, we work on the basis that if there is a piece of original background that is now more extreme than the putative signal, then this is an indication that the original signal was not significantly different from the background and stop.

### Benchmarking with *E. coli* housekeeping genes

To find appropriate *E. coli* sequences to analyse for benchmarking, we searched GenBank using the string ((“Escherichia coli”[Organism] OR escherichia coli[All Fields]) AND rsma[All Fields]) AND “Escherichia coli”[porgn] AND (bacteria[filter] AND (“4500000”[SLEN] : “999999999999999999”[SLEN]) AND (“0001/01/01”[PDAT] : “2021/12/20”[PDAT])). We downloaded sequences in gene-annotated format and then selected (into separate files) sequences corresponding to the housekeeping genes adk, icd, fumC, recA, mdh, gyrB, and purA (i.e. the genes of the Achtman multilocus sequence typing schema^9^). Sequences that did not begin ATG and end with a termination codon, and sequences containing ambiguous nucleotide characters, were omitted from analysis. A list of accession numbers of sequences used in each gene may be found in Tables S4–S10.

Because sequences were highly conserved, it was possible for each gene to undertake manual curation and alignment of sequences. A list of the curation steps undertaken may be found in Table S11.

Aligned sequences corresponding to the individual genes were then analysed using the procedure detailed above, for each permutation of normalised pairwise distances being unweighted/weighted by information, using raw/ranked normalised pairwise distances, and including a check for potentially interfering signals. Output may be found in the Supplementary Information (Figs. S1–S3, S6, S8, and S11–S18).

### Analysis of SARS-CoV-2 sequences

We downloaded sequences from the GISAID database for analysis. Sequences downloaded were all those uploaded up to and including 17th March 2022, with the host set to human and using the “Complete” and “High coverage” options. The rationale for these options is that our analysis aims to understand selection pressures on SARS-CoV-2 in human hosts, and to use our algorithm we ideally need entire genes (or regions for analysis) to have been sequenced unambiguously. A list of downloaded sequences, with submitter attributions, may be found in GISAID EPI_SET 20220720bs^26^.

Nucleotide sequences were aligned to the Wuhan-Hu-1 reference sequence (NCBI reference sequence NC_045512.2, identical to GenBank reference MN908947) using MAFFT^27^ with the keeplength and addfragments options (and otherwise default options). Although aligning to a single reference sequence may generate less optimal alignments than a full alignment, and is done in a way that fails to account for nucleotide insertions, a full alignment was not computationally tractable and the level of conservation of the SARS-CoV-2 genomes resulted in a reasonable alignment with minimal information loss.

Genes and subgenomic regions for analysis were extracted by nucleotide reference in the alignment, taking into account the $-1$ frameshift in the 1ab gene.

Alignments of extracted regions were further curated by removing sequences containing ambiguous nucleotides, by removing sequences without stop codons in the final 30 nucleotide region of genes being analysed, by removing sequences with straightforward frameshifts (a number of gaps that is not a multiple of 3), and by removing sequences with $>5\%$ gaps.

The counts of each codon represented in the alignment at each locus were recorded, and pairwise distances were calculated as described above. For each gene, these pairwise distances were then analysed for significant regions using the procedure detailed above, for each permutation of normalised pairwise distances being unweighted/weighted by information, and using raw/ranked normalised pairwise distances. In each case, a check for potentially interfering signals was undertaken as described above and signals that were significant in the absence of a potentially interfering signal were recorded. Gaps in the alignments were handled as described in our previous work^7^.

Review of significant regions for biological relevance was undertaken only for the analysis with information weighting and ranked normalised pairwise distances, since these options would be expected to produce the fewest spurious results.

For each significant region of length $\le 5500$ nucleotides, one example of each possible sequence observed for the region was placed into an alignment and the alignment folded using RNAalifold^15,16^; a reverse complement of the alignment was similarly folded. To avoid exceeding RNAalifold’s memory limit, a single example of each possible sequence was used to reduce the memory required and folding was not attempted with regions of length $> 5500$ nucleotides.

Regions were manually reviewed for out-of-frame coding potential, and for known features of interest including regions known to affect transcription or translation, packaging signals, and other *cis*-acting sequences, plus regions with possibly artefactual conservation resulting from properties of common sequencing protocols.

Graphical output from this analysis not included in the main text may be found in Figs. S19–S60.

### Analysis of SARS-CoV sequences

We downloaded sequences from GenBank for analysis. The search term used was (“Severe acute respiratory syndrome coronavirus”[Organism] OR sars-cov[All Fields] OR sars[All Fields]) AND (viruses[filter] AND is_nuccore[filter] AND (“29000”[SLEN] : “30000”[SLEN]) AND (“0001/01/01”[PDAT] : “2018/12/31”[PDAT])). The date range in the search reflects the end of the SARS-CoV outbreak and the higher probability that sequences submitted after the end of 2018 are SARS-CoV sequences that have undergone multiple passages in culture, or that SARS-CoV-2 sequences would be included inadvertently. Output was reviewed manually. Animal-derived sequences, and sequences resulting from cell passage intended to generate genetic diversity, were removed. (Whilst the intention was to study genetic diversity, the diversity being studied is the diversity allowed within the constraints of infection of a human host, not the greater diversity seen in cell culture.) A complete list of accession numbers used may be found in Table S12.

Genes and subgenomic regions for analysis were extracted by nucleotide reference in the alignment, taking into account the $-1$ frameshift in the 1ab gene.

Alignments of extracted regions were further curated by removing sequences containing ambiguous nucleotides, and by removing sequences without stop codons in genes being analysed. Amino acid sequences were aligned using MUSCLE^28^ and back-translated to nucleotide sequences.

Codon counts, pairwise distance calculations, analyses for significant regions and review for biological significance was undertaken as described for SARS-CoV-2 above, save that all sequences (rather than one example of each possible sequence observed) were used to produce RNAalifold output.

Graphical output from this analysis not included in the main text may be found in Figs. S61–S96.

## Supplementary Information

Supplementary Information.

## Data Availability

The datasets analysed during the current study are available in the GenBank repository, https://www.ncbi.nlm.nih.gov/genbank/, accession numbers detailed in the supplementary information files, and in the GISAID repository, https://gisaid.org, accession numbers detailed at https://doi.org/10.55876/gis8.220720bs.

## Code availability

Code implementing the algorithms described in this study is available as a Mathematica notebook, from https://github.com/skittrall/RNAdescent.
