# Supplementary material for: A novel approach to finding conserved features in low-variability gene alignments characterises RNA motifs in SARS-CoV and SARS-CoV-2
Source: Sci Rep. 2023 Jul 26;13:12079. doi: 10.1038/s41598-023-39207-1 (PMC10372003; doi:10.1038/s41598-023-39207-1)
Supplement: Supplementary file 1 — Supplementary Information. [file 41598_2023_39207_MOESM1_ESM.pdf]

Supplementary Information for

A novel approach to finding conserved features in low-variability gene alignments characterises RNA motifs in SARS-CoV and SARS-CoV-2

Jordan P. Skittrall, Nerea Irigoyen, Ian Brierley, Julia R. Gog

E-mail: jps55@cam.ac.uk

This PDF file includes:

Supplementary text
Figs. S1 to S96
Tables S1 to S12
SI References

Supporting Information Text

1. Additional discussion of rationale for novel methodology, and results of benchmarking

A. Step 1: Effect of weighting by information content: helps but may be insufficient alone. Our first key idea is to mitigate the effect of loci with artefactually high apparent normalised variability (caused when a high degree of amino acid-level conservation means that there is little possible nucleotide-only conservation), by taking into account the amount of information each locus provides. We do this by weighting each locus by Shannon information (see the Methods for details).

To illustrate possible effects of such a procedure, we apply the procedure to two well-characterised housekeeping genes from *Escherichia coli*, which are highly conserved but have variable regions used in one Multilocus Sequence Typing (MLST) scheme(1). These genes – adenylosuccinate synthetase (purA) and fumarate hydratase C (fumC) – represent the genes used in the scheme with, respectively, the lowest and the highest polymorphism levels per nucleotide. These genes were originally selected for use in typing because their presence and function is highly conserved. They were chosen to allow phylogenetic analysis, but the precise regions within the genes used for such analysis were not chosen to extremise per-locus variability. This means that we might expect to see a mixture of relatively more- and less-conserved regions within what are overall comparatively highly-conserved genetic features, and so these genes are suitable for real-world benchmarking of our algorithm. (We briefly discuss in Section 2 of this Supplementary text known features of these genes, reproduced during our benchmarking.)

In all cases, a slight inverse correlation between the measure of locus variability and the information content of the locus (Fig. S1) simply reflects that the highest Shannon information only occurs in the loci with lowest variability (see Methods). In these low variability genes, any high variability locus may be regarded as an outlier, and many such loci have low information content.

In genes known to have low sequence variability, such weighting indeed reduces the number of outlier loci with apparent high variability (Fig. S2). Mitigating the effects of such loci can reduce identification of regions around these loci identified as conserved. However, paradoxically sometimes this procedure can in fact increase the chances of a region being determined to have low variability (possibly artefactually, possibly by improving signal-to-noise). This is because any remaining high-variability loci are consequently even more outlying than previously, meaning the majority of loci, which are low-variability, is more likely to contain a longer subsequence deemed to be statistically significantly conserved. In such situations, it is important not only to remove artefact, but also to ensure the overall analysis is not skewed by a small number of truly highly variable loci. To accomplish this goal, it is necessary to combine the weighting procedure with the ranking procedure described in the following subsection.

This step is a useful step regardless of the amount of underlying variability in the sequence, because in all cases it would be expected to increase signal-to-noise ratio, and so even in sequences with high variability where there is good delineation between signal and background, one would expect better definition of the boundaries of low variability (conserved) regions.

B. Step 2: Ranking variability. In sequences with low overall variability, single loci with high variability may cause the entirety of the rest of the sequence artefactually to appear highly conserved (Fig. S3). Although weighting by information content reduces this effect (since in general more variable codon usage for an amino acid is less informative than less variable codon usage), it is still possible for this effect to occur when using weighting. The mathematical explanation for the effect is straightforward: the algorithm determining conserved regions appeals to the central limit theorem to argue that the mean variability measure of a sequence tends to a normal distribution. However, when all but a few points in a sequence have very low variability, the distribution is so skewed that this normal limit is not sufficiently approached.

We can address this problem by replacing highly skewed measures of sequence variability with rank values – essentially a non-linear, order-preserving transformation to cause the data to tend faster to the normal limit. As this step results in information loss, it is possible to cause a reduction in sensitivity to detect conserved regions. This technique is therefore mainly appropriate for low-variability datasets that are far from a central limit. Indeed, although weighting will improve the signal-to-noise ratio, it may move the distribution further from the central limit.

We have benchmarked this technique by applying it to two different simulated distributions:

1. A standard normal variable.

2. An exponential distribution with mean 1.

We chose the exponential distribution to illustrate the effect of the technique on a skewed distribution, and the normal distribution to illustrate the effect of the technique on a distribution without skew (where, in fact, ranking positions simply causes information loss). In each case we simulated a "gene" consisting of 500 real numbers, with a "signal" of length 100 in the centre. We varied the "strength" of the signal region (the amount by which the mean of the distribution from which the signal was sampled was reduced).

The results of our benchmarking are shown in Figs. S4 and S5. As expected, we see that when the underlying distribution is not normal, it is more common to identify substantial non-signal regions as signal. This effect can be mitigated by replacing the values at individual loci with their respective ranks; however, the information loss this causes reduces our ability to identify signal regions correctly. It is therefore important to consider whether it is reasonable to analyse any given sequence using the parametric assumption that the central limit is closely approached. If it is reasonable, then using raw values in the analysis will be more sensitive for identifying signals.

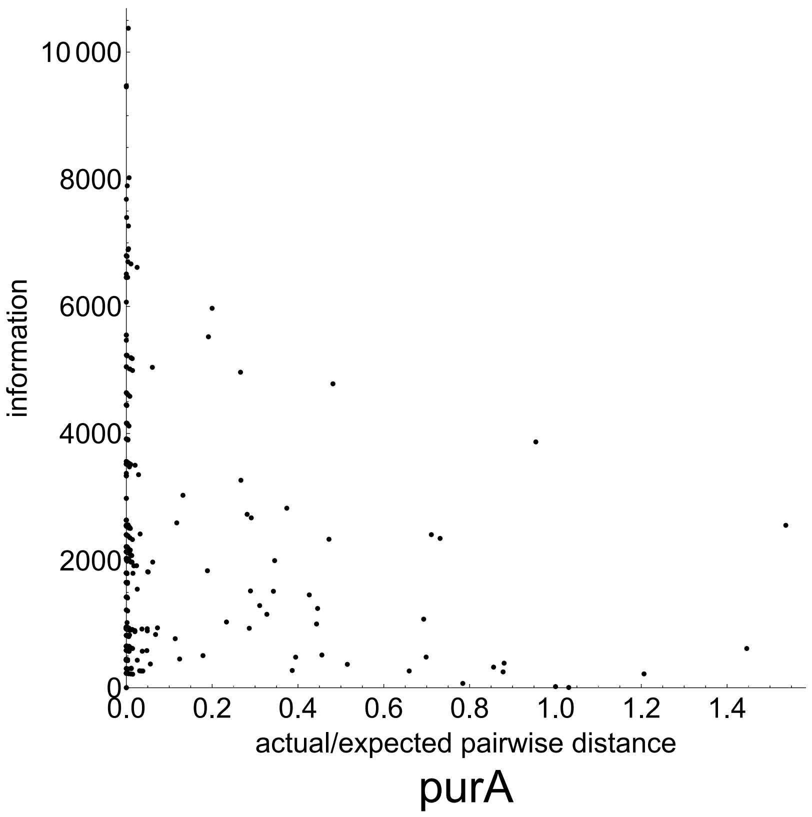

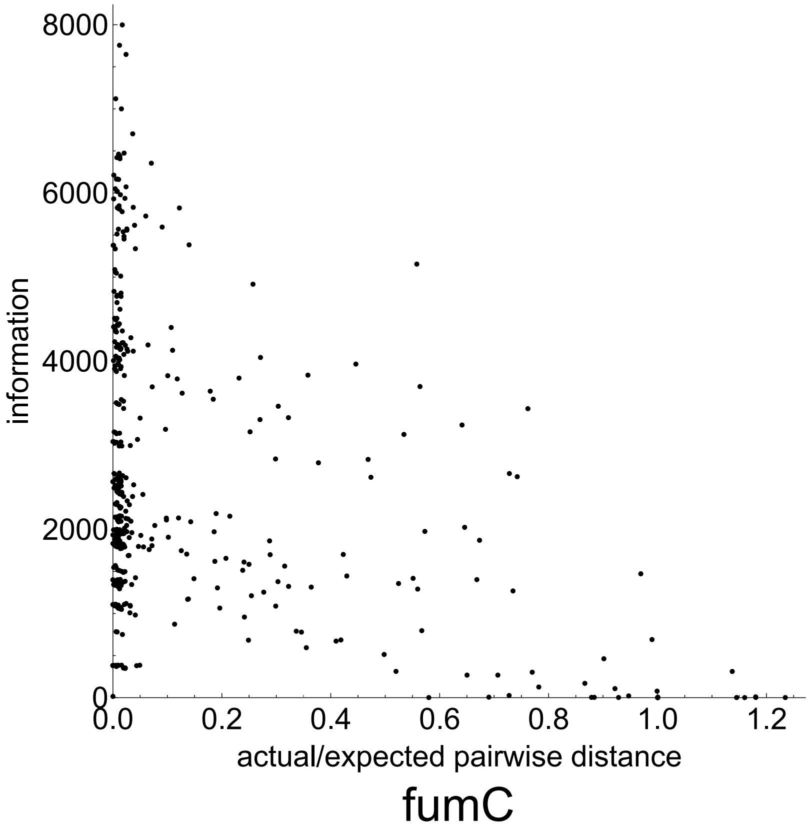

Fig. S1. Shannon information at locus versus measure of locus variability (actual/expected pairwise distance: see Methods for details), for two conserved housekeeping genes of *E. coli*, purA (relatively low polymorphism rate, top) and fumC (relatively high polymorphism rate, bottom). Throughout this work, each locus is a codon within a gene, although in principle different loci could be used with different measures of variability. Each plotted point represents the comparison of information/variability at a single locus. In both cases, most loci have low variability, although the information gained from each low variability locus can vary. High variability loci are rare and many give low information. The absence of high information, high variability loci reflects the high number of combinations of loci producing high variability, meaning the Shannon information cannot be high.

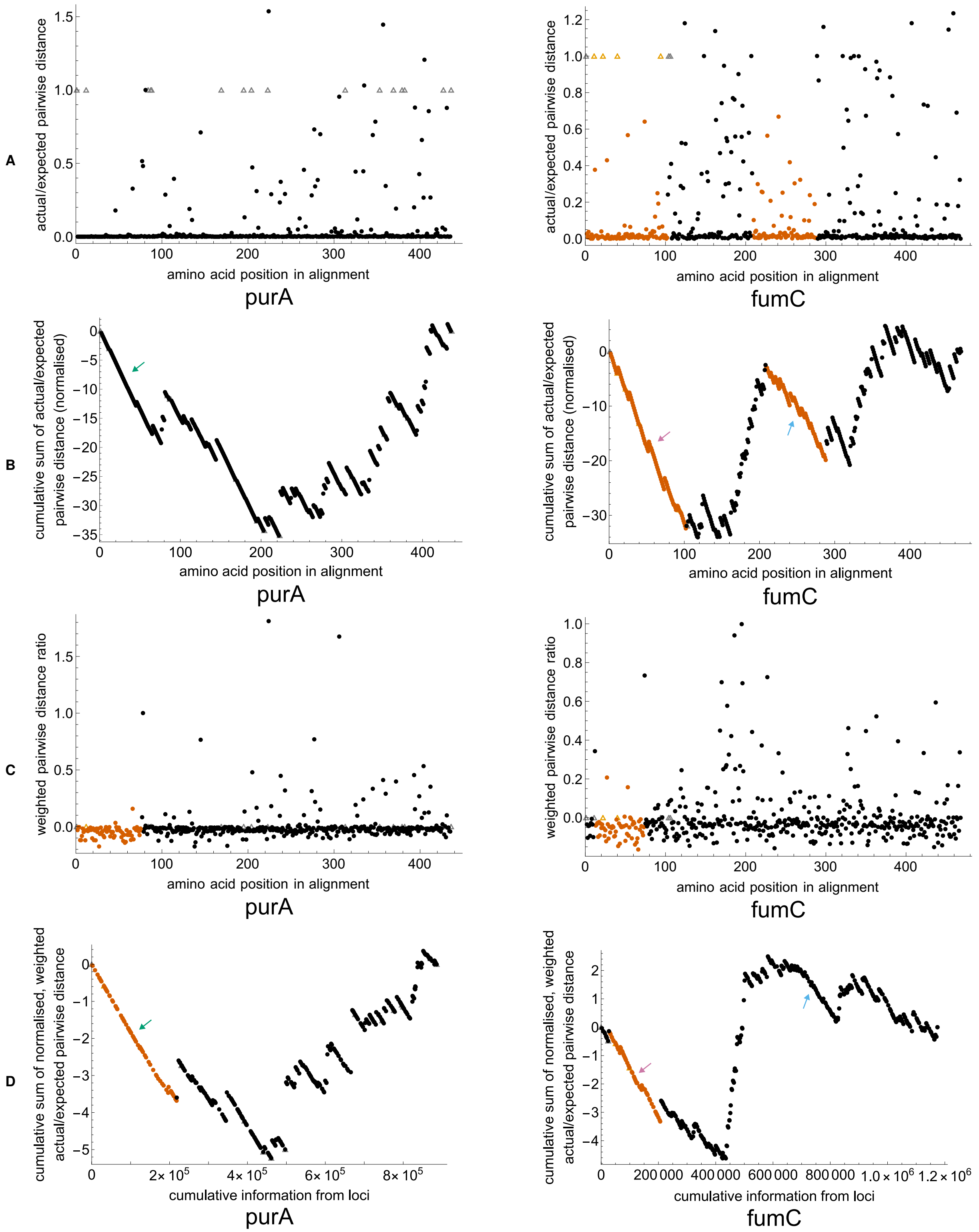

Fig. S2. Effect of weighting loci for information content in *E. coli* housekeeping genes purA (left) and fumC (right). In each case, variability measures are normalised to have mean 0 and standard deviation 1. (**A**) Raw variability measures (actual/expected pairwise distance: see Methods for details). (**B**) Cumulative sums of raw variability measures. (**C**) Weighted variability measures (see Methods for details). (**D**) Cumulative sums of weighted variability measures. Each plotted point represents a single locus (codon within the gene). Orange loci correspond to regions deemed to be significantly conserved by our algorithm. Grey and light orange loci plotted with open triangles yield no information on nucleotide conservation(2). Where raw variability measures are used, such loci are plotted with actual/expected pairwise distance=1 (since actual=expected pairwise distance), but are excluded from analysis. Where weighted variability measures are used, such loci are plotted with weight=0. It can be seen in both cases that weighting reduces the number of outlying, high variability loci contributing to the analysis. In the case of fumC, the effect is that one of the identified conserved regions is delabelled (blue arrows), and the other identified conserved region is circumscribed in extent (pink arrows). In contrast, for purA the reduction in contributions from high variability loci results in a longer low variability region being deemed significantly conserved (green arrows).

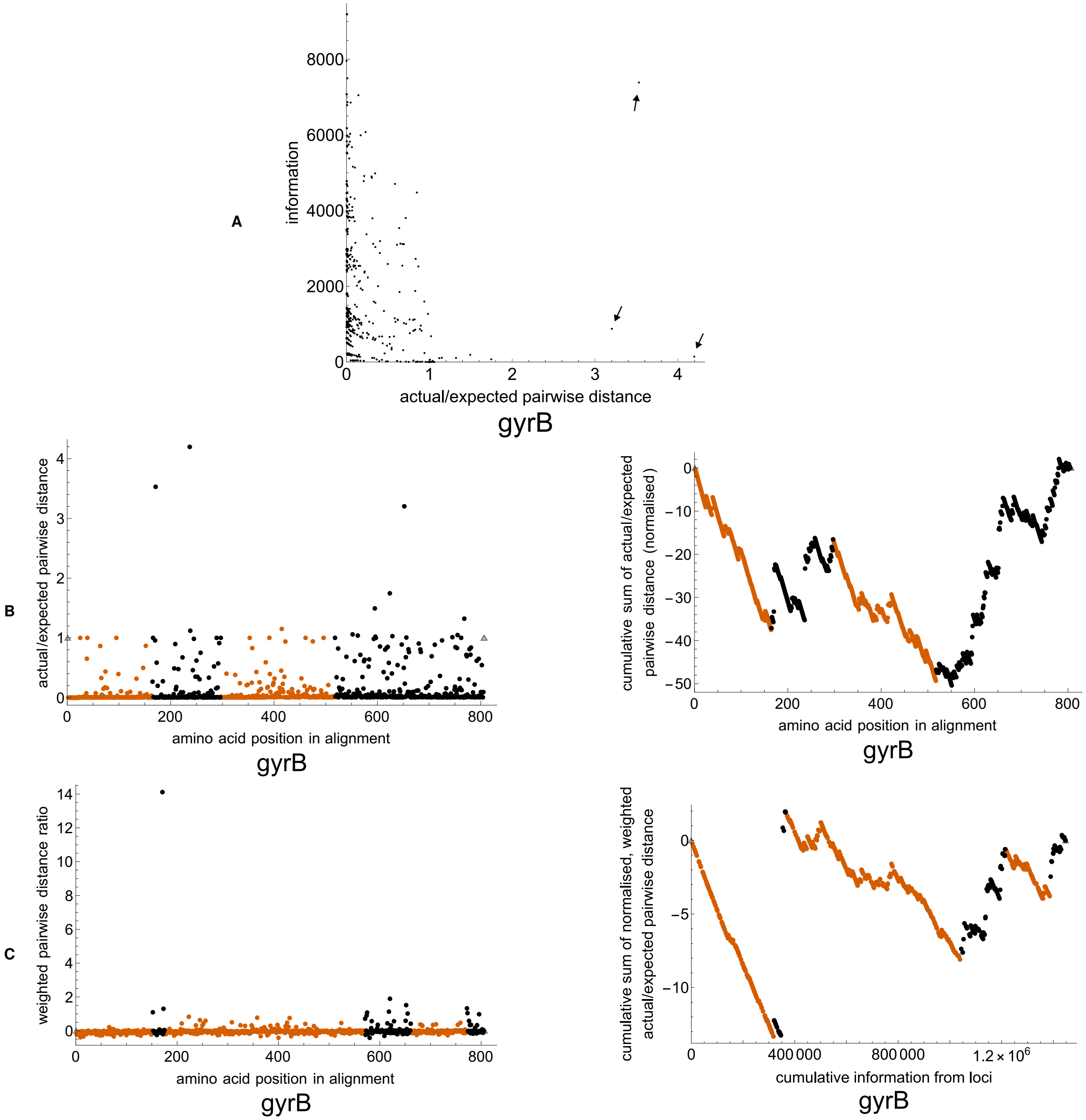

Fig. S3. (**A**) Information content for each codon in the *E. coli* gyrB housekeeping gene, versus measured variability at the codon (see text for explanation). Without weighting by information content, a small number of loci, most with low information, dominate the analysis (arrows). (**B**) Analysis output for *E. coli* gyrB (raw values left, summed values right) with loci equally weighted and raw variability measure, showing output that does distinguish low and high variability regions, but the high variability "ascents" are dominated by a few loci. (**C**) Analysis output for *E. coli* gyrB (raw values left, summed values right) with loci weighted by information content and raw variability measure. Although there is more granularity than with equal weighting, a single high-information locus now dominates the analysis. In both analyses, a consequence of the domination by a small number of high variability points is that a large portion of the gene is determined by the algorithm to be conserved. Weighting reduces the number of loci causing the problem, but because it does not eliminate such loci entirely, the problem persists.

It is possible to combine ranking measured variability with weighting loci for information content (Fig. S6). However, as the ranking of a locus represents an increment dependent on the rank of every locus with lower variability, the weighting of all lower rank loci needs to be taken into account when calculating a weighted rank. The appropriate approach is discussed more fully in the Methods.

C. Step 3: Multiple interfering signals. In our previous work(3), we demonstrated that when looking for contiguous signal and comparing an identified putative signal against background, the presence of an additional signal in the background region unsurprisingly interfered with signal identification, reducing the algorithm's sensitivity. We demonstrated that the effect was worst near where the two signals had similar signal strength. This is appropriate and expected behaviour for an algorithm that aims to find regions of higher conservation in a gene than can be found elsewhere in the gene. However, the aim of the algorithm should be to compare conservation between an identified region and *background* conservation, not the level of conservation in another contiguous, conserved region.

A straightforward adaptation to our algorithm where a region's conservation is deemed non-significant is to check that this is not caused by the presence of another contiguous conserved region. This can be accomplished by repeating the significance analysis in the absence of the next most significant region. We discuss the procedure in detail in the Methods.

We have benchmarked this approach, and results are displayed in Fig. S7. As might be expected, the approach yields an increase in sensitivity for finding signal regions, at the expense of a small decrease in specificity. The trade-off is usually acceptable for two key reasons. First, the initially rejected signal is boosted most when there is a second, long signal – in the absence of such a signal, any boost will come from a small region identified randomly, and so will be much smaller. Second, it is possible to add labels highlighting regions identified only after removal of another region, meaning interpretation can be undertaken with additional caution. In any case, this is a modification to a procedure whose purpose is to identify regions that merit further analysis and/or investigation.

The application of this procedure to the *E. coli* examples shown earlier is displayed in Fig. S8.

D. Lessons from benchmarking. So far, we have considered three possible algorithmic refinements for finding regions of high conservation in genetic data. Given we can choose whether or not to apply each of these refinements, we now have eight possible ways of analysing a given dataset. We therefore consider when each refinement should be applied.

It is important to recognise that from a biological perspective, any combination of these algorithms serves the same purpose: namely, to highlight regions within a genome that merit further biological investigation. Deciding which combination to use is therefore not only a matter of the mathematical properties of an algorithm, but also of how well they serve this biological purpose.

Weighting loci by information content (section A) is the most difficult refinement to benchmark clearly, but appears to improve the signal-to-noise ratio in the data and has a clear mathematical justification. We therefore recommend always including this refinement.

Ranking loci by their variability (section B) appears likely from benchmarking to have a substantial benefit in avoiding false positive results in a minority of cases with non-normal data. There is only a small reduction in identification of true positives. We therefore recommend using the ranking refinement, unless it is clear that the data should approach the (Gaussian) central limit – ideally by formally testing this, but biological context would also be a reasonable way of justifying such a choice. (For example, our earlier work on HIV-1(2) used sequences with high inter-sequence variability, and ranking loci there would be less important than it would be for SARS-CoV-2.)

The procedure for identifying conserved regions that are obscured by other, similarly conserved regions (section C) can always be run, as it is possible to distinguish between regions that are identified using this additional procedure and regions that do not require it, with only a modest increase in computational time. The identification of a region subject to a modestly higher false positive rate can therefore be flagged and taken into account when considering biological significance.

For our application to SARS-CoV and SARS-CoV-2, we therefore analyse data using all the procedural refinements we have described above.

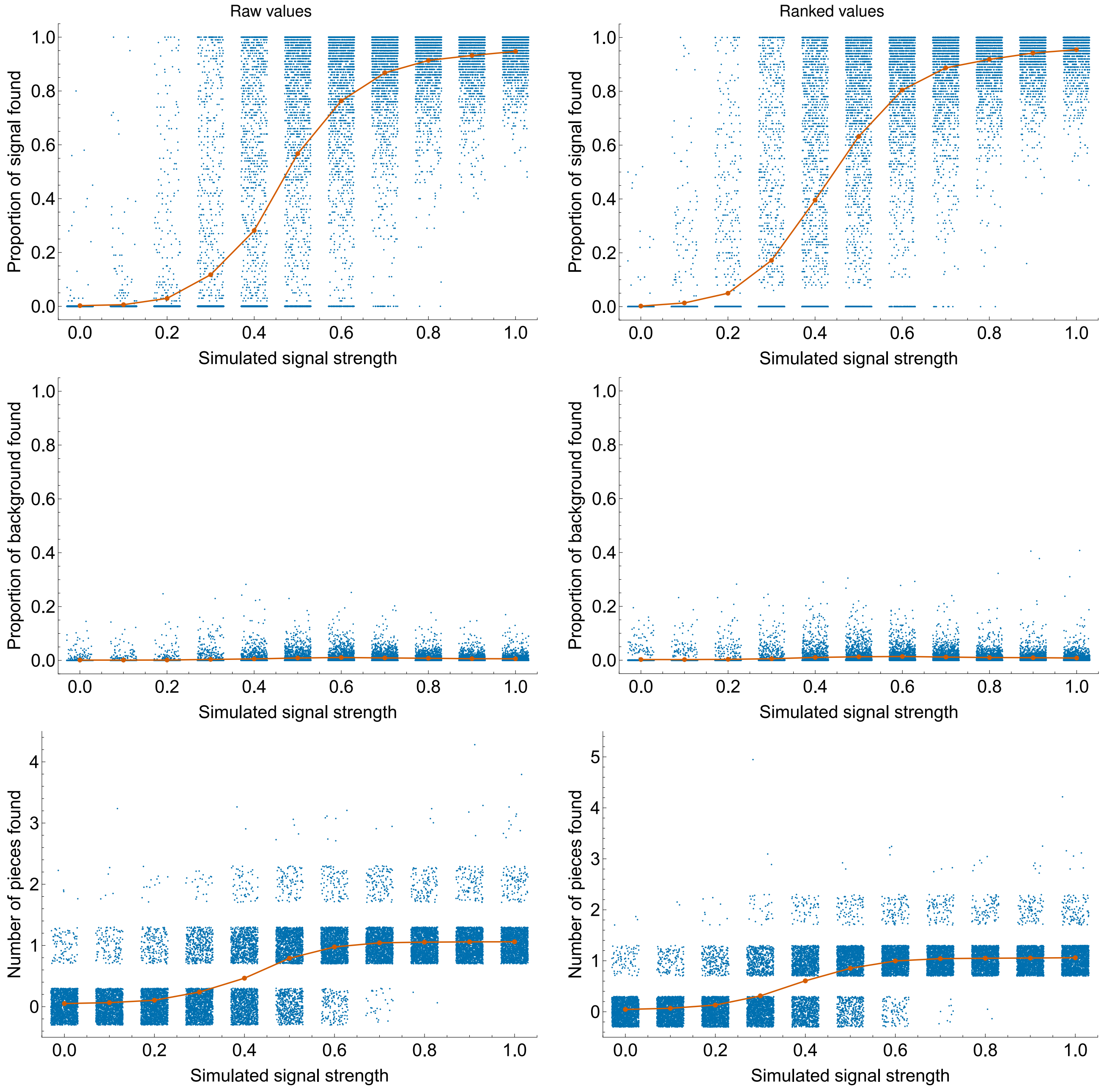

Fig. S4. Effect of analysing Gaussian signal and background using ranking of values. For different signal strengths, 2,000 sequences were simulated with each value in the sequence a standard normal variable, and the middle 100 of 500 positions were offset by the signal strength to yield a signal region. Sequences were generated independently (i.e. not reused for different signal strengths). The algorithm was then applied to attempt to find the signal. The left column shows the effect of running the algorithm on the raw values generated and the right column shows the effect of running the algorithm with the values ranked. The dots show individual data points, dithered horizontally in all plots and vertically as well in the lowest pair of plots. The lines show averages. The upper plots give the proportion of the signal (middle 100 of 500 positions) found by the algorithm. The middle plots give the proportion of the background (other 400 of 500 positions) identified as signal. The bottom plots give the number of separate pieces identified as signal. The results are very similar between the raw and ranked data, with a small increase in proportion of signal found associated with ranking, most probably because in this simulated dataset the smoothing effect of ranking reduces the chances of identifying a smaller piece of signal and missing the remainder.

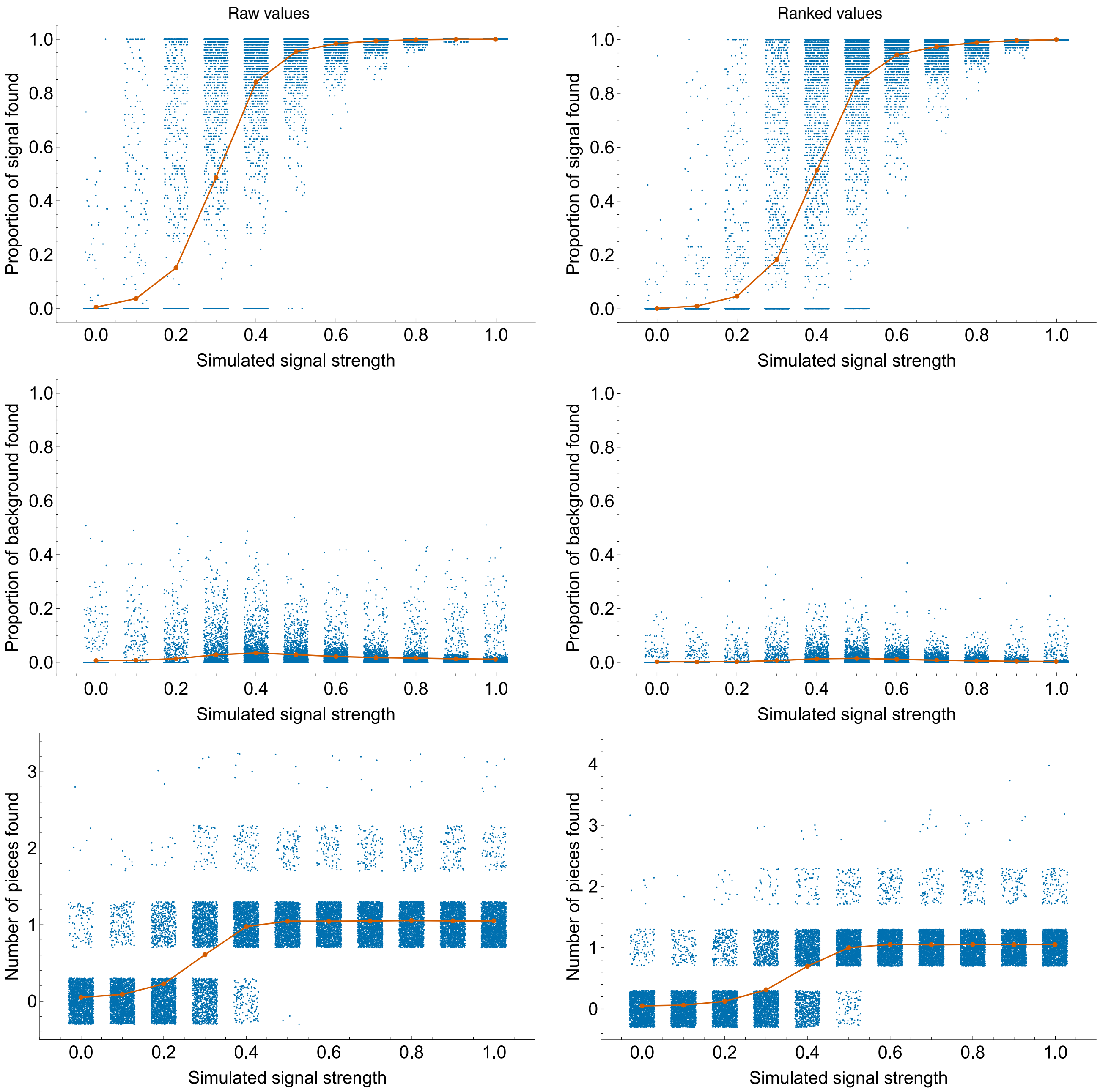

Fig. S5. Effect of analysing exponential signal and background using ranking of values. For different signal strengths, 2,000 sequences were simulated with the middle 100 of 500 positions exponentially distributed with mean $(1 + (\text{simulated signal strength}))$ and the remaining positions exponentially distributed with mean 1. The algorithm was then applied to attempt to find the signal. The left column shows the effect of running the algorithm on the raw values generated and the right column shows the effect of running the algorithm with the values ranked. The dots show individual data points, dithered horizontally in all plots and vertically as well in the lowest pair of plots. The lines show averages. The upper plots give the proportion of the signal (middle 100 of 500 positions) found by the algorithm. The middle plots give the proportion of the background (other 400 of 500 positions) identified as signal. The bottom plots give the number of separate pieces identified as signal. With this skewed distribution, ranking values mitigates a number of instances of miscalling background as signal, at the expense of reducing the sensitivity for finding signal. Note that the algorithm efficiency for finding signal is not directly comparable with the Gaussian signal and background because the standard deviation of signal values is smaller here. We note that although the distribution benchmarked here is skewed, it is still parametric and genetic data with low variability might generate worse algorithm behaviour in misidentifying background as signal.

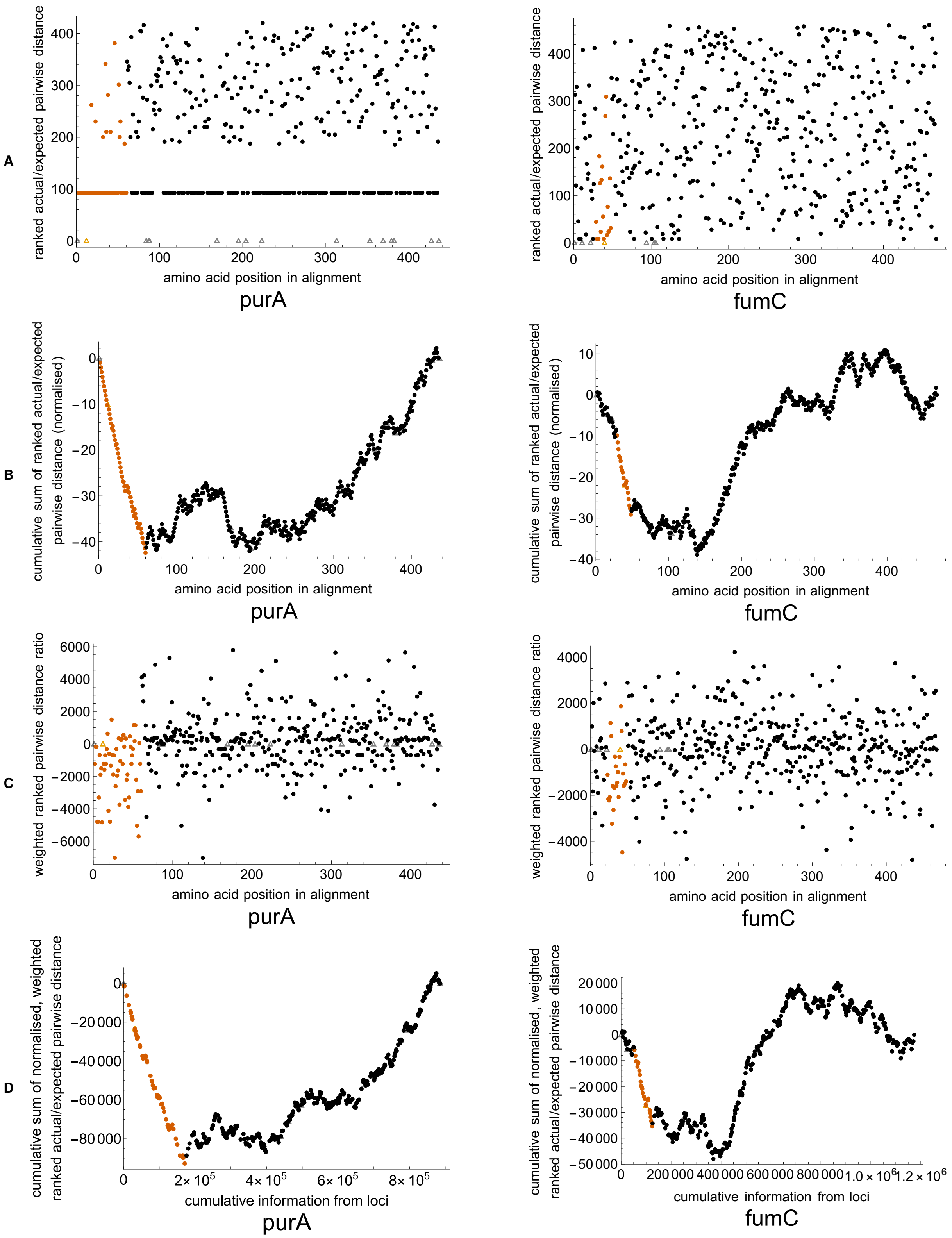

Fig. S6. Effect of ranking information content of loci in *E. coli* housekeeping genes purA (left) and fumC (right). In cumulative sums and where weighting is applied, ranked variability measures are normalised to have mean 0 and standard deviation 1. (**A**) Ranked raw variability measures. (**B**) Cumulative sums of ranked raw variability measures. (**C**) Weighted ranked variability measures (see Methods for details). (**D**) Cumulative sums of weighted ranked variability measures. Orange loci correspond to regions deemed to be significantly conserved by our algorithm. Grey and light orange loci plotted with open triangles yield no information on nucleotide conservation and were not included in analyses(2). Ranking mitigates the effect of outlying, high variability loci. In both cases seen here, ranking alone leads to identification of similar conserved regions to those seen with weighting, and the combined effect is similar, but regions are slightly differently circumscribed.

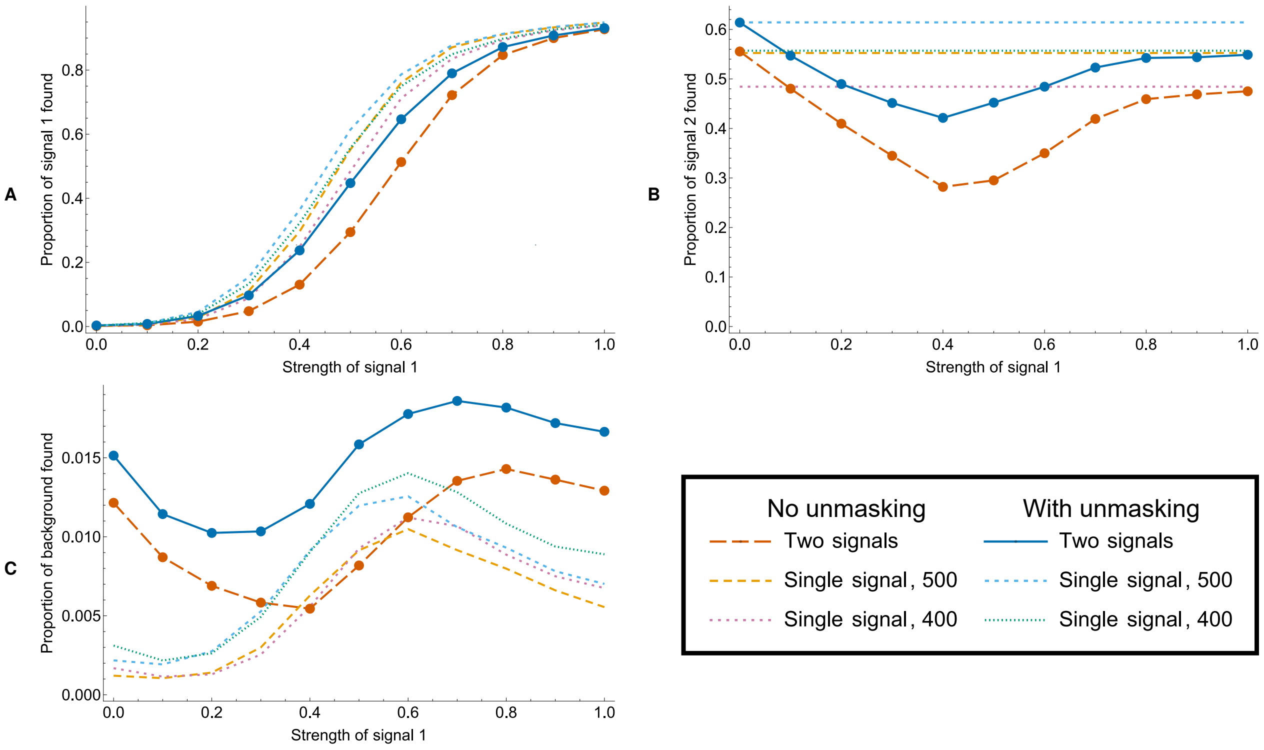

Fig. S7. Effect of implementing a procedure to unmask a signal obscured by the presence of a second signal, by repeating analysis with the next most significant region excluded in the event the first region identified by our algorithm is deemed non-significant. In each case two signals of length 100 are generated in a sequence of length 500. The background points in the sequence are sampled from a standard normal distribution. The signal points are sampled from a normal distribution with mean offset by the plotted "strength" for signal 1, and always offset by 0.5 for signal 2. For each average, 10,000 simulations were run, with the decision on whether to accept/reject a proposed signal region made following 1,000 bootstrap resamples of the simulated data. Results are plotted for the same simulations with and without the signal unmasking procedure used. Also plotted are equivalent results for finding a single signal in a sequence of length 500 and of length 400 (a single signal in a sequence of length 500 is the appropriate comparator when the algorithm searches for the first signal it can find; if this signal is found and excised then the appropriate comparator for finding the second signal is in a sequence of the remaining length of 400). (**A**) Proportion of first signal found as the signal strength varies. (**B**) Proportion of second signal found as the first signal strength varies. (**C**) Proportion of background (non-signal region) found as the first signal strength varies. Note that the average proportion of background found represents at most just over 5 positions in the sequence. Overall, the effectiveness of the algorithm in finding two signals in a sequence differs most from the effectiveness at finding one signal when the two signals are of similar strengths (so that thre is an obscuring effect from the second signal being counted in the background when evaluating the significance of the first signal). The issue can be partially rescued by implementing the unmasking procedure (and this procedure also slightly increases the algorithm's ability to find single signals), at a small cost in increased misidentification of background sequence as signal.

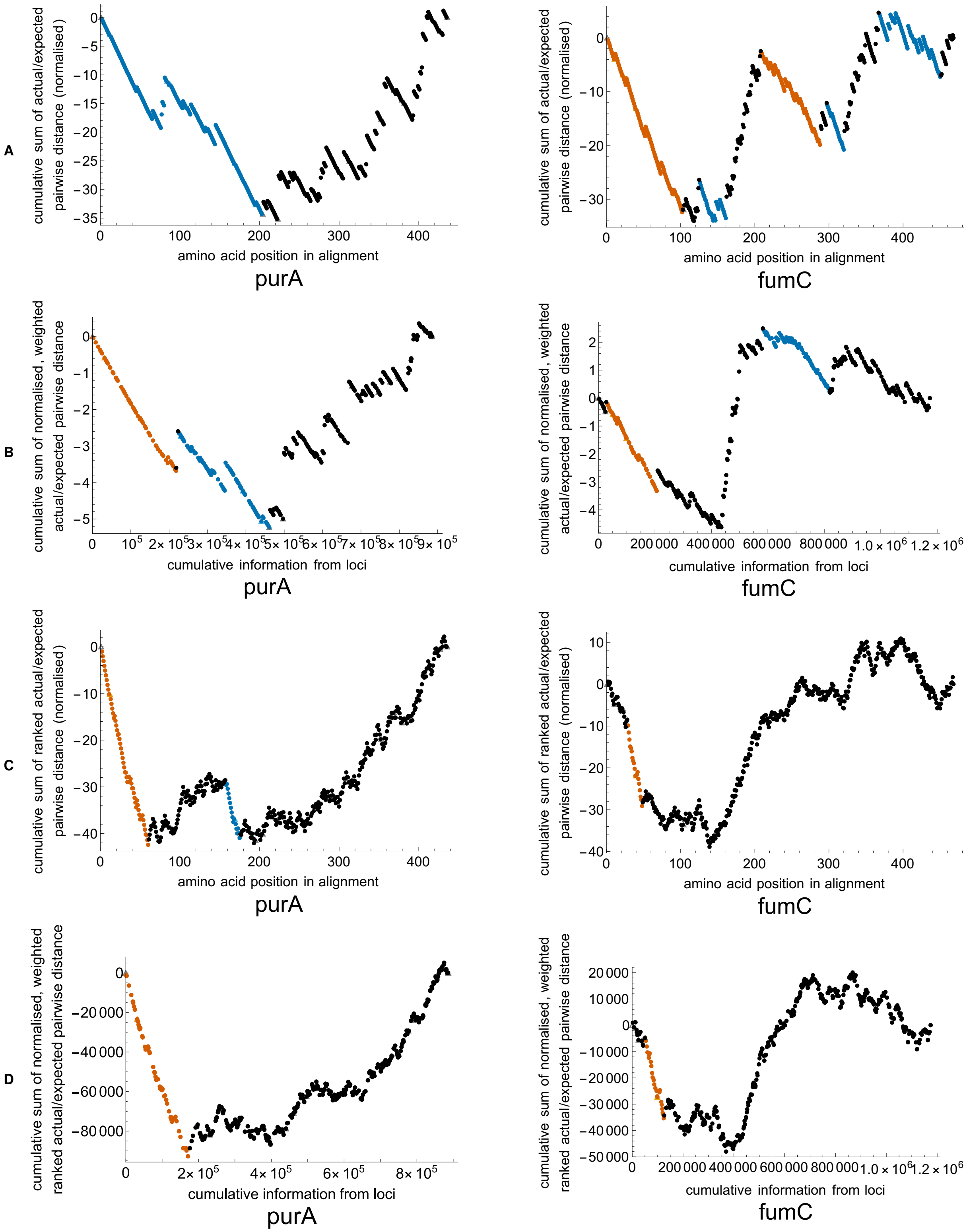

Fig. S8. Effect of including screen for possible interfering signals in *E. coli* housekeeping genes purA (left) and fumC (right). The analyses performed are as in Figs. S2 and S6, save that the analysis for possible interfering signals described in the text is performed. Regions additionally deemed significant after this analysis are highlighted in blue. (**A**) Cumulative sums of raw variability measures. (**B**) Cumulative sums of weighted raw variability measures. (**C**) Cumulative sums of ranked variability measures. (**D**) Cumulative sums of weighted ranked variability measures. Orange loci correspond to regions deemed to be significantly conserved by our algorithm prior to the interfering signals analysis. Grey, light orange and light blue loci plotted with open triangles yield no information on nucleotide conservation and were not included in analyses(2).

2. Notes on conserved regions in *Escherichia coli*

In all of the housekeeping genes we analyse, our algorithm finds regions with relative conservation at the start of the gene. This is a previously described phenomenon (4–11), and serves as a useful positive control for our algorithm. For two genes, we note below additional evidence that corroborates our findings.

Isocitrate dehydrogenase (*icd*) gene There is a region of high diversity at the 3′ end of the gene, visible as a steep ascent in the cumulative sum graphs of Fig. S13. This phenomenon is known to arise from the presence in some strains of *E. coli* of one of two bacteriophages (12). This region of high diversity appropriately results in regions in the middle of the gene being called as relatively conserved by our algorithm in some cases – although the phenomenon being observed could be regarded less as conservation in one region, and more as increased diversity in another.

Malate dehydrogenase (*mdh*) gene Our finding of a conserved region towards the 5′ end of the gene is consistent with the locations of polymorphic loci found in earlier works (13, 14).

3. Additional notes on conserved regions in SARS-CoV-2

Below, we note two regions called as conserved by our algorithm, together with possible biological explanations. In each case, these are not described in the main text because either the evidence for the advanced possible explanation is weak or there is evidence that casts doubt upon the explanation. In each case, we discuss the evidence. We include these discussions as methodological examples of the types of conserved regions that might be found and investigations that should be undertaken to corroborate different putative explanations. We also wish to describe the evidence we have found relating to these regions, in case it later corroborates experimental evidence that should arise.

Candidate negative-sense open reading frame in SARS-CoV-2 nsp7 region Our algorithm identifies two mutually interfering signal regions within the nsp7 region of SARS-CoV-2, each of which is only identified as significant in the absence of the other. The first region identified (NC_045512.2 nucleotide reference 11843–11914) begins with AUG in the +1 negative-sense frame, and ends UAG in that frame. There is not a strong Kozak sequence (UAGAUG**AUG**A) in the reference sequence and there is a UGA in the fifth codon position. If translation were initiated in this frame, then a termination codon close to the initiation complex may simply be read through (with the UGA translated as cysteine or tryptophan), especially in the presence of eIF3 (15); however for the reference sequence there are few other markers of high frequency translational readthrough of termination codons (16). We were therefore motivated to consider for the entire dataset (i) how conserved the putative initiation and termination sites are, (ii) the frequency of Kozak nucleotides near the putative initiation codon, and (iii) frequency of a termination codon in the putative +5 codon position and markers of translational readthrough. Fig S9 shows output of analysing the region for conserved nucleotides in the key positions: there is a high level of conservation.

The region of interest is a long distance from the 5′ terminus of negative-sense RNAs, raising the possibility that if translated, translation initiation may occur via an internal ribosome entry site (IRES) rather than in a cap-dependent fashion.

We undertook searches for known similar peptides with the translated sequence as input (searching with each of cysteine and tryptophan in position 5 of the sequence) using BLASTP 2.13.0+ (17) on the non-redundant proteins database. There were no strong hits: the strongest hit in both cases was a region within a *Pseudomonas* protein (accession WP_019581496.1) with E scores of 0.49 and 1.2 for the tryptophan- and cysteine-containing versions respectively. We also undertook searches for known similar peptides using InterProScan 5 with InterPro 89.0 (18, 19): there were no hits. We note (see the Introduction in the main text) that whilst the absence of known analogues may decrease our confidence that this region represents a translated ORF, the absence of analogues if it were translated would make the resultant protein a more specific drug target.

Overall, the evidence for this region representing a biologically significant ORF is weak: the region of interest is not identified except after removal of a potentially interfering region, it is unclear how translation would be initiated on the negative-sense strand, there is an early termination codon without strong markers of translational readthrough, and the putative product does not have any clearly analogous proteins.

Candidate additional frameshift motif spanning SARS-CoV-2 nsp9 and nsp10 regions Our algorithm identifies a small conserved region within the nsp9 region of SARS-CoV-2. Inspection of the region and surrounding nucleotides demonstrates an A_UUA_AAC motif just 5′ to the conserved nucleotides, NC_045512.2 nucleotide location 12964–12970, as well as several UGGUA motifs within and 3′ to the region (NC_045512.2 nucleotide references 12987–12991, 12994–12998 and 13027–13031) that could pair with the UGCCA motif at NC_045512.2 nucleotide location 13048–13052 to form a pseudoknot (Fig. S10). This is reminiscent of known coronavirus frameshift motifs with a slippery site followed by a pseudoknot (20–22), and manual inspection confirms the possibility of a two-loop motif with pseudoknot. The failure of our algorithm to idenfify the entire region can be partially explained by its spanning nsp9 and nsp10; however, the region that is identified is only called conserved by our algorithm after removal of a potentially interfering signal that is not then itself identified as conserved. This raises the possibility that the identified region is in fact a false positive.

The putative slippery site is similar to, but does not match, known coronavirus frameshift motifs (21). If a −1 frameshift occurred at the putative slippery site, the ribosome would find two UAGs in-frame shortly after frameshifting (NC_045512.2 nucleotide references 12979–12981 and 12997–12999). The putative frameshift would therefore either lead to a premature termination and truncated protein, or there would need to be readthrough of the termination codons (which would likely lead to termination at a UAA at the 5′ end of the nsp10 region, NC_045512.2 nucleotide reference 13030–13032). Premature

termination would not in itself exclude the possibility of a frameshift: indeed, the absence of ribosomal frameshifting at the canonical site, leading to termination and generation of the 1a polyprotein, may be viewed as a premature termination that serves to modulate relative abundances of protein cleavage products. Overall, however, the weak algorithmic conservation call, coupled with the weak slippery site, coupled with the premature termination codons in the −1 frame, mean that further corroboration would be required before there could be considered adequate evidence of a frameshift in this region.

4. Note on additional tables and figures relating to *E. coli* data

Table S1 summarizes locations of conserved regions found by our algorithm in the analysed *E. coli* housekeeping genes, using weighted and ranked codon variability values (see main text for the justification for using this analysis procedure). Fig. S11 contain plots relating per-locus variability measures versus per-locus information, for the analysed housekeeping genes for which plots are not found in the main text. Figs. S12 to S18 contain plots showing variability values and conserved regions applying the different options for analysis procedures.

5. Note on additional tables and figures relating to SARS-CoV-2 and to SARS-CoV data

Tables summarizing locations of conserved regions found by our algorithm in SARS-CoV-2 may be found in the main text. The corresponding tables for SARS-CoV are Tables S2 and S3. Table S12 lists GenBank accession numbers of the sequences used for the SARS-CoV analysis. Figs. S19 and S20 contain plots relating per-locus variability measures versus per-locus information, for the analysed genes and 1ab regions of SARS-CoV-2. The corresponding figures for SARS-CoV are Figs. S61 and S62. Figs. S21 to S28 contain plots showing weighted ranked variability values and conserved regions in SARS-CoV-2 genes and 1ab regions. The corresponding figures for SARS-CoV are Figs. S63 to S71.

6. Note on RNAalifold figures

Figs. S29–S60 and Figs. S72–S96 give predicted folds using RNAalifold of the regions determined to be conserved by our algorithm in SARS-CoV-2 and SARS-CoV, respectively. As noted in the Methods, folds are only attempted for regions ≤ 5500 nucleotides in length – this is motivated primarily by memory constraints when running RNAalifold, but a secondary motivation for avoiding folds of longer regions is that the chances our algorithm has identified the dominant length scale for secondary structure formation would seem lower when there are multiple possible smaller length scales. Figures are not included for SARS-CoV-2 E regions of interest, 7b regions, and the nsp6 region of interest, because RNAalifold does not predict any base pairings in these regions. The fold of the forward sense RNA in the region of nsp16 thought to contain a packaging signal may be found in the main text. Whilst the SARS-CoV folds appear to reflect better the folding potential of the underlying alignments than the SARS-CoV-2 folds (more deep red pairings, indicating all sequences in alignment can make the displayed pairing), it must be remembered that there are far fewer SARS-CoV than SARS-CoV-2 sequences in the respective datasets, and the SARS-CoV sequences represent less evolutionary time than the SARS-CoV-2 sequences used.

References

1. Wirth T, et al. (2006) Sex and virulence in *Escherichia coli*: an evolutionary perspective. *Molecular Microbiology* 60(5):1136–1151.
2. Skittrall JP, Ingemarsdotter CK, Gog JR, Lever AML (2019) A scale-free analysis of the HIV-1 genome demonstrates multiple conserved regions of structural and functional importance. *PLoS Computational Biology* 15(9):e1007345.
3. Gog JR, Lever AML, Skittrall JP (2018) A new method for detecting signal regions in ordered sequences of real numbers, and application to viral genomic data. *PLoS ONE* 13(4):e0195763.
4. Eyre-Walker A, Bulmer M (1993) Reduced synonymous substitution rate at the start of enterobacterial genes. *Nucleic Acids Research* 21(19):4599–4603.
5. Kudla G, Murray AW, Tollervey D, Plotkin JB (2009) Coding-sequence determinants of gene expression in *Escherichia coli*. *Science* 324:255–258.
6. Tuller T, et al. (2010) An evolutionarily conserved mechanism for controlling the efficiency of protein translation. *Cell* 141:344–354.
7. Lenz G, Doron-Faigenboim A, Ron EZ, Tuller T, Gophna U (2011) Sequence features of *E. coli* mRNAs affect their degradation. *PLoS ONE* 6(12):e28544.
8. Bentele K, Saffert P, Rauscher R, Ignatova Z, Blüthgen N (2013) Efficient translation initiation dictates codon usage at gene start. *Molecular Systems Biology* 9:675.
9. Hockenberry AJ, Sirer MI, Nunes Amaral LA, Jewett MC (2014) Quantifying position-dependent codon usage bias. *Molecular Biology and Evolution* 31(7):1880–1893.
10. Bhattacharyya S, et al. (2018) Accessibility of the Shine-Dalgarno sequence dictates N-terminal codon bias in *E. coli*. *Molecular Cell* 70:894–905.
11. Pedersen S, et al. (2019) Fast translation within the first 45 codons decreases mRNA stability and increases premature transcription termination in *E. coli*. *Journal of Molecular Biology* 431:1088–1097.
12. Wang FS, Whittam TS, Selander RK (1997) Evolutionary genetics of the isocitrate dehydrogenase gene (*icd*) in *Escherichia coli* and *Salmonella enterica*. *Journal of Bacteriology* 179(21):6551–6559.

13. Boyd EF, Nelson K, Wang FS, Whittam TS, Selander RK (1994) Molecular genetic basis of allelic polymorphism in malate dehydrogenase (*mdh*) in natural populations of *Escherichia coli* and *Salmonella enterica*. *Proceedings of the National Academy of Sciences of the USA* 91:1280–1284.
14. Pupo GM, Karaolis DKR, Lan R, Reeves PR (1997) Evolutionary relationships among pathogenic and nonpathogenic *Escherichia coli* strains inferred from multilocus enzyme electrophoresis and *mdh* sequence studies. *Infection and Immunity* 65(7):2685–2692.
15. Beznosková P, Wagner S, Jansen ME, von der Haar T, Valášek LS (2015) Translation initiation factor eIF3 promoted programmed stop codon readthrough. *Nucleic Acids Research* 43(10):5099–5111.
16. Dabrowski M, Bukowy-Bieryllo Z, Zietkiewicz E (2015) Translational readthrough potential of natural termination codons in eucaryotes – the impact of RNA sequence. *RNA Biology* 12(9):950–958.
17. Altschul SF, et al. (1997) Gapped BLAST and PSI-BLAST: a new generation of protein database search programs. *Nucleic Acids Research* 25(17):3389–3402.
18. Jones P, et al. (2014) InterProScan 5: genome-scale protein function classification. *Bioinformatics* 30(9):1236–1240.
19. Blum M, et al. (2020) The InterPro protein families and domains database: 20 years on. *Nucleic Acids Research* 49(D1):D344–D354.
20. Dos Ramos F, Carrasco M, Doyle T, Brierley I (2004) Programmed −1 ribosomal frameshifting in the SARS coronavirus. *Biochemical Society Transactions* 32(6):1081–1083.
21. Atkins JF, Loughran G, Bhatt PR, Firth AE, Baranov PV (2016) Ribosomal frameshifting and transcriptional slippage: from genetic steganography and cryptography to adventitious use. *Nucleic Acids Research* 44(15):7007–7008.
22. Bhatt PR, et al. (2021) Structural basis of ribosomal frameshifting during translation of the SARS-CoV-2 RNA genome. *Science* 372(6548):1306–1313.
23. Hsieh PK, et al. (2005) Assembly of severe acute respiratory syndrome coronavirus RNA packaging signal into virus-like particles is nucleocapsid dependent. *Journal of Virology* 79:13848–13855.
24. Woo J, Lee EY, Lee M, Kim T, Cho YE (2019) An *in vivo* cell-based assay for investigating the specific interaction between the SARS-CoV N-protein and its viral RNA packaging sequence. *Biochemical and Biophysical Research Communications* 520:499–506.
25. Crooks GE, Hon G, Chandonia JM, Brenner SE (2004) WebLogo: a sequence logo generator. *Genome Research* 14(6):1188–1190.
26. Reuter JS, Mathews DH (2010) RNAstructure: software for RNA secondary structure prediction and analysis. *BMC Bioinformatics* 11:129.

Table S1. Summary of regions of significant conservation found in *E. coli* genes, using weighted and ranked codon variability values. *Denotes a region only found by excluding a potentially interfering signal. Z- and p-values in parentheses denote values prior to removal of the next most significant signal. If parenthetical values are absent, then such a signal was removed in an earlier step only. †Denotes a gene expressed from the reverse DNA strand.

Gene	Order found	NC_000913 nt location	Z	p
adk	1	497178–497225	3.02	0.0001
icd	1	1195126–1195377	3.05	0.0058
	2*	1195534–1195554	2.42 (2.38)	0.0425 (0.0803)
fumC	1	1686522–1686445†	2.25	0.0151
recA	1	2823766–2823692†	3.23	0.0321
mdh	1	3384247–3384080†	3.56	0.0005
gyrB	2	3879735–3879082†	3.03	<0.0001
	1	3880116–3879739†	6.27	<0.0001
purA	1	4404690–4404866	4.96	<0.0001

Table S2. Summary of regions of significant conservation found in SARS-CoV genes. *Denotes a region only found by excluding a potentially interfering signal. Z- and p-values in parentheses denote values prior to removal of the next most significant signal. If parenthetical values are absent, then such a signal was removed in an earlier step only. sgRNA=subgenomic RNA, TRS=transcription-regulatory sequence (-B=body).

Gene	Order found	AY274119.3 nt location	Z	p	Comment
1ab	1	14211–16889	3.69	0.0227	Reason for conservation unclear.
S	2	21732–22421	2.86	0.0001	Reason for conservation unclear.
	1	24099–24869	3.12	0.0177	Region contains analogues of the 1st and 4th regions identified in SARS-CoV-2 S. Reason for conservation unclear.
E	1	26162–26200	2.64	<0.0001	5′ end of region has identical sequence to region in SARS-CoV-2 that is a possible TRS-B for forming alternate M sgRNA.
7b	1*	27650–27760	1.51 (1.42)	0.0001 (0.0792)	Result dominated by two high variability loci and appears artefactual; although region contains both regions identified in SARS-CoV-2, most of 7b is called conserved here by the algorithm.
8b	1*	27867–28019	1.03 (0.84)	0.0373 (0.5361)	Result dominated by two high variability loci and appears artefactual.
	2*	28023–28100	1.06 (0.97)	0.0106 (0.0852)	Result dominated by two high variability loci and appears artefactual.

Table S3. Summary of regions of significant conservation found in individual proteins of SARS-CoV 1a/1ab. *Denotes a region only found by excluding a potentially interfering signal. Z- and p-values in parentheses denote values prior to removal of the next most significant signal. If parenthetical values are absent, then such a signal was removed in an earlier step only.

Region	Order found	AY274119.3 nt location	Z	p	Comment
NSP2	1*	2017–2547	2.65 (2.47)	0.0343 (0.0941)	Result dominated by high variability loci, even in the weighted ranked setting. Whilst may contain real conservation, there is a risk the result is artefactual.
NSP3	1	6613–6987	2.48	0.0055	Reason for conservation unclear.
NSP6	1*	10978–11412	2.28 (1.99)	0.015 (0.1297)	Reason for conservation unclear.
NSP7	1	11791–11970	1.45	0.0422	Reason for conservation unclear.
	2*	11977–12021	1.47 (1.34)	0.0048 (0.0783)	Reason for conservation unclear.
NSP10	2*	12985–13095	1.74 (1.57)	0.0276 0.1137	Reason for conservation unclear.
	1*	13153–13344	1.72 (1.45)	0.0408 0.3846	Reason for conservation unclear.
NSP12	2*	13372–14037	2.30 (2.04)	0.0479 0.2772	Reason for conservation unclear.
	1*	14208–15852	2.34 (2.02)	0.0421 0.9977	Reason for conservation unclear.
NSP15	1*	19920–20195	2.38 (2.15)	0.0245 0.1014	Putative packaging signal(23, 24); see main text.

Table S4. GenBank accession numbers of sequences used for *E. coli* adk analysis.

AAKB02000001	AGTD01000001	AP017610	AP017617	AP017620	AP018488
AP018784	AP018796	AP018802	AP018808	AP019189	AP019675
AP019856	AP021890	AP021891	AP021892	AP021893	AP021894
AP021895	AP021896	AP021914	AP021933	AP021935	AP021944
AP021946	AP021963	AP021998	AP022003	AP022030	AP022032
AP022036	AP022044	AP022049	AP022064	AP022069	AP022087
AP022095	AP022098	AP022105	AP022107	AP022120	AP022159
AP022161	AP022171	AP022173	AP022177	AP022207	AP022215
AP022221	AP022222	AP022245	AP022261	AP022287	AP022295
AP022298	AP022326	AP022351	AP022362	AP022409	AP022478
AP022482	AP022525	AP022533	AP022536	AP022540	AP022549
AP022650	AP022811	AP022815	AP023190	AP023197	AP023219
AP023224	AP023226	AP023230	AP023235	AP023237	AP023286
AP023427	AP024112	AP024114	AP024123	AP024126	AP024130
AP024131	AP024205	AP024471	AP024473	AP024475	AP024478
AP024518	AP024521	AP024582	AP024687	AP024694	AP024921
AP024924	AP025205	AP025209	AP025214	AP025220	APIN01000002
AXLH01000003	BA000007	BNEX01000001	BNFC01000001	BNFH01000001	BNFI01000001
BNFJ01000001	BNFK01000001	BNFN01000001	BNFQ01000001	BNFS01000001	BNIP01000001
BNIQ01000001	BNIS01000001	BNIT01000001	BNIU01000001	BNIV01000001	BNIW01000001
BNIZ01000001	BNJA01000001	BNJB01000001	BNSX01000001	CABEEY010000003	CABFNM010000001
CABFNN010000001	CABMNJ010000001	CABWHO010000003	CABWIX010000001	CABWJC010000001	CABWJF010000003
CABWJL010000001	CABWJM010000001	CABWJO010000005	CACRWJ010000001	CACRWK010000001	CACRWL010000001
CACRWM010000001	CACRWO010000001	CACRWR010000001	CACRWV010000001	CADDWP010000001	CADDWS010000001
CADDWT010000001	CADDWW010000001	CADDWZ010000001	CADDXF010000001	CADDXG010000001	CADDXH010000001
CADDXI010000001	CADDXJ010000001	CADDXK010000001	CADDXL010000002	CADDXM010000001	CADDXN010000001
CADDXO010000001	CADDXP010000001	CADDXQ010000002	CADDXU010000001	CADDXV010000001	CADEAI010000002
CAJGEG010000001	CAJGEH010000001	CAJGEI010000001	CAJGWN010000001	CAJMUK010000001	CAJMWD010000001
CAJNJR010000001	CAJNTA010000001	CAJNTK010000001	CAJNTR010000001	CAJNTY010000001	CAJNUB010000001
CAJNVV010000001	CAJOCA010000001	CAJVAH010000001	CM000662	CM007915	CP001509
CP002967	CP002970	CP003034	CP006027	CP006698	CP006830
CP006834	CP007133	CP007265	CP007275	CP007392	CP007393
CP007394	CP007491	CP007594	CP008697	CP008805	CP009072
CP009104	CP009106	CP009166	CP009273	CP009578	CP009644
CP009685	CP009789	CP009859	CP010116	CP010117	CP010119
CP010121	CP010125	CP010129	CP010132	CP010133	CP010134
CP010137	CP010140	CP010148	CP010150	CP010151	CP010152
CP010157	CP010160	CP010169	CP010170	CP010177	CP010178
CP010183	CP010186	CP010200	CP010206	CP010221	CP010228
CP010229	CP010230	CP010236	CP010238	CP010240	CP010242
CP010315	CP010344	CP010371	CP010439	CP010440	CP010441
CP010442	CP010816	CP011018	CP011124	CP011134	CP011320
CP011321	CP011322	CP011323	CP011324	CP011342	CP011343
CP011416	CP011495	CP011938	CP012126	CP012376	CP012379
CP012631	CP012635	CP012693	CP012781	CP012870	CP013025
CP013029	CP013483	CP013658	CP013662	CP013831	CP013835
CP013952	CP014092	CP014111	CP014269	CP014270	CP014348
CP014583	CP014667	CP015023	CP015074	CP015085	CP015159
CP015229	CP015831	CP015834	CP015842	CP015843	CP015853
CP015855	CP016182	CP016358	CP016404	CP016497	CP016546
CP016625	CP016755	CP017061	CP017100	CP017220	CP017251
CP017438	CP017440	CP017442	CP017444	CP017631	CP017669
CP017844	CP017979	CP017980	CP018103	CP018206	CP018241
CP018243	CP018245	CP018247	CP018252	CP018323	CP018770
CP018801	CP018840	CP018948	CP018953	CP018965	CP018970
CP018976	CP018983	CP018991	CP018995	CP019000	CP019005
CP019008	CP019012	CP019029	CP019051	CP019243	CP019245
CP019250	CP019256	CP019259	CP019267	CP019273	CP019280
CP019355	CP019361	CP019392	CP019455	CP019558	CP019560
CP019778	CP019903	CP019953	CP019961	CP020025	CP020055
CP020058	CP020092	CP020106	CP020107	CP020116	CP020368
CP020495	CP020509	CP020514	CP020516	CP020545	CP020933
CP021175	CP021179	CP021193	CP021207	CP021288	CP021335
CP021339	CP021454	CP021532	CP021535	CP021689	CP021691
CP021722	CP021726	CP021732	CP021840	CP021879	CP021935
CP022050	CP022086	CP022154	CP022393	CP022407	CP022466
CP022664	CP022689	CP022730	CP022912	CP022959	CP023061
CP023165	CP023200	CP023258	CP023353	CP023357	CP023359
CP023364	CP023366	CP023386	CP023535	CP023541	CP023673
CP023749	CP023826	CP023870	CP023960	CP024056	CP024131
CP024134	CP024147	CP024232	CP024240	CP024245	CP024256
CP024257	CP024263	CP024269	CP024275	CP024289	CP024299

Continued on next page

CP024479	CP024618	CP024650	CP024659	CP024661	CP024667
CP024717	CP024720	CP024801	CP024821	CP024826	CP024830
CP024851	CP024855	CP024862	CP024886	CP024889	CP024978
CP024992	CP024997	CP025036	CP025251	CP025318	CP025520
CP025573	CP025716	CP025739	CP025753	CP025842	CP025847
CP025849	CP025851	CP025856	CP025859	CP025862	CP025865
CP025881	CP025892	CP025903	CP025910	CP025916	CP025920
CP025950	CP026199	CP026202	CP026491	CP026580	CP026755
CP026853	CP026929	CP026932	CP026935	CP026939	CP027105
CP027118	CP027126	CP027205	CP027221	CP027255	CP027307
CP027310	CP027312	CP027319	CP027323	CP027325	CP027328
CP027331	CP027335	CP027338	CP027340	CP027342	CP027347
CP027351	CP027352	CP027355	CP027361	CP027362	CP027363
CP027366	CP027368	CP027373	CP027380	CP027387	CP027388
CP027390	CP027394	CP027430	CP027437	CP027440	CP027442
CP027445	CP027447	CP027449	CP027452	CP027454	CP027457
CP027459	CP027461	CP027520	CP027534	CP027544	CP027546
CP027550	CP027552	CP027555	CP027573	CP027577	CP027579
CP027584	CP027586	CP027587	CP027591	CP027593	CP027597
CP027599	CP027701	CP027766	CP027851	CP028110	CP028112
CP028116	CP028117	CP028122	CP028126	CP028166	CP028192
CP028304	CP028305	CP028308	CP028309	CP028310	CP028381
CP028429	CP028578	CP028590	CP028600	CP028603	CP028609
CP028623	CP028641	CP028647	CP028650	CP028656	CP028674
CP028677	CP028680	CP028685	CP028693	CP028695	CP028698
CP028700	CP028702	CP028703	CP028714	CP029057	CP029108
CP029111	CP029115	CP029164	CP029180	CP029212	CP029239
CP029240	CP029369	CP029371	CP029420	CP029574	CP029576
CP029579	CP029630	CP029692	CP029741	CP029747	CP029973
CP029981	CP030240	CP030331	CP030767	CP030919	CP030939
CP031105	CP031134	CP031157	CP031215	CP031231	CP031293
CP031321	CP031341	CP031345	CP031349	CP031353	CP031546
CP031609	CP031653	CP031706	CP031833	CP031892	CP031902
CP031906	CP031908	CP031910	CP031912	CP031919	CP031922
CP032066	CP032073	CP032078	CP032085	CP032145	CP032201
CP032204	CP032237	CP032261	CP032426	CP032789	CP032793
CP032801	CP032803	CP032805	CP032808	CP032811	CP032879
CP032892	CP032986	CP033096	CP033250	CP033378	CP033762
CP033850	CP034384	CP034404	CP034589	CP034595	CP034658
CP034734	CP034745	CP034792	CP034794	CP034799	CP034801
CP034803	CP034808	CP034958	CP034966	CP035123	CP035317
CP035318	CP035320	CP035321	CP035322	CP035325	CP035330
CP035331	CP035339	CP035350	CP035354	CP035360	CP035377
CP035476	CP035477	CP035498	CP035516	CP035545	CP035720
CP035767	CP035812	CP035817	CP035819	CP035822	CP035832
CP035836	CP035841	CP035846	CP035850	CP035855	CP035860
CP035865	CP035870	CP035874	CP035879	CP035882	CP035884
CP035944	CP036202	CP036245	CP037857	CP037903	CP037910
CP037941	CP037943	CP038138	CP038180	CP038287	CP038300
CP038302	CP038305	CP038313	CP038316	CP038324	CP038328
CP038333	CP038336	CP038339	CP038342	CP038346	CP038353
CP038357	CP038360	CP038366	CP038372	CP038376	CP038380
CP038383	CP038389	CP038394	CP038398	CP038402	CP038405
CP038412	CP038416	CP038419	CP038421	CP038425	CP038428
CP038494	CP038496	CP038505	CP038791	CP038859	CP039403
CP039404	CP039837	CP040263	CP040269	CP040311	CP040313
CP040314	CP040381	CP040390	CP040397	CP040456	CP040572
CP040643	CP040664	CP040886	CP040927	CP041002	CP041031
CP041284	CP041300	CP041304	CP041337	CP041359	CP041416
CP041422	CP041429	CP041431	CP041433	CP041437	CP041442
CP041448	CP041520	CP041526	CP041532	CP041535	CP041538
CP041541	CP041544	CP041546	CP041550	CP041556	CP041559
CP041563	CP041572	CP041578	CP041581	CP041627	CP041628
CP041678	CP041749	CP041919	CP042246	CP042250	CP042298
CP042336	CP042470	CP042585	CP042588	CP042845	CP042846
CP042865	CP042867	CP042878	CP042892	CP042893	CP043181
CP043185	CP043197	CP043199	CP043205	CP043213	CP043217
CP043227	CP043406	CP043414	CP043539	CP043946	CP043950
CP044143	CP044145	CP044148	CP044311	CP044312	CP044313
CP044314	CP044315	CP044403	CP044410	CP045206	CP045209
CP045213	CP045277	CP045457	CP045712	CP045741	CP045827
CP045863	CP045975	CP045977	CP046003	CP046009	CP046259
CP046396	CP046416	CP046527	CP046539	CP046716	CP047002

Continued on next page

CP047010	CP047074	CP047076	CP047077	CP047078	CP047079
CP047127	CP047231	CP047277	CP047378	CP047405	CP047455
CP047461	CP047594	CP047600	CP047665	CP047710	CP048010
CP048107	CP048290	CP048304	CP048310	CP048322	CP048326
CP048330	CP048337	CP048344	CP048359	CP048367	CP048371
CP048376	CP048439	CP048603	CP048604	CP048605	CP048606
CP048647	CP048821	CP048853	CP048855	CP048857	CP048860
CP048863	CP048865	CP048867	CP048870	CP048873	CP048915
CP048920	CP048934	CP048935	CP049050	CP049077	CP049081
CP049085	CP049086	CP049101	CP049118	CP049196	CP049197
CP049198	CP049201	CP049204	CP049299	CP049343	CP049348
CP049353	CP049839	CP049845	CP049852	CP049936	CP049943
CP049967	CP049979	CP050031	CP050036	CP050040	CP050044
CP050047	CP050173	CP050193	CP050195	CP050196	CP050197
CP050198	CP050200	CP050201	CP050202	CP050203	CP050204
CP050205	CP050206	CP050208	CP050210	CP050212	CP050213
CP050214	CP050215	CP050216	CP050219	CP050220	CP050289
CP050382	CP050498	CP050646	CP050862	CP050865	CP051001
CP051158	CP051219	CP051222	CP051609	CP051615	CP051631
CP051688	CP051692	CP051694	CP051698	CP051700	CP051706
CP051711	CP051714	CP051716	CP051719	CP051725	CP051727
CP051733	CP051735	CP051738	CP051744	CP051749	CP051751
CP051753	CP051765	CP051776	CP051778	CP051782	CP051786
CP051790	CP051792	CP051796	CP051798	CP051800	CP051802
CP051804	CP051806	CP051808	CP051811	CP051814	CP051816
CP051819	CP051821	CP051824	CP051827	CP051830	CP051833
CP051837	CP051849	CP052057	CP052877	CP053079	CP053080
CP053211	CP053231	CP053234	CP053245	CP053247	CP053251
CP053258	CP053259	CP053281	CP053284	CP053296	CP053384
CP053600	CP053601	CP053602	CP053603	CP053604	CP053605
CP053607	CP053720	CP053723	CP053730	CP053731	CP053736
CP053785	CP053787	CP053851	CP053852	CP054214	CP054219
CP054224	CP054227	CP054230	CP054232	CP054236	CP054282
CP054314	CP054317	CP054319	CP054325	CP054328	CP054335
CP054343	CP054345	CP054353	CP054363	CP054368	CP054371
CP054372	CP054379	CP054388	CP054407	CP054449	CP054454
CP054457	CP054556	CP054828	CP054940	CP055158	CP055251
CP055256	CP055259	CP055426	CP055438	CP055608	CP055624
CP055649	CP055652	CP055660	CP055669	CP055681	CP055713
CP055718	CP055724	CP055736	CP055748	CP055753	CP055786
CP055796	CP055818	CP055888	CP055918	CP055923	CP055934
CP055941	CP055945	CP055951	CP055958	CP055964	CP055967
CP055971	CP055973	CP055976	CP055981	CP056036	CP056073
CP056077	CP056190	CP056237	CP056242	CP056248	CP056249
CP056263	CP056273	CP056278	CP056282	CP056299	CP056304
CP056315	CP056348	CP056355	CP056357	CP056370	CP056392
CP056396	CP056404	CP056446	CP056470	CP056481	CP056551
CP056556	CP056564	CP056565	CP056569	CP056578	CP056584
CP056594	CP056598	CP056604	CP056608	CP056613	CP056618
CP056620	CP056625	CP056632	CP056642	CP056655	CP056662
CP056786	CP056794	CP056800	CP056810	CP056816	CP056821
CP056842	CP056846	CP056865	CP056867	CP056870	CP056873
CP056876	CP056894	CP056902	CP056914	CP056920	CP056921
CP056928	CP056933	CP056940	CP056947	CP056955	CP056974
CP056985	CP057007	CP057010	CP057012	CP057014	CP057016
CP057018	CP057020	CP057022	CP057024	CP057026	CP057028
CP057031	CP057038	CP057040	CP057045	CP057050	CP057055
CP057057	CP057062	CP057072	CP057075	CP057085	CP057087
CP057090	CP057109	CP057112	CP057115	CP057119	CP057123
CP057126	CP057129	CP057133	CP057137	CP057139	CP057141
CP057144	CP057152	CP057154	CP057160	CP057163	CP057166
CP057167	CP057171	CP057173	CP057175	CP057176	CP057178
CP057185	CP057186	CP057188	CP057190	CP057192	CP057194
CP057200	CP057204	CP057207	CP057223	CP057225	CP057234
CP057247	CP057250	CP057254	CP057259	CP057262	CP057267
CP057270	CP057273	CP057276	CP057281	CP057287	CP057293
CP057302	CP057334	CP057336	CP057340	CP057342	CP057344
CP057345	CP057347	CP057349	CP057354	CP057364	CP057368
CP057371	CP057378	CP057380	CP057396	CP057402	CP057408
CP057417	CP057420	CP057423	CP057429	CP057432	CP057435
CP057438	CP057441	CP057444	CP057447	CP057450	CP057453
CP057455	CP057465	CP057468	CP057470	CP057473	CP057475
CP057479	CP057484	CP057485	CP057487	CP057491	CP057493

Continued on next page

CP057500	CP057507	CP057510	CP057512	CP057516	CP057520
CP057523	CP057525	CP057535	CP057539	CP057545	CP057555
CP057559	CP057570	CP057574	CP057577	CP057580	CP057582
CP057584	CP057587	CP057589	CP057594	CP057597	CP057600
CP057603	CP057605	CP057608	CP057612	CP057615	CP057617
CP057621	CP057626	CP057634	CP057636	CP057638	CP057640
CP057643	CP057645	CP057650	CP057660	CP057665	CP057669
CP057673	CP057677	CP057684	CP057686	CP057690	CP057693
CP057696	CP057705	CP057713	CP057717	CP057725	CP057729
CP057731	CP057738	CP057751	CP057759	CP057767	CP057773
CP057778	CP057784	CP057790	CP057796	CP057802	CP057804
CP057808	CP057812	CP057818	CP057826	CP057829	CP057831
CP057833	CP057838	CP057839	CP057841	CP057843	CP057849
CP057854	CP057860	CP057861	CP057869	CP057873	CP057876
CP057878	CP057879	CP057882	CP057885	CP057888	CP057891
CP057893	CP057900	CP057901	CP057905	CP057906	CP057911
CP057915	CP057916	CP057922	CP057927	CP057930	CP057932
CP057936	CP057941	CP057942	CP057946	CP057951	CP057953
CP057957	CP057959	CP057961	CP057962	CP057964	CP057967
CP057970	CP057973	CP057975	CP057978	CP057980	CP057983
CP057987	CP057990	CP057993	CP057994	CP057995	CP057996
CP058004	CP058007	CP058009	CP058012	CP058014	CP058019
CP058023	CP058025	CP058027	CP058031	CP058063	CP058070
CP058072	CP058074	CP058076	CP058078	CP058080	CP058083
CP058087	CP058090	CP058093	CP058101	CP058104	CP058223
CP058302	CP058308	CP058342	CP058355	CP058571	CP058574
CP058596	CP058618	CP058651	CP058656	CP058661	CP058682
CP058913	CP058948	CP059003	CP059043	CP059119	CP059125
CP059130	CP059137	CP059279	CP059281	CP059283	CP059288
CP059723	CP059835	CP059840	CP059904	CP059907	CP059913
CP059917	CP059922	CP059925	CP059929	CP059931	CP059944
CP059947	CP059953	CP059988	CP060057	CP060061	CP060065
CP060073	CP060075	CP060083	CP060708	CP060709	CP061101
CP061108	CP061185	CP061206	CP061329	CP061337	CP061339
CP061530	CP061749	CP061754	CP061758	CP061764	CP061914
CP062160	CP062203	CP062204	CP062211	CP062228	CP062700
CP062702	CP062705	CP062708	CP062711	CP062713	CP062715
CP062717	CP062719	CP062721	CP062723	CP062725	CP062727
CP062729	CP062731	CP062733	CP062736	CP062739	CP062742
CP062744	CP062746	CP062749	CP062752	CP062755	CP062758
CP062761	CP062763	CP062766	CP062769	CP062771	CP062774
CP062778	CP062780	CP062782	CP062811	CP062815	CP062824
CP062826	CP062832	CP062838	CP062844	CP062851	CP062853
CP062855	CP062863	CP062865	CP062868	CP062871	CP062876
CP062883	CP062889	CP062895	CP062901	CP062908	CP062910
CP062924	CP062927	CP062930	CP062933	CP062967	CP062970
CP063046	CP063153	CP063214	CP063332	CP063341	CP063349
CP063369	CP063459	CP063473	CP063476	CP063479	CP063483
CP063487	CP063492	CP063499	CP063503	CP063511	CP063515
CP063518	CP063711	CP063713	CP063720	CP063729	CP063736
CP063739	CP063742	CP063774	CP063956	CP063958	CP063969
CP063970	CP063979	CP063983	CP064103	CP064167	CP064677
CP064678	CP064679	CP064680	CP064681	CP064682	CP064683
CP064847	CP065136	CP065152	CP065607	CP065611	CP065613
CP065615	CP065619	CP065624	CP065970	CP066032	CP066144
CP066366	CP066748	CP066753	CP066756	CP066759	CP066806
CP066836	CP066844	CP067232	CP067235	CP067241	CP067245
CP067250	CP067265	CP067274	CP067299	CP067303	CP067307
CP067311	CP067342	CP067399	CP067994	CP068035	CP068041
CP068155	CP068279	CP068281	CP068283	CP068394	CP068591
CP068706	CP068795	CP068796	CP068799	CP068800	CP068801
CP068802	CP068803	CP068804	CP068805	CP068806	CP068807
CP068808	CP068809	CP068810	CP068811	CP068812	CP068813
CP068814	CP068815	CP068816	CP068817	CP068818	CP068819
CP068820	CP068821	CP068822	CP068823	CP068824	CP068825
CP068826	CP068827	CP068828	CP068829	CP068830	CP068995
CP069132	CP069133	CP069134	CP069386	CP069438	CP069441
CP069445	CP069447	CP069451	CP069453	CP069459	CP069480
CP069488	CP069500	CP069501	CP069517	CP069522	CP069528
CP069542	CP069549	CP069559	CP069571	CP069583	CP069589
CP069646	CP069657	CP069666	CP069677	CP069682	CP069692
CP069706	CP069707	CP069709	CP069710	CP069712	CP069865
CP069882	CP069890	CP069893	CP069935	CP069945	CP069957

Continued on next page

CP069973	CP069978	CP069980	CP069990	CP069996	CP070007
CP070026	CP070041	CP070045	CP070048	CP070063	CP070066
CP070071	CP070077	CP070098	CP070103	CP070111	CP070115
CP070124	CP070134	CP070148	CP070152	CP070157	CP070162
CP070170	CP070227	CP070232	CP070279	CP070280	CP070393
CP070399	CP070403	CP070593	CP070902	CP070906	CP070914
CP070920	CP070930	CP070962	CP071036	CP071050	CP071073
CP071133	CP071375	CP071436	CP071439	CP071441	CP071521
CP071522	CP071954	CP072054	CP072204	CP072322	CP072539
CP072661	CP072662	CP072663	CP072784	CP072785	CP072786
CP072787	CP072802	CP072858	CP072911	CP072976	CP072980
CP073360	CP073589	CP073591	CP073593	CP073595	CP073597
CP073599	CP073601	CP073603	CP073607	CP073609	CP073613
CP073617	CP073619	CP073621	CP073624	CP073718	CP073719
CP073768	CP074120	CP074576	CP074701	CP075061	CP075062
CP075374	CP075465	CP075627	CP075632	CP075640	CP075649
CP075652	CP075663	CP075665	CP075697	CP075852	CP076123
CP076230	CP076232	CP076235	CP076237	CP076241	CP076243
CP076245	CP076293	CP076301	CP076305	CP076318	CP076344
CP076404	CP076527	CP076645	CP076646	CP076654	CP076687
CP076689	CP076693	CP076697	CP076704	CP076706	CP076709
CP076714	CP077063	CP077215	CP077225	CP077243	CP077250
CP077266	CP077273	CP077281	CP077294	CP077314	CP077325
CP077342	CP077353	CP077361	CP077379	CP077389	CP077396
CP077646	CP077649	CP078137	CP078555	CP078556	CP078565
CP078566	CP078567	CP078568	CP078570	CP078571	CP078572
CP078575	CP078576	CP078577	CP078578	CP078579	CP078581
CP078582	CP078583	CP078584	CP078585	CP078586	CP078587
CP078589	CP078592	CP079735	CP079736	CP079747	CP080001
CP080116	CP080117	CP080118	CP080119	CP080120	CP080121
CP080126	CP080133	CP080139	CP080146	CP080155	CP080163
CP080172	CP080223	CP080233	CP080235	CP080246	CP080253
CP080255	CP080260	CP080370	CP080620	CP080645	CP081007
CP081008	CP081009	CP081010	CP081192	CP081306	CP081677
CP081684	CP081690	CP081698	CP081701	CP081706	CP081711
CP081713	CP081719	CP081724	CP081729	CP081736	CP081742
CP081748	CP081755	CP081762	CP081767	CP081774	CP081782
CP081791	CP081796	CP081805	CP081881	CP082120	CP082127
CP082129	CP082330	CP082349	CP082355	CP082357	CP082824
CP082827	CP082831	CP082949	CP083263	CP083273	CP083410
CP083419	CP083478	CP083481	CP083492	CP083497	CP083503
CP083512	CP083517	CP083530	CP083534	CP083585	CP083701
CP083869	CP083872	CP083877	CP083880	CP083886	CP083888
CP084026	CP084096	CP084529	CP084534	CP084893	CP084894
CP084895	CP084896	CP084897	CP084898	CP084899	CP084900
CP085056	CP085060	CP085063	CP085069	CP085074	CP085183
CP085188	CP085372	CP085488	CP085517	CP085619	CP085622
CP085637	CP085638	CP085842	CP086259	CP086334	CP086336
CP086338	CP086340	CP086342	CP086391	CP086556	CP086616
CP086618	CP086620	CP086622	CP086623	CP086624	CP086625
CP086626	CP086627	CP086662	CP087110	CP087377	CP087570
CP087578	CP088026	CP088028	CP088030	CP088032	CP088034
CP088036	CP088038	CP088040	CP088042	CP088044	CP088046
CP088048	CP088050	CP088052	CP088054	CP088056	CP088058
CP088060	CP088062	CP088064	CP088067	CP088069	CP088071
CP088344	CP088346	CP088348	CP088351	CP088352	CP088356
CP088363	CP088366	CP088369	CP088370	CP088374	CP088381
CP088383	CP088387	CP088390	CP088393	CP088397	CP088400
CP088406	CP088407	CP088408	CP088413	CP088414	CP088417
CP088421	CP088427	CP088433	CP088436	CP088443	CP088451
CP088452	CP088456	CP088461	CP088463	CP088466	CP088468
CP088473	CP088474	CP088479	CP088485	CP088486	CP088490
CP088494	CP088496	CP088500	CP088506	CP088509	CP088512
CP088514	CP088516	CP088520	CP088526	CP088529	CP088534
CP088542	CP088545	CP088547	CP088551	CP088553	CP088555
CP088560	CP088564	CP088566	CP088570	CP088576	CP088578
CP088580	CP088582	CP088584	CP088592	CP088593	CP088596
CP088598	CP088602	CP088607	CP088609	CP088612	CP088613
CP088617	CP088621	CP088624	CP088627	CP088629	CP088632
CP088637	CP088641	CP088643	CP088645	CP088649	CP088652
CP088654	CP088656	CP088658	CP088660	CP088662	CP088665
CP088673	CP088677	CP088681	CP088683	CP088687	CP088692
CP088694	CP088698	CP088701	CP088703	CP088707	CP088710

Continued on next page

CP088714	CP088719	CP088722	CP088723	CP088725	CP088729
CP088734	CP088737	CP088740	CP088744	CP088754	CP088760
CP088764	CP088768	CP088771	CP088773	CP088776	CP088779
CP088782	CP088786	CP088790	CP088795	CP088800	CP088802
CP088804	CP088808	CP088812	CP088818	CP088820	CP088822
CP088826	CP088828	CP088829	CP088833	CP088835	CP088837
CP088839	CP088850	CP088851	CP088855	CP088860	CP088864
CP088866	CP088869	CP088872	CP089031	CP089032	CP089034
CP089036	CP089142	CP089270	CP089272	CP089445	GL878028
GL896790	GL988050	HF572917	HG428755	HG738867	JAADCD010000001
JAAJTL010000001	JAAJTN010000001	JAAJTO010000001	JAAJTP010000001	JAAJTS010000001	JAAJTT010000001
JAAJTU010000001	JAAJTV010000001	JAAKGF010000001	JAALAC010000002	JAATHV010000001	JAATHX010000001
JAATHY010000001	JABWPS010000001	JABXOE010000001	JACDQF010000001	JACDQG010000001	JACGZT020000001
JACIVJ010000001	JACIVK010000001	JACOJH010000001	JACOLT010000001	JACOLU010000001	JACRQJ010000001
JACSXT010000001	JACXXS010000001	JACXYM010000001	JACYGB010000002	JACYGC010000008	JACYGD010000009
JACYGE010000009	JACYGM010000004	JACYGN010000008	JADALV010000001	JADALW010000001	JADDLL010000001
JADDLM010000001	JADDLP010000001	JADDLR010000001	JADDMK010000001	JAEFCJ010000001	JAEFCL010000001
JAEFCM010000001	JAEFCP010000001	JAEFCQ010000001	JAEPRM010000001	JAFBIF010000001	JAFCXR010000001
JAFFIC010000001	JAGDML010000001	JAGEVC010000001	JAGFYA010000002	JAGFYB010000001	JAGFYC010000001
JAGFYF010000001	JAGFZN010000001	JAGFZR010000001	JAGFZU010000001	JAGHKJ010000001	JAHMAB010000001
JAHQKD010000001	JAHQKR010000001	JAHQLA010000001	JAHQLB010000001	JAHSRN010000001	JAHSRO010000001
JAHSRP010000001	JAHSRQ010000001	JAHSRR010000001	JAHSRS010000001	JAHSRT010000001	JAHSRU010000001
JAHSRV010000001	JAHSRX010000001	JAHSRY010000001	JAHSRZ010000001	JAHSSA010000001	JAHSSB010000001
JAHSSC010000001	JAHSSD010000001	JAHSSE010000001	JAINSH010000001	JAINST010000001	JAIQZL010000008
JAJJWC010000001	JAPJ02000001	JASU01000001	JH648165	JJMY02000001	JMJD01000003
KB732454	KB732530	KB732535	KB732574	KB732580	KB732702
KB732854	KB732889	KB732916	KB732931	KB733000	KB733023
KB733040	KB733069	KB733071	KB733111	KB733130	KB733141
KB733172	KB733173	KB733185	KB733201	KB733361	KE136980
KE137028	KE137155	KE137162	KE137211	KE700230	KE700461
KE700478	KE700731	KI929668	KI929682	KI929714	KI929774
KI929782	KI929804	KK583188	KK736296	KQ087687	LAZO01000001
LM993812	LM995446	LN832404	LN874954	LN877770	LORP02000005
LORS02000001	LPWC02000002	LQSL02000003	LQTX02000001	LQUJ02000001	LQVG02000001
LQVV02000005	LR025096	LR025101	LR130532	LR130545	LR130562
LR134000	LR134031	LR134075	LR134079	LR134080	LR134082
LR134220	LR134222	LR134227	LR134228	LR134234	LR134237
LR134239	LR134240	LR134247	LR134248	LR134295	LR134315
LR217818	LR536430	LR536431	LR595691	LR607328	LR607331
LR740758	LR778141	LR778143	LR778146	LR778147	LR778148
LR778151	LR778152	LR778153	LR881940	LR882050	LR882052
LR882057	LR882493	LR882973	LR882978	LR882990	LR882997
LR883006	LR883012	LR883050	LR883965	LR890198	LR890270
LR890288	LR890299	LR890326	LR890334	LR890349	LR890405
LR890410	LR890466	LR890508	LR890536	LR890603	LR890651
LR890693	LR898868	LR898874	LRDE01000001	LRDF01000001	LS483296
LS483297	LS483303	LS992166	LS992168	LS992171	LS992180
LS992185	LS992190	LS992192	LS998785	LS999560	LSUP01000001
LT601384	LT632320	LT795502	LT827011	LT838200	LT883142
LT903847	LT906474	LUYD01000001	LVRH02000001	LVRJ02000001	LVSP02000001
LWDC01000001	MBNU01000003	MBNW01000001	MBNX01000003	MBNY01000001	MCRE01000001
MDLJ02000001	MIIL01000001	MPGR01000001	MPGS01000001	MPGT01000001	MRDN01000001
NBIY01000001	NC_002695	NC_011601	NC_011741	NC_011742	NC_011751
NC_012759	NC_012947	NC_013364	NC_013941	NC_017626	NC_017631
NC_017632	NC_017660	NC_018650	NC_018661	NC_022370	NHYO01000001
NHYP01000001	NHYQ01000001	NHYR01000001	NHYS01000001	NHYT01000001	NIFW01000002
NJEX02000002	NJIR01000001	NQCP02000001	NQCR02000001	NQLI02000001	NWVR01000001
NXER01000001	OU342919	OU349837	OU349838	OU349839	OU349840
OU349841	OU349842	OU349843	OU349844	OU349845	OU349846
OU349848	OU349849	OU356913	PDDT01000002	PDED01000001	PDEK01000002
PDLD01000001	PDLF01000002	PDLJ01000002	PSQV01000007	PSQW01000002	PSQX01000006
PVMG01000001	QBDM01000001	QLVH01000001	QMGK01000003	QSMQ02000006	QSMV02000001
QWLO01000001	QXTI01000001	RKJE01000005	RKJJ01000004	RKJK01000003	RKJN01000003
RRCS01000001	RYCV01000003	SESS01000001	SEVF01000002	SEVL01000002	SEVN01000002
SEVP01000003	SEVQ01000001	SEVT01000005	SEVU01000007	SEVW01000003	SEVY01000001
SEVZ01000001	SEWA01000004	SGYH01000001	SGYI01000001	SSWI01000001	SWKA01000005
UASF01000007	UEFM01000001	UFYL01000003	UFYZ01000002	UFZD01000005	UFZE01000001
UFZF01000006	UFZG01000001	UFZI01000001	UFZV01000003	UFZW01000006	UGAD01000001
UGAK01000003	UGAL01000001	UGAM01000005	UGAO01000001	UGAS01000002	UGAT01000002
UGAU01000003	UGAV01000006	UGAX01000001	UGAY01000002	UGAZ01000001	UGBA01000002
UGBD01000002	UGBE01000003	UGBJ01000001	UGBK01000002	UGBL01000001	UGBM01000005
UGBP01000004	UGBQ01000003	UGBS01000001	UGBV01000003	UGBX01000001	UGCB01000003
UGCI01000003	UGCW01000003	UGCY01000004	UGDG01000002	UGDO01000002	UGDR01000001

Continued on next page

UGDU01000003	UGDV01000004	UGDW01000001	UGEC01000002	UGEE01000003	UGES01000005
UGEU01000002	UGEV01000002	UGEZ01000005	UGFA01000002	UGFB01000004	UGFD01000001
UGFL01000003	UGFO01000006	UGFR01000001	UGFT01000003	UGFV01000001	UGFZ01000001
UGGA01000003	UGGG01000003	UGGI01000001	UWVA01000001	UWVG01000001	UWYG01000001
UYIJ01000003	VCAQ01000001	VCYN01000001	VCYX01000001	VCYY01000001	VCZH01000001
VCZK01000001	VCZP01000001	VCZT01000001	VDAA01000002	VDAW01000001	VDBF01000002
VDBH01000001	VFAG01000002	WIPD02000001	WMHS01000001	WMLE01000001	WNKN01000001
WSNS01000001	WXYW01000001				

Table S5. GenBank accession numbers of sequences used for *E. coli* icd analysis.

AAKB02000001	ABHK02000001	AM946981	AP017610	AP017617	AP017620
AP018488	AP018796	AP018802	AP018808	AP019189	AP019675
AP019761	AP019856	AP021890	AP021891	AP021892	AP021893
AP021894	AP021895	AP021896	AP021914	AP021933	AP021935
AP021944	AP021946	AP021963	AP021998	AP022003	AP022030
AP022032	AP022036	AP022044	AP022049	AP022064	AP022069
AP022087	AP022095	AP022098	AP022105	AP022107	AP022120
AP022159	AP022161	AP022171	AP022173	AP022177	AP022207
AP022215	AP022221	AP022222	AP022245	AP022261	AP022287
AP022295	AP022298	AP022326	AP022351	AP022362	AP022409
AP022478	AP022482	AP022525	AP022533	AP022536	AP022540
AP022549	AP022811	AP022815	AP023190	AP023219	AP023226
AP023230	AP023235	AP023286	AP023427	AP024112	AP024114
AP024123	AP024126	AP024130	AP024131	AP024205	AP024471
AP024473	AP024475	AP024478	AP024560	AP024561	AP024921
AP024924	AP025205	AP025220	APIN01000002	AXLH01000003	BA000007
BNEX01000001	BNFC01000001	BNFH01000001	BNFJ01000001	BNFK01000001	BNFL01000001
BNFN01000001	BNFQ01000001	BNIP01000001	BNIQ01000001	BNIR01000001	BNIS01000001
BNIT01000001	BNIV01000001	BNIW01000001	BNIZ01000001	BNJA01000001	BNJB01000001
BNJC01000001	BNSX01000001	CABEEY010000003	CABFNM010000001	CABWHO010000003	CABWIU010000001
CABWIX010000001	CABWJF010000003	CABWJL010000001	CABWJO010000005	CACRWJ010000001	CACRWK010000001
CACRWL010000001	CACRWM010000001	CACRWO010000001	CACRWV010000001	CADDWP010000001	CADDWR010000001
CADDWS010000001	CADDWT010000001	CADDWW010000001	CADDWX010000001	CADDWY010000002	CADDWZ010000001
CADDXF010000001	CADDXG010000001	CADDXH010000001	CADDXI010000001	CADDXJ010000001	CADDXL010000002
CADDXM010000001	CADDXN010000001	CADDXO010000001	CADDXP010000001	CADDXQ010000002	CADDXS010000001
CADEAI010000002	CAJGEG010000001	CAJGEH010000001	CAJGEI010000001	CAJGWN010000001	CAJMUK010000001
CAJNTK010000001	CAJOCA010000001	CAJOCB010000001	CAJSJQ010000001	CM000662	CM003707
CM007915	CP001509	CP002967	CP002970	CP005930	CP006027
CP006632	CP006636	CP006698	CP006830	CP007136	CP007275
CP007391	CP007393	CP007491	CP007592	CP007594	CP008805
CP009072	CP009106	CP009166	CP009273	CP009644	CP009685
CP009789	CP009859	CP010116	CP010117	CP010119	CP010122
CP010125	CP010132	CP010133	CP010134	CP010137	CP010140
CP010143	CP010145	CP010148	CP010150	CP010151	CP010160
CP010163	CP010167	CP010169	CP010170	CP010172	CP010177
CP010178	CP010180	CP010183	CP010206	CP010219	CP010221
CP010226	CP010228	CP010229	CP010231	CP010235	CP010236
CP010237	CP010238	CP010240	CP010242	CP010304	CP010315
CP010344	CP010371	CP010439	CP010441	CP010443	CP010444
CP010585	CP010876	CP011061	CP011124	CP011134	CP011320
CP011321	CP011322	CP011323	CP011324	CP011331	CP011342
CP011343	CP011416	CP011495	CP011915	CP012126	CP012376
CP012379	CP012380	CP012633	CP012635	CP012693	CP012802
CP012868	CP012870	CP013025	CP013029	CP013031	CP013112
CP013185	CP013835	CP013952	CP014092	CP014270	CP014272
CP014314	CP014348	CP014488	CP014497	CP014522	CP014583
CP014667	CP015020	CP015023	CP015069	CP015074	CP015076
CP015085	CP015138	CP015228	CP015229	CP015240	CP015241
CP015831	CP015832	CP015834	CP015842	CP015843	CP015846
CP015855	CP015912	CP015995	CP016007	CP016358	CP016404
CP016497	CP016628	CP016755	CP017100	CP017220	CP017438
CP017442	CP017446	CP017631	CP017844	CP017980	CP018103
CP018206	CP018237	CP018243	CP018247	CP018250	CP018252
CP018323	CP018770	CP018840	CP018948	CP018953	CP018957
CP018965	CP018970	CP018983	CP018991	CP019008	CP019012
CP019015	CP019051	CP019243	CP019245	CP019256	CP019259
CP019267	CP019271	CP019280	CP019355	CP019359	CP019361
CP019392	CP019455	CP019558	CP019560	CP019778	CP019903
CP019944	CP019953	CP019961	CP020025	CP020048	CP020055
CP020058	CP020092	CP020106	CP020107	CP020368	CP020495

Continued on next page

CP020509	CP020516	CP020543	CP020545	CP020835	CP020933
CP021175	CP021179	CP021193	CP021335	CP021339	CP021454
CP021532	CP021691	CP021722	CP021726	CP021840	CP021935
CP022050	CP022086	CP022164	CP022229	CP022407	CP022414
CP022466	CP022664	CP022686	CP022689	CP022730	CP022912
CP023061	CP023165	CP023200	CP023201	CP023258	CP023346
CP023349	CP023353	CP023357	CP023359	CP023366	CP023377
CP023531	CP023535	CP023541	CP023644	CP023673	CP023749
CP023815	CP023820	CP023844	CP023849	CP023853	CP023870
CP024056	CP024127	CP024131	CP024134	CP024147	CP024223
CP024228	CP024232	CP024243	CP024245	CP024256	CP024257
CP024263	CP024269	CP024273	CP024275	CP024278	CP024289
CP024293	CP024299	CP024479	CP024618	CP024650	CP024659
CP024661	CP024667	CP024717	CP024801	CP024821	CP024830
CP024851	CP024855	CP024859	CP024886	CP024889	CP024992
CP024997	CP025036	CP025048	CP025328	CP025401	CP025573
CP025627	CP025747	CP025753	CP025840	CP025847	CP025849
CP025851	CP025856	CP025859	CP025862	CP025865	CP025881
CP025903	CP025910	CP025913	CP025916	CP025920	CP025950
CP026199	CP026399	CP026473	CP026491	CP026580	CP026723
CP026755	CP026853	CP026929	CP026932	CP026935	CP026939
CP027060	CP027126	CP027134	CP027140	CP027205	CP027221
CP027255	CP027307	CP027310	CP027312	CP027317	CP027323
CP027325	CP027328	CP027331	CP027335	CP027338	CP027347
CP027351	CP027355	CP027361	CP027362	CP027363	CP027366
CP027368	CP027371	CP027373	CP027380	CP027387	CP027388
CP027394	CP027435	CP027437	CP027442	CP027445	CP027447
CP027449	CP027452	CP027457	CP027459	CP027461	CP027462
CP027520	CP027534	CP027544	CP027548	CP027550	CP027555
CP027573	CP027579	CP027582	CP027584	CP027586	CP027587
CP027591	CP027597	CP027599	CP027701	CP027766	CP027851
CP028110	CP028112	CP028116	CP028122	CP028126	CP028166
CP028192	CP028304	CP028305	CP028306	CP028307	CP028309
CP028310	CP028320	CP028379	CP028381	CP028429	CP028432
CP028589	CP028590	CP028592	CP028600	CP028603	CP028607
CP028609	CP028614	CP028654	CP028674	CP028677	CP028685
CP028693	CP028695	CP028700	CP028702	CP028703	CP028714
CP029057	CP029108	CP029115	CP029122	CP029164	CP029239
CP029240	CP029369	CP029371	CP029420	CP029574	CP029579
CP029630	CP029741	CP029973	CP030111	CP030240	CP030331
CP030337	CP030767	CP030768	CP030939	CP031105	CP031134
CP031157	CP031231	CP031256	CP031293	CP031321	CP031341
CP031343	CP031349	CP031546	CP031609	CP031653	CP031706
CP031892	CP031910	CP031912	CP031916	CP031919	CP031922
CP032066	CP032073	CP032145	CP032201	CP032204	CP032237
CP032261	CP032265	CP032426	CP032515	CP032667	CP032789
CP032791	CP032793	CP032795	CP032797	CP032801	CP032803
CP032805	CP032808	CP032811	CP032879	CP032892	CP032936
CP032989	CP033092	CP033096	CP033158	CP033250	CP033401
CP033605	CP033635	CP033762	CP033850	CP033884	CP034106
CP034384	CP034389	CP034399	CP034404	CP034589	CP034595
CP034658	CP034738	CP034792	CP034794	CP034797	CP034799
CP034801	CP034803	CP034806	CP034808	CP034843	CP034953
CP034966	CP035317	CP035318	CP035322	CP035325	CP035331
CP035350	CP035360	CP035366	CP035377	CP035476	CP035477
CP035486	CP035498	CP035516	CP035545	CP035751	CP035767
CP035770	CP035772	CP035812	CP035817	CP035819	CP035822
CP035832	CP035836	CP035846	CP035850	CP035860	CP035865
CP035870	CP035874	CP035879	CP035882	CP035884	CP036202
CP036245	CP037449	CP037857	CP037910	CP037943	CP037945
CP038138	CP038282	CP038284	CP038287	CP038295	CP038302
CP038305	CP038309	CP038313	CP038319	CP038336	CP038342
CP038349	CP038351	CP038353	CP038360	CP038363	CP038372
CP038374	CP038376	CP038383	CP038386	CP038394	CP038408
CP038412	CP038416	CP038421	CP038423	CP038425	CP038428
CP038494	CP038496	CP038505	CP038859	CP039403	CP039404
CP039834	CP040090	CP040107	CP040263	CP040269	CP040307
CP040309	CP040311	CP040313	CP040314	CP040316	CP040390
CP040397	CP040456	CP040572	CP040643	CP040664	CP040667
CP040886	CP041002	CP041031	CP041304	CP041337	CP041359
CP041411	CP041422	CP041425	CP041429	CP041431	CP041433
CP041435	CP041437	CP041442	CP041448	CP041452	CP041520
CP041522	CP041526	CP041529	CP041532	CP041535	CP041538

Continued on next page

CP041541	CP041544	CP041550	CP041556	CP041559	CP041563
CP041572	CP041578	CP041581	CP041623	CP041627	CP041628
CP041678	CP041749	CP041955	CP041996	CP042246	CP042250
CP042298	CP042336	CP042350	CP042470	CP042588	CP042843
CP042845	CP042846	CP042878	CP042892	CP042901	CP042969
CP043033	CP043181	CP043187	CP043199	CP043213	CP043217
CP043414	CP043478	CP043486	CP043487	CP043539	CP043541
CP043542	CP043852	CP043950	CP044140	CP044143	CP044145
CP044148	CP044293	CP044311	CP044312	CP044314	CP044315
CP044403	CP044410	CP045206	CP045209	CP045277	CP045457
CP045712	CP045741	CP045827	CP045975	CP045977	CP046000
CP046009	CP046259	CP046396	CP046416	CP046527	CP046539
CP046716	CP047002	CP047010	CP047074	CP047075	CP047077
CP047078	CP047079	CP047127	CP047277	CP047378	CP047405
CP047571	CP047576	CP047594	CP047600	CP047609	CP047658
CP047662	CP047665	CP047710	CP048010	CP048025	CP048107
CP048290	CP048304	CP048310	CP048322	CP048326	CP048330
CP048337	CP048344	CP048359	CP048367	CP048371	CP048376
CP048439	CP048603	CP048604	CP048605	CP048606	CP048647
CP048821	CP048853	CP048855	CP048857	CP048860	CP048863
CP048865	CP048867	CP048870	CP048873	CP048915	CP048920
CP048934	CP048935	CP049050	CP049077	CP049081	CP049085
CP049086	CP049101	CP049118	CP049196	CP049197	CP049198
CP049201	CP049204	CP049299	CP049348	CP049353	CP049839
CP049845	CP049852	CP049936	CP049943	CP049967	CP049979
CP050031	CP050036	CP050040	CP050044	CP050047	CP050173
CP050193	CP050195	CP050196	CP050197	CP050198	CP050200
CP050202	CP050204	CP050206	CP050208	CP050209	CP050211
CP050213	CP050216	CP050217	CP050218	CP050219	CP050220
CP050221	CP050222	CP050289	CP050382	CP050498	CP050646
CP050862	CP050865	CP051158	CP051219	CP051222	CP051609
CP051615	CP051631	CP051688	CP051692	CP051694	CP051698
CP051700	CP051706	CP051711	CP051714	CP051716	CP051719
CP051725	CP051727	CP051733	CP051735	CP051738	CP051744
CP051749	CP051751	CP051753	CP051765	CP051776	CP051778
CP051782	CP051786	CP051790	CP051792	CP051796	CP051798
CP051800	CP051802	CP051804	CP051806	CP051808	CP051811
CP051814	CP051816	CP051819	CP051821	CP051824	CP051827
CP051830	CP051833	CP051837	CP051849	CP052057	CP052877
CP053079	CP053080	CP053211	CP053231	CP053234	CP053245
CP053247	CP053251	CP053258	CP053259	CP053281	CP053284
CP053296	CP053384	CP053600	CP053601	CP053602	CP053603
CP053604	CP053605	CP053607	CP053720	CP053723	CP053730
CP053731	CP053736	CP053785	CP053787	CP053851	CP053852
CP054214	CP054219	CP054224	CP054227	CP054230	CP054232
CP054236	CP054282	CP054314	CP054317	CP054319	CP054328
CP054343	CP054345	CP054353	CP054363	CP054368	CP054371
CP054372	CP054379	CP054388	CP054407	CP054449	CP054454
CP054556	CP054828	CP054940	CP055158	CP055251	CP055256
CP055259	CP055426	CP055438	CP055608	CP055624	CP055649
CP055652	CP055660	CP055669	CP055681	CP055713	CP055718
CP055724	CP055736	CP055748	CP055753	CP055786	CP055796
CP055818	CP055888	CP055918	CP055923	CP055934	CP055941
CP055945	CP055951	CP055958	CP055964	CP055967	CP055971
CP055973	CP055976	CP055981	CP056036	CP056073	CP056077
CP056190	CP056237	CP056242	CP056248	CP056249	CP056263
CP056273	CP056278	CP056282	CP056299	CP056304	CP056315
CP056348	CP056355	CP056357	CP056370	CP056392	CP056396
CP056404	CP056446	CP056470	CP056481	CP056551	CP056556
CP056564	CP056565	CP056569	CP056578	CP056584	CP056594
CP056598	CP056604	CP056608	CP056613	CP056618	CP056620
CP056625	CP056632	CP056642	CP056655	CP056662	CP056786
CP056794	CP056800	CP056810	CP056816	CP056821	CP056842
CP056846	CP056865	CP056867	CP056870	CP056873	CP056876
CP056894	CP056902	CP056914	CP056920	CP056921	CP056928
CP056933	CP056940	CP056947	CP056955	CP056974	CP056985
CP057007	CP057010	CP057012	CP057014	CP057016	CP057018
CP057020	CP057022	CP057024	CP057026	CP057028	CP057031
CP057038	CP057040	CP057045	CP057050	CP057055	CP057057
CP057062	CP057072	CP057075	CP057085	CP057087	CP057090
CP057109	CP057112	CP057115	CP057119	CP057123	CP057126
CP057129	CP057133	CP057137	CP057139	CP057141	CP057144
CP057152	CP057154	CP057160	CP057163	CP057166	CP057167

Continued on next page

CP057171	CP057173	CP057175	CP057176	CP057178	CP057185
CP057186	CP057188	CP057190	CP057192	CP057194	CP057200
CP057204	CP057207	CP057223	CP057225	CP057234	CP057247
CP057250	CP057254	CP057259	CP057262	CP057267	CP057270
CP057273	CP057276	CP057281	CP057287	CP057293	CP057302
CP057334	CP057336	CP057340	CP057342	CP057344	CP057345
CP057347	CP057349	CP057354	CP057364	CP057368	CP057371
CP057378	CP057380	CP057396	CP057402	CP057408	CP057417
CP057420	CP057423	CP057429	CP057432	CP057435	CP057438
CP057441	CP057444	CP057447	CP057450	CP057453	CP057455
CP057465	CP057468	CP057470	CP057473	CP057475	CP057479
CP057484	CP057485	CP057487	CP057491	CP057493	CP057500
CP057507	CP057510	CP057512	CP057516	CP057520	CP057523
CP057525	CP057535	CP057539	CP057545	CP057555	CP057559
CP057570	CP057574	CP057577	CP057580	CP057582	CP057584
CP057587	CP057589	CP057594	CP057597	CP057600	CP057603
CP057605	CP057608	CP057612	CP057615	CP057617	CP057621
CP057626	CP057634	CP057636	CP057638	CP057640	CP057643
CP057645	CP057650	CP057660	CP057665	CP057669	CP057673
CP057677	CP057684	CP057686	CP057690	CP057693	CP057696
CP057705	CP057713	CP057717	CP057725	CP057729	CP057731
CP057738	CP057751	CP057759	CP057767	CP057773	CP057778
CP057784	CP057790	CP057796	CP057802	CP057804	CP057808
CP057812	CP057818	CP057826	CP057829	CP057831	CP057833
CP057838	CP057839	CP057841	CP057843	CP057849	CP057854
CP057860	CP057861	CP057869	CP057873	CP057876	CP057878
CP057879	CP057882	CP057885	CP057888	CP057891	CP057893
CP057900	CP057901	CP057905	CP057906	CP057911	CP057915
CP057916	CP057922	CP057927	CP057930	CP057932	CP057936
CP057941	CP057942	CP057946	CP057951	CP057953	CP057957
CP057959	CP057961	CP057962	CP057964	CP057967	CP057970
CP057973	CP057975	CP057978	CP057980	CP057983	CP057987
CP057990	CP057993	CP057994	CP057995	CP057996	CP058004
CP058007	CP058009	CP058012	CP058014	CP058019	CP058023
CP058025	CP058027	CP058031	CP058063	CP058070	CP058072
CP058074	CP058076	CP058078	CP058080	CP058083	CP058087
CP058090	CP058093	CP058101	CP058104	CP058223	CP058302
CP058308	CP058342	CP058355	CP058571	CP058574	CP058596
CP058618	CP058651	CP058656	CP058661	CP058682	CP058913
CP058948	CP059003	CP059043	CP059119	CP059125	CP059130
CP059137	CP059279	CP059281	CP059283	CP059288	CP059723
CP059835	CP059904	CP059907	CP059913	CP059917	CP059922
CP059925	CP059929	CP059931	CP059944	CP059947	CP059953
CP060057	CP060061	CP060065	CP060073	CP060075	CP060083
CP060121	CP060708	CP060709	CP061101	CP061108	CP061185
CP061206	CP061329	CP061337	CP061339	CP061530	CP061749
CP061754	CP061758	CP061764	CP061914	CP062160	CP062203
CP062204	CP062211	CP062228	CP062249	CP062700	CP062702
CP062705	CP062708	CP062711	CP062713	CP062715	CP062717
CP062719	CP062721	CP062723	CP062725	CP062727	CP062729
CP062731	CP062733	CP062736	CP062739	CP062742	CP062744
CP062746	CP062749	CP062752	CP062755	CP062758	CP062761
CP062763	CP062766	CP062769	CP062771	CP062774	CP062778
CP062780	CP062782	CP062811	CP062815	CP062824	CP062826
CP062832	CP062838	CP062844	CP062851	CP062853	CP062855
CP062863	CP062865	CP062868	CP062871	CP062876	CP062883
CP062889	CP062895	CP062901	CP062908	CP062910	CP062924
CP062927	CP062930	CP062933	CP062967	CP062970	CP063046
CP063153	CP063332	CP063341	CP063349	CP063369	CP063459
CP063473	CP063476	CP063479	CP063483	CP063487	CP063492
CP063499	CP063503	CP063511	CP063515	CP063518	CP063711
CP063713	CP063720	CP063729	CP063736	CP063739	CP063742
CP063774	CP063956	CP063958	CP063969	CP063970	CP063979
CP064103	CP064167	CP064677	CP064678	CP064679	CP064680
CP064681	CP064682	CP064683	CP064847	CP065136	CP065152
CP065607	CP065611	CP065613	CP065615	CP065619	CP065624
CP065970	CP066032	CP066144	CP066366	CP066748	CP066753
CP066756	CP066759	CP066806	CP066836	CP066844	CP067232
CP067235	CP067241	CP067245	CP067250	CP067265	CP067274
CP067299	CP067303	CP067307	CP067311	CP067342	CP067399
CP067994	CP068035	CP068041	CP068155	CP068279	CP068281
CP068283	CP068394	CP068591	CP068706	CP068795	CP068796
CP068799	CP068800	CP068801	CP068802	CP068803	CP068804

Continued on next page

CP068805	CP068806	CP068807	CP068808	CP068809	CP068810
CP068811	CP068812	CP068813	CP068814	CP068815	CP068816
CP068817	CP068818	CP068819	CP068820	CP068821	CP068822
CP068823	CP068824	CP068825	CP068826	CP068827	CP068828
CP068829	CP068830	CP068995	CP069132	CP069133	CP069134
CP069386	CP069438	CP069441	CP069445	CP069447	CP069451
CP069453	CP069459	CP069480	CP069488	CP069500	CP069501
CP069517	CP069522	CP069528	CP069542	CP069549	CP069559
CP069571	CP069583	CP069589	CP069646	CP069657	CP069666
CP069677	CP069682	CP069692	CP069706	CP069707	CP069709
CP069710	CP069712	CP069865	CP069882	CP069890	CP069893
CP069935	CP069945	CP069957	CP069973	CP069978	CP069980
CP069990	CP069996	CP070007	CP070026	CP070041	CP070045
CP070048	CP070063	CP070066	CP070071	CP070077	CP070098
CP070103	CP070111	CP070115	CP070124	CP070134	CP070148
CP070152	CP070157	CP070162	CP070170	CP070227	CP070232
CP070279	CP070280	CP070393	CP070399	CP070403	CP070593
CP070902	CP070906	CP070914	CP070920	CP070930	CP070962
CP071036	CP071050	CP071073	CP071133	CP071375	CP071436
CP071439	CP071441	CP071521	CP071522	CP071954	CP072054
CP072204	CP072322	CP072539	CP072661	CP072662	CP072663
CP072784	CP072785	CP072786	CP072787	CP072802	CP072858
CP072911	CP072942	CP072976	CP072980	CP072986	CP073360
CP073589	CP073591	CP073593	CP073595	CP073597	CP073599
CP073601	CP073603	CP073607	CP073613	CP073617	CP073619
CP073621	CP073624	CP073718	CP073719	CP073768	CP074576
CP074701	CP075061	CP075062	CP075374	CP075469	CP075474
CP075480	CP075484	CP075627	CP075632	CP075640	CP075649
CP075652	CP075663	CP075665	CP075697	CP075852	CP076123
CP076230	CP076232	CP076235	CP076237	CP076241	CP076243
CP076245	CP076293	CP076301	CP076305	CP076318	CP076344
CP076404	CP076527	CP076645	CP076646	CP076654	CP076687
CP076689	CP076693	CP076697	CP076704	CP076706	CP076709
CP076714	CP077063	CP077215	CP077225	CP077243	CP077250
CP077266	CP077273	CP077281	CP077294	CP077314	CP077325
CP077342	CP077353	CP077361	CP077379	CP077389	CP077396
CP077646	CP077649	CP078137	CP078555	CP078556	CP078557
CP078558	CP078565	CP078566	CP078567	CP078568	CP078571
CP078572	CP078573	CP078574	CP078575	CP078577	CP078578
CP078580	CP078581	CP078582	CP078583	CP078589	CP078592
CP079735	CP079736	CP079747	CP080116	CP080117	CP080118
CP080119	CP080120	CP080121	CP080126	CP080133	CP080139
CP080146	CP080155	CP080163	CP080172	CP080223	CP080233
CP080235	CP080253	CP080255	CP080260	CP080370	CP080620
CP080645	CP081007	CP081008	CP081009	CP081010	CP081192
CP081306	CP081677	CP081684	CP081690	CP081698	CP081701
CP081706	CP081711	CP081713	CP081719	CP081724	CP081729
CP081736	CP081742	CP081748	CP081755	CP081762	CP081767
CP081774	CP081782	CP081791	CP081796	CP081805	CP081881
CP082120	CP082127	CP082129	CP082349	CP082355	CP082357
CP082824	CP082827	CP082831	CP082949	CP083263	CP083273
CP083410	CP083419	CP083478	CP083481	CP083492	CP083497
CP083503	CP083512	CP083517	CP083530	CP083534	CP083585
CP083701	CP083869	CP083872	CP083877	CP083880	CP083886
CP083888	CP084026	CP084096	CP084529	CP084534	CP084893
CP084894	CP084895	CP084896	CP084897	CP084898	CP084899
CP085056	CP085060	CP085063	CP085069	CP085074	CP085183
CP085188	CP085372	CP085488	CP085517	CP085619	CP085622
CP085637	CP085638	CP085842	CP086259	CP086334	CP086336
CP086338	CP086340	CP086342	CP086391	CP086556	CP086616
CP086618	CP086620	CP086622	CP086623	CP086624	CP086625
CP086626	CP086627	CP086662	CP087110	CP087377	CP087570
CP087578	CP087958	CP088026	CP088028	CP088030	CP088032
CP088034	CP088036	CP088038	CP088040	CP088042	CP088044
CP088046	CP088048	CP088050	CP088052	CP088054	CP088056
CP088058	CP088060	CP088062	CP088064	CP088067	CP088069
CP088071	CP088344	CP088346	CP088348	CP088351	CP088352
CP088356	CP088363	CP088366	CP088369	CP088370	CP088374
CP088381	CP088383	CP088387	CP088390	CP088393	CP088400
CP088406	CP088407	CP088408	CP088413	CP088414	CP088417
CP088421	CP088427	CP088433	CP088436	CP088443	CP088451
CP088452	CP088456	CP088461	CP088463	CP088466	CP088468
CP088473	CP088474	CP088479	CP088485	CP088486	CP088490

Continued on next page

CP088494	CP088496	CP088500	CP088506	CP088509	CP088512
CP088514	CP088516	CP088520	CP088526	CP088529	CP088534
CP088542	CP088545	CP088547	CP088551	CP088553	CP088555
CP088560	CP088564	CP088566	CP088570	CP088576	CP088578
CP088580	CP088582	CP088592	CP088593	CP088596	CP088598
CP088602	CP088607	CP088609	CP088612	CP088613	CP088617
CP088621	CP088624	CP088627	CP088629	CP088632	CP088637
CP088641	CP088643	CP088645	CP088649	CP088652	CP088654
CP088656	CP088658	CP088662	CP088665	CP088673	CP088677
CP088681	CP088683	CP088687	CP088692	CP088694	CP088698
CP088701	CP088703	CP088707	CP088710	CP088714	CP088722
CP088723	CP088725	CP088729	CP088734	CP088737	CP088740
CP088744	CP088754	CP088760	CP088764	CP088768	CP088771
CP088773	CP088776	CP088779	CP088782	CP088786	CP088790
CP088795	CP088800	CP088802	CP088804	CP088808	CP088812
CP088818	CP088820	CP088822	CP088826	CP088828	CP088829
CP088833	CP088835	CP088837	CP088839	CP088850	CP088851
CP088855	CP088860	CP088864	CP088866	CP088869	CP088872
CP089031	CP089032	CP089034	CP089036	CP089142	CP089270
CP089272	CP089445	GL878028	GL896790	GL988050	HF572917
HG738867	JAAJTL010000001	JAAJTN010000001	JAAJTO010000001	JAAJTP010000001	JAAJTR010000001
JAAJTS010000001	JAAJTT010000001	JAAJTU010000001	JAAJTV010000001	JAAKGF010000001	JAALAC010000002
JAANTD010000001	JAATHU010000001	JAATHX010000001	JAATHY010000001	JABWPS010000001	JACDQE010000001
JACDQF010000001	JACDQG010000001	JACGZM020000001	JACIVJ010000001	JACIVK010000001	JACOJH010000001
JACOLT010000001	JACOLU010000001	JACRQJ010000001	JACSXT010000001	JACXXU010000001	JACXXV010000001
JACXYM010000001	JACYGB010000002	JACYGC010000008	JACYGD010000009	JACYGE010000009	JACYGM010000004
JACYGN010000008	JADALV010000001	JADALW010000001	JADDLL010000001	JADDLM010000001	JADDLN010000001
JADDLP010000001	JADDLR010000001	JADDMK010000001	JADDML010000001	JAEFCJ010000001	JAEFCL010000001
JAEFCM010000001	JAEFCO010000001	JAEFCP010000001	JAEFCQ010000001	JAEPRM010000001	JAFBIF010000001
JAFCXR010000001	JAFFIC010000001	JAGEVC010000001	JAGFYA010000002	JAGFYB010000001	JAGFYC010000001
JAGFYF010000001	JAGFZN010000001	JAGFZR010000001	JAGFZU010000001	JAHMAB010000001	JAHQKD010000001
JAHQKR010000001	JAHQKS010000001	JAHQLA010000001	JAHQLB010000001	JAHSRN010000001	JAHSRO010000001
JAHSRP010000001	JAHSRQ010000001	JAHSRR010000001	JAHSRS010000001	JAHSRT010000001	JAHSRU010000001
JAHSRV010000001	JAHSRW010000001	JAHSRX010000001	JAHSRY010000001	JAHSRZ010000001	JAHSSA010000001
JAHSSB010000001	JAHSSC010000001	JAHSSD010000001	JAHSSE010000001	JAINSH010000001	JAINTH010000001
JAIQZL010000008	JAJBZO010000001	JAJJVT010000001	JAJJVV010000001	JAJJWB010000001	JAJJWE010000001
JAJJWF010000001	JASQ01000001	JASU01000001	JH648165	JJMY02000001	KB732454
KB732535	KB732574	KB732702	KB732916	KB732993	KB733023
KB733040	KB733071	KB733078	KB733130	KB733135	KB733141
KB733172	KB733173	KB733185	KB733201	KB733361	KE136980
KE137028	KE137162	KE137211	KE700100	KE700230	KE700461
KE700478	KE700541	KE700731	KI929668	KI929682	KI929714
KI929774	KI929787	KI929804	KK583188	KK736296	KQ087662
KQ087687	KQ087890	LM993812	LM995446	LN874954	LN877770
LORS02000001	LQSL02000003	LQTX02000001	LQUJ02000001	LQVG02000001	LR025096
LR025101	LR130532	LR130545	LR130562	LR130564	LR134031
LR134075	LR134079	LR134080	LR134082	LR134220	LR134222
LR134234	LR134239	LR134248	LR134295	LR536430	LR595691
LR607328	LR607331	LR740758	LR778141	LR778142	LR778143
LR778147	LR778148	LR778149	LR778150	LR778151	LR778153
LR782232	LR881940	LR882050	LR882052	LR882057	LR882493
LR882973	LR882990	LR882997	LR883006	LR883012	LR883050
LR883965	LR890270	LR890299	LR890326	LR890334	LR890349
LR890410	LR890466	LR890508	LR890536	LR890603	LR890606
LR890651	LR890693	LR898868	LR898874	LRDE01000001	LRDF01000001
LS483297	LS992166	LS992168	LS992171	LS992180	LS992185
LS992190	LS992192	LS998785	LS999560	LSUP01000001	LT594504
LT601384	LT632320	LT795502	LT827011	LT838200	LT883142
LT903847	LUYD01000001	LVRJ02000001	LVSP02000001	LWDC01000001	MBNU01000003
MBNW01000001	MBNX01000003	MBNY01000001	MCRE01000001	MDLJ02000001	MIIL01000001
MPGR01000001	MPGS01000001	MRDN01000001	NBIY01000001	NC_002695	NC_004431
NC_007779	NC_007946	NC_009801	NC_010498	NC_011353	NC_011601
NC_011745	NC_011748	NC_011750	NC_012759	NC_012947	NC_012967
NC_013008	NC_013361	NC_013654	NC_017626	NC_017628	NC_017631
NC_017632	NC_017641	NC_017646	NC_017652	NC_017656	NC_017660
NC_017906	NC_018650	NC_018658	NC_018661	NC_020163	NC_022370
NGSF01000001	NHYO01000001	NHYP01000001	NHYQ01000001	NHYR01000001	NHYS01000001
NHYT01000001	NIFW01000002	NJEX02000002	NQCO02000001	NQCP02000001	NQCQ02000001
NQCR02000001	NQLI02000001	NWVR01000001	NXER01000001	OU342919	OU349837
OU349838	OU349839	OU349840	OU349841	OU349842	OU349843
OU349845	OU349846	OU349848	OU356913	PDED01000001	PDEK01000002
PDLD01000001	PDLF01000002	PDLJ01000002	PSQV01000007	PSQW01000002	PSQX01000006
PVMG01000001	QBDM01000001	QDAJ01000001	QLVH01000001	QSMQ02000006	QSMR02000002

Continued on next page

QSMV02000001	QWLO01000001	QXTI01000001	RKJE01000005	RKJJ01000004	RKJK01000003
RKJN01000003	RRCS01000001	RYCV01000003	SESS01000001	SEVB01000002	SEVD01000001
SEVE01000003	SEVI01000004	SEVL01000002	SEVM01000001	SEVN01000002	SEVP01000003
SEVQ01000001	SEVS01000006	SEVU01000007	SEVW01000003	SEVX01000001	SEVY01000001
SGYH01000001	SGYI01000001	SSWI01000001	UEFM01000001	UFYZ01000002	UFZD01000005
UFZE01000001	UFZF01000006	UFZG01000001	UFZI01000001	UFZT01000001	UFZV01000003
UGAD01000001	UGAE01000003	UGAH01000002	UGAL01000001	UGAM01000005	UGAO01000001
UGAP01000001	UGAS01000002	UGAT01000002	UGAY01000002	UGBA01000002	UGBB01000002
UGBE01000003	UGBF01000004	UGBK01000002	UGBL01000001	UGBM01000005	UGBP01000004
UGBQ01000003	UGBS01000001	UGBV01000003	UGBW01000003	UGBX01000001	UGCH01000001
UGCT01000004	UGCY01000004	UGDB01000003	UGDG01000002	UGDJ01000004	UGDK01000002
UGDO01000002	UGDQ01000001	UGDR01000001	UGDU01000003	UGDV01000004	UGDW01000001
UGDY01000003	UGEC01000002	UGEE01000003	UGEJ01000002	UGEL01000003	UGES01000005
UGEU01000002	UGEV01000002	UGEZ01000005	UGFA01000002	UGFB01000004	UGFO01000006
UGFP01000001	UGFR01000001	UGFS01000002	UGFT01000003	UGFV01000001	UGFZ01000001
UGGA01000003	UGGG01000003	UGGH01000001	UGGI01000001	UGGK01000002	UWVA01000001
UWVG01000001	UWVZ01000001	UWWG01000001	UWYG01000001	UYIJ01000003	VCAQ01000001
VCYN01000001	VCYX01000001	VCYY01000001	VCZJ01000001	VCZK01000001	VCZP01000001
VCZX01000001	VCZY01000001	VDBH01000001	VDBI01000001	VFAG01000002	WMHS01000001
WMHV01000001	WMLE01000001	WNKN01000001	WSNS01000001		

Table S6. GenBank accession numbers of sequences used for *E. coli* fumC analysis.

AAKB02000001	AP017610	AP017617	AP017620	AP018488	AP018784
AP018796	AP018802	AP018808	AP019189	AP019675	AP019761
AP019856	AP021890	AP021891	AP021892	AP021893	AP021894
AP021895	AP021896	AP021914	AP021933	AP021935	AP021944
AP021946	AP021963	AP021998	AP022003	AP022030	AP022032
AP022036	AP022044	AP022049	AP022064	AP022069	AP022087
AP022095	AP022098	AP022105	AP022107	AP022120	AP022159
AP022161	AP022171	AP022173	AP022177	AP022207	AP022215
AP022221	AP022222	AP022245	AP022261	AP022287	AP022295
AP022298	AP022326	AP022362	AP022409	AP022478	AP022482
AP022525	AP022533	AP022536	AP022540	AP022549	AP022650
AP022811	AP022815	AP023190	AP023197	AP023219	AP023226
AP023230	AP023286	AP023427	AP024112	AP024114	AP024123
AP024126	AP024130	AP024131	AP024205	AP024471	AP024473
AP024475	AP024478	AP024518	AP024521	AP024560	AP024561
AP024582	AP024687	AP024694	AP024921	AP024924	AP025205
AP025209	AP025214	AP025220	APIN01000002	AXLH01000003	AXLI01000001
BA000007	BNEX01000001	BNFC01000001	BNFH01000001	BNFI01000001	BNFJ01000001
BNFK01000001	BNFN01000001	BNFQ01000001	BNFS01000001	BNIP01000001	BNIQ01000001
BNIS01000001	BNIV01000001	BNIW01000001	BNIZ01000001	BNJA01000001	BNJB01000001
BNSX01000001	CABFNM010000001	CABMNJ010000001	CABWIT010000001	CABWIU010000001	CABWIX010000001
CABWJC010000001	CABWJF010000003	CABWJL010000001	CABWJM010000001	CABWJO010000005	CACRWM010000001
CACRWO010000001	CACRWV010000001	CADDWP010000001	CADDWR010000001	CADDWS010000001	CADDWX010000001
CADDWY010000002	CADDWZ010000001	CADDXF010000001	CADDXG010000001	CADDXI010000001	CADDXJ010000001
CADDXL010000002	CADDXM010000001	CADDXN010000001	CADDXO010000001	CADDXP010000001	CADDXQ010000002
CADDXS010000001	CADEAI010000002	CAJGEG010000001	CAJGEH010000001	CAJGEI010000001	CAJGWN010000001
CAJNTK010000001	CAJNVV010000001	CAJOCA010000001	CAJOCB010000001	CM000662	CP001509
CP002967	CP002970	CP003034	CP006027	CP006632	CP006636
CP006830	CP006834	CP007133	CP007265	CP007391	CP007394
CP007491	CP007799	CP008805	CP009072	CP009166	CP009273
CP009644	CP009685	CP009789	CP009859	CP010117	CP010119
CP010122	CP010125	CP010132	CP010133	CP010137	CP010145
CP010148	CP010150	CP010151	CP010160	CP010163	CP010167
CP010169	CP010170	CP010171	CP010172	CP010177	CP010180
CP010183	CP010186	CP010191	CP010200	CP010213	CP010219
CP010226	CP010228	CP010229	CP010230	CP010235	CP010237
CP010238	CP010240	CP010242	CP010344	CP010438	CP010441
CP010442	CP010443	CP010585	CP010876	CP011018	CP011124
CP011134	CP011320	CP011321	CP011322	CP011323	CP011324
CP011342	CP011343	CP011416	CP011495	CP012376	CP012379
CP012380	CP012693	CP012781	CP012802	CP013025	CP013048
CP013112	CP013483	CP013658	CP013831	CP013835	CP013952
CP014092	CP014111	CP014225	CP014269	CP014348	CP014488
CP014495	CP014497	CP014522	CP014583	CP015020	CP015023
CP015069	CP015074	CP015076	CP015138	CP015228	CP015240
CP015241	CP015832	CP015834	CP015842	CP015843	CP015846
CP015853	CP015855	CP015912	CP015995	CP016007	CP016182
CP016358	CP016497	CP016546	CP016625	CP016628	CP016755

Continued on next page

CP016828	CP017061	CP017220	CP017251	CP017438	CP017669
CP017844	CP017979	CP017980	CP018241	CP018245	CP018247
CP018252	CP018323	CP018801	CP018840	CP018948	CP018953
CP018957	CP018976	CP018979	CP018991	CP019000	CP019005
CP019012	CP019015	CP019029	CP019051	CP019213	CP019243
CP019245	CP019250	CP019256	CP019259	CP019267	CP019271
CP019273	CP019280	CP019355	CP019361	CP019392	CP019455
CP019558	CP019778	CP019903	CP019953	CP019961	CP020025
CP020048	CP020055	CP020058	CP020106	CP020368	CP020516
CP020545	CP020835	CP021175	CP021179	CP021193	CP021207
CP021288	CP021335	CP021339	CP021454	CP021532	CP021535
CP021689	CP021691	CP021732	CP021736	CP021840	CP021879
CP021935	CP022050	CP022086	CP022154	CP022229	CP022393
CP022407	CP022414	CP022466	CP022686	CP022689	CP022730
CP023165	CP023200	CP023201	CP023258	CP023346	CP023349
CP023357	CP023364	CP023371	CP023377	CP023383	CP023388
CP023531	CP023535	CP023541	CP023644	CP023815	CP023820
CP023826	CP023834	CP023844	CP023849	CP023853	CP023870
CP023899	CP023960	CP024056	CP024090	CP024127	CP024138
CP024141	CP024240	CP024243	CP024245	CP024256	CP024257
CP024260	CP024263	CP024269	CP024275	CP024278	CP024289
CP024293	CP024479	CP024618	CP024650	CP024659	CP024661
CP024667	CP024717	CP024720	CP024815	CP024821	CP024830
CP024851	CP024855	CP024859	CP024862	CP024886	CP024978
CP024992	CP024997	CP025036	CP025048	CP025251	CP025328
CP025401	CP025520	CP025573	CP025703	CP025707	CP025739
CP025753	CP025840	CP025842	CP025847	CP025849	CP025851
CP025856	CP025862	CP025865	CP025869	CP025881	CP025892
CP025903	CP025910	CP025916	CP025920	CP025950	CP026028
CP026199	CP026202	CP026357	CP026473	CP026491	CP026580
CP026723	CP026755	CP026853	CP026929	CP026932	CP026935
CP026939	CP027060	CP027105	CP027134	CP027140	CP027205
CP027255	CP027307	CP027312	CP027317	CP027319	CP027323
CP027325	CP027328	CP027335	CP027338	CP027342	CP027351
CP027352	CP027355	CP027362	CP027363	CP027366	CP027371
CP027373	CP027387	CP027388	CP027390	CP027394	CP027435
CP027437	CP027440	CP027442	CP027445	CP027447	CP027449
CP027452	CP027457	CP027459	CP027472	CP027520	CP027534
CP027544	CP027546	CP027548	CP027550	CP027552	CP027555
CP027572	CP027577	CP027582	CP027584	CP027586	CP027587
CP027591	CP027593	CP027597	CP027599	CP027701	CP027766
CP027851	CP028112	CP028116	CP028126	CP028166	CP028306
CP028308	CP028310	CP028320	CP028483	CP028578	CP028589
CP028590	CP028592	CP028600	CP028607	CP028650	CP028652
CP028656	CP028674	CP028677	CP028685	CP028693	CP028695
CP028703	CP029057	CP029108	CP029111	CP029122	CP029164
CP029180	CP029238	CP029239	CP029240	CP029242	CP029371
CP029420	CP029574	CP029579	CP029630	CP029687	CP029741
CP029981	CP030111	CP030240	CP030337	CP030767	CP030768
CP030919	CP030939	CP031105	CP031134	CP031157	CP031231
CP031256	CP031293	CP031321	CP031341	CP031343	CP031345
CP031347	CP031349	CP031353	CP031355	CP031546	CP031609
CP031653	CP031706	CP031902	CP031906	CP031910	CP031912
CP031916	CP031919	CP031922	CP032066	CP032073	CP032078
CP032085	CP032145	CP032201	CP032204	CP032265	CP032426
CP032515	CP032789	CP032795	CP032797	CP032801	CP032803
CP032808	CP032879	CP032892	CP032936	CP033092	CP033158
CP033378	CP033401	CP033635	CP033762	CP033850	CP033884
CP034384	CP034399	CP034404	CP034426	CP034658	CP034727
CP034734	CP034738	CP034745	CP034787	CP034792	CP034794
CP034797	CP034799	CP034803	CP034806	CP034808	CP034843
CP034953	CP034958	CP034966	CP035123	CP035312	CP035317
CP035318	CP035320	CP035321	CP035322	CP035325	CP035330
CP035331	CP035339	CP035350	CP035354	CP035360	CP035366
CP035377	CP035476	CP035477	CP035486	CP035498	CP035516
CP035545	CP035706	CP035720	CP035767	CP035768	CP035770
CP035772	CP035812	CP035817	CP035819	CP035822	CP035832
CP035836	CP035841	CP035846	CP035850	CP035860	CP035865
CP035870	CP035874	CP035879	CP035882	CP035884	CP035944
CP036177	CP036202	CP036245	CP037449	CP037857	CP037941
CP037943	CP037945	CP038180	CP038282	CP038284	CP038287
CP038290	CP038292	CP038295	CP038300	CP038302	CP038305
CP038309	CP038313	CP038316	CP038319	CP038321	CP038324

Continued on next page

CP038328	CP038333	CP038336	CP038339	CP038342	CP038344
CP038346	CP038349	CP038351	CP038353	CP038355	CP038357
CP038360	CP038363	CP038366	CP038369	CP038372	CP038374
CP038376	CP038380	CP038383	CP038386	CP038389	CP038394
CP038398	CP038402	CP038405	CP038408	CP038412	CP038414
CP038416	CP038419	CP038421	CP038423	CP038425	CP038428
CP038453	CP038494	CP038496	CP038505	CP038791	CP038857
CP038859	CP039298	CP039403	CP039404	CP039753	CP039834
CP039837	CP040067	CP040090	CP040107	CP040263	CP040269
CP040305	CP040307	CP040309	CP040311	CP040313	CP040314
CP040316	CP040381	CP040390	CP040397	CP040456	CP040570
CP040572	CP040643	CP040663	CP040664	CP040667	CP040886
CP040919	CP040927	CP041002	CP041031	CP041110	CP041284
CP041300	CP041302	CP041304	CP041337	CP041359	CP041392
CP041411	CP041416	CP041422	CP041425	CP041429	CP041431
CP041433	CP041435	CP041437	CP041442	CP041448	CP041452
CP041520	CP041522	CP041526	CP041529	CP041532	CP041535
CP041538	CP041541	CP041544	CP041546	CP041550	CP041556
CP041559	CP041563	CP041572	CP041578	CP041581	CP041623
CP041627	CP041628	CP041678	CP041747	CP041749	CP041919
CP041955	CP041996	CP042246	CP042250	CP042298	CP042336
CP042350	CP042470	CP042585	CP042588	CP042599	CP042645
CP042844	CP042846	CP042871	CP042878	CP042892	CP042893
CP042896	CP042899	CP042901	CP042903	CP042934	CP042969
CP042973	CP042981	CP042982	CP043033	CP043181	CP043183
CP043185	CP043187	CP043189	CP043191	CP043193	CP043195
CP043197	CP043199	CP043201	CP043203	CP043205	CP043207
CP043209	CP043211	CP043213	CP043217	CP043227	CP043406
CP043414	CP043478	CP043486	CP043487	CP043539	CP043541
CP043542	CP043733	CP043736	CP043739	CP043742	CP043744
CP043747	CP043750	CP043752	CP043754	CP043757	CP043852
CP043942	CP043946	CP043950	CP044021	CP044140	CP044143
CP044148	CP044291	CP044293	CP044298	CP044305	CP044311
CP044312	CP044313	CP044314	CP044315	CP044346	CP044403
CP044410	CP045190	CP045206	CP045209	CP045213	CP045277
CP045457	CP045712	CP045741	CP045827	CP045863	CP045975
CP045977	CP046000	CP046003	CP046006	CP046009	CP046025
CP046259	CP046396	CP046416	CP046527	CP046539	CP046716
CP047002	CP047010	CP047074	CP047077	CP047078	CP047079
CP047127	CP047231	CP047277	CP047378	CP047405	CP047455
CP047461	CP047571	CP047576	CP047594	CP047600	CP047609
CP047658	CP047662	CP047665	CP047710	CP047876	CP048010
CP048025	CP048107	CP048290	CP048310	CP048322	CP048326
CP048330	CP048337	CP048344	CP048359	CP048367	CP048371
CP048376	CP048439	CP048603	CP048604	CP048605	CP048606
CP048647	CP048821	CP048853	CP048855	CP048857	CP048860
CP048863	CP048865	CP048867	CP048870	CP048873	CP048915
CP048920	CP048934	CP048935	CP049050	CP049077	CP049081
CP049085	CP049086	CP049101	CP049118	CP049197	CP049198
CP049201	CP049204	CP049299	CP049348	CP049353	CP049839
CP049845	CP049852	CP049936	CP049943	CP049967	CP049979
CP050031	CP050036	CP050040	CP050044	CP050047	CP050173
CP050193	CP050194	CP050195	CP050197	CP050198	CP050199
CP050200	CP050201	CP050202	CP050203	CP050204	CP050205
CP050206	CP050208	CP050209	CP050210	CP050211	CP050212
CP050213	CP050214	CP050215	CP050216	CP050217	CP050218
CP050219	CP050220	CP050221	CP050222	CP050289	CP050382
CP050498	CP050646	CP050862	CP050865	CP050998	CP051001
CP051158	CP051219	CP051222	CP051609	CP051615	CP051631
CP051688	CP051692	CP051694	CP051698	CP051700	CP051706
CP051711	CP051714	CP051716	CP051719	CP051725	CP051727
CP051733	CP051735	CP051738	CP051744	CP051749	CP051751
CP051753	CP051765	CP051776	CP051778	CP051782	CP051786
CP051790	CP051792	CP051796	CP051798	CP051800	CP051802
CP051804	CP051806	CP051808	CP051811	CP051814	CP051816
CP051819	CP051821	CP051824	CP051827	CP051830	CP051833
CP051837	CP051849	CP052057	CP052877	CP053079	CP053080
CP053211	CP053231	CP053234	CP053245	CP053247	CP053251
CP053258	CP053259	CP053281	CP053284	CP053296	CP053384
CP053600	CP053601	CP053602	CP053603	CP053604	CP053605
CP053607	CP053720	CP053723	CP053730	CP053731	CP053736
CP053785	CP053787	CP053851	CP053852	CP054214	CP054219
CP054224	CP054227	CP054230	CP054232	CP054236	CP054282

Continued on next page

CP054335	CP054343	CP054345	CP054353	CP054363	CP054368
CP054371	CP054372	CP054379	CP054388	CP054407	CP054449
CP054454	CP054556	CP054828	CP054940	CP055251	CP055256
CP055259	CP055426	CP055438	CP055608	CP055624	CP055649
CP055652	CP055660	CP055669	CP055681	CP055713	CP055718
CP055724	CP055736	CP055748	CP055753	CP055786	CP055796
CP055818	CP055888	CP055918	CP055923	CP055934	CP055941
CP055945	CP055951	CP055958	CP055964	CP055967	CP055971
CP055973	CP055976	CP055981	CP056036	CP056073	CP056077
CP056190	CP056237	CP056242	CP056248	CP056249	CP056263
CP056273	CP056278	CP056282	CP056299	CP056304	CP056315
CP056348	CP056355	CP056357	CP056370	CP056392	CP056396
CP056404	CP056446	CP056470	CP056481	CP056551	CP056556
CP056564	CP056565	CP056569	CP056578	CP056584	CP056594
CP056598	CP056604	CP056608	CP056613	CP056618	CP056620
CP056625	CP056632	CP056642	CP056655	CP056662	CP056786
CP056794	CP056800	CP056810	CP056816	CP056821	CP056842
CP056846	CP056865	CP056867	CP056870	CP056873	CP056876
CP056894	CP056902	CP056914	CP056920	CP056921	CP056928
CP056933	CP056940	CP056947	CP056955	CP056974	CP056985
CP057007	CP057010	CP057012	CP057014	CP057016	CP057018
CP057020	CP057022	CP057024	CP057026	CP057028	CP057031
CP057038	CP057040	CP057045	CP057050	CP057055	CP057057
CP057062	CP057072	CP057075	CP057085	CP057087	CP057090
CP057109	CP057112	CP057115	CP057119	CP057123	CP057126
CP057129	CP057133	CP057137	CP057139	CP057141	CP057144
CP057152	CP057154	CP057160	CP057163	CP057166	CP057167
CP057171	CP057173	CP057175	CP057176	CP057178	CP057185
CP057186	CP057188	CP057190	CP057192	CP057194	CP057200
CP057204	CP057207	CP057223	CP057225	CP057234	CP057247
CP057250	CP057254	CP057259	CP057262	CP057267	CP057270
CP057273	CP057276	CP057281	CP057287	CP057293	CP057302
CP057334	CP057336	CP057340	CP057342	CP057344	CP057345
CP057347	CP057349	CP057354	CP057364	CP057368	CP057371
CP057378	CP057380	CP057396	CP057402	CP057408	CP057417
CP057420	CP057423	CP057429	CP057432	CP057435	CP057438
CP057441	CP057444	CP057447	CP057450	CP057453	CP057455
CP057465	CP057468	CP057470	CP057473	CP057475	CP057479
CP057484	CP057485	CP057487	CP057491	CP057493	CP057500
CP057507	CP057510	CP057512	CP057516	CP057520	CP057523
CP057525	CP057535	CP057539	CP057545	CP057555	CP057559
CP057570	CP057574	CP057577	CP057580	CP057582	CP057584
CP057587	CP057589	CP057594	CP057597	CP057600	CP057603
CP057605	CP057608	CP057612	CP057615	CP057617	CP057621
CP057626	CP057634	CP057636	CP057638	CP057640	CP057643
CP057645	CP057650	CP057660	CP057665	CP057669	CP057673
CP057677	CP057684	CP057686	CP057690	CP057693	CP057696
CP057705	CP057713	CP057717	CP057725	CP057729	CP057731
CP057738	CP057751	CP057759	CP057767	CP057773	CP057778
CP057784	CP057790	CP057796	CP057802	CP057804	CP057808
CP057812	CP057818	CP057826	CP057829	CP057831	CP057833
CP057838	CP057839	CP057841	CP057843	CP057849	CP057854
CP057860	CP057861	CP057869	CP057873	CP057876	CP057878
CP057879	CP057882	CP057885	CP057888	CP057891	CP057893
CP057900	CP057901	CP057905	CP057906	CP057911	CP057915
CP057916	CP057922	CP057927	CP057930	CP057932	CP057936
CP057941	CP057942	CP057946	CP057951	CP057953	CP057957
CP057959	CP057961	CP057962	CP057964	CP057967	CP057970
CP057973	CP057975	CP057978	CP057980	CP057983	CP057987
CP057990	CP057993	CP057994	CP057995	CP057996	CP058004
CP058007	CP058009	CP058012	CP058014	CP058019	CP058023
CP058025	CP058027	CP058031	CP058063	CP058070	CP058072
CP058074	CP058076	CP058078	CP058080	CP058083	CP058087
CP058090	CP058093	CP058101	CP058104	CP058223	CP058302
CP058308	CP058342	CP058355	CP058574	CP058596	CP058618
CP058651	CP058656	CP058661	CP058682	CP058913	CP058948
CP059003	CP059043	CP059119	CP059125	CP059130	CP059137
CP059279	CP059281	CP059283	CP059288	CP059723	CP059835
CP059904	CP059907	CP059913	CP059917	CP059922	CP059925
CP059929	CP059931	CP059944	CP059947	CP059953	CP059988
CP060057	CP060061	CP060065	CP060073	CP060075	CP060083
CP060121	CP060708	CP060709	CP061101	CP061108	CP061185
CP061206	CP061329	CP061337	CP061339	CP061530	CP061749

Continued on next page

CP061754	CP061758	CP061764	CP061914	CP062160	CP062203
CP062204	CP062211	CP062228	CP062700	CP062702	CP062705
CP062708	CP062711	CP062713	CP062715	CP062717	CP062719
CP062721	CP062723	CP062725	CP062727	CP062729	CP062731
CP062733	CP062736	CP062739	CP062742	CP062744	CP062746
CP062749	CP062752	CP062755	CP062758	CP062761	CP062763
CP062766	CP062769	CP062771	CP062774	CP062778	CP062780
CP062782	CP062811	CP062815	CP062824	CP062826	CP062832
CP062838	CP062844	CP062851	CP062853	CP062855	CP062863
CP062865	CP062868	CP062871	CP062876	CP062883	CP062889
CP062895	CP062901	CP062908	CP062910	CP062924	CP062927
CP062930	CP062933	CP062967	CP062970	CP063046	CP063153
CP063214	CP063332	CP063341	CP063349	CP063369	CP063459
CP063473	CP063476	CP063479	CP063483	CP063487	CP063492
CP063499	CP063503	CP063511	CP063515	CP063518	CP063711
CP063713	CP063720	CP063729	CP063736	CP063739	CP063742
CP063774	CP063956	CP063958	CP063969	CP063970	CP063979
CP063983	CP064103	CP064167	CP064677	CP064678	CP064679
CP064680	CP064681	CP064682	CP064683	CP064847	CP065136
CP065152	CP065607	CP065611	CP065613	CP065615	CP065619
CP065624	CP065970	CP066032	CP066144	CP066366	CP066748
CP066753	CP066756	CP066759	CP066806	CP066836	CP066844
CP067232	CP067235	CP067241	CP067245	CP067250	CP067265
CP067274	CP067299	CP067303	CP067307	CP067311	CP067342
CP067399	CP067994	CP068035	CP068041	CP068155	CP068279
CP068281	CP068283	CP068394	CP068591	CP068706	CP068795
CP068796	CP068799	CP068800	CP068801	CP068802	CP068803
CP068804	CP068805	CP068806	CP068807	CP068808	CP068809
CP068810	CP068811	CP068812	CP068813	CP068814	CP068815
CP068816	CP068817	CP068818	CP068819	CP068820	CP068821
CP068822	CP068823	CP068824	CP068825	CP068826	CP068827
CP068828	CP068829	CP068830	CP068995	CP069132	CP069133
CP069134	CP069386	CP069438	CP069441	CP069445	CP069447
CP069451	CP069453	CP069459	CP069480	CP069488	CP069500
CP069501	CP069517	CP069522	CP069528	CP069542	CP069549
CP069559	CP069571	CP069583	CP069589	CP069646	CP069657
CP069666	CP069677	CP069682	CP069692	CP069706	CP069707
CP069709	CP069710	CP069712	CP069865	CP069882	CP069890
CP069893	CP069935	CP069945	CP069957	CP069973	CP069978
CP069980	CP069990	CP069996	CP070007	CP070026	CP070041
CP070045	CP070048	CP070063	CP070066	CP070071	CP070077
CP070098	CP070103	CP070111	CP070115	CP070124	CP070134
CP070148	CP070152	CP070157	CP070162	CP070170	CP070227
CP070232	CP070279	CP070280	CP070393	CP070399	CP070403
CP070593	CP070902	CP070906	CP070914	CP070920	CP070930
CP070962	CP071036	CP071050	CP071073	CP071133	CP071375
CP071436	CP071439	CP071441	CP071521	CP071522	CP071954
CP072054	CP072204	CP072322	CP072539	CP072661	CP072662
CP072663	CP072784	CP072785	CP072786	CP072787	CP072802
CP072858	CP072911	CP072976	CP072980	CP073360	CP073589
CP073591	CP073593	CP073595	CP073597	CP073599	CP073601
CP073603	CP073607	CP073609	CP073613	CP073617	CP073619
CP073621	CP073624	CP073718	CP073719	CP073768	CP074120
CP074576	CP074701	CP075061	CP075062	CP075374	CP075477
CP075480	CP075484	CP075627	CP075632	CP075640	CP075649
CP075652	CP075663	CP075665	CP075697	CP075852	CP076123
CP076230	CP076232	CP076235	CP076237	CP076241	CP076243
CP076245	CP076293	CP076301	CP076305	CP076318	CP076344
CP076404	CP076527	CP076645	CP076646	CP076654	CP076687
CP076689	CP076693	CP076697	CP076704	CP076706	CP076709
CP076714	CP077215	CP077225	CP077243	CP077250	CP077266
CP077273	CP077281	CP077294	CP077314	CP077325	CP077342
CP077353	CP077361	CP077379	CP077389	CP077396	CP077646
CP077649	CP077969	CP078137	CP078555	CP078556	CP078558
CP078565	CP078568	CP078570	CP078571	CP078572	CP078576
CP078578	CP078585	CP078586	CP078588	CP078590	CP079735
CP079736	CP079747	CP080001	CP080116	CP080117	CP080118
CP080119	CP080120	CP080121	CP080126	CP080133	CP080139
CP080146	CP080155	CP080163	CP080172	CP080223	CP080233
CP080235	CP080246	CP080253	CP080255	CP080260	CP080370
CP080620	CP080645	CP081007	CP081008	CP081009	CP081010
CP081192	CP081306	CP081677	CP081684	CP081690	CP081698
CP081701	CP081706	CP081711	CP081713	CP081719	CP081724

Continued on next page

CP081729	CP081736	CP081742	CP081748	CP081755	CP081762
CP081767	CP081774	CP081782	CP081791	CP081796	CP081805
CP081881	CP082120	CP082127	CP082129	CP082330	CP082349
CP082355	CP082357	CP082824	CP082827	CP082831	CP082949
CP083263	CP083273	CP083410	CP083419	CP083478	CP083481
CP083492	CP083497	CP083503	CP083512	CP083517	CP083530
CP083534	CP083585	CP083701	CP083869	CP083872	CP083877
CP083880	CP083886	CP083888	CP084026	CP084096	CP084529
CP084534	CP084893	CP084894	CP084895	CP084896	CP084897
CP084898	CP084899	CP085056	CP085060	CP085063	CP085069
CP085074	CP085183	CP085188	CP085372	CP085488	CP085619
CP085622	CP085637	CP085638	CP085842	CP086259	CP086334
CP086336	CP086338	CP086340	CP086342	CP086391	CP086556
CP086616	CP086618	CP086620	CP086622	CP086623	CP086624
CP086625	CP086626	CP086627	CP086662	CP087110	CP087377
CP087570	CP087578	CP087958	CP088026	CP088028	CP088030
CP088032	CP088034	CP088036	CP088038	CP088040	CP088042
CP088044	CP088046	CP088048	CP088050	CP088052	CP088054
CP088056	CP088058	CP088060	CP088062	CP088064	CP088067
CP088069	CP088071	CP088344	CP088346	CP088348	CP088351
CP088352	CP088356	CP088363	CP088366	CP088369	CP088370
CP088374	CP088381	CP088383	CP088387	CP088390	CP088393
CP088397	CP088400	CP088406	CP088407	CP088408	CP088413
CP088414	CP088417	CP088421	CP088427	CP088433	CP088436
CP088443	CP088451	CP088452	CP088456	CP088461	CP088463
CP088466	CP088468	CP088473	CP088474	CP088479	CP088485
CP088486	CP088490	CP088494	CP088496	CP088500	CP088506
CP088509	CP088512	CP088514	CP088516	CP088520	CP088526
CP088529	CP088534	CP088542	CP088545	CP088547	CP088551
CP088553	CP088555	CP088560	CP088564	CP088566	CP088570
CP088576	CP088578	CP088580	CP088582	CP088584	CP088592
CP088593	CP088596	CP088598	CP088602	CP088607	CP088609
CP088612	CP088613	CP088617	CP088621	CP088624	CP088627
CP088629	CP088632	CP088637	CP088641	CP088643	CP088645
CP088649	CP088652	CP088654	CP088656	CP088658	CP088660
CP088662	CP088665	CP088673	CP088677	CP088681	CP088683
CP088687	CP088692	CP088694	CP088698	CP088701	CP088703
CP088707	CP088710	CP088714	CP088719	CP088722	CP088723
CP088725	CP088729	CP088734	CP088737	CP088740	CP088744
CP088754	CP088760	CP088764	CP088768	CP088771	CP088773
CP088776	CP088779	CP088782	CP088786	CP088790	CP088795
CP088800	CP088802	CP088804	CP088808	CP088812	CP088818
CP088820	CP088822	CP088826	CP088828	CP088829	CP088833
CP088835	CP088837	CP088839	CP088850	CP088851	CP088855
CP088860	CP088864	CP088866	CP088869	CP088872	CP089031
CP089032	CP089034	CP089036	CP089142	CP089270	CP089272
CP089445	FXBK01000002	GL878028	GL988050	HE964769	HF572917
HG428755	HG738867	JAAJTL010000001	JAAJTN010000001	JAAJTO010000001	JAAJTP010000001
JAAJTQ010000001	JAAJTR010000001	JAAJTT010000001	JAAJTU010000001	JAAJTV010000001	JAAKGF010000001
JAALAC010000002	JAANTD010000001	JAATHU010000001	JAATHV010000001	JAATHY010000001	JABWPS010000001
JABXOE010000001	JACDQE010000001	JACDQF010000001	JACGZL020000001	JACGZO020000001	JACGZP020000001
JACGZT020000001	JACIVJ010000001	JACIVK010000001	JACOJH010000001	JACOLT010000001	JACOLU010000001
JACRQJ010000001	JACSXT010000001	JACXXU010000001	JACXXV010000001	JACXYM010000001	JACYGB010000002
JACYGC010000008	JACYGD010000009	JACYGE010000009	JACYGN010000008	JADALR010000001	JADALV010000001
JADALW010000001	JADDLL010000001	JADDLM010000001	JADDLP010000001	JADDLQ010000001	JADDMK010000001
JADDML010000001	JAEFCJ010000001	JAEFCL010000001	JAEFCM010000001	JAEFCO010000001	JAEFCP010000001
JAEPRM010000001	JAFBIE010000001	JAFCXR010000001	JAFFIC010000001	JAGEVC010000001	JAGFYA010000002
JAGFYB010000001	JAGFYC010000001	JAGFYF010000001	JAGFZN010000001	JAGFZR010000001	JAGFZU010000001
JAGHKJ010000001	JAHMAB010000001	JAHQKD010000001	JAHQKR010000001	JAHQKS010000001	JAHQLA010000001
JAHQLB010000001	JAHSRN010000001	JAHSRO010000001	JAHSRP010000001	JAHSRQ010000001	JAHSRR010000001
JAHSRS010000001	JAHSRT010000001	JAHSRU010000001	JAHSRV010000001	JAHSRW010000001	JAHSRX010000001
JAHSRY010000001	JAHSRZ010000001	JAHSSA010000001	JAHSSC010000001	JAHSSD010000001	JAHSSE010000001
JAINST010000001	JAINTH010000001	JAIQZL010000008	JAJJVS010000001	JAJJWF010000001	JAPJ02000001
JASQ01000001	JASU01000001	JH648165	JJMY02000001	JMJD01000003	KB732454
KB732522	KB732530	KB732580	KB732702	KB732739	KB732931
KB732993	KB733023	KB733071	KB733078	KB733130	KB733135
KB733141	KB733164	KB733172	KB733185	KB733235	KB733361
KE137004	KE137028	KE137211	KE700478	KI929682	KI929714
KI929774	KI929782	KI929787	KI929804	KK583188	KQ087662
KQ087687	KQ087890	LAZO01000001	LM993812	LM995446	LN832404
LN874954	LN877770	LORP02000005	LORS02000001	LPWC02000002	LQSL02000003
LQTX02000001	LQUJ02000001	LQVG02000001	LR025096	LR025101	LR130532
LR130545	LR130562	LR134079	LR134082	LR134092	LR134152

Continued on next page

LR134222	LR134226	LR134227	LR134234	LR134237	LR134240
LR217818	LR536430	LR536431	LR595691	LR607328	LR607331
LR740758	LR740776	LR778141	LR778142	LR778143	LR778145
LR778146	LR778147	LR778148	LR778149	LR778150	LR778151
LR881940	LR882050	LR882052	LR882057	LR882493	LR882973
LR882978	LR882990	LR882997	LR883006	LR883012	LR883050
LR883965	LR890270	LR890299	LR890326	LR890334	LR890349
LR890405	LR890410	LR890466	LR890508	LR890603	LR890606
LR890651	LR890693	LR890714	LR898868	LR898874	LRDF01000001
LS992166	LS992168	LS992171	LS992180	LS992185	LS992190
LS992192	LS998785	LS999560	LT594504	LT601384	LT615374
LT615379	LT827011	LT838200	LT883142	LT903847	LUYD01000001
LVRH02000001	LVRJ02000001	LVSP02000001	LVTB02000001	LWDC01000001	MBNU01000003
MBNW01000001	MBNX01000003	MBNY01000001	MCRE01000001	MDLJ02000001	MIIL01000001
MPGR01000001	MPGS01000001	MPGT01000001	MRDN01000001	NBIY01000001	NC_002655
NC_002695	NC_007779	NC_008253	NC_008563	NC_009800	NC_010473
NC_011415	NC_011742	NC_011750	NC_011751	NC_012759	NC_013353
NC_013941	NC_017628	NC_017632	NC_017641	NC_017646	NC_017651
NC_017652	NC_017656	NC_017660	NC_017664	NC_018650	NC_018661
NC_022364	NC_022370	NGRV02000001	NGSF01000001	NHYO01000001	NHYP01000001
NHYQ01000001	NHYR01000001	NHYS01000001	NHYT01000001	NIFW01000002	NJEX02000002
NJIR01000001	NQCO02000001	NQCP02000001	NQCQ02000001	NQCR02000001	NQLI02000001
NWVR01000001	NXER01000001	OU342919	OU349837	OU349838	OU349839
OU349840	OU349841	OU349842	OU349843	OU349844	OU349845
OU349846	OU349847	OU349849	OU356913	PDDT01000002	PDED01000001
PDEK01000002	PDLD01000001	PDLF01000002	PDLJ01000002	PSQV01000007	PSQW01000002
PVMG01000001	QBDM01000001	QDAJ01000001	QLVH01000001	QMGM01000001	QSMV02000001
QWLO01000001	QXTI01000001	RKJE01000005	RKJJ01000004	RKJK01000003	RKJN01000003
RRCS01000001	RYCV01000003	SESS01000001	SEVA01000001	SEVB01000002	SEVD01000001
SEVE01000003	SEVF01000002	SEVG01000002	SEVI01000004	SEVL01000002	SEVM01000001
SEVN01000002	SEVO01000002	SEVP01000003	SEVQ01000001	SEVT01000005	SEVW01000003
SEVX01000001	SEVY01000001	SEWA01000004	SGYH01000001	SGYI01000001	SSWI01000001
SSWJ01000001	SSWK01000001	UEFM01000001	UEFR01000001	UFYL01000003	UFYZ01000002
UFZE01000001	UFZF01000006	UFZG01000001	UFZI01000001	UGAD01000001	UGAE01000003
UGAH01000002	UGAK01000003	UGAM01000005	UGAO01000001	UGAS01000002	UGAU01000003
UGAV01000006	UGBA01000002	UGBB01000002	UGBE01000003	UGBM01000005	UGBP01000004
UGBV01000003	UGBW01000003	UGBX01000001	UGCH01000001	UGCI01000003	UGCT01000004
UGCY01000004	UGDB01000003	UGDG01000002	UGDH01000001	UGDJ01000004	UGDO01000002
UGDR01000001	UGDV01000004	UGDW01000001	UGDY01000003	UGEC01000002	UGEF01000002
UGEL01000003	UGEO01000001	UGES01000005	UGEU01000002	UGEV01000002	UGEY01000001
UGEZ01000005	UGFA01000002	UGFD01000001	UGFL01000003	UGFO01000006	UGFT01000003
UGFU01000001	UGFV01000001	UGGA01000003	UGGI01000001	UGGK01000002	UWUZ01000001
UWVZ01000001	UWWG01000001	UWXK01000001	UWYG01000001	VCAQ01000001	VCYN01000001
VCYV01000001	VCYX01000001	VCZA01000001	VCZH01000001	VCZP01000001	VCZT01000001
VCZV01000001	VCZY01000001	VDBC01000001	VDBH01000001	VFAG01000002	VMRI01000001
WIPD02000001	WMHS01000001	WMLE01000001	WNKN01000001	WNKO01000001	WSNS01000001
WXYW01000001					

Table S7. GenBank accession numbers of sequences used for *E. coli* recA analysis.

AAKB02000001	ABHK02000001	AELA02000001	AP017610	AP017617	AP017620
AP018488	AP018784	AP018796	AP018802	AP018808	AP019189
AP019675	AP019761	AP019856	AP021890	AP021891	AP021892
AP021893	AP021894	AP021895	AP021896	AP021914	AP021933
AP021935	AP021944	AP021946	AP021963	AP021998	AP022003
AP022030	AP022032	AP022036	AP022044	AP022049	AP022064
AP022069	AP022087	AP022095	AP022098	AP022105	AP022107
AP022120	AP022159	AP022161	AP022171	AP022173	AP022177
AP022207	AP022215	AP022221	AP022222	AP022245	AP022261
AP022287	AP022295	AP022326	AP022351	AP022362	AP022409
AP022478	AP022482	AP022525	AP022533	AP022536	AP022540
AP022549	AP022650	AP022811	AP022815	AP023190	AP023197
AP023219	AP023230	AP023235	AP023286	AP023427	AP024112
AP024114	AP024123	AP024126	AP024130	AP024205	AP024471
AP024473	AP024475	AP024478	AP024518	AP024521	AP024560
AP024582	AP024687	AP024694	AP024921	AP024924	AP025205
AP025209	AP025214	AP025220	APIN01000002	BA000007	BNEX01000001
BNFH01000001	BNFI01000001	BNFJ01000001	BNFK01000001	BNFN01000001	BNIP01000001
BNIQ01000001	BNIS01000001	BNIT01000001	BNIV01000001	BNIZ01000001	BNJA01000001
BNJB01000001	BNSX01000001	CABFNM010000001	CABMNJ010000001	CABWHO010000003	CABWIU010000001
CABWIW010000002	CABWIX010000001	CABWJC010000001	CABWJF010000003	CABWJL010000001	CABWJO010000005

Continued on next page

CACRWJ010000001	CACRWL010000001	CACRWM010000001	CACRWO010000001	CACRWV010000001	CADDWP010000001
CADDWR010000001	CADDWS010000001	CADDWT010000001	CADDWX010000001	CADDWY010000002	CADDWZ010000001
CADDXF010000001	CADDXH010000001	CADDXI010000001	CADDXJ010000001	CADDXL010000002	CADDXM010000001
CADDXN010000001	CADDXO010000001	CADDXP010000001	CADDXQ010000002	CADDXS010000001	CADDXU010000001
CADDXV010000001	CADEAI010000002	CAJGEG010000001	CAJGEH010000001	CAJGWN010000001	CAJMWD010000001
CAJNTK010000001	CAJNUE010000001	CAJNVV010000001	CAJOCB010000001	CAJSIA010000001	CM000662
CM000960	CP001509	CP002967	CP002970	CP003034	CP005930
CP006632	CP006636	CP006698	CP006830	CP006834	CP007133
CP007136	CP007265	CP007390	CP007393	CP007394	CP007491
CP007592	CP007799	CP008805	CP008957	CP009050	CP009072
CP009106	CP009166	CP009273	CP009578	CP009644	CP009685
CP009789	CP009859	CP010116	CP010117	CP010119	CP010122
CP010125	CP010132	CP010134	CP010137	CP010140	CP010145
CP010148	CP010151	CP010152	CP010160	CP010163	CP010167
CP010169	CP010170	CP010171	CP010172	CP010176	CP010177
CP010186	CP010196	CP010206	CP010219	CP010221	CP010226
CP010228	CP010230	CP010235	CP010236	CP010237	CP010238
CP010242	CP010344	CP010371	CP010816	CP010876	CP011018
CP011061	CP011113	CP011124	CP011320	CP011321	CP011322
CP011323	CP011324	CP011331	CP011342	CP011343	CP011495
CP011915	CP012376	CP012379	CP012380	CP012781	CP012802
CP012870	CP013025	CP013031	CP013048	CP013112	CP013658
CP013662	CP013663	CP013835	CP014092	CP014111	CP014270
CP014272	CP014314	CP014316	CP014348	CP014488	CP014495
CP014522	CP014667	CP015020	CP015023	CP015069	CP015074
CP015076	CP015159	CP015228	CP015229	CP015240	CP015241
CP015244	CP015834	CP015842	CP015846	CP015853	CP015855
CP015912	CP016182	CP016358	CP016404	CP016497	CP016546
CP016625	CP017061	CP017100	CP017220	CP017438	CP017669
CP017844	CP017979	CP017980	CP018103	CP018109	CP018115
CP018121	CP018206	CP018239	CP018241	CP018243	CP018245
CP018250	CP018252	CP018323	CP018770	CP018801	CP018953
CP018957	CP018970	CP018976	CP018991	CP019000	CP019005
CP019008	CP019012	CP019015	CP019029	CP019051	CP019213
CP019243	CP019245	CP019250	CP019259	CP019273	CP019280
CP019355	CP019359	CP019361	CP019392	CP019455	CP019558
CP019560	CP019629	CP019777	CP019778	CP019903	CP019953
CP019961	CP020025	CP020048	CP020055	CP020058	CP020092
CP020106	CP020116	CP020509	CP020545	CP020835	CP020933
CP021175	CP021179	CP021202	CP021207	CP021288	CP021339
CP021532	CP021535	CP021689	CP021722	CP021726	CP021732
CP021736	CP021840	CP021935	CP022086	CP022229	CP022393
CP022407	CP022466	CP022689	CP022730	CP022912	CP023142
CP023165	CP023346	CP023353	CP023357	CP023364	CP023366
CP023383	CP023531	CP023535	CP023541	CP023644	CP023673
CP023749	CP023815	CP023820	CP023826	CP023834	CP023844
CP023849	CP023853	CP023960	CP024056	CP024127	CP024141
CP024147	CP024228	CP024232	CP024240	CP024243	CP024245
CP024256	CP024257	CP024260	CP024263	CP024278	CP024289
CP024299	CP024479	CP024618	CP024650	CP024659	CP024661
CP024667	CP024717	CP024720	CP024815	CP024821	CP024826
CP024830	CP024851	CP024855	CP024859	CP024862	CP024886
CP024889	CP024978	CP024997	CP025251	CP025401	CP025520
CP025573	CP025707	CP025716	CP025739	CP025753	CP025840
CP025847	CP025849	CP025851	CP025856	CP025859	CP025862
CP025865	CP025881	CP025892	CP025903	CP025910	CP025913
CP025920	CP025950	CP026027	CP026028	CP026199	CP026355
CP026357	CP026399	CP026491	CP026580	CP026723	CP026853
CP026929	CP026932	CP026935	CP026939	CP027060	CP027103
CP027105	CP027126	CP027134	CP027140	CP027221	CP027255
CP027307	CP027310	CP027312	CP027317	CP027323	CP027325
CP027328	CP027331	CP027335	CP027338	CP027342	CP027347
CP027351	CP027355	CP027361	CP027362	CP027363	CP027366
CP027368	CP027373	CP027388	CP027390	CP027394	CP027430
CP027435	CP027437	CP027440	CP027442	CP027445	CP027447
CP027452	CP027454	CP027457	CP027459	CP027461	CP027472
CP027520	CP027534	CP027546	CP027548	CP027552	CP027555
CP027572	CP027573	CP027577	CP027579	CP027582	CP027584
CP027587	CP027593	CP027597	CP027599	CP027701	CP027766
CP027851	CP028112	CP028116	CP028117	CP028122	CP028126
CP028166	CP028192	CP028304	CP028306	CP028307	CP028309
CP028310	CP028379	CP028381	CP028429	CP028483	CP028578
CP028589	CP028600	CP028607	CP028695	CP028698	CP028702

Continued on next page

CP029057	CP029108	CP029115	CP029122	CP029164	CP029180
CP029239	CP029240	CP029242	CP029369	CP029420	CP029574
CP029576	CP029579	CP029630	CP029692	CP029973	CP029981
CP030111	CP030240	CP030331	CP030337	CP030767	CP030768
CP030919	CP031134	CP031215	CP031231	CP031256	CP031293
CP031321	CP031341	CP031343	CP031345	CP031347	CP031349
CP031353	CP031355	CP031546	CP031609	CP031706	CP031902
CP031910	CP031912	CP031916	CP032066	CP032073	CP032078
CP032085	CP032145	CP032201	CP032204	CP032261	CP032265
CP032426	CP032811	CP032879	CP032892	CP032936	CP032992
CP033096	CP033401	CP033605	CP033762	CP033850	CP033884
CP034106	CP034384	CP034404	CP034426	CP034595	CP034658
CP034727	CP034734	CP034738	CP034745	CP034787	CP034792
CP034794	CP034797	CP034799	CP034801	CP034803	CP034806
CP034808	CP034843	CP034953	CP034958	CP034966	CP035123
CP035312	CP035317	CP035318	CP035320	CP035321	CP035322
CP035325	CP035330	CP035331	CP035339	CP035350	CP035354
CP035360	CP035366	CP035377	CP035476	CP035477	CP035486
CP035498	CP035516	CP035545	CP035706	CP035720	CP035751
CP035767	CP035768	CP035770	CP035772	CP035812	CP035817
CP035819	CP035822	CP035832	CP035836	CP035841	CP035846
CP035850	CP035855	CP035860	CP035865	CP035870	CP035874
CP035879	CP035882	CP035884	CP035944	CP036177	CP036202
CP036245	CP037449	CP037857	CP037941	CP037943	CP037945
CP038180	CP038282	CP038284	CP038287	CP038290	CP038292
CP038295	CP038300	CP038302	CP038305	CP038309	CP038313
CP038316	CP038319	CP038321	CP038324	CP038328	CP038333
CP038336	CP038339	CP038342	CP038344	CP038346	CP038349
CP038351	CP038353	CP038355	CP038357	CP038360	CP038363
CP038366	CP038369	CP038372	CP038374	CP038376	CP038380
CP038383	CP038386	CP038389	CP038394	CP038398	CP038402
CP038405	CP038408	CP038412	CP038414	CP038416	CP038419
CP038421	CP038423	CP038425	CP038428	CP038453	CP038494
CP038496	CP038505	CP038791	CP038857	CP038859	CP039298
CP039403	CP039404	CP039753	CP039834	CP039837	CP040067
CP040090	CP040107	CP040263	CP040269	CP040305	CP040307
CP040309	CP040311	CP040313	CP040314	CP040316	CP040381
CP040390	CP040397	CP040570	CP040572	CP040643	CP040663
CP040664	CP040667	CP040886	CP040919	CP040927	CP041002
CP041031	CP041110	CP041284	CP041300	CP041302	CP041304
CP041337	CP041359	CP041392	CP041411	CP041416	CP041422
CP041425	CP041429	CP041431	CP041433	CP041435	CP041437
CP041442	CP041448	CP041452	CP041520	CP041522	CP041526
CP041529	CP041532	CP041535	CP041538	CP041541	CP041544
CP041546	CP041550	CP041556	CP041559	CP041563	CP041572
CP041578	CP041581	CP041623	CP041627	CP041628	CP041678
CP041747	CP041749	CP041919	CP041955	CP041996	CP042246
CP042250	CP042298	CP042336	CP042350	CP042585	CP042588
CP042599	CP042615	CP042627	CP042638	CP042645	CP042843
CP042845	CP042846	CP042867	CP042871	CP042878	CP042892
CP042893	CP042896	CP042899	CP042901	CP042903	CP042934
CP042969	CP042973	CP042981	CP042982	CP043033	CP043181
CP043183	CP043185	CP043187	CP043189	CP043191	CP043193
CP043195	CP043197	CP043199	CP043201	CP043203	CP043205
CP043207	CP043209	CP043211	CP043213	CP043217	CP043227
CP043406	CP043414	CP043478	CP043486	CP043487	CP043539
CP043541	CP043542	CP043733	CP043736	CP043739	CP043742
CP043744	CP043747	CP043750	CP043752	CP043754	CP043757
CP043852	CP043942	CP043946	CP043950	CP044021	CP044143
CP044145	CP044148	CP044291	CP044293	CP044298	CP044305
CP044311	CP044312	CP044313	CP044314	CP044315	CP044346
CP044403	CP044410	CP045190	CP045206	CP045209	CP045213
CP045457	CP045712	CP045741	CP045827	CP045863	CP045975
CP045977	CP046000	CP046003	CP046006	CP046009	CP046025
CP046259	CP046396	CP046416	CP046527	CP046539	CP046676
CP046716	CP047002	CP047010	CP047074	CP047077	CP047078
CP047079	CP047127	CP047231	CP047277	CP047378	CP047405
CP047455	CP047461	CP047571	CP047576	CP047594	CP047600
CP047609	CP047658	CP047662	CP047665	CP047710	CP047876
CP048010	CP048025	CP048107	CP048290	CP048304	CP048310
CP048322	CP048326	CP048330	CP048337	CP048344	CP048359
CP048367	CP048371	CP048376	CP048439	CP048603	CP048604
CP048605	CP048606	CP048647	CP048821	CP048853	CP048855

Continued on next page

CP048857	CP048860	CP048863	CP048865	CP048867	CP048870
CP048873	CP048915	CP048920	CP048934	CP048935	CP049050
CP049077	CP049081	CP049085	CP049086	CP049101	CP049118
CP049196	CP049197	CP049201	CP049204	CP049299	CP049348
CP049353	CP049839	CP049845	CP049852	CP049936	CP049943
CP049967	CP049979	CP050031	CP050036	CP050040	CP050044
CP050047	CP050173	CP050193	CP050194	CP050195	CP050196
CP050197	CP050199	CP050200	CP050201	CP050202	CP050203
CP050204	CP050205	CP050206	CP050207	CP050208	CP050209
CP050210	CP050211	CP050212	CP050213	CP050214	CP050215
CP050216	CP050217	CP050218	CP050219	CP050220	CP050221
CP050222	CP050289	CP050382	CP050498	CP050646	CP050862
CP050865	CP050998	CP051001	CP051158	CP051219	CP051222
CP051263	CP051609	CP051615	CP051631	CP051688	CP051692
CP051694	CP051698	CP051700	CP051706	CP051711	CP051714
CP051716	CP051719	CP051725	CP051727	CP051733	CP051735
CP051738	CP051744	CP051749	CP051751	CP051753	CP051765
CP051776	CP051778	CP051782	CP051786	CP051790	CP051792
CP051796	CP051798	CP051800	CP051802	CP051804	CP051806
CP051808	CP051811	CP051814	CP051816	CP051819	CP051821
CP051824	CP051827	CP051830	CP051833	CP051837	CP051849
CP052057	CP052877	CP053079	CP053080	CP053211	CP053231
CP053234	CP053245	CP053247	CP053251	CP053258	CP053259
CP053281	CP053284	CP053296	CP053384	CP053600	CP053601
CP053602	CP053603	CP053604	CP053605	CP053607	CP053720
CP053723	CP053730	CP053731	CP053736	CP053785	CP053787
CP053851	CP053852	CP054214	CP054219	CP054224	CP054227
CP054230	CP054232	CP054236	CP054282	CP054314	CP054317
CP054319	CP054328	CP054343	CP054345	CP054353	CP054363
CP054368	CP054371	CP054372	CP054379	CP054388	CP054407
CP054449	CP054454	CP054556	CP054828	CP054940	CP055158
CP055251	CP055256	CP055259	CP055426	CP055438	CP055608
CP055624	CP055649	CP055652	CP055660	CP055669	CP055681
CP055713	CP055718	CP055724	CP055736	CP055748	CP055753
CP055786	CP055796	CP055818	CP055888	CP055918	CP055923
CP055934	CP055941	CP055945	CP055951	CP055958	CP055964
CP055967	CP055971	CP055973	CP055976	CP055981	CP056036
CP056073	CP056077	CP056190	CP056237	CP056242	CP056248
CP056249	CP056263	CP056273	CP056278	CP056282	CP056299
CP056304	CP056315	CP056348	CP056355	CP056357	CP056370
CP056392	CP056396	CP056404	CP056446	CP056470	CP056481
CP056551	CP056556	CP056564	CP056565	CP056569	CP056578
CP056584	CP056594	CP056598	CP056604	CP056608	CP056613
CP056618	CP056620	CP056625	CP056632	CP056642	CP056655
CP056662	CP056786	CP056794	CP056800	CP056810	CP056816
CP056821	CP056842	CP056846	CP056865	CP056867	CP056870
CP056873	CP056876	CP056894	CP056902	CP056914	CP056920
CP056921	CP056928	CP056933	CP056940	CP056947	CP056955
CP056974	CP056985	CP057007	CP057010	CP057012	CP057014
CP057016	CP057018	CP057020	CP057022	CP057024	CP057026
CP057028	CP057031	CP057038	CP057040	CP057045	CP057050
CP057055	CP057057	CP057062	CP057072	CP057075	CP057085
CP057087	CP057090	CP057109	CP057112	CP057115	CP057119
CP057123	CP057126	CP057129	CP057133	CP057137	CP057139
CP057141	CP057144	CP057152	CP057154	CP057160	CP057163
CP057166	CP057167	CP057171	CP057173	CP057175	CP057176
CP057178	CP057185	CP057186	CP057188	CP057190	CP057192
CP057194	CP057200	CP057204	CP057207	CP057223	CP057225
CP057234	CP057247	CP057250	CP057254	CP057259	CP057262
CP057267	CP057270	CP057273	CP057276	CP057281	CP057287
CP057293	CP057302	CP057334	CP057336	CP057340	CP057342
CP057344	CP057345	CP057347	CP057349	CP057354	CP057364
CP057368	CP057371	CP057378	CP057380	CP057396	CP057402
CP057408	CP057417	CP057420	CP057423	CP057429	CP057432
CP057435	CP057438	CP057441	CP057444	CP057447	CP057450
CP057453	CP057455	CP057465	CP057468	CP057470	CP057473
CP057475	CP057479	CP057484	CP057485	CP057487	CP057491
CP057493	CP057500	CP057507	CP057510	CP057512	CP057516
CP057520	CP057523	CP057525	CP057535	CP057539	CP057545
CP057555	CP057559	CP057570	CP057574	CP057577	CP057580
CP057582	CP057584	CP057587	CP057589	CP057594	CP057597
CP057600	CP057603	CP057605	CP057608	CP057612	CP057615
CP057617	CP057621	CP057626	CP057634	CP057636	CP057638

Continued on next page

CP057640	CP057643	CP057645	CP057650	CP057660	CP057665
CP057669	CP057673	CP057677	CP057684	CP057686	CP057690
CP057693	CP057696	CP057705	CP057713	CP057717	CP057725
CP057729	CP057731	CP057738	CP057751	CP057759	CP057767
CP057773	CP057778	CP057784	CP057790	CP057796	CP057802
CP057804	CP057808	CP057812	CP057818	CP057826	CP057829
CP057831	CP057833	CP057838	CP057839	CP057841	CP057843
CP057849	CP057854	CP057860	CP057861	CP057869	CP057873
CP057876	CP057878	CP057879	CP057882	CP057885	CP057888
CP057891	CP057893	CP057900	CP057901	CP057905	CP057906
CP057911	CP057915	CP057916	CP057922	CP057927	CP057930
CP057932	CP057936	CP057941	CP057942	CP057946	CP057951
CP057953	CP057957	CP057959	CP057961	CP057962	CP057964
CP057967	CP057970	CP057973	CP057975	CP057978	CP057980
CP057983	CP057987	CP057990	CP057993	CP057994	CP057995
CP057996	CP058004	CP058007	CP058009	CP058012	CP058014
CP058019	CP058023	CP058025	CP058027	CP058031	CP058063
CP058070	CP058072	CP058074	CP058076	CP058078	CP058080
CP058083	CP058087	CP058090	CP058093	CP058101	CP058104
CP058223	CP058302	CP058308	CP058342	CP058355	CP058571
CP058574	CP058596	CP058618	CP058651	CP058656	CP058661
CP058682	CP058913	CP058948	CP059003	CP059043	CP059119
CP059125	CP059130	CP059137	CP059279	CP059283	CP059288
CP059723	CP059835	CP059904	CP059907	CP059913	CP059917
CP059925	CP059929	CP059931	CP059944	CP059947	CP059953
CP059988	CP060057	CP060061	CP060065	CP060073	CP060075
CP060083	CP060121	CP060709	CP061101	CP061108	CP061185
CP061206	CP061329	CP061337	CP061339	CP061530	CP061749
CP061754	CP061758	CP061764	CP061914	CP062160	CP062203
CP062204	CP062211	CP062228	CP062249	CP062700	CP062702
CP062705	CP062708	CP062711	CP062713	CP062715	CP062717
CP062719	CP062721	CP062723	CP062725	CP062727	CP062729
CP062731	CP062733	CP062736	CP062739	CP062742	CP062744
CP062746	CP062749	CP062752	CP062755	CP062758	CP062761
CP062763	CP062766	CP062769	CP062771	CP062774	CP062778
CP062780	CP062782	CP062811	CP062815	CP062824	CP062826
CP062832	CP062838	CP062844	CP062851	CP062853	CP062855
CP062863	CP062865	CP062868	CP062871	CP062876	CP062883
CP062889	CP062895	CP062901	CP062908	CP062910	CP062924
CP062927	CP062930	CP062933	CP062967	CP062970	CP062985
CP063046	CP063153	CP063214	CP063332	CP063341	CP063349
CP063369	CP063459	CP063473	CP063476	CP063479	CP063483
CP063487	CP063492	CP063499	CP063503	CP063511	CP063515
CP063518	CP063711	CP063713	CP063720	CP063729	CP063736
CP063739	CP063742	CP063774	CP063956	CP063958	CP063969
CP063970	CP063979	CP063983	CP064103	CP064167	CP064677
CP064678	CP064679	CP064680	CP064681	CP064682	CP064683
CP064847	CP065136	CP065152	CP065607	CP065611	CP065613
CP065615	CP065619	CP065624	CP065970	CP066032	CP066144
CP066366	CP066748	CP066753	CP066756	CP066759	CP066806
CP066836	CP066844	CP067232	CP067235	CP067241	CP067245
CP067250	CP067265	CP067274	CP067299	CP067303	CP067307
CP067311	CP067342	CP067399	CP067994	CP068035	CP068041
CP068155	CP068279	CP068281	CP068283	CP068394	CP068591
CP068706	CP068795	CP068796	CP068799	CP068800	CP068801
CP068802	CP068803	CP068804	CP068805	CP068806	CP068807
CP068808	CP068809	CP068810	CP068811	CP068812	CP068813
CP068814	CP068815	CP068816	CP068817	CP068818	CP068819
CP068820	CP068821	CP068822	CP068823	CP068824	CP068825
CP068826	CP068827	CP068828	CP068829	CP068830	CP068995
CP069132	CP069133	CP069134	CP069386	CP069438	CP069441
CP069445	CP069447	CP069451	CP069453	CP069459	CP069480
CP069488	CP069500	CP069501	CP069517	CP069522	CP069528
CP069542	CP069549	CP069559	CP069571	CP069583	CP069589
CP069646	CP069657	CP069666	CP069677	CP069682	CP069692
CP069706	CP069707	CP069709	CP069710	CP069712	CP069865
CP069882	CP069890	CP069893	CP069935	CP069945	CP069957
CP069973	CP069978	CP069980	CP069990	CP069996	CP070007
CP070026	CP070041	CP070045	CP070048	CP070063	CP070066
CP070071	CP070077	CP070098	CP070103	CP070111	CP070115
CP070124	CP070134	CP070148	CP070152	CP070157	CP070162
CP070170	CP070227	CP070232	CP070279	CP070280	CP070393
CP070399	CP070403	CP070593	CP070902	CP070906	CP070914

Continued on next page

CP070920	CP070930	CP070962	CP071036	CP071050	CP071073
CP071133	CP071375	CP071436	CP071439	CP071441	CP071521
CP071522	CP071954	CP072054	CP072204	CP072322	CP072539
CP072661	CP072662	CP072663	CP072784	CP072785	CP072786
CP072787	CP072802	CP072858	CP072911	CP072942	CP072976
CP072980	CP073360	CP073589	CP073591	CP073593	CP073595
CP073597	CP073599	CP073601	CP073603	CP073607	CP073609
CP073613	CP073617	CP073619	CP073621	CP073624	CP073718
CP073719	CP073768	CP074120	CP074576	CP074701	CP075061
CP075062	CP075374	CP075627	CP075632	CP075640	CP075649
CP075652	CP075663	CP075665	CP075697	CP075852	CP076123
CP076230	CP076232	CP076235	CP076237	CP076241	CP076243
CP076245	CP076293	CP076301	CP076305	CP076318	CP076344
CP076404	CP076527	CP076645	CP076646	CP076654	CP076687
CP076689	CP076693	CP076697	CP076704	CP076706	CP076709
CP076714	CP077063	CP077215	CP077225	CP077243	CP077250
CP077266	CP077273	CP077281	CP077294	CP077314	CP077325
CP077342	CP077353	CP077361	CP077379	CP077389	CP077396
CP077646	CP077649	CP078137	CP078555	CP078556	CP078558
CP078565	CP078566	CP078567	CP078568	CP078571	CP078573
CP078574	CP078575	CP078576	CP078577	CP078581	CP078584
CP078585	CP078586	CP078588	CP078589	CP078590	CP078592
CP079735	CP079736	CP079747	CP080001	CP080116	CP080117
CP080118	CP080119	CP080120	CP080121	CP080126	CP080133
CP080139	CP080146	CP080155	CP080163	CP080172	CP080223
CP080233	CP080235	CP080253	CP080255	CP080260	CP080370
CP080620	CP080645	CP081007	CP081008	CP081009	CP081010
CP081192	CP081306	CP081677	CP081684	CP081690	CP081698
CP081701	CP081706	CP081711	CP081713	CP081719	CP081724
CP081729	CP081736	CP081742	CP081748	CP081755	CP081762
CP081767	CP081774	CP081782	CP081791	CP081796	CP081805
CP081881	CP082120	CP082127	CP082129	CP082349	CP082355
CP082357	CP082824	CP082827	CP082831	CP082949	CP083263
CP083273	CP083410	CP083419	CP083478	CP083481	CP083492
CP083497	CP083503	CP083512	CP083517	CP083530	CP083534
CP083585	CP083701	CP083869	CP083872	CP083877	CP083880
CP083886	CP083888	CP084026	CP084096	CP084529	CP084534
CP084893	CP084894	CP084895	CP084896	CP084897	CP084898
CP084899	CP085056	CP085060	CP085063	CP085069	CP085074
CP085183	CP085188	CP085372	CP085488	CP085517	CP085619
CP085622	CP085637	CP085638	CP085842	CP086259	CP086334
CP086336	CP086338	CP086340	CP086342	CP086391	CP086556
CP086616	CP086618	CP086620	CP086622	CP086623	CP086624
CP086625	CP086626	CP086627	CP086662	CP087110	CP087377
CP087570	CP087578	CP088026	CP088028	CP088030	CP088032
CP088034	CP088036	CP088038	CP088040	CP088042	CP088044
CP088046	CP088048	CP088050	CP088052	CP088054	CP088056
CP088058	CP088060	CP088062	CP088064	CP088067	CP088069
CP088071	CP088344	CP088346	CP088348	CP088351	CP088352
CP088356	CP088363	CP088366	CP088369	CP088370	CP088374
CP088381	CP088383	CP088387	CP088390	CP088393	CP088397
CP088400	CP088406	CP088407	CP088408	CP088413	CP088414
CP088417	CP088421	CP088427	CP088433	CP088436	CP088443
CP088451	CP088452	CP088456	CP088461	CP088463	CP088466
CP088468	CP088473	CP088474	CP088479	CP088485	CP088486
CP088490	CP088494	CP088496	CP088500	CP088506	CP088509
CP088512	CP088514	CP088516	CP088520	CP088526	CP088529
CP088534	CP088542	CP088545	CP088547	CP088551	CP088553
CP088555	CP088560	CP088564	CP088566	CP088570	CP088576
CP088578	CP088580	CP088582	CP088584	CP088592	CP088593
CP088596	CP088598	CP088602	CP088607	CP088609	CP088612
CP088613	CP088617	CP088621	CP088624	CP088627	CP088629
CP088632	CP088637	CP088641	CP088643	CP088645	CP088649
CP088652	CP088654	CP088656	CP088658	CP088660	CP088662
CP088665	CP088673	CP088677	CP088681	CP088683	CP088687
CP088692	CP088694	CP088698	CP088701	CP088703	CP088707
CP088710	CP088714	CP088719	CP088722	CP088723	CP088725
CP088729	CP088734	CP088737	CP088740	CP088744	CP088754
CP088760	CP088764	CP088768	CP088771	CP088773	CP088776
CP088779	CP088782	CP088786	CP088790	CP088795	CP088800
CP088802	CP088804	CP088808	CP088812	CP088818	CP088820
CP088822	CP088826	CP088828	CP088829	CP088833	CP088835
CP088837	CP088839	CP088850	CP088851	CP088855	CP088860

Continued on next page

CP088864	CP088866	CP088869	CP088872	CP089031	CP089032
CP089034	CP089036	CP089142	CP089270	CP089272	CP089445
GL878028	GL896790	GL988050	HE572566	HE964769	HF572917
HG428755	HG738867	HG941718	JAADCD010000001	JAAJTL010000001	JAAJTN010000001
JAAJTO010000001	JAAJTP010000001	JAAJTR010000001	JAAJTS010000001	JAAJTT010000001	JAAJTU010000001
JAAJTV010000001	JAAKGF010000001	JAALAC010000002	JAANTD010000001	JAATHU010000001	JAATHV010000001
JAATHY010000001	JABWPS010000001	JABXOE010000001	JACDQE010000001	JACDQF010000001	JACDQG010000001
JACGZT020000001	JACIVJ010000001	JACIVK010000001	JACOJH010000001	JACOLT010000001	JACOLU010000001
JACRQJ010000001	JACSXT010000001	JACYGC010000008	JACYGD010000009	JACYGE010000009	JACYGM010000004
JACYGN010000008	JADALR010000001	JADALV010000001	JADALW010000001	JADDLK010000001	JADDLL010000001
JADDLM010000001	JADDLN010000001	JADDLP010000001	JADDLQ010000001	JAEFCJ010000001	JAEFCL010000001
JAEFCM010000001	JAEFCO010000001	JAEFCP010000001	JAEFCQ010000001	JAEPRM010000001	JAFBIE010000001
JAFCXR010000001	JAFFIC010000001	JAGDML010000001	JAGEVC010000001	JAGFYA010000002	JAGFYB010000001
JAGFYC010000001	JAGFYF010000001	JAGFZN010000001	JAGFZR010000001	JAGFZU010000001	JAGHKJ010000001
JAHMAB010000001	JAHQKD010000001	JAHQKR010000001	JAHQKS010000001	JAHQLA010000001	JAHQLB010000001
JAHSRN010000001	JAHSRO010000001	JAHSRP010000001	JAHSRQ010000001	JAHSRR010000001	JAHSRS010000001
JAHSRT010000001	JAHSRU010000001	JAHSRV010000001	JAHSRX010000001	JAHSRY010000001	JAHSRZ010000001
JAHSSA010000001	JAHSSB010000001	JAHSSC010000001	JAHSSD010000001	JAHSSE010000001	JAHWZI010000001
JAINSH010000001	JAINST010000001	JAINTH010000001	JAIQZL010000008	JAJATQ010000001	JAJGBP010000001
JAJJWB010000001	JAJJWE010000001	JASQ01000001	JH648165	JJMY02000001	JMJD01000003
KB732454	KB732522	KB732530	KB733023	KB733040	KB733069
KB733071	KB733078	KB733088	KB733094	KB733130	KB733135
KB733141	KB733164	KB733172	KB733201	KB733235	KB733365
KE137004	KE137028	KE137162	KE700461	KE700478	KE700541
KE700731	KI929668	KI929682	KI929714	KI929774	KI929787
KI929804	KK583188	KK736296	KQ087662	KQ087890	LAZO01000001
LM993812	LM995446	LN832404	LN874954	LORP02000005	LORS02000001
LQSL02000003	LQTX02000001	LQUJ02000001	LQVG02000001	LQVV02000005	LR025096
LR025101	LR130532	LR130552	LR130555	LR134152	LR134227
LR134234	LR134237	LR134247	LR134315	LR536431	LR595691
LR607328	LR607331	LR740758	LR740776	LR778140	LR778141
LR778142	LR778145	LR778146	LR778147	LR778148	LR778149
LR778151	LR778152	LR778153	LR782232	LR881940	LR882050
LR882052	LR882057	LR882493	LR882973	LR882978	LR882990
LR882997	LR883006	LR883012	LR883050	LR883965	LR890270
LR890299	LR890326	LR890334	LR890349	LR890410	LR890466
LR890508	LR890536	LR890606	LR890651	LR890693	LR890714
LR898868	LRDE01000001	LRDF01000001	LS483296	LS992166	LS992168
LS992171	LS992180	LS992185	LS992190	LS992192	LS998785
LS999560	LSUP01000001	LT615379	LT827011	LT838200	LT883142
LUYD01000001	LVRH02000001	LVSP02000001	LWDC01000001	MBNW01000001	MBNX01000003
MBNY01000001	MCRE01000001	MDLJ02000001	MPGR01000001	MPGS01000001	MPGT01000001
NBJI01000001	NC_000913	NC_002695	NC_004431	NC_007779	NC_008253
NC_009801	NC_010473	NC_011741	NC_011742	NC_011745	NC_011750
NC_011993	NC_012759	NC_012971	NC_013008	NC_013353	NC_013361
NC_013364	NC_013654	NC_013941	NC_017626	NC_017628	NC_017633
NC_017634	NC_017641	NC_017646	NC_017651	NC_017652	NC_017656
NC_017664	NC_020163	NC_022370	NC_022648	NGRV02000001	NGSF01000001
NHYO01000001	NHYP01000001	NHYQ01000001	NHYR01000001	NHYT01000001	NIFW01000002
NJEX02000002	NJIR01000001	NQCO02000001	NQCP02000001	NQCQ02000001	NQCR02000001
NQLI02000001	NWVR01000001	NXER01000001	OU342919	OU349837	OU349838
OU349839	OU349840	OU349841	OU349842	OU349843	OU349844
OU349845	OU349847	OU349848	OU349849	OU356913	PDDT01000002
PDED01000001	PDEK01000002	PDLD01000001	PDLF01000002	PDLJ01000002	PSQV01000007
PSQW01000002	PSQX01000006	PVMG01000001	QBDM01000001	QDAJ01000001	QLVH01000001
QSMQ02000006	QSMR02000002	QSMV02000001	QWLO01000001	QXTI01000001	RKJE01000005
RKJJ01000004	RKJK01000003	RKJN01000003	RRCS01000001	RYCV01000003	SEVB01000002
SEVD01000001	SEVE01000003	SEVF01000002	SEVG01000002	SEVI01000004	SEVL01000002
SEVM01000001	SEVN01000002	SEVO01000002	SEVP01000003	SEVQ01000001	SEVS01000006
SEVT01000005	SEVU01000007	SEVW01000003	SEVX01000001	SEVY01000001	SEWA01000004
SGYH01000001	SGYI01000001	SSWI01000001	SSWK01000001	U00096	UEFM01000001
UEFR01000001	UFYM01000003	UFYY01000003	UFYZ01000002	UFZD01000005	UFZF01000006
UFZG01000001	UFZT01000001	UGAE01000003	UGAH01000002	UGAK01000003	UGAO01000001
UGAS01000002	UGAV01000006	UGBA01000002	UGBB01000002	UGBD01000002	UGBE01000003
UGBF01000004	UGBJ01000001	UGBK01000002	UGBM01000005	UGBQ01000003	UGBV01000003
UGBX01000001	UGCH01000001	UGCI01000003	UGCT01000004	UGCW01000003	UGCY01000004
UGDB01000003	UGDG01000002	UGDJ01000004	UGDN01000003	UGDO01000002	UGDV01000004
UGDW01000001	UGDY01000003	UGEC01000002	UGEE01000003	UGEJ01000002	UGEL01000003
UGEU01000002	UGEV01000002	UGEZ01000005	UGFA01000002	UGFB01000004	UGFL01000003
UGFO01000006	UGFP01000001	UGFZ01000001	UGGK01000002	UWUZ01000001	UWVA01000001
UWVZ01000001	UWWG01000001	UWWW01000001	UWXK01000001	UWYG01000001	VCAQ01000001
VCYN01000001	VCYV01000001	VCZA01000001	VCZH01000001	VCZV01000001	VCZY01000001
VDAA01000002	VDBC01000001	VDBH01000001	VDBI01000001	VFAG01000002	VMRI01000001

Continued on next page

WIPD02000001	WMHS01000001	WMLE01000001	WNKN01000001	WNKO01000001	WSNS01000001
WSNT01000001	WXYW01000001				

Table S8. GenBank accession numbers of sequences used for *E. coli* mdh analysis.

AAKB02000001	AP017610	AP017620	AP018488	AP018784	AP018796
AP018802	AP018808	AP019189	AP019675	AP019761	AP019856
AP021890	AP021891	AP021892	AP021893	AP021894	AP021895
AP021896	AP021914	AP021933	AP021935	AP021944	AP021946
AP021963	AP021998	AP022003	AP022030	AP022032	AP022036
AP022044	AP022049	AP022064	AP022069	AP022087	AP022095
AP022098	AP022105	AP022107	AP022120	AP022159	AP022161
AP022171	AP022173	AP022177	AP022207	AP022215	AP022221
AP022222	AP022245	AP022261	AP022287	AP022295	AP022326
AP022351	AP022362	AP022409	AP022478	AP022482	AP022525
AP022533	AP022536	AP022540	AP022549	AP022650	AP022811
AP022815	AP023190	AP023197	AP023219	AP023224	AP023230
AP023235	AP023286	AP023427	AP024112	AP024114	AP024123
AP024126	AP024130	AP024131	AP024205	AP024471	AP024473
AP024475	AP024478	AP024518	AP024521	AP024560	AP024561
AP024582	AP024687	AP024694	AP024921	AP024924	AP025205
AP025209	AP025214	AP025220	APIN01000002	AXLH01000003	BA000007
BNEX01000001	BNEZ01000001	BNFC01000001	BNFH01000001	BNFI01000001	BNFJ01000001
BNFK01000001	BNFM01000001	BNFN01000001	BNFQ01000001	BNFS01000001	BNFV01000001
BNIP01000001	BNIQ01000001	BNIS01000001	BNIU01000001	BNIZ01000001	BNJA01000001
BNJB01000001	BNJC01000001	BNSX01000001	CABFNM010000001	CABFNN010000001	CABMNJ010000001
CABWHO010000003	CABWIT010000001	CABWIV010000003	CABWIW010000002	CABWIX010000001	CABWJF010000003
CABWJL010000001	CABWJM010000001	CABWJO010000005	CACRWL010000001	CACRWM010000001	CACRWO010000001
CACRWY010000001	CADDWP010000001	CADDWS010000001	CADDWT010000001	CADDWW010000001	CADDWX010000001
CADDWZ010000001	CADDXF010000001	CADDXG010000001	CADDXH010000001	CADDXI010000001	CADDXJ010000001
CADDXK010000001	CADDXL010000002	CADDXM010000001	CADDXN010000001	CADDXO010000001	CADDXP010000001
CADDXQ010000002	CADDXU010000001	CADEAI010000002	CAJGEG010000001	CAJGEH010000001	CAJGEI010000001
CAJGWN010000001	CAJMUK010000001	CAJMWD010000001	CAJNVV010000001	CAJOCA010000001	CM000662
CM000960	CM003707	CM007915	CP001509	CP002967	CP002970
CP003034	CP005930	CP006027	CP006262	CP006636	CP006698
CP006830	CP006834	CP007133	CP007136	CP007265	CP007391
CP007392	CP007393	CP007394	CP007442	CP007491	CP007592
CP007594	CP007799	CP008697	CP008801	CP008805	CP008957
CP009050	CP009072	CP009104	CP009106	CP009166	CP009273
CP009578	CP009644	CP009685	CP009789	CP009859	CP010116
CP010121	CP010122	CP010125	CP010132	CP010133	CP010134
CP010137	CP010145	CP010148	CP010150	CP010151	CP010152
CP010160	CP010163	CP010167	CP010169	CP010170	CP010172
CP010176	CP010177	CP010178	CP010180	CP010183	CP010186
CP010191	CP010196	CP010200	CP010206	CP010219	CP010221
CP010226	CP010229	CP010230	CP010231	CP010235	CP010237
CP010238	CP010240	CP010315	CP010344	CP010371	CP010439
CP010440	CP010441	CP010443	CP010585	CP010876	CP011124
CP011134	CP011320	CP011321	CP011322	CP011323	CP011324
CP011331	CP011342	CP011343	CP011915	CP011938	CP012126
CP012376	CP012380	CP012781	CP012802	CP012868	CP012870
CP013029	CP013031	CP013112	CP013658	CP013662	CP013663
CP013835	CP013952	CP014092	CP014111	CP014225	CP014269
CP014272	CP014314	CP014348	CP014488	CP014495	CP014583
CP015020	CP015023	CP015069	CP015074	CP015076	CP015085
CP015138	CP015159	CP015228	CP015229	CP015240	CP015241
CP015244	CP015831	CP015834	CP015842	CP015846	CP015853
CP015855	CP015912	CP016007	CP016358	CP016404	CP016546
CP016625	CP016755	CP017100	CP017220	CP017440	CP017444
CP017669	CP017844	CP017980	CP018103	CP018109	CP018206
CP018237	CP018239	CP018241	CP018243	CP018245	CP018247
CP018250	CP018252	CP018323	CP018770	CP018801	CP018948
CP018957	CP018965	CP018970	CP018976	CP018979	CP018983
CP018991	CP018995	CP019000	CP019008	CP019012	CP019029
CP019051	CP019213	CP019243	CP019245	CP019250	CP019256
CP019259	CP019267	CP019273	CP019280	CP019359	CP019392
CP019455	CP019558	CP019560	CP019629	CP019961	CP020025
CP020048	CP020055	CP020058	CP020092	CP020106	CP020107
CP020116	CP020368	CP020514	CP020835	CP020933	CP021179
CP021193	CP021202	CP021207	CP021288	CP021335	CP021339
CP021532	CP021535	CP021683	CP021691	CP021722	CP021732

Continued on next page

CP021736	CP021840	CP021879	CP021935	CP022229	CP022407
CP022414	CP022466	CP022664	CP022686	CP022689	CP022730
CP022912	CP023142	CP023165	CP023200	CP023201	CP023258
CP023346	CP023349	CP023357	CP023364	CP023383	CP023388
CP023531	CP023535	CP023541	CP023749	CP023815	CP023826
CP023844	CP023849	CP023870	CP023899	CP023960	CP024056
CP024138	CP024147	CP024223	CP024228	CP024232	CP024240
CP024243	CP024245	CP024256	CP024257	CP024260	CP024269
CP024273	CP024289	CP024293	CP024299	CP024479	CP024618
CP024650	CP024659	CP024661	CP024667	CP024720	CP024815
CP024821	CP024826	CP024830	CP024851	CP024855	CP024859
CP024862	CP024886	CP024889	CP024978	CP024992	CP025036
CP025048	CP025251	CP025328	CP025401	CP025520	CP025573
CP025627	CP025707	CP025716	CP025753	CP025842	CP025847
CP025849	CP025851	CP025859	CP025865	CP025869	CP025881
CP025892	CP025903	CP025910	CP025913	CP025916	CP025920
CP025950	CP026027	CP026028	CP026199	CP026355	CP026399
CP026491	CP026580	CP026723	CP026853	CP026929	CP026932
CP026935	CP026939	CP027060	CP027103	CP027105	CP027118
CP027134	CP027205	CP027221	CP027255	CP027307	CP027310
CP027312	CP027319	CP027328	CP027331	CP027335	CP027340
CP027342	CP027347	CP027351	CP027352	CP027355	CP027362
CP027363	CP027366	CP027368	CP027371	CP027373	CP027380
CP027387	CP027388	CP027390	CP027394	CP027430	CP027435
CP027437	CP027442	CP027445	CP027447	CP027449	CP027452
CP027454	CP027457	CP027459	CP027462	CP027472	CP027520
CP027534	CP027544	CP027546	CP027548	CP027550	CP027552
CP027572	CP027573	CP027577	CP027582	CP027584	CP027586
CP027587	CP027593	CP027597	CP027701	CP027766	CP027851
CP028110	CP028116	CP028117	CP028122	CP028126	CP028192
CP028305	CP028306	CP028307	CP028308	CP028309	CP028320
CP028379	CP028381	CP028429	CP028432	CP028589	CP028590
CP028698	CP028703	CP028714	CP029057	CP029108	CP029111
CP029115	CP029122	CP029164	CP029180	CP029238	CP029242
CP029369	CP029371	CP029420	CP029574	CP029576	CP029579
CP029630	CP029741	CP029747	CP029973	CP029981	CP030240
CP030331	CP030337	CP030767	CP030768	CP030919	CP031105
CP031134	CP031214	CP031215	CP031231	CP031256	CP031341
CP031343	CP031345	CP031347	CP031349	CP031353	CP031355
CP031546	CP031609	CP031653	CP031706	CP031916	CP031919
CP032066	CP032073	CP032085	CP032145	CP032201	CP032261
CP032265	CP032426	CP032515	CP032791	CP032793	CP032811
CP032879	CP032892	CP032936	CP033092	CP033096	CP033158
CP033605	CP033850	CP033884	CP034106	CP034384	CP034404
CP034428	CP034589	CP034658	CP034738	CP034792	CP034794
CP034797	CP034799	CP034801	CP034806	CP034808	CP034958
CP034966	CP035318	CP035321	CP035322	CP035330	CP035331
CP035339	CP035354	CP035366	CP035377	CP035476	CP035477
CP035486	CP035516	CP035720	CP035751	CP035767	CP035768
CP035770	CP035772	CP035812	CP035817	CP035819	CP035822
CP035832	CP035836	CP035841	CP035846	CP035850	CP035855
CP035860	CP035865	CP035870	CP035874	CP035879	CP035882
CP035884	CP036202	CP036245	CP037449	CP037857	CP037941
CP037943	CP037945	CP038180	CP038282	CP038284	CP038287
CP038290	CP038292	CP038295	CP038300	CP038302	CP038305
CP038309	CP038313	CP038316	CP038319	CP038321	CP038324
CP038328	CP038333	CP038336	CP038339	CP038342	CP038344
CP038346	CP038349	CP038351	CP038353	CP038355	CP038357
CP038360	CP038363	CP038366	CP038369	CP038372	CP038374
CP038376	CP038380	CP038383	CP038386	CP038389	CP038394
CP038398	CP038402	CP038405	CP038408	CP038412	CP038414
CP038416	CP038419	CP038421	CP038423	CP038425	CP038428
CP038453	CP038494	CP038496	CP038505	CP038791	CP038857
CP038859	CP039298	CP039403	CP039404	CP039753	CP039834
CP039837	CP040067	CP040090	CP040107	CP040263	CP040269
CP040305	CP040307	CP040309	CP040311	CP040313	CP040314
CP040316	CP040381	CP040390	CP040397	CP040456	CP040570
CP040572	CP040643	CP040663	CP040664	CP040667	CP040886
CP040919	CP040927	CP041002	CP041031	CP041110	CP041284
CP041300	CP041302	CP041304	CP041337	CP041359	CP041392
CP041411	CP041416	CP041422	CP041425	CP041429	CP041431
CP041433	CP041435	CP041437	CP041442	CP041448	CP041452
CP041520	CP041522	CP041526	CP041529	CP041532	CP041535

Continued on next page

CP041538	CP041541	CP041544	CP041546	CP041550	CP041556
CP041559	CP041563	CP041572	CP041578	CP041581	CP041623
CP041627	CP041628	CP041678	CP041747	CP041749	CP041919
CP041955	CP041996	CP042246	CP042250	CP042298	CP042336
CP042350	CP042470	CP042585	CP042588	CP042606	CP042843
CP042844	CP042845	CP042846	CP042871	CP042878	CP042892
CP042893	CP042896	CP042899	CP042901	CP042903	CP042934
CP042969	CP042973	CP042981	CP043033	CP043181	CP043183
CP043185	CP043187	CP043189	CP043191	CP043193	CP043195
CP043197	CP043199	CP043201	CP043203	CP043205	CP043207
CP043209	CP043211	CP043213	CP043217	CP043227	CP043406
CP043414	CP043478	CP043486	CP043487	CP043539	CP043541
CP043542	CP043733	CP043736	CP043739	CP043742	CP043744
CP043747	CP043750	CP043752	CP043754	CP043757	CP043852
CP043942	CP043946	CP043950	CP044021	CP044140	CP044145
CP044148	CP044291	CP044293	CP044305	CP044311	CP044312
CP044313	CP044314	CP044315	CP044346	CP044403	CP044410
CP045190	CP045206	CP045209	CP045213	CP045457	CP045712
CP045741	CP045827	CP045863	CP045975	CP045977	CP046000
CP046003	CP046006	CP046009	CP046025	CP046259	CP046396
CP046416	CP046527	CP046539	CP046676	CP046716	CP047002
CP047010	CP047074	CP047075	CP047076	CP047078	CP047079
CP047127	CP047231	CP047277	CP047378	CP047405	CP047455
CP047461	CP047571	CP047576	CP047594	CP047600	CP047609
CP047658	CP047662	CP047665	CP047710	CP047876	CP048010
CP048025	CP048107	CP048290	CP048304	CP048310	CP048322
CP048326	CP048330	CP048337	CP048344	CP048359	CP048367
CP048371	CP048376	CP048439	CP048603	CP048604	CP048605
CP048606	CP048647	CP048821	CP048853	CP048855	CP048857
CP048860	CP048863	CP048865	CP048867	CP048870	CP048873
CP048915	CP048920	CP048934	CP049050	CP049077	CP049081
CP049085	CP049086	CP049101	CP049118	CP049196	CP049197
CP049198	CP049201	CP049204	CP049299	CP049348	CP049353
CP049839	CP049845	CP049852	CP049936	CP049943	CP049967
CP049979	CP050031	CP050036	CP050040	CP050044	CP050047
CP050173	CP050193	CP050194	CP050195	CP050196	CP050197
CP050198	CP050199	CP050200	CP050202	CP050203	CP050204
CP050205	CP050206	CP050207	CP050208	CP050209	CP050210
CP050211	CP050213	CP050214	CP050216	CP050217	CP050218
CP050219	CP050220	CP050221	CP050289	CP050382	CP050498
CP050646	CP050862	CP050865	CP050998	CP051001	CP051158
CP051219	CP051222	CP051609	CP051615	CP051631	CP051688
CP051692	CP051694	CP051698	CP051700	CP051706	CP051711
CP051714	CP051716	CP051719	CP051725	CP051727	CP051733
CP051735	CP051738	CP051744	CP051749	CP051751	CP051753
CP051765	CP051776	CP051778	CP051782	CP051786	CP051790
CP051792	CP051796	CP051798	CP051800	CP051802	CP051804
CP051806	CP051808	CP051811	CP051814	CP051816	CP051819
CP051821	CP051824	CP051827	CP051830	CP051833	CP051837
CP051849	CP052057	CP052877	CP053079	CP053080	CP053211
CP053231	CP053234	CP053245	CP053247	CP053251	CP053258
CP053259	CP053281	CP053284	CP053296	CP053384	CP053600
CP053601	CP053602	CP053603	CP053604	CP053605	CP053607
CP053720	CP053723	CP053730	CP053731	CP053736	CP053785
CP053787	CP053851	CP053852	CP054214	CP054219	CP054224
CP054227	CP054230	CP054232	CP054236	CP054282	CP054317
CP054319	CP054325	CP054328	CP054335	CP054343	CP054345
CP054353	CP054363	CP054368	CP054371	CP054372	CP054379
CP054388	CP054407	CP054449	CP054454	CP054457	CP054556
CP054940	CP055158	CP055251	CP055256	CP055259	CP055426
CP055438	CP055608	CP055624	CP055649	CP055652	CP055660
CP055669	CP055681	CP055713	CP055718	CP055724	CP055736
CP055748	CP055753	CP055786	CP055796	CP055818	CP055888
CP055918	CP055923	CP055934	CP055941	CP055945	CP055951
CP055958	CP055964	CP055967	CP055971	CP055973	CP055976
CP055981	CP056036	CP056073	CP056077	CP056190	CP056237
CP056242	CP056248	CP056249	CP056263	CP056273	CP056278
CP056282	CP056299	CP056304	CP056315	CP056348	CP056355
CP056357	CP056370	CP056392	CP056396	CP056404	CP056446
CP056470	CP056481	CP056551	CP056556	CP056564	CP056565
CP056569	CP056578	CP056584	CP056594	CP056598	CP056604
CP056608	CP056613	CP056618	CP056620	CP056625	CP056632
CP056642	CP056655	CP056662	CP056786	CP056794	CP056800

Continued on next page

CP056810	CP056816	CP056821	CP056842	CP056846	CP056865
CP056867	CP056870	CP056873	CP056876	CP056894	CP056902
CP056914	CP056920	CP056921	CP056928	CP056933	CP056940
CP056947	CP056955	CP056974	CP056985	CP057007	CP057010
CP057012	CP057014	CP057016	CP057018	CP057020	CP057022
CP057024	CP057026	CP057028	CP057031	CP057038	CP057040
CP057045	CP057050	CP057055	CP057057	CP057062	CP057072
CP057075	CP057085	CP057087	CP057090	CP057109	CP057112
CP057115	CP057119	CP057123	CP057126	CP057129	CP057133
CP057137	CP057139	CP057141	CP057144	CP057152	CP057154
CP057160	CP057163	CP057166	CP057167	CP057171	CP057173
CP057175	CP057176	CP057178	CP057185	CP057186	CP057188
CP057190	CP057192	CP057194	CP057200	CP057204	CP057207
CP057223	CP057225	CP057234	CP057247	CP057250	CP057254
CP057259	CP057262	CP057267	CP057270	CP057273	CP057276
CP057281	CP057287	CP057293	CP057302	CP057334	CP057336
CP057340	CP057342	CP057344	CP057345	CP057347	CP057349
CP057354	CP057364	CP057368	CP057371	CP057378	CP057380
CP057396	CP057402	CP057408	CP057417	CP057420	CP057423
CP057429	CP057432	CP057435	CP057438	CP057441	CP057444
CP057447	CP057450	CP057453	CP057455	CP057465	CP057468
CP057470	CP057473	CP057475	CP057479	CP057484	CP057485
CP057487	CP057491	CP057493	CP057500	CP057507	CP057510
CP057512	CP057516	CP057520	CP057523	CP057525	CP057535
CP057539	CP057545	CP057555	CP057559	CP057570	CP057574
CP057577	CP057580	CP057582	CP057584	CP057587	CP057589
CP057594	CP057597	CP057600	CP057603	CP057605	CP057608
CP057612	CP057615	CP057617	CP057621	CP057626	CP057634
CP057636	CP057638	CP057640	CP057643	CP057645	CP057650
CP057660	CP057665	CP057669	CP057673	CP057677	CP057684
CP057686	CP057690	CP057693	CP057696	CP057705	CP057713
CP057717	CP057725	CP057729	CP057731	CP057738	CP057751
CP057759	CP057767	CP057773	CP057778	CP057784	CP057790
CP057796	CP057802	CP057804	CP057808	CP057812	CP057818
CP057826	CP057829	CP057831	CP057833	CP057838	CP057839
CP057841	CP057843	CP057849	CP057854	CP057860	CP057861
CP057869	CP057873	CP057876	CP057878	CP057879	CP057882
CP057885	CP057888	CP057891	CP057893	CP057900	CP057901
CP057905	CP057906	CP057911	CP057915	CP057916	CP057922
CP057927	CP057930	CP057932	CP057936	CP057941	CP057942
CP057946	CP057951	CP057953	CP057957	CP057959	CP057961
CP057962	CP057964	CP057967	CP057970	CP057973	CP057975
CP057978	CP057980	CP057983	CP057987	CP057990	CP057993
CP057994	CP057995	CP057996	CP058004	CP058007	CP058009
CP058012	CP058014	CP058019	CP058023	CP058025	CP058027
CP058031	CP058063	CP058070	CP058072	CP058074	CP058076
CP058078	CP058080	CP058083	CP058087	CP058090	CP058093
CP058101	CP058104	CP058223	CP058302	CP058308	CP058342
CP058355	CP058571	CP058574	CP058596	CP058618	CP058651
CP058656	CP058661	CP058682	CP058913	CP058948	CP059003
CP059043	CP059119	CP059125	CP059130	CP059137	CP059279
CP059281	CP059283	CP059288	CP059723	CP059835	CP059904
CP059907	CP059913	CP059917	CP059922	CP059929	CP059931
CP059944	CP059947	CP059953	CP059988	CP060057	CP060061
CP060065	CP060073	CP060075	CP060083	CP060121	CP060708
CP060709	CP061101	CP061108	CP061185	CP061206	CP061337
CP061339	CP061530	CP061749	CP061754	CP061758	CP061764
CP061914	CP062160	CP062203	CP062204	CP062211	CP062228
CP062249	CP062700	CP062702	CP062705	CP062708	CP062711
CP062713	CP062715	CP062717	CP062719	CP062721	CP062723
CP062725	CP062727	CP062729	CP062731	CP062733	CP062736
CP062739	CP062742	CP062744	CP062746	CP062749	CP062752
CP062755	CP062758	CP062761	CP062763	CP062766	CP062769
CP062771	CP062774	CP062778	CP062780	CP062782	CP062811
CP062815	CP062824	CP062826	CP062832	CP062838	CP062844
CP062851	CP062853	CP062855	CP062863	CP062865	CP062868
CP062871	CP062876	CP062883	CP062889	CP062895	CP062901
CP062908	CP062910	CP062924	CP062927	CP062930	CP062933
CP062967	CP062970	CP063046	CP063153	CP063214	CP063332
CP063341	CP063349	CP063369	CP063459	CP063473	CP063476
CP063479	CP063483	CP063487	CP063492	CP063499	CP063503
CP063511	CP063515	CP063518	CP063711	CP063713	CP063720
CP063729	CP063736	CP063739	CP063742	CP063774	CP063956

Continued on next page

CP063958	CP063969	CP063970	CP063979	CP063983	CP064103
CP064167	CP064677	CP064678	CP064679	CP064680	CP064681
CP064682	CP064683	CP064825	CP064847	CP065136	CP065152
CP065607	CP065611	CP065613	CP065615	CP065619	CP065624
CP065970	CP066032	CP066144	CP066366	CP066748	CP066753
CP066756	CP066759	CP066806	CP066836	CP066844	CP067232
CP067235	CP067241	CP067245	CP067250	CP067265	CP067274
CP067299	CP067303	CP067307	CP067311	CP067342	CP067399
CP067994	CP068035	CP068041	CP068155	CP068279	CP068281
CP068283	CP068394	CP068591	CP068706	CP068795	CP068796
CP068799	CP068800	CP068801	CP068802	CP068803	CP068804
CP068805	CP068806	CP068807	CP068808	CP068809	CP068810
CP068811	CP068812	CP068813	CP068814	CP068815	CP068816
CP068817	CP068818	CP068819	CP068820	CP068821	CP068822
CP068823	CP068824	CP068825	CP068826	CP068827	CP068828
CP068829	CP068830	CP068995	CP069132	CP069133	CP069134
CP069386	CP069438	CP069441	CP069445	CP069447	CP069451
CP069453	CP069459	CP069480	CP069488	CP069500	CP069501
CP069517	CP069522	CP069528	CP069542	CP069549	CP069559
CP069571	CP069583	CP069589	CP069646	CP069657	CP069666
CP069677	CP069682	CP069692	CP069706	CP069707	CP069709
CP069710	CP069712	CP069865	CP069882	CP069890	CP069893
CP069935	CP069945	CP069957	CP069973	CP069978	CP069980
CP069990	CP069996	CP070007	CP070026	CP070041	CP070045
CP070048	CP070063	CP070066	CP070071	CP070077	CP070098
CP070103	CP070111	CP070115	CP070124	CP070134	CP070148
CP070152	CP070157	CP070162	CP070170	CP070227	CP070232
CP070279	CP070280	CP070393	CP070399	CP070403	CP070593
CP070902	CP070906	CP070914	CP070920	CP070930	CP070962
CP071036	CP071050	CP071073	CP071133	CP071375	CP071436
CP071439	CP071441	CP071521	CP071522	CP071954	CP072054
CP072204	CP072322	CP072539	CP072661	CP072662	CP072663
CP072784	CP072785	CP072786	CP072787	CP072802	CP072858
CP072911	CP072976	CP072980	CP073360	CP073589	CP073591
CP073593	CP073595	CP073597	CP073599	CP073601	CP073603
CP073607	CP073609	CP073613	CP073617	CP073619	CP073621
CP073624	CP073718	CP073719	CP073768	CP074120	CP074576
CP074701	CP075061	CP075062	CP075374	CP075627	CP075632
CP075640	CP075649	CP075652	CP075663	CP075665	CP075697
CP075852	CP076123	CP076230	CP076232	CP076235	CP076237
CP076241	CP076243	CP076245	CP076293	CP076301	CP076305
CP076318	CP076344	CP076404	CP076527	CP076645	CP076646
CP076654	CP076687	CP076689	CP076693	CP076697	CP076704
CP076706	CP076709	CP076714	CP077215	CP077225	CP077243
CP077250	CP077266	CP077273	CP077281	CP077294	CP077314
CP077325	CP077342	CP077353	CP077361	CP077379	CP077389
CP077396	CP077646	CP077649	CP077969	CP078137	CP078557
CP078558	CP078565	CP078566	CP078570	CP078571	CP078573
CP078575	CP078577	CP078579	CP078579	CP078580	CP078581
CP078583	CP078584	CP078586	CP078587	CP078588	CP078589
CP078590	CP078591	CP078592	CP079735	CP079736	CP079747
CP080001	CP080116	CP080117	CP080118	CP080119	CP080120
CP080121	CP080126	CP080133	CP080139	CP080146	CP080155
CP080163	CP080172	CP080223	CP080233	CP080235	CP080253
CP080255	CP080260	CP080370	CP080620	CP080645	CP081007
CP081008	CP081009	CP081010	CP081192	CP081306	CP081677
CP081684	CP081690	CP081698	CP081701	CP081706	CP081711
CP081713	CP081719	CP081724	CP081729	CP081736	CP081742
CP081748	CP081755	CP081762	CP081767	CP081774	CP081782
CP081791	CP081796	CP081805	CP081881	CP082120	CP082127
CP082129	CP082349	CP082355	CP082357	CP082824	CP082827
CP082831	CP082949	CP083263	CP083273	CP083410	CP083419
CP083478	CP083481	CP083492	CP083497	CP083503	CP083512
CP083517	CP083530	CP083534	CP083585	CP083701	CP083869
CP083872	CP083877	CP083880	CP083886	CP083888	CP084026
CP084096	CP084529	CP084534	CP084893	CP084894	CP084895
CP084896	CP084897	CP084898	CP084899	CP085056	CP085060
CP085063	CP085069	CP085074	CP085183	CP085188	CP085372
CP085488	CP085517	CP085619	CP085622	CP085637	CP085638
CP085842	CP086259	CP086334	CP086336	CP086338	CP086340
CP086342	CP086391	CP086556	CP086616	CP086618	CP086620
CP086622	CP086623	CP086624	CP086625	CP086626	CP086627
CP086662	CP087110	CP087377	CP087570	CP087578	CP087958

Continued on next page

CP088026	CP088028	CP088030	CP088032	CP088034	CP088036
CP088038	CP088040	CP088042	CP088044	CP088046	CP088048
CP088050	CP088052	CP088054	CP088056	CP088058	CP088060
CP088062	CP088064	CP088067	CP088069	CP088071	CP088344
CP088346	CP088348	CP088351	CP088352	CP088356	CP088363
CP088366	CP088369	CP088370	CP088374	CP088381	CP088383
CP088387	CP088390	CP088393	CP088397	CP088400	CP088406
CP088407	CP088408	CP088413	CP088414	CP088417	CP088421
CP088427	CP088433	CP088436	CP088443	CP088451	CP088452
CP088456	CP088461	CP088463	CP088466	CP088468	CP088473
CP088474	CP088479	CP088485	CP088486	CP088490	CP088494
CP088496	CP088500	CP088506	CP088509	CP088512	CP088514
CP088516	CP088520	CP088526	CP088529	CP088534	CP088542
CP088545	CP088547	CP088551	CP088553	CP088555	CP088560
CP088564	CP088566	CP088570	CP088576	CP088578	CP088580
CP088582	CP088584	CP088592	CP088593	CP088596	CP088598
CP088602	CP088607	CP088609	CP088612	CP088613	CP088617
CP088621	CP088624	CP088627	CP088629	CP088632	CP088637
CP088641	CP088643	CP088645	CP088649	CP088652	CP088654
CP088656	CP088658	CP088660	CP088662	CP088665	CP088673
CP088677	CP088681	CP088683	CP088687	CP088692	CP088694
CP088698	CP088701	CP088703	CP088707	CP088710	CP088714
CP088719	CP088722	CP088723	CP088725	CP088729	CP088734
CP088737	CP088740	CP088744	CP088754	CP088760	CP088764
CP088768	CP088771	CP088773	CP088776	CP088779	CP088782
CP088786	CP088790	CP088795	CP088800	CP088802	CP088804
CP088808	CP088812	CP088818	CP088820	CP088822	CP088826
CP088828	CP088829	CP088833	CP088835	CP088837	CP088839
CP088850	CP088851	CP088855	CP088860	CP088864	CP088866
CP088869	CP088872	CP089031	CP089032	CP089034	CP089036
CP089142	CP089270	CP089272	CP089445	GL878028	GL896790
GL988050	HE572566	HE964769	HF572917	HG428755	HG738867
HG941718	JAAJTN010000001	JAAJTO010000001	JAAJTS010000001	JAAJTT010000001	JAAJTU010000001
JAAJTV010000001	JAAKGF010000001	JAALAC010000002	JAANTD010000001	JAATHU010000001	JAATHV010000001
JAATHY010000001	JABXOE010000001	JACDQE010000001	JACDQF010000001	JACDQG010000001	JACGZT020000001
JACIVJ010000001	JACOJH010000001	JACOLT010000001	JACOLU010000001	JACRQJ010000001	JACSXT010000001
JACXXS010000001	JACXXV010000001	JACXYM010000001	JACYGB010000002	JACYGC010000008	JACYGD010000009
JACYGE010000009	JACYGM010000004	JACYGN010000008	JADALR010000001	JADALV010000001	JADALW010000001
JADDLL010000001	JADDLN010000001	JADDLP010000001	JADDLQ010000001	JADDMK010000001	JADDML010000001
JAEFCL010000001	JAEFCM010000001	JAEFCO010000001	JAEFCP010000001	JAEFCQ010000001	JAEPRM010000001
JAFBIE010000001	JAFBIF010000001	JAFCXR010000001	JAFFIC010000001	JAGEVC010000001	JAGFYA010000002
JAGFYB010000001	JAGFYC010000001	JAGFYF010000001	JAGFZN010000001	JAGFZR010000001	JAGFZU010000001
JAGHKJ010000001	JAHMAB010000001	JAHQKD010000001	JAHQKS010000001	JAHQLA010000001	JAHQLB010000001
JAHSRN010000001	JAHSRO010000001	JAHSRP010000001	JAHSRQ010000001	JAHSRR010000001	JAHSRS010000001
JAHSRT010000001	JAHSRU010000001	JAHSRV010000001	JAHSRX010000001	JAHSRY010000001	JAHSRZ010000001
JAHSSA010000001	JAHSSC010000001	JAHSSD010000001	JAHSSE010000001	JAINSH010000001	JAINTH010000001
JAIQZL010000008	JAJATQ010000001	JAJGBP010000001	JAJJWA010000001	JAJJWB010000001	JAJJWE010000001
JAJJWF010000001	JASQ01000001	JASU01000001	JH648165	JJMY02000001	JMJD01000003
KB732454	KB732530	KB732574	KB732702	KB732739	KB732889
KB732916	KB732931	KB732993	KB733023	KB733040	KB733069
KB733078	KB733088	KB733094	KB733111	KB733130	KB733135
KB733141	KB733164	KB733172	KB733173	KB733235	KB733365
KE136980	KE137004	KE137028	KE137162	KE137211	KE700100
KE700461	KE700478	KE700541	KI929682	KI929714	KI929774
KI929782	KI929787	KI929804	KK583188	KK736296	KQ087687
KQ087890	LAZO01000001	LM993812	LM995446	LN832404	LN874954
LN877770	LORP02000005	LORS02000001	LPWC02000002	LQSL02000003	LQUJ02000001
LQVG02000001	LR025096	LR025101	LR130532	LR130545	LR134092
LR134220	LR134234	LR536430	LR536431	LR595691	LR607328
LR607331	LR740758	LR740776	LR778142	LR778143	LR778145
LR778146	LR778147	LR778148	LR778149	LR778151	LR778153
LR782232	LR882050	LR882052	LR882057	LR882973	LR882990
LR882997	LR883012	LR883050	LR883965	LR890270	LR890299
LR890326	LR890334	LR890410	LR890466	LR890508	LR890536
LR890606	LR890651	LR890693	LR890714	LR898874	LRDE01000001
LRDF01000001	LS992166	LS992168	LS992171	LS992180	LS992185
LS992190	LS992192	LS998785	LS999560	LSUP01000001	LT594504
LT601384	LT615372	LT615377	LT615379	LT827011	LT883142
LT903847	LUYD01000001	LVRH02000001	LVRJ02000001	LVSP02000001	LVTB02000001
LWDC01000001	MBNU01000003	MBNW01000001	MBNX01000003	MBNY01000001	MCRE01000001
MDLJ02000001	MIIL01000001	MPGR01000001	MPGS01000001	MPGT01000001	MRDN01000001
NBJG01000001	NC_002655	NC_002695	NC_004431	NC_007779	NC_008253
NC_008563	NC_009800	NC_010498	NC_011353	NC_011415	NC_011745

Continued on next page

NC_011748	NC_011751	NC_011993	NC_012759	NC_013008	NC_013353
NC_013361	NC_013654	NC_017626	NC_017628	NC_017631	NC_017632
NC_017634	NC_017635	NC_017638	NC_017641	NC_017646	NC_017651
NC_017656	NC_017660	NC_017664	NC_017906	NC_022364	NC_022370
NC_022648	NGSF01000001	NHYO01000001	NHYP01000001	NHYQ01000001	NHYR01000001
NHYS01000001	NIFW01000002	NJEX02000002	NJIR01000001	NQCO02000001	NQCP02000001
NQCQ02000001	NQCR02000001	NQLI02000001	NWVR01000001	NXER01000001	OU342919
OU349837	OU349838	OU349839	OU349840	OU349841	OU349842
OU349843	OU349844	OU349845	OU349846	OU349847	OU349848
OU356913	PDDT01000002	PDED01000001	PDEK01000002	PDLD01000001	PDLF01000002
PDLJ01000002	PSQV01000007	PSQW01000002	PSQX01000006	PVMG01000001	QBDM01000001
QDAJ01000001	QLVH01000001	QSMQ02000006	QSMR02000002	QSMV02000001	QWLO01000001
QXTI01000001	RKJE01000005	RKJJ01000004	RKJK01000003	RKJN01000003	RRCS01000001
RYCV01000003	SESS01000001	SEVB01000002	SEVD01000001	SEVE01000003	SEVG01000002
SEVI01000004	SEVM01000001	SEVN01000002	SEVP01000003	SEVS01000006	SEVT01000005
SEVU01000007	SEVW01000003	SEVX01000001	SEVY01000001	SGYH01000001	SGYI01000001
SSWI01000001	SSWJ01000001	SSWK01000001	UEFR01000001	UFYE01000002	UFYL01000003
UFYY01000003	UFYZ01000002	UFZB01000004	UFZG01000001	UFZI01000001	UFZV01000003
UFZW01000006	UGAE01000003	UGAH01000002	UGAK01000003	UGAL01000001	UGAM01000005
UGAO01000001	UGAS01000002	UGAU01000003	UGAV01000006	UGAX01000001	UGAZ01000001
UGBA01000002	UGBB01000002	UGBD01000002	UGBE01000003	UGBF01000004	UGBK01000002
UGBM01000005	UGBQ01000003	UGBV01000003	UGBW01000003	UGBX01000001	UGCB01000003
UGCH01000001	UGCI01000003	UGCT01000004	UGDB01000003	UGDG01000002	UGDJ01000004
UGDO01000002	UGDR01000001	UGDU01000003	UGDV01000004	UGDW01000001	UGDY01000003
UGEC01000002	UGEE01000003	UGEL01000003	UGES01000005	UGEU01000002	UGFA01000002
UGFB01000004	UGFL01000003	UGFO01000006	UGFT01000003	UGFU01000001	UGGA01000003
UGGG01000003	UGGI01000001	UGGK01000002	UWUZ01000001	UWVA01000001	UWVZ01000001
UWWG01000001	UYIJ01000003	VCYT01000001	VCYX01000001	VCZA01000001	VCZH01000001
VCZK01000001	VCZP01000001	VCZT01000001	VCZY01000001	VDAA01000002	VDAT01000001
VDBC01000001	VDBH01000001	VDBI01000001	VFAG01000002	WIPD02000001	WMHS01000001
WMLE01000001	WNKN01000001	WNKO01000001	WSNS01000001	WXYW01000001	

Table S9. GenBank accession numbers of sequences used for *E. coli* gyrB analysis.

AAKB02000001	ABHK02000001	AELA02000001	AKBV01000001	AM946981	AP017610
AP017617	AP017620	AP018488	AP018784	AP018796	AP018802
AP018808	AP019189	AP019675	AP019856	AP021890	AP021891
AP021892	AP021893	AP021894	AP021895	AP021896	AP021914
AP021933	AP021935	AP021944	AP021946	AP021963	AP021998
AP022003	AP022030	AP022032	AP022036	AP022044	AP022049
AP022064	AP022069	AP022087	AP022095	AP022098	AP022105
AP022107	AP022120	AP022159	AP022161	AP022171	AP022173
AP022177	AP022207	AP022215	AP022221	AP022222	AP022245
AP022261	AP022287	AP022295	AP022326	AP022351	AP022362
AP022409	AP022478	AP022482	AP022525	AP022533	AP022536
AP022540	AP022549	AP022650	AP022811	AP022815	AP023190
AP023197	AP023219	AP023230	AP023235	AP023286	AP023427
AP024112	AP024114	AP024123	AP024126	AP024130	AP024131
AP024205	AP024471	AP024473	AP024475	AP024478	AP024518
AP024521	AP024560	AP024582	AP024687	AP024694	AP024921
AP024924	AP025209	AP025214	APIN01000002	AXLH01000003	AXLI01000001
BA000007	BNEX01000001	BNFH01000001	BNFI01000001	BNFJ01000001	BNFK01000001
BNFN01000001	BNIP01000001	BNIQ01000001	BNIS01000001	BNSX01000001	CABEEY010000003
CABFNM010000001	CABFNN010000001	CABMNJ010000001	CABWHO010000003	CABWIT010000001	CABWIU010000001
CABWIV010000003	CABWIW010000002	CABWIX010000001	CABWIZ010000001	CABWJA010000001	CABWJB010000001
CABWJF010000003	CABWJL010000001	CABWJO010000005	CACRWJ010000001	CACRWL010000001	CACRWM010000001
CACRWO010000001	CACRWV010000001	CADDWP010000001	CADDWR010000001	CADDWS010000001	CADDWT010000001
CADDWW010000001	CADDWX010000001	CADDWY010000002	CADDWZ010000001	CADDXF010000001	CADDXG010000001
CADDXH010000001	CADDXI010000001	CADDXJ010000001	CADDXL010000002	CADDXM010000001	CADDXN010000001
CADDXP010000001	CADDXQ010000002	CADDXS010000001	CADDXU010000001	CADDXV010000001	CADEAI010000002
CAJGEG010000001	CAJGEH010000001	CAJGEI010000001	CAJGWN010000001	CAJMUK010000001	CAJMWD010000001
CAJNTK010000001	CAJNVV010000001	CAJSJQ010000001	CM000662	CP001509	CP002967
CP002970	CP003034	CP005930	CP006027	CP006632	CP006698
CP006830	CP007136	CP007275	CP007392	CP007393	CP007394
CP007442	CP007491	CP007592	CP007594	CP007799	CP008697
CP008805	CP009050	CP009072	CP009104	CP009106	CP009166
CP009273	CP009578	CP009644	CP009789	CP009859	CP010116
CP010117	CP010119	CP010121	CP010122	CP010125	CP010129
CP010132	CP010133	CP010134	CP010137	CP010140	CP010148
CP010151	CP010152	CP010157	CP010167	CP010169	CP010171
CP010172	CP010176	CP010177	CP010178	CP010180	CP010186

Continued on next page

CP010191	CP010196	CP010200	CP010213	CP010221	CP010228
CP010229	CP010230	CP010231	CP010235	CP010236	CP010237
CP010238	CP010240	CP010242	CP010304	CP010315	CP010344
CP010438	CP010439	CP010440	CP010442	CP010443	CP010585
CP010816	CP010876	CP011061	CP011124	CP011134	CP011320
CP011321	CP011322	CP011323	CP011324	CP011342	CP011343
CP011416	CP011938	CP012125	CP012126	CP012127	CP012376
CP012379	CP012380	CP012802	CP012870	CP013025	CP013029
CP013031	CP013112	CP013483	CP013658	CP013831	CP013952
CP014092	CP014111	CP014225	CP014270	CP014272	CP014314
CP014316	CP014488	CP014497	CP014522	CP014667	CP015020
CP015023	CP015069	CP015076	CP015085	CP015138	CP015159
CP015228	CP015229	CP015241	CP015244	CP015834	CP015843
CP015846	CP015853	CP015855	CP015912	CP016007	CP016182
CP016358	CP016404	CP016497	CP016546	CP017100	CP017669
CP017844	CP017979	CP017980	CP018103	CP018109	CP018206
CP018237	CP018239	CP018241	CP018243	CP018245	CP018247
CP018250	CP018252	CP018770	CP018801	CP018840	CP018948
CP018957	CP018965	CP018970	CP018976	CP018979	CP018983
CP018991	CP018995	CP019000	CP019005	CP019008	CP019012
CP019015	CP019020	CP019029	CP019051	CP019243	CP019245
CP019256	CP019259	CP019267	CP019271	CP019273	CP019280
CP019355	CP019359	CP019361	CP019392	CP019455	CP019558
CP019560	CP019629	CP019777	CP019778	CP019903	CP019961
CP020025	CP020048	CP020092	CP020116	CP020368	CP020545
CP020933	CP021175	CP021179	CP021202	CP021207	CP021288
CP021335	CP021339	CP021532	CP021683	CP021689	CP021691
CP021722	CP021726	CP021732	CP021736	CP021840	CP021879
CP021935	CP022229	CP022393	CP022407	CP022414	CP022466
CP022686	CP022689	CP022730	CP022912	CP023165	CP023200
CP023201	CP023258	CP023346	CP023349	CP023359	CP023364
CP023383	CP023388	CP023535	CP023541	CP023749	CP023815
CP023820	CP023826	CP023834	CP023844	CP023849	CP023853
CP023899	CP023960	CP024056	CP024127	CP024141	CP024223
CP024228	CP024232	CP024240	CP024243	CP024245	CP024256
CP024257	CP024260	CP024263	CP024269	CP024275	CP024278
CP024289	CP024479	CP024618	CP024650	CP024659	CP024661
CP024667	CP024717	CP024720	CP024815	CP024826	CP024830
CP024851	CP024855	CP024859	CP024862	CP024889	CP024978
CP024992	CP024997	CP025036	CP025048	CP025251	CP025268
CP025328	CP025401	CP025703	CP025707	CP025716	CP025739
CP025753	CP025840	CP025842	CP025847	CP025849	CP025851
CP025859	CP025862	CP025865	CP025869	CP025881	CP025892
CP025903	CP025910	CP025913	CP025916	CP025920	CP025950
CP026026	CP026027	CP026199	CP026202	CP026355	CP026357
CP026399	CP026491	CP026723	CP026853	CP026929	CP026932
CP026935	CP026939	CP027060	CP027103	CP027105	CP027134
CP027205	CP027219	CP027221	CP027255	CP027307	CP027310
CP027317	CP027323	CP027325	CP027328	CP027331	CP027338
CP027340	CP027355	CP027361	CP027362	CP027363	CP027366
CP027371	CP027380	CP027387	CP027388	CP027390	CP027430
CP027435	CP027437	CP027440	CP027442	CP027445	CP027447
CP027452	CP027454	CP027461	CP027462	CP027472	CP027534
CP027544	CP027548	CP027552	CP027572	CP027573	CP027577
CP027579	CP027582	CP027584	CP027586	CP027587	CP027591
CP027593	CP027597	CP027701	CP027766	CP027851	CP028110
CP028112	CP028116	CP028117	CP028126	CP028166	CP028192
CP028304	CP028305	CP028308	CP028309	CP028310	CP028381
CP028429	CP028432	CP028483	CP028589	CP028714	CP029057
CP029108	CP029111	CP029115	CP029122	CP029180	CP029238
CP029239	CP029371	CP029420	CP029574	CP029576	CP029579
CP029630	CP029692	CP029741	CP029747	CP029973	CP029981
CP030240	CP030331	CP030767	CP030768	CP030919	CP031105
CP031134	CP031157	CP031215	CP031231	CP031256	CP031321
CP031341	CP031345	CP031349	CP031353	CP031355	CP031546
CP031609	CP031706	CP032066	CP032085	CP032145	CP032201
CP032204	CP032261	CP032265	CP032426	CP032515	CP032805
CP032892	CP032936	CP033092	CP033158	CP033605	CP033635
CP033762	CP033884	CP034106	CP034384	CP034399	CP034426
CP034428	CP034589	CP034658	CP034738	CP034794	CP034797
CP034799	CP034801	CP034803	CP034806	CP034808	CP034958
CP034966	CP035317	CP035318	CP035320	CP035321	CP035322
CP035330	CP035339	CP035350	CP035354	CP035360	CP035366

Continued on next page

CP035377	CP035476	CP035477	CP035486	CP035516	CP035545
CP035720	CP035751	CP035767	CP035768	CP035770	CP035772
CP035812	CP035817	CP035819	CP035822	CP035832	CP035836
CP035841	CP035846	CP035850	CP035855	CP035860	CP035865
CP035870	CP035874	CP035879	CP035882	CP035884	CP036202
CP036245	CP037449	CP037857	CP037941	CP037943	CP037945
CP038351	CP038374	CP038376	CP038412	CP038496	CP038859
CP039298	CP039403	CP039404	CP039837	CP040269	CP040305
CP040307	CP040309	CP040311	CP040313	CP040314	CP040316
CP040456	CP040643	CP040663	CP040664	CP040886	CP040919
CP041002	CP041031	CP041284	CP041300	CP041302	CP041304
CP041337	CP041359	CP041422	CP041442	CP041448	CP041452
CP041520	CP041522	CP041526	CP041529	CP041532	CP041535
CP041538	CP041541	CP041544	CP041546	CP041550	CP041556
CP041559	CP041563	CP041572	CP041578	CP041581	CP041623
CP041627	CP041628	CP041749	CP041996	CP042246	CP042250
CP042336	CP042350	CP042470	CP042843	CP042844	CP042845
CP042846	CP042896	CP043033	CP043185	CP043195	CP043197
CP043199	CP043213	CP043217	CP043227	CP043406	CP043414
CP043478	CP043487	CP043539	CP043541	CP043736	CP043744
CP043852	CP043950	CP044140	CP044143	CP044145	CP044148
CP044293	CP044311	CP044312	CP044313	CP044314	CP044315
CP044403	CP044410	CP045190	CP045206	CP045209	CP045213
CP045457	CP045712	CP045741	CP045827	CP045863	CP045975
CP045977	CP046000	CP046003	CP046006	CP046009	CP046025
CP046259	CP046396	CP046416	CP046527	CP046539	CP046716
CP047002	CP047010	CP047074	CP047075	CP047076	CP047079
CP047127	CP047231	CP047277	CP047405	CP047455	CP047461
CP047571	CP047576	CP047594	CP047600	CP047609	CP047658
CP047662	CP047665	CP047710	CP047876	CP048010	CP048025
CP048107	CP048290	CP048304	CP048310	CP048322	CP048326
CP048330	CP048337	CP048344	CP048359	CP048367	CP048371
CP048376	CP048439	CP048603	CP048604	CP048605	CP048606
CP048647	CP048821	CP048853	CP048855	CP048857	CP048860
CP048863	CP048865	CP048867	CP048870	CP048873	CP048915
CP048920	CP048934	CP049050	CP049077	CP049081	CP049085
CP049086	CP049101	CP049118	CP049198	CP049201	CP049204
CP049299	CP049348	CP049353	CP049839	CP049845	CP049852
CP049936	CP049943	CP049967	CP049979	CP050031	CP050036
CP050040	CP050044	CP050047	CP050173	CP050193	CP050195
CP050196	CP050197	CP050198	CP050199	CP050200	CP050201
CP050202	CP050203	CP050204	CP050205	CP050206	CP050209
CP050210	CP050212	CP050213	CP050218	CP050219	CP050220
CP050221	CP050222	CP050289	CP050382	CP050498	CP050646
CP050862	CP050865	CP050998	CP051158	CP051219	CP051222
CP051609	CP051615	CP051631	CP051688	CP051694	CP051698
CP051700	CP051706	CP051711	CP051714	CP051716	CP051719
CP051725	CP051727	CP051733	CP051735	CP051738	CP051744
CP051749	CP051751	CP051753	CP051765	CP051776	CP051778
CP051782	CP051786	CP051790	CP051792	CP051796	CP051798
CP051800	CP051802	CP051804	CP051806	CP051808	CP051811
CP051814	CP051816	CP051819	CP051821	CP051824	CP051827
CP051830	CP051833	CP051837	CP051849	CP052057	CP052877
CP053079	CP053080	CP053211	CP053231	CP053234	CP053245
CP053247	CP053251	CP053258	CP053259	CP053281	CP053284
CP053296	CP053384	CP053600	CP053601	CP053602	CP053603
CP053604	CP053605	CP053607	CP053720	CP053723	CP053730
CP053731	CP053736	CP053785	CP053787	CP053851	CP053852
CP054214	CP054219	CP054224	CP054227	CP054230	CP054232
CP054236	CP054282	CP054314	CP054317	CP054319	CP054325
CP054328	CP054335	CP054343	CP054345	CP054353	CP054363
CP054368	CP054371	CP054372	CP054379	CP054388	CP054407
CP054449	CP054454	CP054457	CP054556	CP054940	CP055251
CP055256	CP055259	CP055426	CP055438	CP055608	CP055624
CP055649	CP055652	CP055660	CP055669	CP055681	CP055713
CP055718	CP055724	CP055736	CP055748	CP055753	CP055786
CP055796	CP055818	CP055888	CP055918	CP055923	CP055934
CP055941	CP055945	CP055951	CP055958	CP055964	CP055967
CP055971	CP055973	CP055976	CP055981	CP056036	CP056073
CP056077	CP056190	CP056237	CP056242	CP056248	CP056249
CP056263	CP056273	CP056278	CP056282	CP056299	CP056304
CP056315	CP056348	CP056355	CP056357	CP056370	CP056392
CP056396	CP056404	CP056446	CP056470	CP056481	CP056551

Continued on next page

CP056556	CP056564	CP056565	CP056569	CP056578	CP056584
CP056594	CP056598	CP056604	CP056608	CP056613	CP056618
CP056620	CP056625	CP056632	CP056642	CP056655	CP056662
CP056786	CP056794	CP056800	CP056810	CP056816	CP056821
CP056842	CP056846	CP056865	CP056867	CP056870	CP056873
CP056876	CP056894	CP056902	CP056914	CP056920	CP056921
CP056928	CP056933	CP056940	CP056947	CP056955	CP056974
CP056985	CP057007	CP057010	CP057012	CP057014	CP057016
CP057018	CP057020	CP057022	CP057024	CP057026	CP057028
CP057031	CP057038	CP057040	CP057045	CP057050	CP057055
CP057057	CP057062	CP057072	CP057075	CP057085	CP057087
CP057090	CP057109	CP057112	CP057115	CP057119	CP057123
CP057126	CP057129	CP057133	CP057137	CP057139	CP057141
CP057144	CP057152	CP057154	CP057160	CP057163	CP057166
CP057167	CP057171	CP057173	CP057175	CP057176	CP057178
CP057185	CP057186	CP057188	CP057190	CP057192	CP057194
CP057200	CP057204	CP057207	CP057223	CP057225	CP057234
CP057247	CP057250	CP057254	CP057259	CP057262	CP057267
CP057270	CP057273	CP057276	CP057281	CP057287	CP057293
CP057302	CP057334	CP057336	CP057340	CP057342	CP057344
CP057345	CP057347	CP057349	CP057354	CP057364	CP057368
CP057371	CP057378	CP057380	CP057396	CP057402	CP057408
CP057417	CP057420	CP057423	CP057429	CP057432	CP057435
CP057438	CP057441	CP057444	CP057447	CP057450	CP057453
CP057455	CP057465	CP057468	CP057470	CP057473	CP057475
CP057479	CP057484	CP057485	CP057487	CP057491	CP057493
CP057500	CP057507	CP057510	CP057512	CP057516	CP057520
CP057523	CP057525	CP057535	CP057539	CP057545	CP057555
CP057559	CP057570	CP057574	CP057577	CP057580	CP057582
CP057584	CP057587	CP057589	CP057594	CP057597	CP057600
CP057603	CP057605	CP057608	CP057612	CP057615	CP057617
CP057621	CP057626	CP057634	CP057636	CP057638	CP057640
CP057643	CP057645	CP057650	CP057660	CP057665	CP057669
CP057673	CP057677	CP057684	CP057686	CP057690	CP057693
CP057696	CP057705	CP057713	CP057717	CP057725	CP057729
CP057731	CP057738	CP057751	CP057759	CP057767	CP057773
CP057778	CP057784	CP057790	CP057796	CP057802	CP057804
CP057808	CP057812	CP057818	CP057826	CP057829	CP057831
CP057833	CP057838	CP057839	CP057841	CP057843	CP057849
CP057854	CP057860	CP057861	CP057869	CP057873	CP057876
CP057878	CP057879	CP057882	CP057885	CP057888	CP057891
CP057893	CP057900	CP057901	CP057905	CP057906	CP057911
CP057915	CP057916	CP057922	CP057927	CP057930	CP057932
CP057936	CP057941	CP057942	CP057946	CP057951	CP057953
CP057957	CP057959	CP057961	CP057962	CP057964	CP057967
CP057970	CP057973	CP057975	CP057978	CP057980	CP057983
CP057987	CP057990	CP057993	CP057994	CP057995	CP057996
CP058004	CP058007	CP058009	CP058012	CP058014	CP058019
CP058023	CP058025	CP058027	CP058031	CP058063	CP058070
CP058072	CP058074	CP058076	CP058078	CP058080	CP058083
CP058087	CP058090	CP058093	CP058101	CP058104	CP058223
CP058302	CP058308	CP058342	CP058355	CP058571	CP058574
CP058596	CP058618	CP058651	CP058656	CP058661	CP058682
CP058913	CP058948	CP059003	CP059043	CP059119	CP059125
CP059130	CP059137	CP059279	CP059281	CP059283	CP059288
CP059723	CP059835	CP059904	CP059907	CP059913	CP059917
CP059922	CP059925	CP059929	CP059931	CP059944	CP059947
CP059953	CP059988	CP060057	CP060061	CP060065	CP060073
CP060075	CP060083	CP060121	CP060708	CP060709	CP061101
CP061108	CP061185	CP061206	CP061329	CP061337	CP061339
CP061530	CP061749	CP061754	CP061758	CP061764	CP061914
CP062160	CP062203	CP062204	CP062211	CP062228	CP062243
CP062249	CP062700	CP062702	CP062705	CP062708	CP062711
CP062713	CP062715	CP062717	CP062719	CP062721	CP062723
CP062725	CP062727	CP062729	CP062731	CP062733	CP062736
CP062739	CP062742	CP062744	CP062746	CP062749	CP062752
CP062755	CP062758	CP062761	CP062763	CP062766	CP062769
CP062771	CP062774	CP062778	CP062780	CP062782	CP062811
CP062815	CP062824	CP062826	CP062832	CP062838	CP062844
CP062851	CP062853	CP062855	CP062863	CP062865	CP062868
CP062871	CP062876	CP062883	CP062889	CP062895	CP062901
CP062908	CP062910	CP062924	CP062927	CP062930	CP062933
CP062967	CP062970	CP062985	CP063046	CP063153	CP063332

Continued on next page

CP063341	CP063349	CP063369	CP063459	CP063473	CP063476
CP063479	CP063483	CP063487	CP063492	CP063499	CP063503
CP063511	CP063515	CP063518	CP063711	CP063713	CP063720
CP063729	CP063736	CP063739	CP063742	CP063774	CP063956
CP063958	CP063969	CP063970	CP063979	CP063983	CP064103
CP064167	CP064677	CP064678	CP064679	CP064680	CP064681
CP064682	CP064683	CP064847	CP065136	CP065152	CP065607
CP065611	CP065613	CP065615	CP065619	CP065624	CP065970
CP066032	CP066144	CP066366	CP066748	CP066753	CP066756
CP066759	CP066806	CP066836	CP066844	CP067232	CP067235
CP067241	CP067245	CP067250	CP067265	CP067274	CP067299
CP067303	CP067307	CP067311	CP067342	CP067399	CP067994
CP068035	CP068041	CP068155	CP068279	CP068281	CP068283
CP068394	CP068591	CP068706	CP068795	CP068796	CP068799
CP068800	CP068801	CP068802	CP068803	CP068804	CP068805
CP068806	CP068807	CP068808	CP068809	CP068810	CP068811
CP068812	CP068813	CP068814	CP068815	CP068816	CP068817
CP068818	CP068819	CP068820	CP068821	CP068822	CP068823
CP068824	CP068825	CP068826	CP068827	CP068828	CP068829
CP068830	CP068995	CP069132	CP069133	CP069134	CP069386
CP069438	CP069441	CP069445	CP069447	CP069451	CP069453
CP069459	CP069480	CP069488	CP069500	CP069501	CP069517
CP069522	CP069528	CP069542	CP069549	CP069559	CP069571
CP069583	CP069589	CP069646	CP069657	CP069666	CP069677
CP069682	CP069692	CP069706	CP069707	CP069709	CP069710
CP069712	CP069865	CP069882	CP069890	CP069893	CP069935
CP069945	CP069957	CP069973	CP069978	CP069980	CP069990
CP069996	CP070007	CP070026	CP070041	CP070045	CP070048
CP070063	CP070066	CP070071	CP070077	CP070098	CP070103
CP070111	CP070115	CP070124	CP070134	CP070148	CP070152
CP070157	CP070162	CP070170	CP070227	CP070232	CP070279
CP070280	CP070393	CP070399	CP070403	CP070593	CP070902
CP070906	CP070914	CP070920	CP070930	CP070962	CP071036
CP071050	CP071073	CP071133	CP071375	CP071436	CP071439
CP071441	CP071521	CP071522	CP071954	CP072054	CP072204
CP072322	CP072539	CP072661	CP072662	CP072663	CP072784
CP072785	CP072786	CP072787	CP072802	CP072858	CP072911
CP072976	CP072980	CP073589	CP073591	CP073593	CP073595
CP073597	CP073599	CP073601	CP073603	CP073607	CP073609
CP073613	CP073617	CP073619	CP073621	CP073624	CP073718
CP073719	CP073768	CP074120	CP074576	CP074701	CP075061
CP075062	CP075374	CP075472	CP075474	CP075477	CP075484
CP075487	CP075627	CP075632	CP075640	CP075649	CP075652
CP075663	CP075665	CP075697	CP075852	CP076123	CP076230
CP076232	CP076235	CP076237	CP076241	CP076243	CP076245
CP076293	CP076301	CP076305	CP076318	CP076344	CP076404
CP076527	CP076645	CP076646	CP076654	CP076687	CP076689
CP076693	CP076697	CP076704	CP076706	CP076709	CP076714
CP077063	CP077215	CP077225	CP077243	CP077250	CP077266
CP077273	CP077281	CP077294	CP077314	CP077325	CP077342
CP077353	CP077361	CP077379	CP077389	CP077396	CP077646
CP077649	CP078137	CP078555	CP078557	CP078565	CP078566
CP078568	CP078570	CP078571	CP078573	CP078575	CP078576
CP078578	CP078581	CP078582	CP078583	CP078584	CP078585
CP078586	CP078587	CP078588	CP078589	CP078591	CP078592
CP079735	CP079736	CP079747	CP080001	CP080116	CP080117
CP080118	CP080119	CP080120	CP080121	CP080126	CP080133
CP080139	CP080146	CP080155	CP080163	CP080172	CP080223
CP080233	CP080235	CP080253	CP080255	CP080260	CP080370
CP080620	CP080645	CP081007	CP081008	CP081009	CP081010
CP081192	CP081306	CP081677	CP081684	CP081690	CP081698
CP081701	CP081706	CP081711	CP081713	CP081719	CP081724
CP081729	CP081736	CP081742	CP081748	CP081755	CP081762
CP081767	CP081774	CP081782	CP081791	CP081796	CP081805
CP081881	CP082120	CP082127	CP082129	CP082349	CP082355
CP082357	CP082824	CP082827	CP082831	CP082949	CP083263
CP083273	CP083410	CP083419	CP083478	CP083481	CP083492
CP083497	CP083503	CP083512	CP083517	CP083530	CP083534
CP083585	CP083701	CP083869	CP083872	CP083877	CP083880
CP083886	CP083888	CP084026	CP084096	CP084529	CP084534
CP084893	CP084894	CP084895	CP084896	CP084897	CP084898
CP084899	CP085056	CP085060	CP085063	CP085069	CP085074
CP085183	CP085188	CP085372	CP085488	CP085517	CP085619

Continued on next page

CP085622	CP085637	CP085638	CP085842	CP086259	CP086334
CP086336	CP086338	CP086340	CP086342	CP086391	CP086556
CP086616	CP086618	CP086620	CP086622	CP086623	CP086624
CP086625	CP086626	CP086627	CP086662	CP087110	CP087377
CP087570	CP087578	CP087958	CP088026	CP088028	CP088030
CP088032	CP088034	CP088036	CP088038	CP088040	CP088042
CP088044	CP088046	CP088048	CP088050	CP088052	CP088054
CP088056	CP088058	CP088060	CP088062	CP088064	CP088067
CP088069	CP088071	CP088344	CP088346	CP088348	CP088351
CP088352	CP088356	CP088363	CP088366	CP088369	CP088370
CP088374	CP088381	CP088383	CP088387	CP088390	CP088393
CP088397	CP088400	CP088406	CP088407	CP088408	CP088413
CP088414	CP088417	CP088421	CP088427	CP088433	CP088436
CP088443	CP088451	CP088452	CP088456	CP088461	CP088463
CP088466	CP088468	CP088473	CP088474	CP088479	CP088485
CP088486	CP088490	CP088494	CP088496	CP088500	CP088506
CP088509	CP088512	CP088514	CP088516	CP088520	CP088526
CP088529	CP088542	CP088545	CP088547	CP088551	CP088553
CP088555	CP088560	CP088564	CP088566	CP088570	CP088576
CP088578	CP088580	CP088582	CP088584	CP088592	CP088593
CP088596	CP088598	CP088602	CP088607	CP088609	CP088612
CP088613	CP088617	CP088621	CP088624	CP088627	CP088629
CP088632	CP088637	CP088641	CP088643	CP088645	CP088649
CP088652	CP088654	CP088656	CP088658	CP088660	CP088662
CP088665	CP088673	CP088677	CP088681	CP088683	CP088687
CP088692	CP088694	CP088698	CP088701	CP088703	CP088707
CP088710	CP088714	CP088719	CP088722	CP088723	CP088725
CP088729	CP088734	CP088737	CP088740	CP088744	CP088754
CP088760	CP088764	CP088768	CP088771	CP088773	CP088776
CP088779	CP088782	CP088786	CP088790	CP088795	CP088800
CP088802	CP088804	CP088808	CP088812	CP088818	CP088820
CP088822	CP088826	CP088828	CP088829	CP088833	CP088835
CP088837	CP088839	CP088850	CP088851	CP088855	CP088860
CP088864	CP088866	CP088869	CP088872	CP089031	CP089032
CP089034	CP089036	CP089142	CP089270	CP089272	CP089445
GL878028	GL896790	GL988050	HE572566	HE964769	HF572917
HG428755	HG738867	HG941718	JAAJTO010000001	JAAJTP010000001	JAAJTS010000001
JAAJTT010000001	JAAJTU010000001	JAAKGF010000001	JAALAC010000002	JAANTD010000001	JAATHU010000001
JAATHV010000001	JAATHY010000001	JABWPS010000001	JACDQE010000001	JACDQF010000001	JACDQG010000001
JACGZL020000001	JACGZM020000001	JACGZT020000001	JACIVJ010000001	JACIVK010000001	JACOJC010000001
JACOJH010000001	JACOLT010000001	JACOLU010000001	JACRQJ010000001	JACSXT010000001	JACXYM010000001
JACYGB010000002	JACYGC010000008	JACYGD010000009	JACYGE010000009	JACYGM010000004	JACYGN010000008
JADALR010000001	JADALV010000001	JADALW010000001	JADDLL010000001	JADDLN010000001	JADDLP010000001
JADDMK010000001	JAEFCL010000001	JAEFCM010000001	JAEFCO010000001	JAEFCP010000001	JAEFCQ010000001
JAEPRM010000001	JAEPRN010000001	JAFBIE010000001	JAFBIF010000001	JAFCXR010000001	JAFFIC010000001
JAGDML010000001	JAGFYA010000002	JAGFYC010000001	JAGFYF010000001	JAGFZN010000001	JAGFZR010000001
JAGFZU010000001	JAGHKJ010000001	JAHQKD010000001	JAHSRO010000001	JAHSRQ010000001	JAHSRR010000001
JAHSRS010000001	JAHSRT010000001	JAHSRV010000001	JAHSRX010000001	JAHSSA010000001	JAHSSC010000001
JAINTH010000001	JAIQZL010000008	JAJJWB010000001	JAJJWE010000001	JAJJWF010000001	JASQ01000001
JASU01000001	JH648165	JMJD01000003	KB732454	KB732530	KB732535
KB732574	KB732580	KB732702	KB732916	KB732931	KB732993
KB733023	KB733071	KB733078	KB733094	KB733111	KB733130
KB733135	KB733141	KB733164	KB733173	KB733201	KB733361
KB733365	KE137028	KE137155	KE137162	KE700230	KE700461
KE700478	KE700541	KE700731	KI929668	KI929682	KI929714
KI929774	KI929782	KI929787	KI929804	KK583188	KK736296
KQ087662	KQ087687	KQ087890	LAZO01000001	LM993812	LM995446
LN832404	LN877770	LORP02000005	LORS02000001	LPWC02000002	LQSL02000003
LQUJ02000001	LQVG02000001	LR025096	LR025101	LR130532	LR134234
LR595691	LR607328	LR607331	LR740758	LR740776	LR778141
LR778142	LR778143	LR778145	LR778146	LR778147	LR778148
LR778149	LR778150	LR778151	LR778152	LR778153	LR782232
LR881940	LR882050	LR882052	LR882057	LR882493	LR882973
LR882990	LR882997	LR883006	LR883012	LR883050	LR883965
LR890270	LR890299	LR890326	LR890334	LR890410	LR890466
LR890508	LR890693	LRDE01000001	LRDF01000001	LS992166	LS992168
LS992171	LS992180	LS992185	LS992190	LS992192	LS998785
LS999560	LT594504	LT601384	LT615372	LT615374	LT615377
LT632320	LT827011	LT883142	LT903847	LUYD01000001	LVRH02000001
LVRJ02000001	LVSP02000001	LVTB02000001	LWDC01000001	MBNU01000003	MBNW01000001
MBNX01000003	MBNY01000001	MCRE01000001	MDLJ02000001	MIIL01000001	MPGR01000001
MPGS01000001	MPGT01000001	MRDN01000001	NBJI01000001	NC_000913	NC_002655
NC_002695	NC_007779	NC_008253	NC_008563	NC_009800	NC_009801

Continued on next page

NC_010473	NC_010498	NC_011353	NC_011415	NC_011601	NC_011741
NC_011748	NC_011750	NC_012759	NC_012967	NC_013008	NC_013361
NC_013364	NC_013654	NC_013941	NC_017626	NC_017633	NC_017634
NC_017635	NC_017641	NC_017646	NC_017651	NC_017652	NC_017656
NC_017660	NC_017664	NC_022364	NC_022370	NGSF01000001	NHYP01000001
NHYQ01000001	NHYR01000001	NHYS01000001	NHYT01000001	NIFW01000002	NJEX02000002
NJIR01000001	NQCO02000001	NQCP02000001	NQCQ02000001	NQCR02000001	NQLI02000001
NWVR01000001	NXER01000001	OU342919	OU349837	OU349838	OU349839
OU349840	OU349841	OU349842	OU349843	OU349845	OU349846
OU349849	OU356913	PDDT01000002	PDED01000001	PDEK01000002	PDLD01000001
PDLF01000002	PDLJ01000002	PSQV01000007	PSQW01000002	PSQX01000006	PVMG01000001
QBDM01000001	QDAJ01000001	QLVH01000001	QSMQ02000006	QSMR02000002	QSMV02000001
QWLO01000001	QXTI01000001	RKJE01000005	RKJJ01000004	RKJK01000003	RKJN01000003
RRCS01000001	SESS01000001	SEVA01000001	SEVB01000002	SEVD01000001	SEVE01000003
SEVF01000002	SEVI01000004	SEVL01000002	SEVM01000001	SEVN01000002	SEVO01000002
SEVP01000003	SEVQ01000001	SEVS01000006	SEVT01000005	SEVU01000007	SEVW01000003
SEVX01000001	SEVY01000001	SEWA01000004	SGYH01000001	SGYI01000001	SWKA01000005
U00096	UEFM01000001	UEFR01000001	UFYE01000002	UFYL01000003	UFYY01000003
UFYZ01000002	UFZB01000004	UFZD01000005	UFZF01000006	UFZV01000003	UFZW01000006
UGAE01000003	UGAH01000002	UGAM01000005	UGAS01000002	UGAT01000002	UGAU01000003
UGAV01000006	UGAY01000002	UGBA01000002	UGBB01000002	UGBD01000002	UGBE01000003
UGBF01000004	UGBK01000002	UGBM01000005	UGBP01000004	UGBX01000001	UGCB01000003
UGCH01000001	UGCI01000003	UGCJ01000003	UGCT01000004	UGCY01000004	UGCZ01000001
UGDB01000003	UGDG01000002	UGDJ01000004	UGDK01000002	UGDO01000002	UGDU01000003
UGDV01000004	UGDY01000003	UGEC01000002	UGEE01000003	UGEF01000002	UGEJ01000002
UGEL01000003	UGEU01000002	UGEV01000002	UGEZ01000005	UGFB01000004	UGFO01000006
UGFT01000003	UGGG01000003	UGGI01000001	UGGK01000002	UWUZ01000001	UWVC01000001
UWVG01000001	UWVZ01000001	UWWG01000001	UWXK01000001	UWYG01000001	VCAQ01000001
VCYN01000001	VCYV01000001	VCYX01000001	VCZA01000001	VCZG01000001	VCZH01000001
VCZI01000001	VCZK01000001	VCZP01000001	VCZX01000001	VCZY01000001	VDAA01000002
VDAB01000001	VDAW01000001	VDBC01000001	VDBH01000001	VFAG01000002	VHKN01000001
VMRI01000001	WIPD02000001	WMHS01000001	WNKO01000001	WSNS01000001	

Table S10. GenBank accession numbers of sequences used for *E. coli* purA analysis.

AAKB02000001	ABHK02000001	AELA02000001	AP017610	AP017617	AP017620
AP018488	AP018784	AP018796	AP018802	AP018808	AP019189
AP019675	AP019761	AP019856	AP021890	AP021891	AP021892
AP021893	AP021894	AP021895	AP021896	AP021914	AP021933
AP021935	AP021944	AP021946	AP021963	AP021998	AP022003
AP022030	AP022032	AP022036	AP022044	AP022049	AP022064
AP022069	AP022087	AP022095	AP022098	AP022105	AP022107
AP022120	AP022159	AP022161	AP022171	AP022173	AP022177
AP022207	AP022215	AP022221	AP022222	AP022245	AP022261
AP022287	AP022295	AP022298	AP022326	AP022351	AP022362
AP022409	AP022478	AP022482	AP022525	AP022533	AP022536
AP022540	AP022549	AP022650	AP022811	AP022815	AP023190
AP023197	AP023219	AP023226	AP023230	AP023235	AP023237
AP023286	AP023427	AP024112	AP024114	AP024123	AP024126
AP024130	AP024131	AP024205	AP024471	AP024473	AP024475
AP024478	AP024518	AP024521	AP024560	AP024582	AP024687
AP024694	AP024921	AP024924	AP025205	AP025209	AP025214
AP025220	APIN01000002	AXLH01000003	AXLI01000001	BA000007	BNEX01000001
BNFC01000001	BNFH01000001	BNFI01000001	BNFJ01000001	BNFK01000001	BNFN01000001
BNFQ01000001	BNIP01000001	BNIQ01000001	BNIR01000001	BNIS01000001	BNIT01000001
BNIV01000001	BNIW01000001	BNIZ01000001	BNJA01000001	BNJB01000001	BNSX01000001
CABEEY010000003	CABFNM010000001	CABFNN010000001	CABMNJ010000001	CABWHO010000003	CABWIU010000001
CABWIX010000001	CABWJC010000001	CABWJF010000003	CABWJL010000001	CABWJM010000001	CABWJO010000005
CACRWJ010000001	CACRWK010000001	CACRWL010000001	CACRWM010000001	CACRWO010000001	CACRWV010000001
CADDWP010000001	CADDWR010000001	CADDWS010000001	CADDWW010000001	CADDWX010000001	CADDWY010000002
CADDWZ010000001	CADDXF010000001	CADDXJ010000001	CADDXL010000002	CADDXM010000001	CADDXP010000001
CADDXQ010000002	CADDXU010000001	CADEAI010000002	CAJGEG010000001	CAJGEH010000001	CAJGEI010000001
CAJGWN010000001	CAJMUK010000001	CAJMWD010000001	CAJNJR010000001	CAJNTK010000001	CAJNVV010000001
CAJNXN010000001	CAJOCA010000001	CAJSIA010000001	CAJSJQ010000001	CM000662	CM000960
CM003707	CP001509	CP002967	CP002970	CP003034	CP005930
CP006027	CP006698	CP006830	CP006834	CP007133	CP007136
CP007265	CP007391	CP007392	CP007393	CP007592	CP007594
CP007799	CP008805	CP008957	CP009050	CP009072	CP009104
CP009106	CP009166	CP009273	CP009578	CP009644	CP009685
CP009789	CP009859	CP010116	CP010117	CP010119	CP010121
CP010132	CP010133	CP010134	CP010143	CP010148	CP010150

Continued on next page

CP010151	CP010160	CP010163	CP010167	CP010169	CP010171
CP010176	CP010177	CP010180	CP010183	CP010186	CP010191
CP010206	CP010213	CP010219	CP010226	CP010228	CP010229
CP010231	CP010235	CP010237	CP010238	CP010240	CP010304
CP010344	CP010444	CP010876	CP011018	CP011113	CP011124
CP011134	CP011320	CP011321	CP011322	CP011323	CP011324
CP011342	CP011343	CP011416	CP011495	CP011915	CP012376
CP012379	CP012380	CP012633	CP012693	CP012781	CP012802
CP013025	CP013029	CP013031	CP013048	CP013185	CP013483
CP013658	CP013662	CP013663	CP013831	CP013835	CP013952
CP014092	CP014225	CP014316	CP014348	CP014522	CP015020
CP015023	CP015069	CP015074	CP015076	CP015085	CP015138
CP015159	CP015228	CP015229	CP015240	CP015241	CP015831
CP015832	CP015834	CP015842	CP015843	CP015846	CP015853
CP015855	CP016007	CP016182	CP016404	CP016546	CP016625
CP016755	CP016828	CP017251	CP017434	CP017436	CP017438
CP017440	CP017442	CP017446	CP017631	CP017669	CP017980
CP018103	CP018109	CP018115	CP018206	CP018237	CP018239
CP018241	CP018243	CP018245	CP018247	CP018250	CP018252
CP018323	CP018801	CP018948	CP018953	CP018965	CP018970
CP018976	CP018979	CP018983	CP018991	CP019005	CP019008
CP019012	CP019015	CP019029	CP019051	CP019071	CP019213
CP019245	CP019250	CP019256	CP019267	CP019271	CP019273
CP019280	CP019355	CP019359	CP019361	CP019392	CP019455
CP019560	CP019629	CP019903	CP019944	CP019953	CP019961
CP020025	CP020048	CP020055	CP020058	CP020092	CP020106
CP020107	CP020116	CP020368	CP020495	CP020509	CP020516
CP020520	CP020545	CP020933	CP021175	CP021179	CP021193
CP021202	CP021207	CP021288	CP021335	CP021339	CP021454
CP021532	CP021535	CP021689	CP021691	CP021722	CP021726
CP021732	CP021736	CP021840	CP021844	CP021879	CP021935
CP022050	CP022086	CP022154	CP022229	CP022279	CP022393
CP022407	CP022466	CP022686	CP022689	CP022730	CP023061
CP023165	CP023201	CP023258	CP023346	CP023349	CP023353
CP023359	CP023364	CP023377	CP023383	CP023386	CP023541
CP023673	CP023815	CP023820	CP023826	CP023834	CP023844
CP023849	CP023853	CP023870	CP023960	CP024127	CP024134
CP024138	CP024141	CP024147	CP024223	CP024228	CP024232
CP024243	CP024245	CP024256	CP024257	CP024260	CP024269
CP024275	CP024289	CP024293	CP024479	CP024618	CP024650
CP024659	CP024661	CP024667	CP024717	CP024720	CP024801
CP024815	CP024821	CP024826	CP024830	CP024851	CP024859
CP024862	CP024886	CP024978	CP024992	CP024997	CP025036
CP025048	CP025251	CP025268	CP025318	CP025328	CP025401
CP025573	CP025703	CP025707	CP025716	CP025747	CP025753
CP025840	CP025847	CP025849	CP025851	CP025856	CP025859
CP025862	CP025865	CP025869	CP025881	CP025892	CP025903
CP025910	CP025916	CP025920	CP025950	CP026026	CP026027
CP026028	CP026202	CP026355	CP026399	CP026491	CP026580
CP026755	CP026853	CP026929	CP026932	CP026935	CP026939
CP027060	CP027103	CP027105	CP027118	CP027126	CP027134
CP027140	CP027205	CP027219	CP027221	CP027255	CP027310
CP027312	CP027317	CP027319	CP027323	CP027325	CP027328
CP027331	CP027335	CP027338	CP027340	CP027342	CP027347
CP027351	CP027352	CP027355	CP027361	CP027362	CP027363
CP027366	CP027368	CP027373	CP027380	CP027387	CP027388
CP027390	CP027430	CP027435	CP027437	CP027440	CP027449
CP027452	CP027454	CP027457	CP027461	CP027462	CP027520
CP027534	CP027544	CP027546	CP027550	CP027573	CP027584
CP027586	CP027587	CP027591	CP027593	CP027597	CP027599
CP027701	CP027851	CP028110	CP028112	CP028116	CP028122
CP028126	CP028305	CP028309	CP028310	CP028320	CP028379
CP028429	CP028432	CP028483	CP028578	CP028589	CP028592
CP028600	CP028603	CP028607	CP028609	CP028623	CP028641
CP028647	CP028650	CP028652	CP028654	CP028674	CP028677
CP028680	CP028685	CP028693	CP028695	CP028698	CP028703
CP028714	CP029057	CP029108	CP029111	CP029115	CP029164
CP029180	CP029238	CP029239	CP029420	CP029574	CP029579
CP029630	CP029687	CP029692	CP029741	CP029747	CP029973
CP029981	CP030240	CP030281	CP030331	CP030337	CP030767
CP030768	CP030939	CP031105	CP031134	CP031157	CP031214
CP031215	CP031231	CP031256	CP031321	CP031341	CP031343
CP031345	CP031347	CP031349	CP031353	CP031355	CP031609

Continued on next page

CP031653	CP031706	CP031892	CP031898	CP031902	CP031912
CP031919	CP031922	CP032066	CP032073	CP032078	CP032085
CP032145	CP032201	CP032204	CP032237	CP032261	CP032265
CP032515	CP032789	CP032791	CP032793	CP032797	CP032801
CP032803	CP032805	CP032808	CP032811	CP032879	CP032936
CP032989	CP032992	CP033096	CP033158	CP033250	CP033378
CP033401	CP033635	CP033762	CP033850	CP033884	CP034384
CP034399	CP034404	CP034428	CP034589	CP034658	CP034727
CP034734	CP034738	CP034745	CP034787	CP034792	CP034797
CP034799	CP034801	CP034803	CP034806	CP034808	CP034843
CP034953	CP034958	CP034966	CP035123	CP035312	CP035317
CP035318	CP035320	CP035321	CP035322	CP035325	CP035330
CP035331	CP035339	CP035350	CP035354	CP035360	CP035366
CP035377	CP035476	CP035477	CP035486	CP035498	CP035516
CP035545	CP035706	CP035720	CP035751	CP035767	CP035768
CP035770	CP035772	CP035812	CP035817	CP035819	CP035822
CP035832	CP035836	CP035841	CP035846	CP035850	CP035855
CP035860	CP035865	CP035870	CP035874	CP035879	CP035882
CP035884	CP035944	CP036177	CP036202	CP036245	CP037449
CP037857	CP037941	CP037943	CP037945	CP038138	CP038180
CP038282	CP038284	CP038287	CP038290	CP038292	CP038295
CP038300	CP038302	CP038305	CP038309	CP038313	CP038316
CP038319	CP038321	CP038324	CP038328	CP038333	CP038336
CP038339	CP038342	CP038344	CP038346	CP038349	CP038351
CP038353	CP038355	CP038357	CP038360	CP038363	CP038366
CP038369	CP038372	CP038374	CP038376	CP038380	CP038383
CP038386	CP038389	CP038394	CP038398	CP038402	CP038405
CP038408	CP038412	CP038414	CP038416	CP038419	CP038421
CP038423	CP038425	CP038428	CP038453	CP038494	CP038496
CP038505	CP038791	CP038857	CP038859	CP039298	CP039403
CP039404	CP039753	CP039834	CP039837	CP040067	CP040090
CP040107	CP040263	CP040269	CP040305	CP040307	CP040309
CP040311	CP040313	CP040314	CP040316	CP040381	CP040390
CP040397	CP040456	CP040570	CP040572	CP040643	CP040663
CP040664	CP040667	CP040886	CP040919	CP040927	CP041002
CP041031	CP041110	CP041284	CP041300	CP041302	CP041304
CP041337	CP041359	CP041392	CP041411	CP041416	CP041422
CP041425	CP041429	CP041431	CP041433	CP041435	CP041437
CP041442	CP041448	CP041452	CP041520	CP041522	CP041526
CP041529	CP041532	CP041535	CP041538	CP041541	CP041544
CP041546	CP041550	CP041556	CP041559	CP041563	CP041572
CP041578	CP041581	CP041623	CP041627	CP041628	CP041678
CP041747	CP041749	CP041919	CP041955	CP041996	CP042246
CP042250	CP042298	CP042336	CP042350	CP042470	CP042585
CP042588	CP042615	CP042845	CP042846	CP042865	CP042871
CP042878	CP042892	CP042893	CP042896	CP042899	CP042901
CP042903	CP042934	CP042969	CP042973	CP042981	CP042982
CP043033	CP043181	CP043183	CP043185	CP043187	CP043189
CP043191	CP043193	CP043195	CP043197	CP043199	CP043201
CP043203	CP043205	CP043207	CP043209	CP043211	CP043213
CP043217	CP043227	CP043406	CP043414	CP043478	CP043486
CP043487	CP043539	CP043541	CP043542	CP043733	CP043736
CP043739	CP043742	CP043744	CP043747	CP043750	CP043752
CP043754	CP043757	CP043852	CP043942	CP043946	CP043950
CP044021	CP044143	CP044145	CP044148	CP044291	CP044293
CP044305	CP044311	CP044312	CP044313	CP044314	CP044315
CP044346	CP044403	CP044410	CP045190	CP045206	CP045209
CP045213	CP045457	CP045712	CP045741	CP045827	CP045863
CP045975	CP045977	CP046000	CP046003	CP046006	CP046009
CP046025	CP046259	CP046396	CP046416	CP046527	CP046539
CP046716	CP047002	CP047010	CP047074	CP047076	CP047127
CP047231	CP047277	CP047378	CP047405	CP047455	CP047461
CP047571	CP047576	CP047594	CP047600	CP047609	CP047658
CP047662	CP047665	CP047710	CP047876	CP048010	CP048025
CP048107	CP048290	CP048304	CP048310	CP048322	CP048326
CP048330	CP048337	CP048344	CP048359	CP048367	CP048371
CP048376	CP048439	CP048603	CP048604	CP048605	CP048606
CP048647	CP048821	CP048853	CP048855	CP048857	CP048860
CP048863	CP048865	CP048867	CP048870	CP048873	CP048915
CP048920	CP048934	CP048935	CP049050	CP049077	CP049081
CP049085	CP049086	CP049101	CP049118	CP049197	CP049198
CP049201	CP049204	CP049299	CP049348	CP049353	CP049839
CP049845	CP049852	CP049936	CP049943	CP049967	CP049979

Continued on next page

CP050031	CP050036	CP050040	CP050044	CP050047	CP050173
CP050194	CP050196	CP050197	CP050199	CP050200	CP050201
CP050202	CP050203	CP050204	CP050205	CP050206	CP050207
CP050208	CP050209	CP050210	CP050211	CP050212	CP050213
CP050214	CP050216	CP050217	CP050218	CP050219	CP050220
CP050221	CP050222	CP050289	CP050382	CP050498	CP050646
CP050862	CP050865	CP050998	CP051001	CP051158	CP051219
CP051222	CP051263	CP051609	CP051615	CP051631	CP051688
CP051692	CP051694	CP051698	CP051700	CP051706	CP051711
CP051714	CP051716	CP051719	CP051725	CP051727	CP051733
CP051735	CP051738	CP051744	CP051749	CP051751	CP051753
CP051765	CP051776	CP051778	CP051782	CP051786	CP051790
CP051792	CP051796	CP051798	CP051800	CP051802	CP051804
CP051806	CP051808	CP051811	CP051814	CP051816	CP051819
CP051821	CP051824	CP051827	CP051830	CP051833	CP051837
CP051849	CP052057	CP052877	CP053079	CP053080	CP053211
CP053231	CP053234	CP053245	CP053247	CP053251	CP053258
CP053259	CP053281	CP053284	CP053296	CP053384	CP053600
CP053601	CP053602	CP053603	CP053604	CP053605	CP053607
CP053720	CP053723	CP053730	CP053731	CP053736	CP053785
CP053787	CP053851	CP053852	CP054214	CP054219	CP054224
CP054227	CP054230	CP054232	CP054236	CP054282	CP054314
CP054317	CP054325	CP054335	CP054343	CP054345	CP054353
CP054363	CP054368	CP054371	CP054372	CP054379	CP054388
CP054407	CP054556	CP054828	CP054940	CP055158	CP055251
CP055256	CP055259	CP055426	CP055438	CP055608	CP055624
CP055649	CP055652	CP055660	CP055669	CP055681	CP055713
CP055718	CP055724	CP055736	CP055748	CP055753	CP055786
CP055796	CP055818	CP055888	CP055918	CP055923	CP055934
CP055941	CP055945	CP055951	CP055958	CP055964	CP055967
CP055971	CP055973	CP055976	CP055981	CP056036	CP056073
CP056077	CP056190	CP056237	CP056242	CP056248	CP056249
CP056263	CP056273	CP056278	CP056282	CP056299	CP056304
CP056315	CP056348	CP056355	CP056357	CP056370	CP056392
CP056396	CP056404	CP056446	CP056470	CP056481	CP056551
CP056556	CP056564	CP056565	CP056569	CP056578	CP056584
CP056594	CP056598	CP056604	CP056608	CP056613	CP056618
CP056620	CP056625	CP056632	CP056642	CP056655	CP056662
CP056786	CP056794	CP056800	CP056810	CP056816	CP056821
CP056842	CP056846	CP056865	CP056867	CP056870	CP056873
CP056876	CP056894	CP056902	CP056914	CP056920	CP056921
CP056928	CP056933	CP056940	CP056947	CP056955	CP056974
CP056985	CP057007	CP057010	CP057012	CP057014	CP057016
CP057018	CP057020	CP057022	CP057024	CP057026	CP057028
CP057031	CP057038	CP057040	CP057045	CP057050	CP057055
CP057057	CP057062	CP057072	CP057075	CP057085	CP057087
CP057090	CP057109	CP057112	CP057115	CP057119	CP057123
CP057126	CP057129	CP057133	CP057137	CP057139	CP057141
CP057144	CP057152	CP057154	CP057160	CP057163	CP057166
CP057167	CP057171	CP057173	CP057175	CP057176	CP057178
CP057185	CP057186	CP057188	CP057190	CP057192	CP057194
CP057200	CP057204	CP057207	CP057223	CP057225	CP057234
CP057247	CP057250	CP057254	CP057259	CP057262	CP057267
CP057270	CP057273	CP057276	CP057281	CP057287	CP057293
CP057302	CP057334	CP057336	CP057340	CP057342	CP057344
CP057345	CP057347	CP057349	CP057354	CP057364	CP057368
CP057371	CP057378	CP057380	CP057396	CP057402	CP057408
CP057417	CP057420	CP057423	CP057429	CP057432	CP057435
CP057438	CP057441	CP057444	CP057447	CP057450	CP057453
CP057455	CP057465	CP057468	CP057470	CP057473	CP057475
CP057479	CP057484	CP057485	CP057487	CP057491	CP057493
CP057500	CP057507	CP057510	CP057512	CP057516	CP057520
CP057523	CP057525	CP057535	CP057539	CP057545	CP057555
CP057559	CP057570	CP057574	CP057577	CP057580	CP057582
CP057584	CP057587	CP057589	CP057594	CP057597	CP057600
CP057603	CP057605	CP057608	CP057612	CP057615	CP057617
CP057621	CP057626	CP057634	CP057636	CP057638	CP057640
CP057643	CP057645	CP057650	CP057660	CP057665	CP057669
CP057673	CP057677	CP057684	CP057686	CP057690	CP057693
CP057696	CP057705	CP057713	CP057717	CP057725	CP057729
CP057731	CP057738	CP057751	CP057759	CP057767	CP057773
CP057778	CP057784	CP057790	CP057796	CP057802	CP057804
CP057808	CP057812	CP057818	CP057826	CP057829	CP057831

Continued on next page

CP057833	CP057838	CP057839	CP057841	CP057843	CP057849
CP057854	CP057860	CP057861	CP057869	CP057873	CP057876
CP057878	CP057879	CP057882	CP057885	CP057888	CP057891
CP057893	CP057900	CP057901	CP057905	CP057906	CP057911
CP057915	CP057916	CP057922	CP057927	CP057930	CP057932
CP057936	CP057941	CP057942	CP057946	CP057951	CP057953
CP057957	CP057959	CP057961	CP057962	CP057964	CP057967
CP057970	CP057973	CP057975	CP057978	CP057980	CP057983
CP057987	CP057990	CP057993	CP057994	CP057995	CP057996
CP058004	CP058007	CP058009	CP058012	CP058014	CP058019
CP058023	CP058025	CP058027	CP058031	CP058063	CP058070
CP058072	CP058074	CP058076	CP058078	CP058080	CP058083
CP058087	CP058090	CP058093	CP058101	CP058104	CP058223
CP058302	CP058308	CP058342	CP058355	CP058571	CP058574
CP058596	CP058618	CP058651	CP058656	CP058661	CP058682
CP058913	CP058948	CP059003	CP059043	CP059119	CP059125
CP059130	CP059137	CP059279	CP059281	CP059283	CP059288
CP059723	CP059835	CP059904	CP059907	CP059913	CP059917
CP059922	CP059925	CP059929	CP059931	CP059944	CP059947
CP059953	CP059988	CP060057	CP060061	CP060065	CP060073
CP060075	CP060083	CP060121	CP060708	CP060709	CP061101
CP061108	CP061185	CP061206	CP061329	CP061337	CP061339
CP061530	CP061749	CP061754	CP061758	CP061764	CP061914
CP062160	CP062203	CP062204	CP062211	CP062228	CP062239
CP062243	CP062700	CP062702	CP062705	CP062708	CP062711
CP062713	CP062715	CP062717	CP062719	CP062721	CP062723
CP062725	CP062727	CP062729	CP062731	CP062733	CP062736
CP062739	CP062742	CP062744	CP062746	CP062749	CP062752
CP062755	CP062758	CP062761	CP062763	CP062766	CP062769
CP062771	CP062774	CP062778	CP062780	CP062782	CP062811
CP062815	CP062824	CP062826	CP062832	CP062838	CP062844
CP062851	CP062853	CP062855	CP062863	CP062865	CP062868
CP062871	CP062876	CP062883	CP062889	CP062895	CP062901
CP062908	CP062910	CP062924	CP062927	CP062930	CP062933
CP062967	CP062970	CP063046	CP063153	CP063214	CP063332
CP063341	CP063349	CP063369	CP063459	CP063473	CP063476
CP063479	CP063483	CP063487	CP063492	CP063499	CP063503
CP063511	CP063515	CP063518	CP063711	CP063713	CP063720
CP063729	CP063736	CP063739	CP063742	CP063774	CP063956
CP063958	CP063969	CP063970	CP063979	CP063983	CP064103
CP064167	CP064677	CP064678	CP064679	CP064680	CP064681
CP064682	CP064683	CP064847	CP065136	CP065152	CP065607
CP065611	CP065613	CP065615	CP065619	CP065624	CP065970
CP066032	CP066144	CP066366	CP066748	CP066753	CP066756
CP066759	CP066806	CP066836	CP066844	CP067232	CP067235
CP067241	CP067245	CP067250	CP067265	CP067274	CP067299
CP067303	CP067307	CP067311	CP067342	CP067399	CP067994
CP068035	CP068041	CP068155	CP068279	CP068281	CP068283
CP068394	CP068591	CP068706	CP068795	CP068796	CP068799
CP068800	CP068801	CP068802	CP068803	CP068804	CP068805
CP068806	CP068807	CP068808	CP068809	CP068810	CP068811
CP068812	CP068813	CP068814	CP068815	CP068816	CP068817
CP068818	CP068819	CP068820	CP068821	CP068822	CP068823
CP068824	CP068825	CP068826	CP068827	CP068828	CP068829
CP068830	CP068995	CP069132	CP069133	CP069134	CP069386
CP069438	CP069441	CP069445	CP069447	CP069451	CP069453
CP069459	CP069480	CP069488	CP069500	CP069501	CP069517
CP069522	CP069528	CP069542	CP069549	CP069559	CP069571
CP069583	CP069589	CP069646	CP069657	CP069666	CP069677
CP069682	CP069692	CP069706	CP069707	CP069709	CP069710
CP069712	CP069865	CP069882	CP069890	CP069893	CP069945
CP069957	CP069973	CP069978	CP069980	CP069990	CP069996
CP070007	CP070026	CP070041	CP070045	CP070048	CP070063
CP070066	CP070071	CP070077	CP070098	CP070103	CP070111
CP070115	CP070124	CP070134	CP070148	CP070152	CP070157
CP070162	CP070170	CP070227	CP070232	CP070279	CP070280
CP070393	CP070399	CP070403	CP070593	CP070902	CP070906
CP070914	CP070920	CP070930	CP071036	CP071050	CP071073
CP071133	CP071375	CP071436	CP071439	CP071441	CP071521
CP071522	CP071954	CP072054	CP072204	CP072322	CP072539
CP072661	CP072662	CP072663	CP072784	CP072785	CP072786
CP072787	CP072802	CP072858	CP072911	CP072976	CP072980
CP072986	CP073360	CP073591	CP073593	CP073595	CP073597

Continued on next page

CP073599	CP073601	CP073603	CP073607	CP073609	CP073613
CP073617	CP073619	CP073621	CP073624	CP073718	CP073719
CP073768	CP074120	CP074576	CP074701	CP075061	CP075062
CP075374	CP075462	CP075465	CP075469	CP075627	CP075632
CP075640	CP075649	CP075652	CP075663	CP075665	CP075697
CP075852	CP076123	CP076230	CP076232	CP076235	CP076237
CP076241	CP076243	CP076245	CP076293	CP076301	CP076305
CP076318	CP076344	CP076404	CP076527	CP076645	CP076646
CP076654	CP076687	CP076689	CP076693	CP076697	CP076704
CP076706	CP076709	CP076714	CP077063	CP077215	CP077225
CP077243	CP077250	CP077266	CP077273	CP077281	CP077294
CP077314	CP077325	CP077342	CP077353	CP077361	CP077379
CP077389	CP077396	CP077646	CP077649	CP078137	CP078555
CP078556	CP078557	CP078565	CP078566	CP078567	CP078568
CP078570	CP078571	CP078573	CP078575	CP078577	CP078578
CP078580	CP078581	CP078582	CP078585	CP078587	CP078588
CP078589	CP078590	CP078591	CP078592	CP079735	CP079736
CP079747	CP080001	CP080116	CP080117	CP080118	CP080119
CP080120	CP080121	CP080126	CP080133	CP080139	CP080146
CP080155	CP080163	CP080172	CP080223	CP080233	CP080235
CP080246	CP080253	CP080255	CP080260	CP080370	CP080620
CP080645	CP081007	CP081008	CP081009	CP081010	CP081192
CP081306	CP081677	CP081684	CP081690	CP081698	CP081701
CP081706	CP081711	CP081713	CP081719	CP081724	CP081729
CP081736	CP081742	CP081748	CP081755	CP081762	CP081767
CP081774	CP081782	CP081791	CP081796	CP081805	CP081881
CP082120	CP082127	CP082129	CP082349	CP082355	CP082357
CP082824	CP082827	CP082831	CP082949	CP083263	CP083273
CP083410	CP083419	CP083478	CP083481	CP083492	CP083497
CP083503	CP083512	CP083517	CP083530	CP083534	CP083585
CP083701	CP083869	CP083872	CP083877	CP083880	CP083886
CP083888	CP084026	CP084096	CP084529	CP084534	CP084893
CP084894	CP084895	CP084896	CP084897	CP084898	CP084899
CP084900	CP085056	CP085060	CP085063	CP085069	CP085074
CP085183	CP085188	CP085372	CP085488	CP085517	CP085619
CP085622	CP085637	CP085638	CP085842	CP086259	CP086334
CP086336	CP086338	CP086340	CP086342	CP086391	CP086556
CP086616	CP086618	CP086620	CP086622	CP086623	CP086624
CP086625	CP086626	CP086627	CP086662	CP087110	CP087377
CP087570	CP087578	CP087958	CP088026	CP088028	CP088030
CP088032	CP088034	CP088036	CP088038	CP088040	CP088042
CP088044	CP088046	CP088048	CP088050	CP088052	CP088054
CP088056	CP088058	CP088060	CP088062	CP088064	CP088067
CP088069	CP088071	CP088344	CP088346	CP088348	CP088351
CP088352	CP088356	CP088363	CP088366	CP088369	CP088370
CP088374	CP088381	CP088383	CP088387	CP088390	CP088393
CP088397	CP088400	CP088406	CP088407	CP088408	CP088413
CP088414	CP088417	CP088421	CP088427	CP088433	CP088436
CP088443	CP088451	CP088452	CP088456	CP088461	CP088463
CP088466	CP088468	CP088473	CP088474	CP088479	CP088485
CP088486	CP088490	CP088494	CP088496	CP088500	CP088506
CP088509	CP088512	CP088514	CP088516	CP088520	CP088526
CP088529	CP088534	CP088542	CP088545	CP088547	CP088553
CP088555	CP088560	CP088564	CP088566	CP088576	CP088578
CP088580	CP088582	CP088584	CP088592	CP088593	CP088596
CP088598	CP088602	CP088607	CP088609	CP088612	CP088613
CP088617	CP088621	CP088624	CP088627	CP088629	CP088632
CP088637	CP088641	CP088643	CP088645	CP088649	CP088652
CP088654	CP088656	CP088658	CP088660	CP088662	CP088665
CP088673	CP088677	CP088681	CP088683	CP088687	CP088692
CP088694	CP088698	CP088701	CP088703	CP088707	CP088710
CP088714	CP088719	CP088722	CP088723	CP088725	CP088729
CP088734	CP088737	CP088740	CP088744	CP088754	CP088760
CP088764	CP088768	CP088771	CP088773	CP088776	CP088779
CP088782	CP088786	CP088790	CP088795	CP088800	CP088802
CP088804	CP088808	CP088812	CP088818	CP088820	CP088822
CP088826	CP088828	CP088829	CP088833	CP088835	CP088837
CP088839	CP088850	CP088851	CP088855	CP088860	CP088864
CP088866	CP088869	CP088872	CP089031	CP089032	CP089034
CP089036	CP089142	CP089270	CP089272	CP089445	GL878028
GL896790	GL988050	HF572917	HG428755	HG994851	JAADCD010000001
JAAJTL010000001	JAAJTN010000001	JAAJTO010000001	JAAJTP010000001	JAAJTQ010000001	JAAJTR010000001
JAAJTS010000001	JAAJTT010000001	JAAJTU010000001	JAAJTV010000001	JAAKGF010000001	JAALAC010000002

Continued on next page

JAANTD010000001	JAATHU010000001	JAATHV010000001	JAATHX010000001	JAATHY010000001	JABWPS010000001
JABXOE010000001	JACDQE010000001	JACDQF010000001	JACDQG010000001	JACGZL020000001	JACGZP020000001
JACGZQ020000001	JACGZT020000001	JACIVJ010000001	JACIVK010000001	JACOJH010000001	JACOLT010000001
JACOLU010000001	JACRQJ010000001	JACSXT010000001	JACXXS010000001	JACXXV010000001	JACXYM010000001
JACYGB010000002	JACYGC010000008	JACYGD010000009	JACYGE010000009	JACYGN010000008	JADALR010000001
JADALV010000001	JADALW010000001	JADDLK010000001	JADDLL010000001	JADDLM010000001	JADDLP010000001
JADDMK010000001	JADDML010000001	JAEFCJ010000001	JAEFCL010000001	JAEFCM010000001	JAEFCO010000001
JAEFCP010000001	JAEFCQ010000001	JAEPRM010000001	JAFBIE010000001	JAFBIF010000001	JAFCXR010000001
JAFFIC010000001	JAGEVC010000001	JAGFYA010000002	JAGFYC010000001	JAGFYF010000001	JAGFZN010000001
JAGFZR010000001	JAGFZU010000001	JAGHKJ010000001	JAHMAB010000001	JAHQKD010000001	JAHQKR010000001
JAHQKS010000001	JAHQLA010000001	JAHQLB010000001	JAHSRN010000001	JAHSRO010000001	JAHSRP010000001
JAHSRQ010000001	JAHSRR010000001	JAHSRS010000001	JAHSRT010000001	JAHSRU010000001	JAHSRV010000001
JAHSRW010000001	JAHSRX010000001	JAHSRY010000001	JAHSRZ010000001	JAHSSA010000001	JAHSSB010000001
JAHSSC010000001	JAHSSD010000001	JAHSSE010000001	JAINSH010000001	JAINTH010000001	JAJBZO010000001
JAJBZP010000001	JAJJWB010000001	JAJJWE010000001	JAJJWF010000001	JASQ01000001	JASU01000001
JH648165	JJMY02000001	JMJD01000003	KB732454	KB732530	KB732535
KB732574	KB732580	KB732854	KB732889	KB732931	KB732993
KB733023	KB733040	KB733069	KB733071	KB733094	KB733111
KB733130	KB733135	KB733141	KB733164	KB733172	KB733173
KB733185	KB733235	KB733361	KB733365	KE136980	KE137028
KE137162	KE137211	KE700100	KI929682	KI929782	KI929787
KK583188	KQ087662	LM993812	LM995446	LN832404	LN874954
LN877770	LORP02000005	LORS02000001	LPWC02000002	LQUJ02000001	LR025096
LR025101	LR130532	LR130545	LR130555	LR130562	LR130564
LR134000	LR134031	LR134075	LR134079	LR134080	LR134216
LR134226	LR134227	LR134228	LR134231	LR134239	LR134247
LR134296	LR217818	LR536430	LR595691	LR607328	LR607331
LR740758	LR778141	LR778143	LR778146	LR778147	LR778149
LR778150	LR778151	LR778152	LR778153	LR782232	LR881940
LR882050	LR882052	LR882057	LR882493	LR882973	LR882978
LR882990	LR882997	LR883006	LR883012	LR883050	LR883965
LR890198	LR890270	LR890288	LR890299	LR890326	LR890334
LR890349	LR890410	LR890508	LR890536	LR890603	LR890651
LR890693	LR890714	LR898868	LR898874	LRDE01000001	LRDF01000001
LS483296	LS992166	LS992168	LS992171	LS992180	LS992185
LS992190	LS992192	LS998785	LS999560	LSUP01000001	LT594504
LT601384	LT615372	LT615377	LT632320	LT827011	LT883142
LT906474	LUYD01000001	LVRH02000001	LVSP02000001	LWDC01000001	MBNW01000001
MBNX01000003	MBNY01000001	MCRE01000001	MDLJ02000001	MIIL01000001	MPGR01000001
MPGS01000001	MPGT01000001	MRDN01000001	NBJI01000001	NC_000913	NC_002655
NC_002695	NC_004431	NC_008253	NC_008563	NC_009801	NC_010468
NC_010473	NC_011353	NC_011741	NC_011742	NC_011745	NC_011748
NC_011750	NC_011751	NC_012759	NC_013353	NC_013361	NC_013364
NC_013941	NC_017625	NC_017628	NC_017631	NC_017633	NC_017651
NC_017656	NC_017660	NC_017906	NC_018661	NC_020163	NC_022648
NGSF01000001	NHYO01000001	NHYP01000001	NHYQ01000001	NHYR01000001	NHYS01000001
NIFW01000002	NQCO02000001	NQCP02000001	NQCQ02000001	NQCR02000001	NQLI02000001
NWVR01000001	NXER01000001	OU342919	OU349837	OU349838	OU349839
OU349840	OU349841	OU349842	OU349843	OU349844	OU349845
OU349846	OU349848	OU349849	OU356913	PDDT01000002	PDED01000001
PDEK01000002	PDLD01000001	PDLF01000002	PDLJ01000002	PSQV01000007	PSQW01000002
PSQX01000006	PVMG01000001	QBDM01000001	QDAJ01000001	QLVH01000001	QMGK01000003
QMGM01000001	QSMQ02000006	QSMR02000002	QSMV02000001	QWLO01000001	QXTI01000001
RKJE01000005	RKJJ01000004	RKJK01000003	RKJN01000003	RRCS01000001	RYCV01000003
SESS01000001	SEVB01000002	SEVD01000001	SEVE01000003	SEVF01000002	SEVI01000004
SEVL01000002	SEVM01000001	SEVN01000002	SEVO01000002	SEVP01000003	SEVQ01000001
SEVS01000006	SEVT01000005	SEVU01000007	SEVW01000003	SEVX01000001	SEVY01000001
SEWA01000004	SGYH01000001	SSWI01000001	U00096	UEFM01000001	UEFR01000001
UFYE01000002	UFZD01000005	UFZE01000001	UFZG01000001	UFZI01000001	UFZT01000001
UFZW01000006	UGAD01000001	UGAE01000003	UGAH01000002	UGAK01000003	UGAL01000001
UGAM01000005	UGAO01000001	UGAS01000002	UGAT01000002	UGAU01000003	UGAV01000006
UGBA01000002	UGBB01000002	UGBE01000003	UGBF01000004	UGBJ01000001	UGBK01000002
UGBM01000005	UGBP01000004	UGBV01000003	UGBX01000001	UGCB01000003	UGCH01000001
UGCI01000003	UGCT01000004	UGCW01000003	UGCY01000004	UGCZ01000001	UGDG01000002
UGDH01000001	UGDO01000002	UGDR01000001	UGDU01000003	UGDW01000001	UGDY01000003
UGEC01000002	UGEE01000003	UGEF01000002	UGEJ01000002	UGEL01000003	UGEO01000001
UGES01000005	UGEU01000002	UGEV01000002	UGEZ01000005	UGFB01000004	UGFD01000001
UGFL01000003	UGFO01000006	UGFS01000002	UGFT01000003	UGFU01000001	UGFV01000001
UGGG01000003	UGGI01000001	UWUZ01000001	UWVA01000001	UWVG01000001	UWVZ01000001
UWWG01000001	UWXG01000001	UWXK01000001	UWYG01000001	VCAQ01000001	VCYZ01000001
VCZJ01000001	VCZX01000001	VDBC01000001	VDBD01000001	VDBH01000001	VFAG01000002
VMRI01000001	WIPD02000001	WMHS01000001	WMLE01000001	WNKN01000001	WNKO01000001
WSNS01000001	WXYW01000001				

Table S11. Manual curation steps applied to *E. coli* housekeeping gene sequence collections

adk	Removed because of frameshift mutations: CP055158, CP059988, CP082330.<br>Edited to remove extra nucleotides at 5′ end: CP003034, BA000007, AP018488, NC_002695.
icd	Removed because of frameshift mutations: CP054325, CP054335, CP054457, CP059988, CP063983, CP073609, CP082330, CP025520, CP035841.<br>Removed owing to poor sequence identity (likely wrong gene labelled in database): LN832404, LT615379, LT615374, LT615372.
fumC	Removed because of frameshift mutations: CP054317, CP054319, CP055518, CP048304, CABWHO010000003, UFZW01000006, CP023749, CP027340, SEVU01000007, CP005930, CP006698, CP085517, CP058571, CP077063, CP054457, CP054328, CP054325, CP042627, CP042638, CP042606, CADDWW010000001.<br>Removed as 5′ region not sequenced: CP054314, HE572566, UGFP01000001.<br>Removed owing to poor sequence identity (likely wrong gene labelled in database): UGEE01000003.
recA	Removed because of frameshift mutations: CP042606, AP054335, CP054457, CP059922, CP082330, CNFQ01000001, CP059281.<br>Removed as 3′ region not sequenced: CP054325.
mdh	Removed because of frameshift mutations: VMRI01000001, CP042599, CP042638, CP042627, CP042645, CP042615, CP042982, CP054314, CP054828, CP059925, CP048935, CP077063, CP082330.<br>Removed as 5′ region not sequenced: CP044298.
gyrB	Removed because of frameshift mutations: CP054457, CP082330, NHYO01000001, CP029240, CP088534, CP048935, CP042606, CP042599, CP042638, CP042627, CP042645, CP042615, CP055158, CP051692, CP054828, CP046676.<br>Removed as 5′ region not sequenced: AP024561, CP050214, CP050216, CP050208.<br>Removed as 3′ region not sequenced: CP050194.<br>Edited to remove extra nucleotides at 5′ end: AP025214, CP011342, CP011343, AP024126.<br>Edited by adding gaps to align sequence: CP025703.
purA	Removed because of frameshift mutations: CP042599, CP042638, CP042645, CP054449, CP054454, CP046676, CP082330, CADDWT010000001, CP054457, CP054319, CP044298, CP042627, CP042626.<br>Removed as 5′ region not sequenced: CP088551, CP088570.<br>Removed as sequence contains premature termination codon: CP054328.

Table S12. GenBank accession numbers of sequences used in the SARS-CoV analysis.

AP006557.1	AP006558.1	AP006559.1	AP006560.1	AP006561.1	AY274119.3	AY278487.3	AY278488.2	AY278489.2	AY278490.3
AY278491.2	AY278554.2	AY278741.1	AY279354.2	AY282752.2	AY283794.1	AY283795.1	AY283796.1	AY283797.1	AY283798.2
AY291315.1	AY291451.1	AY297028.1	AY304495.1	AY310120.1	AY313906.1	AY321118.1	AY323977.2	AY338174.1	AY338175.1
AY345986.1	AY345987.1	AY345988.1	AY348314.1	AY350750.1	AY351680.1	AY357075.1	AY357076.1	AY362698.1	AY362699.1
AY390556.1	AY394850.2	AY394977.1	AY394978.1	AY394979.1	AY394980.1	AY394981.1	AY394982.1	AY394983.1	AY394984.1
AY394985.1	AY394986.1	AY394987.1	AY394988.1	AY394989.1	AY394990.1	AY394991.1	AY394992.1	AY394993.1	AY394994.1
AY394995.1	AY394996.1	AY394997.1	AY394998.1	AY394999.1	AY395000.1	AY395001.1	AY395002.1	AY395003.1	AY395004.1
AY427439.1	AY461660.1	AY463059.1	AY463060.1	AY485277.1	AY485278.1	AY502923.1	AY502924.1	AY502925.1	AY502926.1
AY502927.1	AY502928.1	AY502929.1	AY502930.1	AY502931.1	AY502932.1	AY508724.1	AY559083.1	AY559084.1	AY559087.1
AY568539.1	AY595412.1	AY613947.1	AY686864.1	AY714217.1	AY772062.1	AY864805.1	AY864806.1	DQ182595.1	DQ640652.1
DQ898174.1	EU371559.1	EU371560.1	EU371561.1	EU371562.1	EU371563.1	EU371564.1	FJ882926.1	FJ882930.1	FJ882931.1
FJ882938.1	FJ882963.1	GU553363.1	GU553364.1	JF292921.1	JF292922.1	JN854286.1	JQ316196.1	JX162087.1	

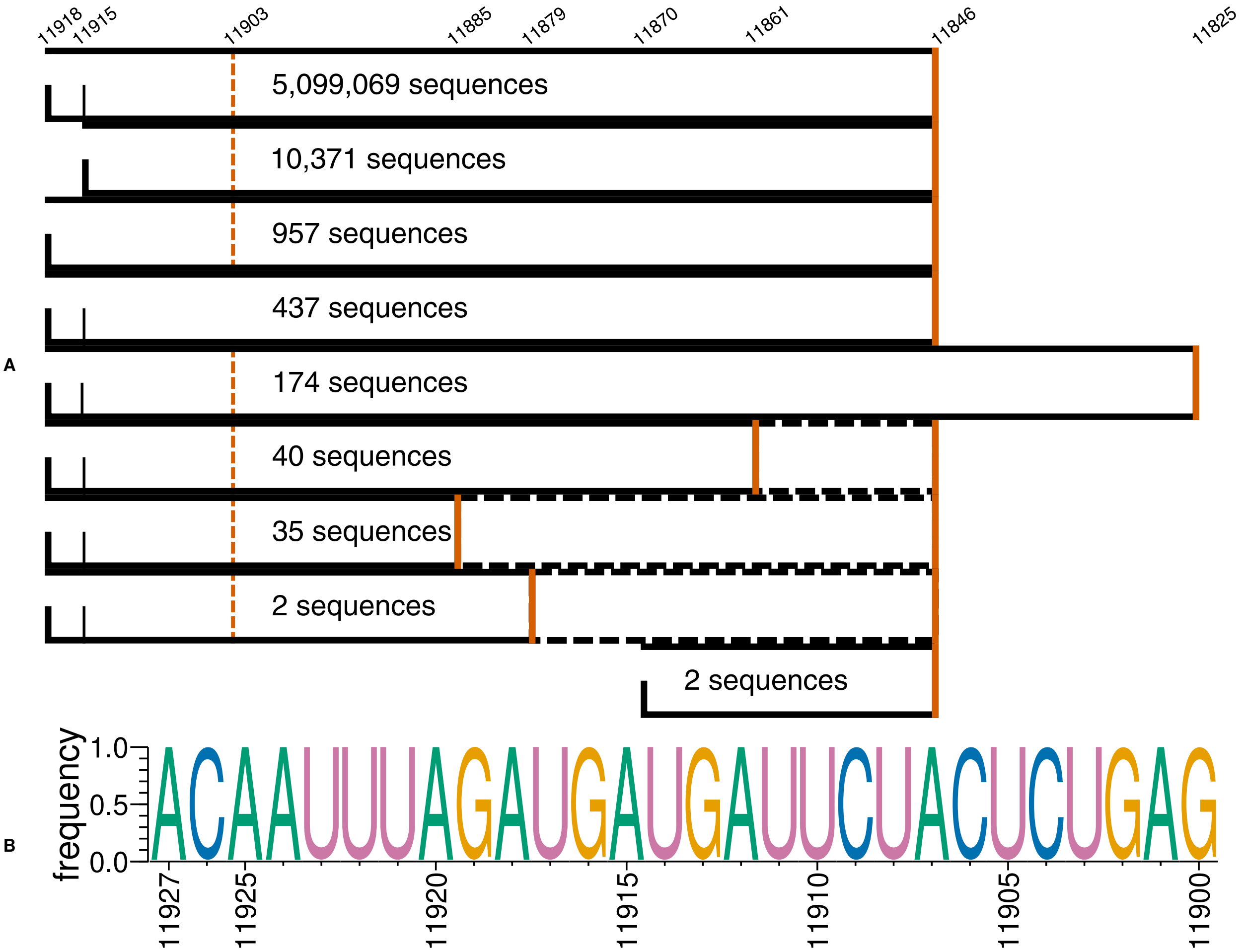

Fig. S9. (A) Schematic of the open reading frame potential of the first region of interest identified within the nsp7 region. Nucleotide references for the $5'$ end of codons in frame for the putative ORF are given at the top of the schematic. Black vertical half-bars represent postulated initiation codons. Orange vertical bars represent termination codons, with the termination codon that would require translational readthrough marked with a dashed line. Regions following apparent premature termination codons are outlined with dashed lines. There is commonly a termination codon in the putative +5 codon position, so that translational readthrough would almost always be required. If such readthrough is possible, then by the crude measures of the presence of initiation and termination codons, almost all sequences contain candidates for this putative ORF. **(B)** Sequence of the putative initiation region and early termination codon in the region of interest, generated using WebLogo3 (25). Although the sequence is highly conserved, it does not yield a strong Kozak sequence and would require translational readthrough of the early termination codon.

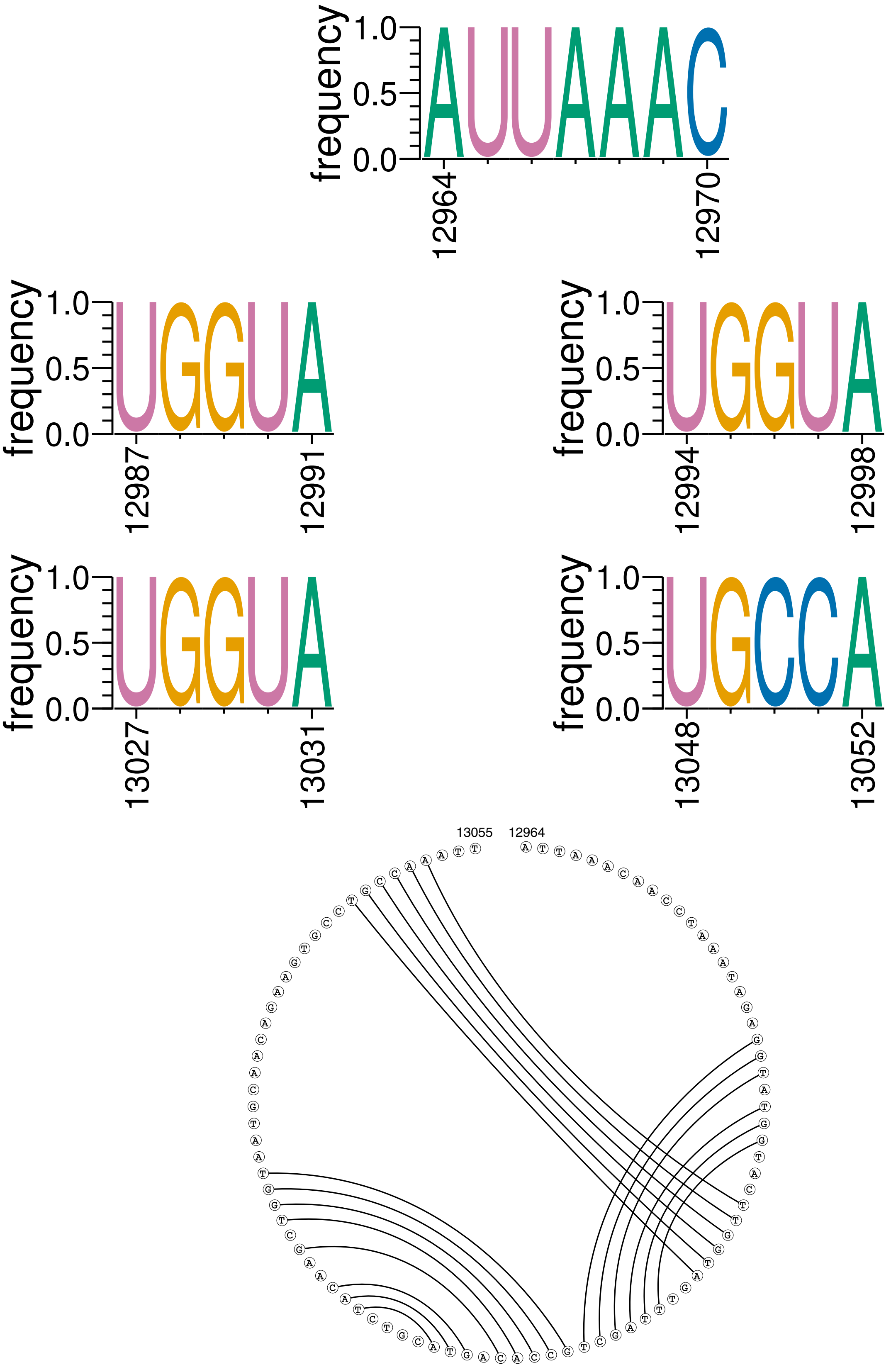

Fig. S10. Top rows: nucleotide frequencies in our dataset for the putative slippery site for a −1 frameshift within the nsp9 region, and for possible pseudoknot motifs, generated using WebLogo3 (25). Bottom: possible secondary structure consisting of two stem-loops with a pseudoknot, drawn in circularised form using the RNAstructure package (26). Note that this particular structure is generated by manual inspection, rather than running free-energy minimisation algorithms.

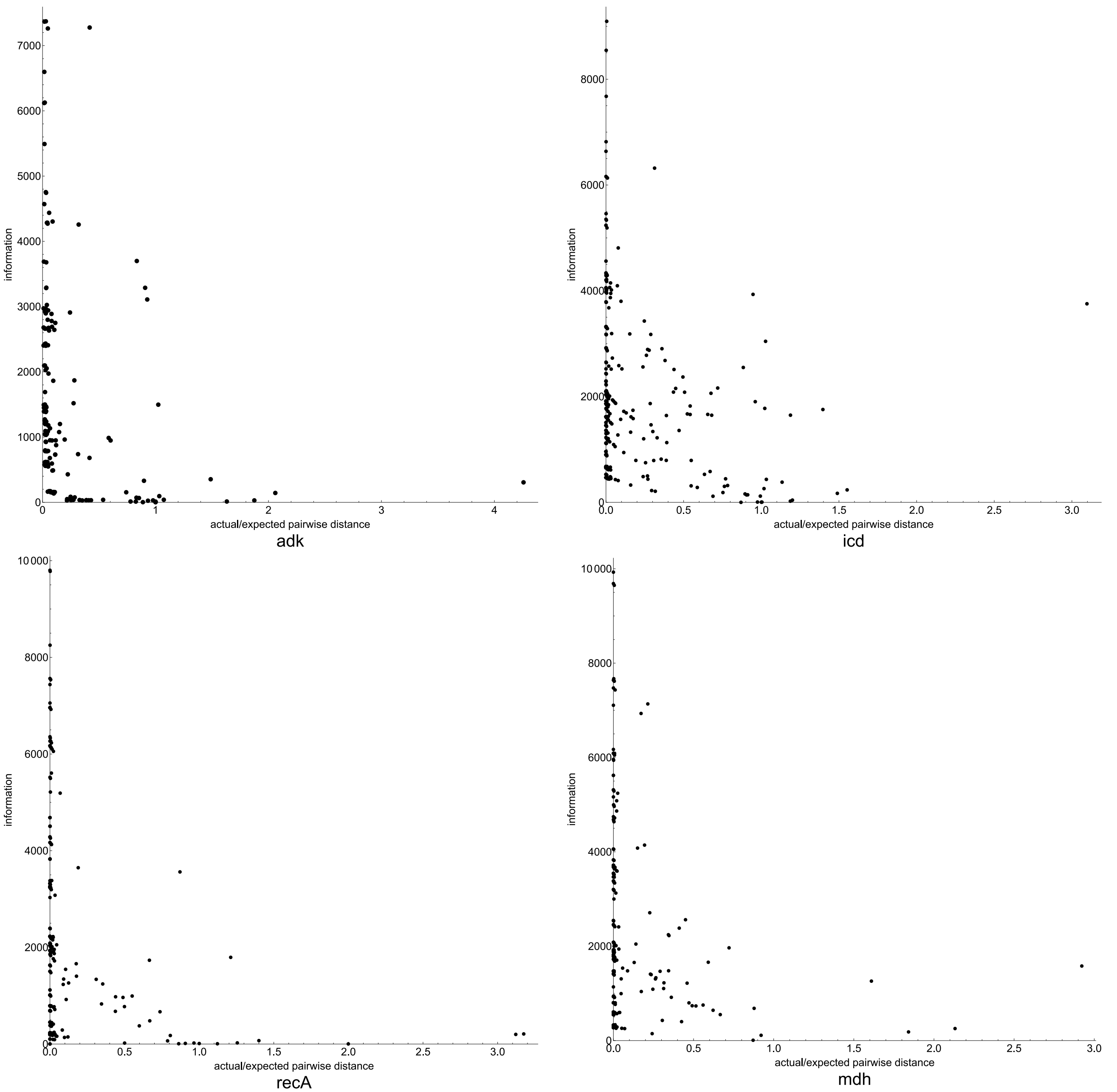

Fig. S11. Shannon information at locus versus measures of variability (actual/expected pairwise distance: see Methods for details) for housekeeping genes adk, icd, recA and mdh of *E. coli*. The plot for gyrB may be found in the main text. Throughout this work, each locus is a codon within a gene, although in principle different loci could be used with different measures of variability. Each plotted point represents the comparison of information/variability at a single locus. In each case, most loci have low variability, although the information gained from each low variability locus can vary. High variability loci are rare and many give low information. The absence of high information, high variability loci reflects the high number of combinations of loci producing high variability, meaning the Shannon information cannot be high.

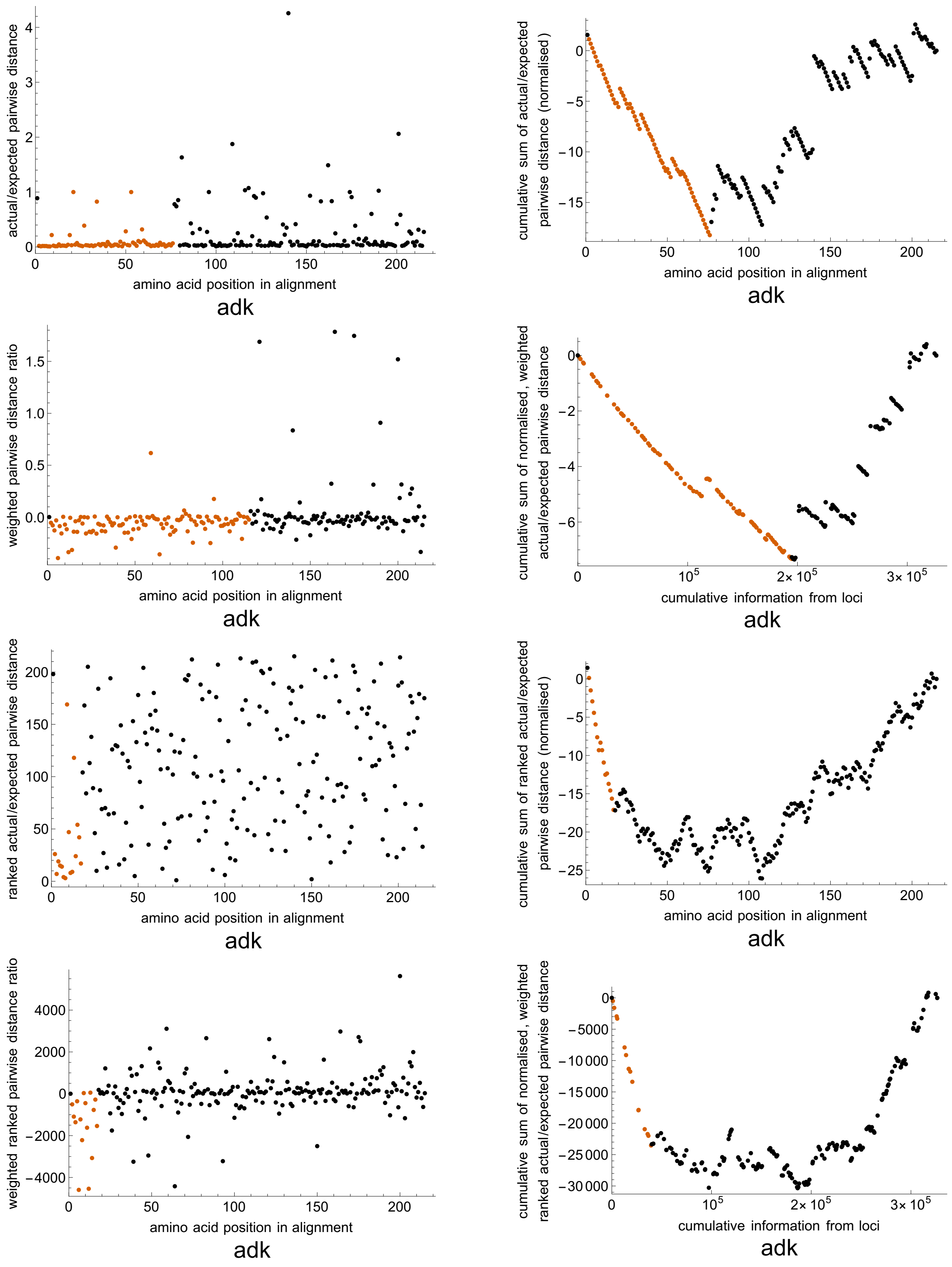

Fig. S12. Output from analyses of *E. coli* adk gene, with raw analysis output on left and cumulative sums on right. Each dot represents one codon. In each case, regions deemed significant by the algorithm are highlighted in orange and regions deemed significant after removal of a potentially interfering region are highlighted in blue. Codons contributing no information to the analysis are in a lighter colour and plotted with open triangles. Top row: using raw (unweighted/unranked) pairwise distance ratios. Second row: using weighted, unranked ratios. Third row: using unweighted, ranked ratios. Bottom row: using weighted, ranked ratios.

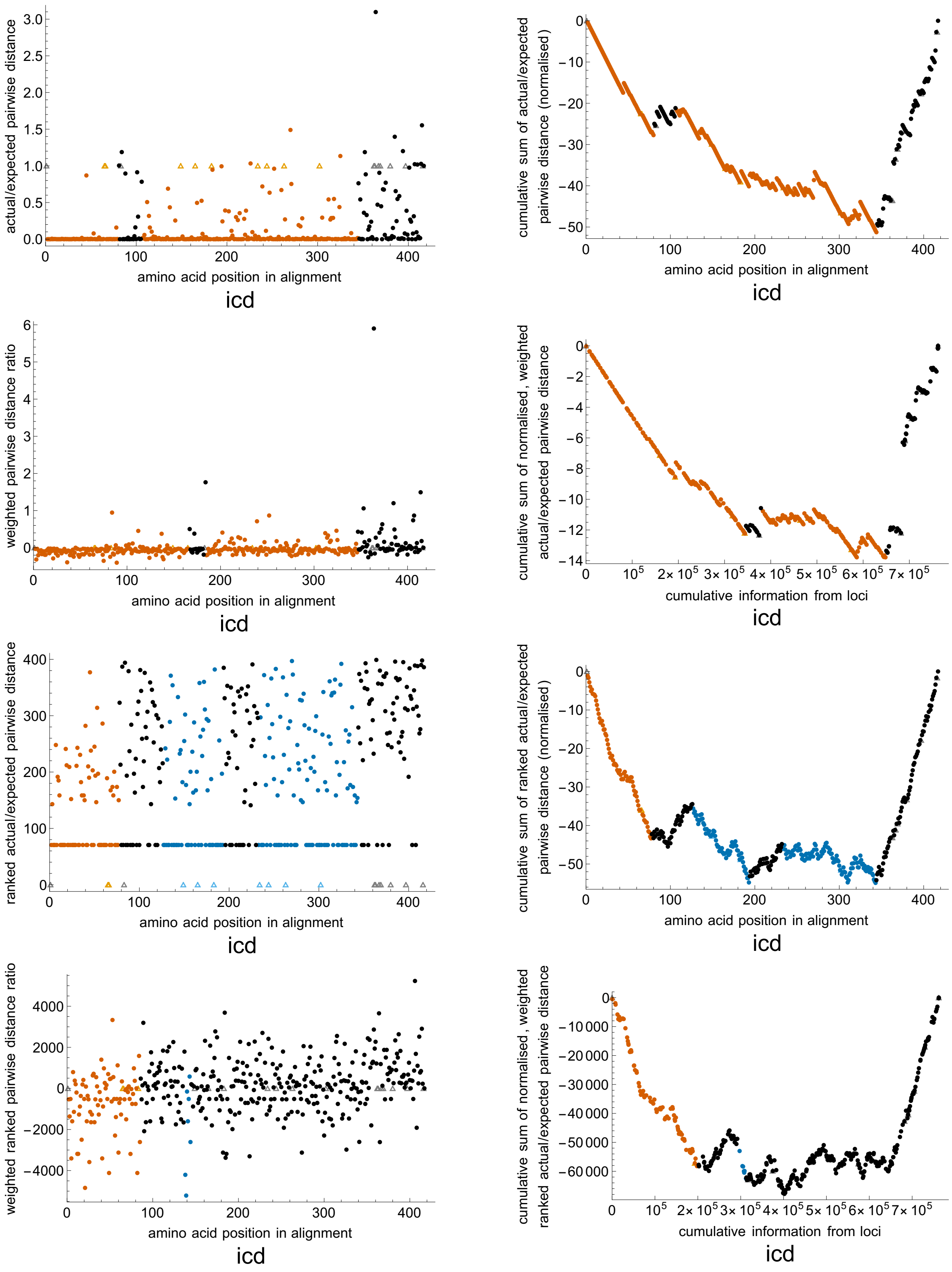

Fig. S13. Output from analyses of *E. coli* icd gene, with raw analysis output on left and cumulative sums on right. Each dot represents one codon. In each case, regions deemed significant by the algorithm are highlighted in orange and regions deemed significant after removal of a potentially interfering region are highlighted in blue. Codons contributing no information to the analysis are in a lighter colour and plotted with open triangles. Top row: using raw (unweighted/unranked) pairwise distance ratios. Second row: using weighted, unranked ratios. Third row: using unweighted, ranked ratios. Bottom row: using weighted, ranked ratios.

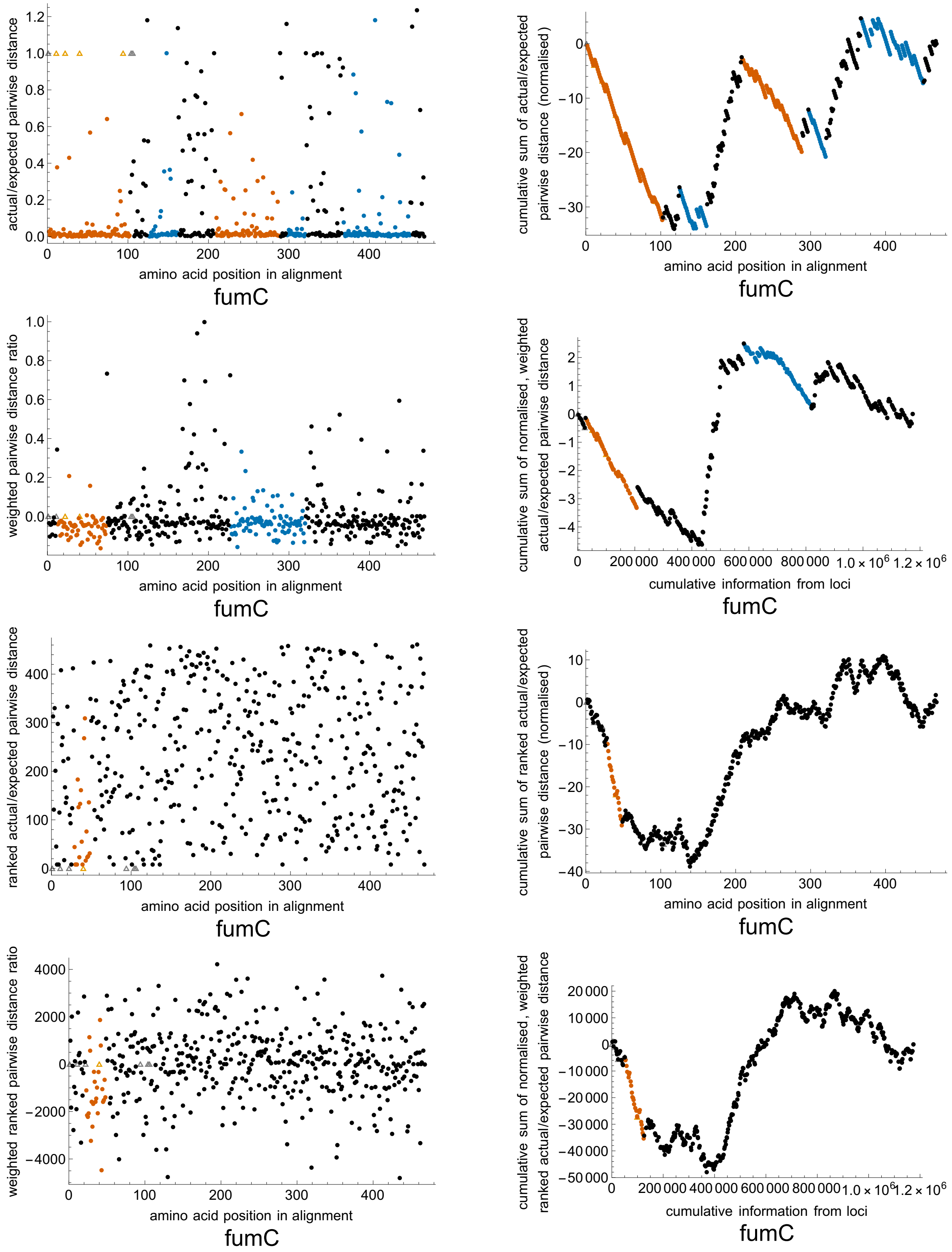

Fig. S14. Output from analyses of *E. coli* fumC gene, with raw analysis output on left and cumulative sums on right. Each dot represents one codon. In each case, regions deemed significant by the algorithm are highlighted in orange and regions deemed significant after removal of a potentially interfering region are highlighted in blue. Codons contributing no information to the analysis are in a lighter colour and plotted with open triangles. Top row: using raw (unweighted/unranked) pairwise distance ratios. Second row: using weighted, unranked ratios. Third row: using unweighted, ranked ratios. Bottom row: using weighted, ranked ratios.

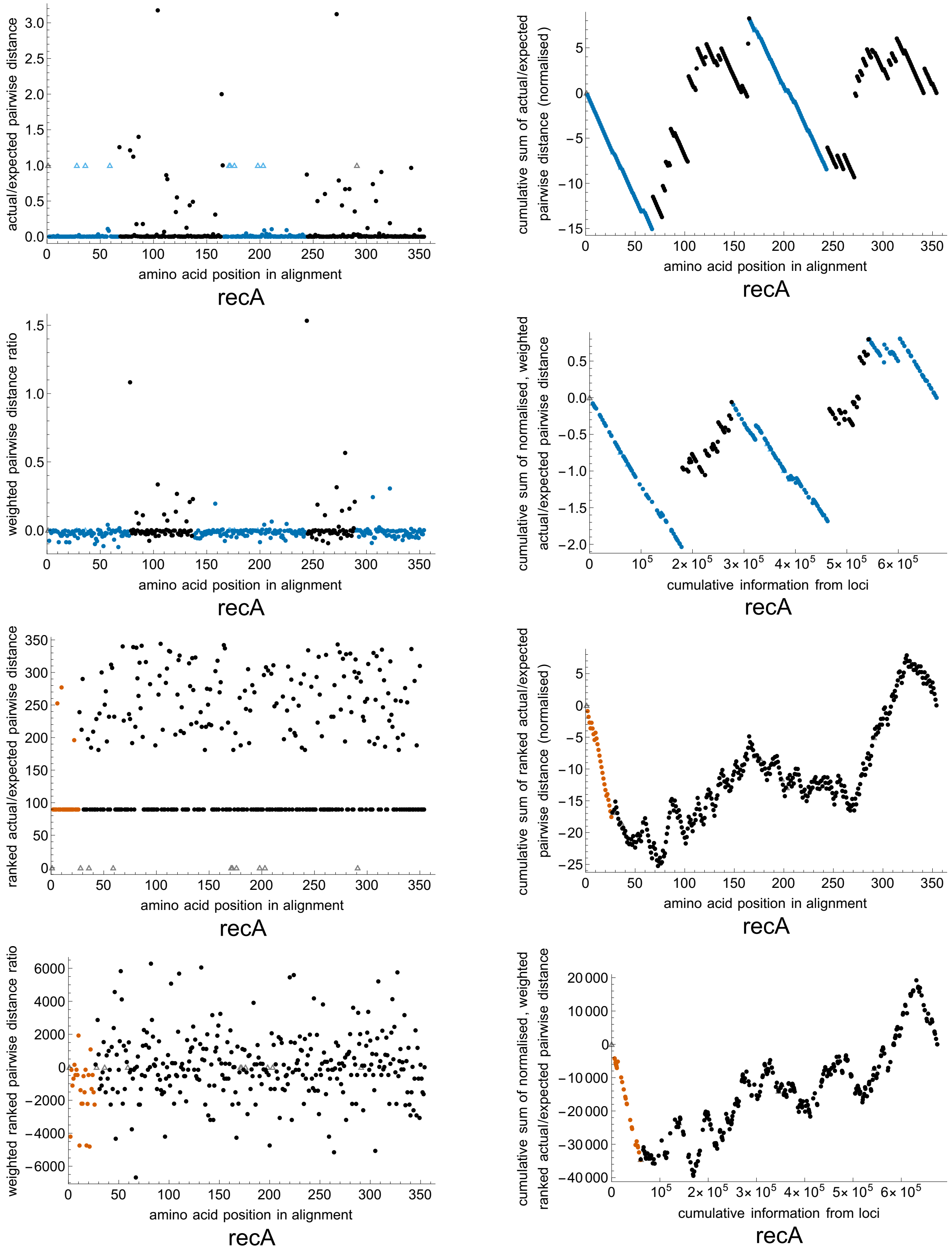

Fig. S15. Output from analyses of *E. coli* recA gene, with raw analysis output on left and cumulative sums on right. Each dot represents one codon. In each case, regions deemed significant by the algorithm are highlighted in orange and regions deemed significant after removal of a potentially interfering region are highlighted in blue. Codons contributing no information to the analysis are in a lighter colour and plotted with open triangles. Top row: using raw (unweighted/unranked) pairwise distance ratios. Second row: using weighted, unranked ratios. Third row: using unweighted, ranked ratios. Bottom row: using weighted, ranked ratios.

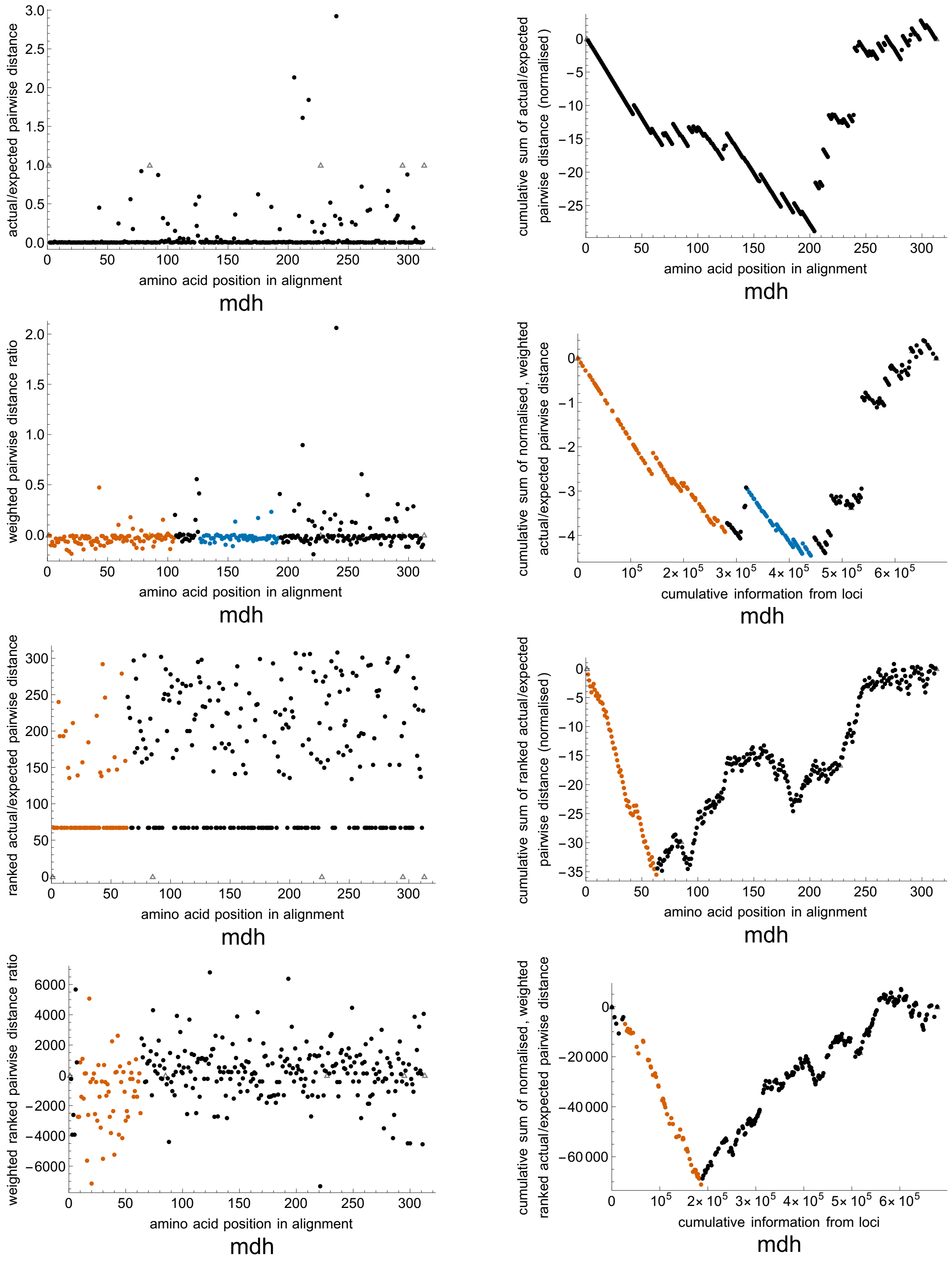

Fig. S16. Output from analyses of *E. coli* mdh gene, with raw analysis output on left and cumulative sums on right. Each dot represents one codon. In each case, regions deemed significant by the algorithm are highlighted in orange and regions deemed significant after removal of a potentially interfering region are highlighted in blue. Codons contributing no information to the analysis are in a lighter colour and plotted with open triangles. Top row: using raw (unweighted/unranked) pairwise distance ratios. Second row: using weighted, unranked ratios. Third row: using unweighted, ranked ratios. Bottom row: using weighted, ranked ratios.

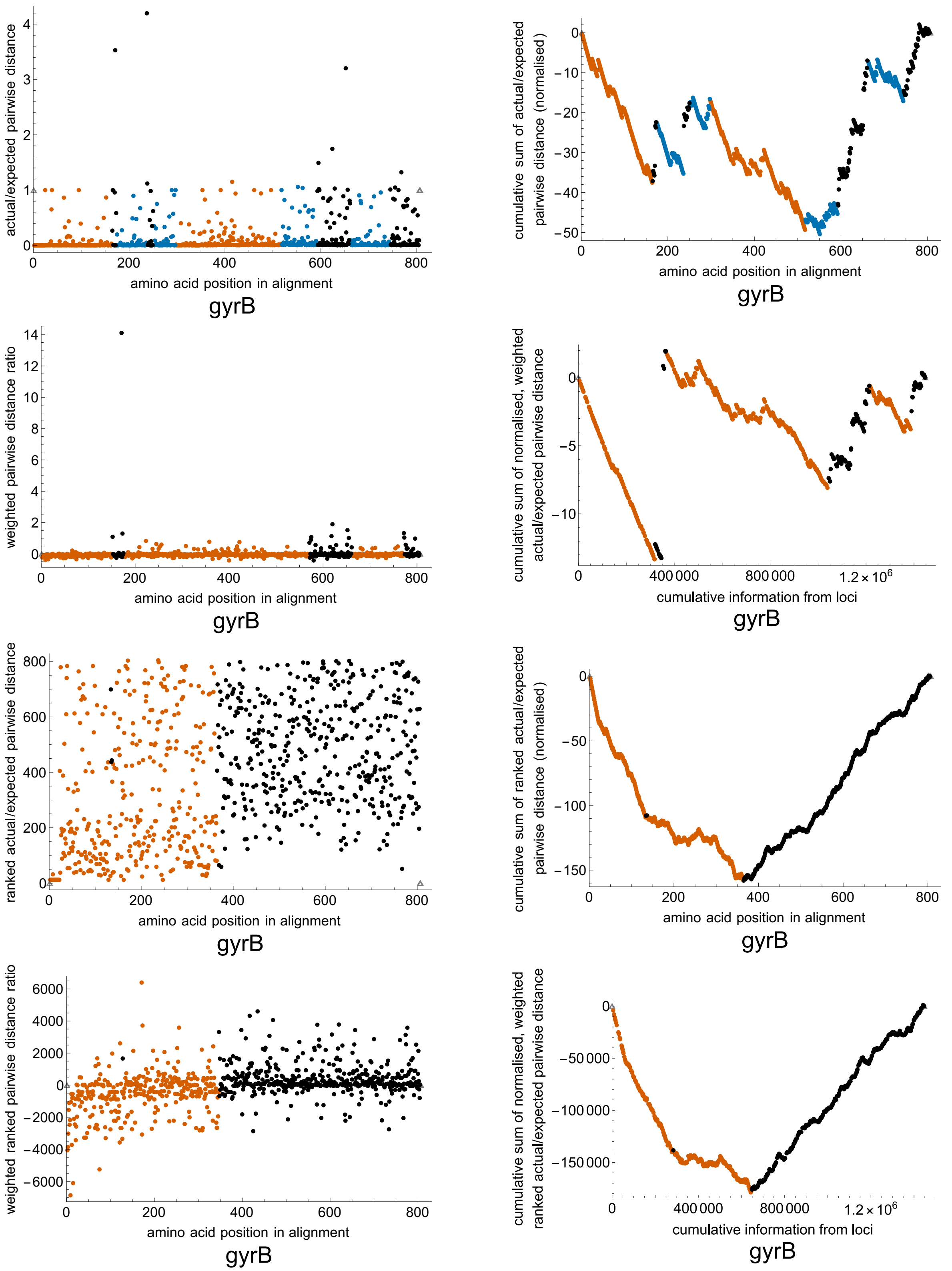

Fig. S17. Output from analyses of *E. coli* gyrB gene, with raw analysis output on left and cumulative sums on right. Each dot represents one codon. In each case, regions deemed significant by the algorithm are highlighted in orange and regions deemed significant after removal of a potentially interfering region are highlighted in blue. Codons contributing no information to the analysis are in a lighter colour and plotted with open triangles. Top row: using raw (unweighted/unranked) pairwise distance ratios. Second row: using weighted, unranked ratios. Third row: using unweighted, ranked ratios. Bottom row: using weighted, ranked ratios.

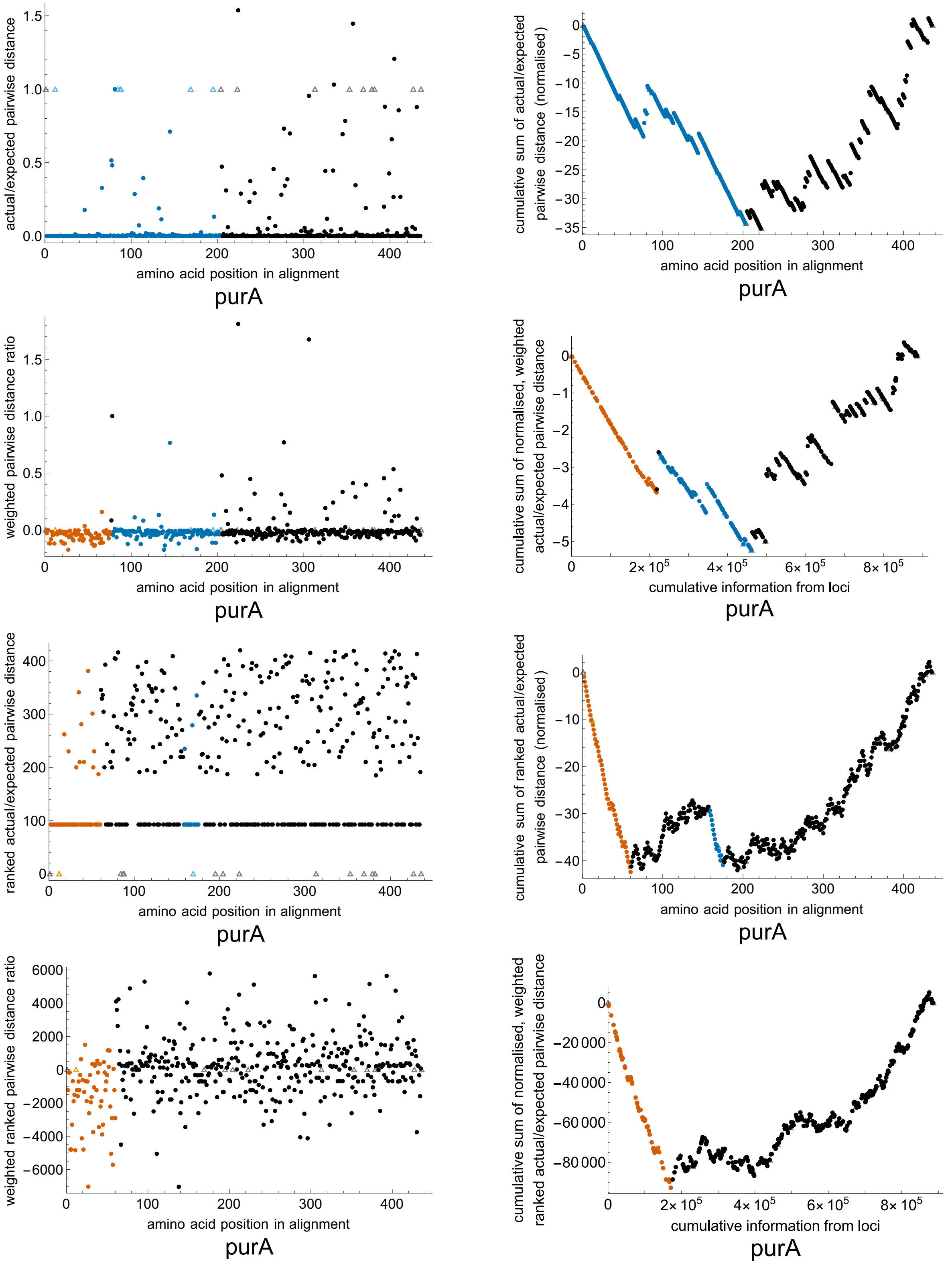

Fig. S18. Output from analyses of *E. coli* purA gene, with raw analysis output on left and cumulative sums on right. Each dot represents one codon. In each case, regions deemed significant by the algorithm are highlighted in orange and regions deemed significant after removal of a potentially interfering region are highlighted in blue. Codons contributing no information to the analysis are in a lighter colour and plotted with open triangles. Top row: using raw (unweighted/unranked) pairwise distance ratios. Second row: using weighted, unranked ratios. Third row: using unweighted, ranked ratios. Bottom row: using weighted, ranked ratios.

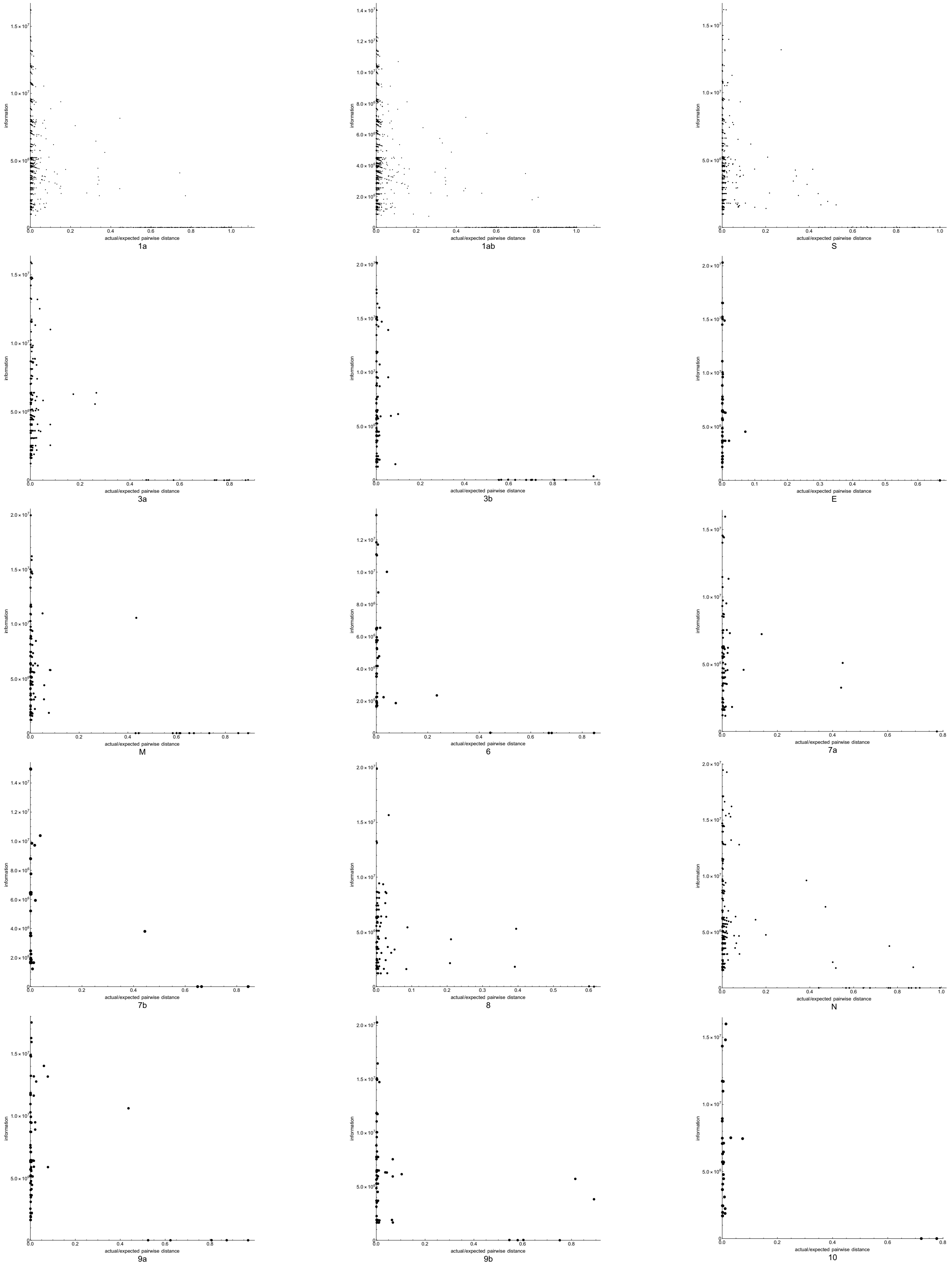

Fig. S19. Locus information versus variability for genes of SARS-CoV-2.

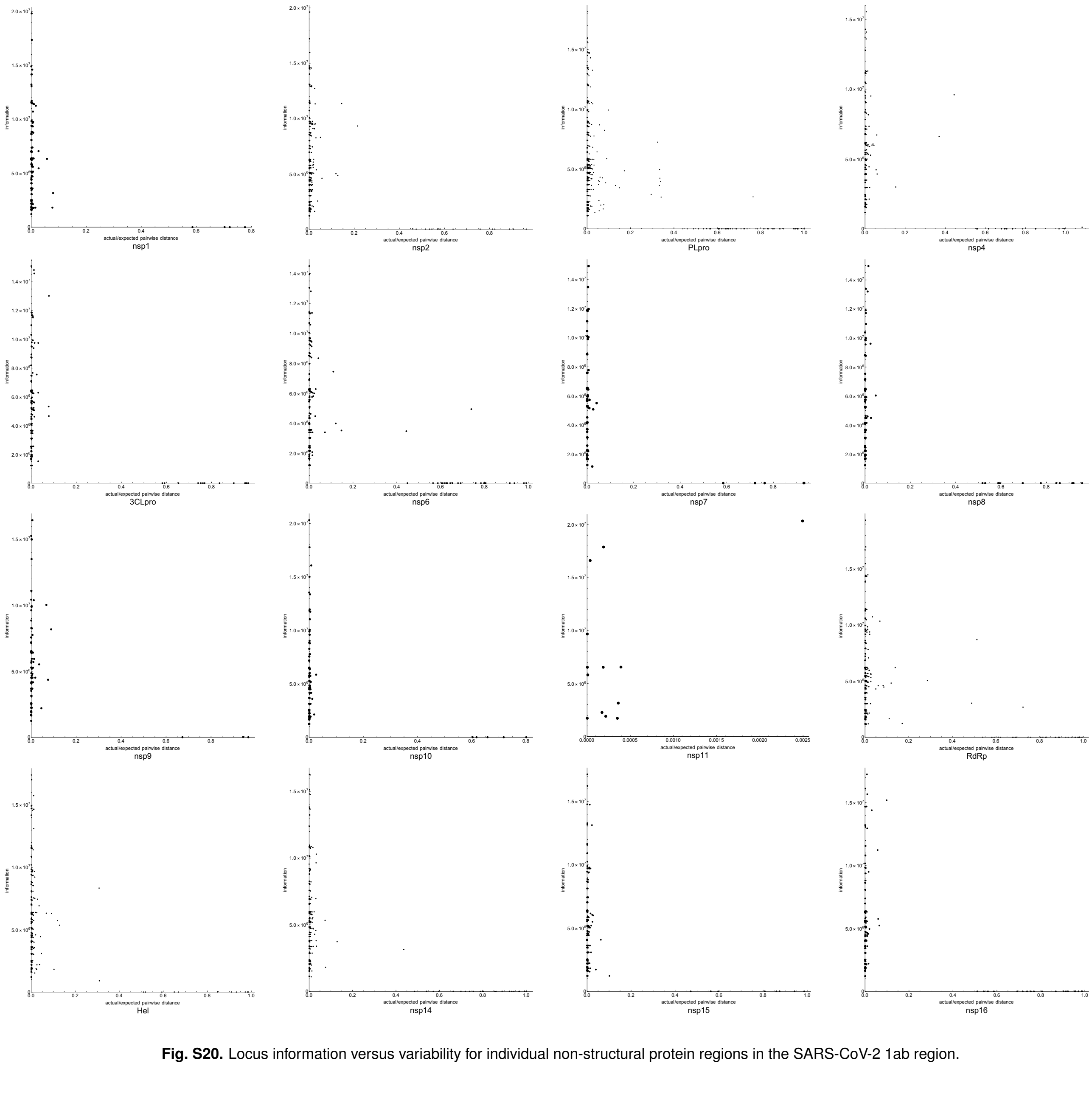

Fig. S20. Locus information versus variability for individual non-structural protein regions in the SARS-CoV-2 1ab region.

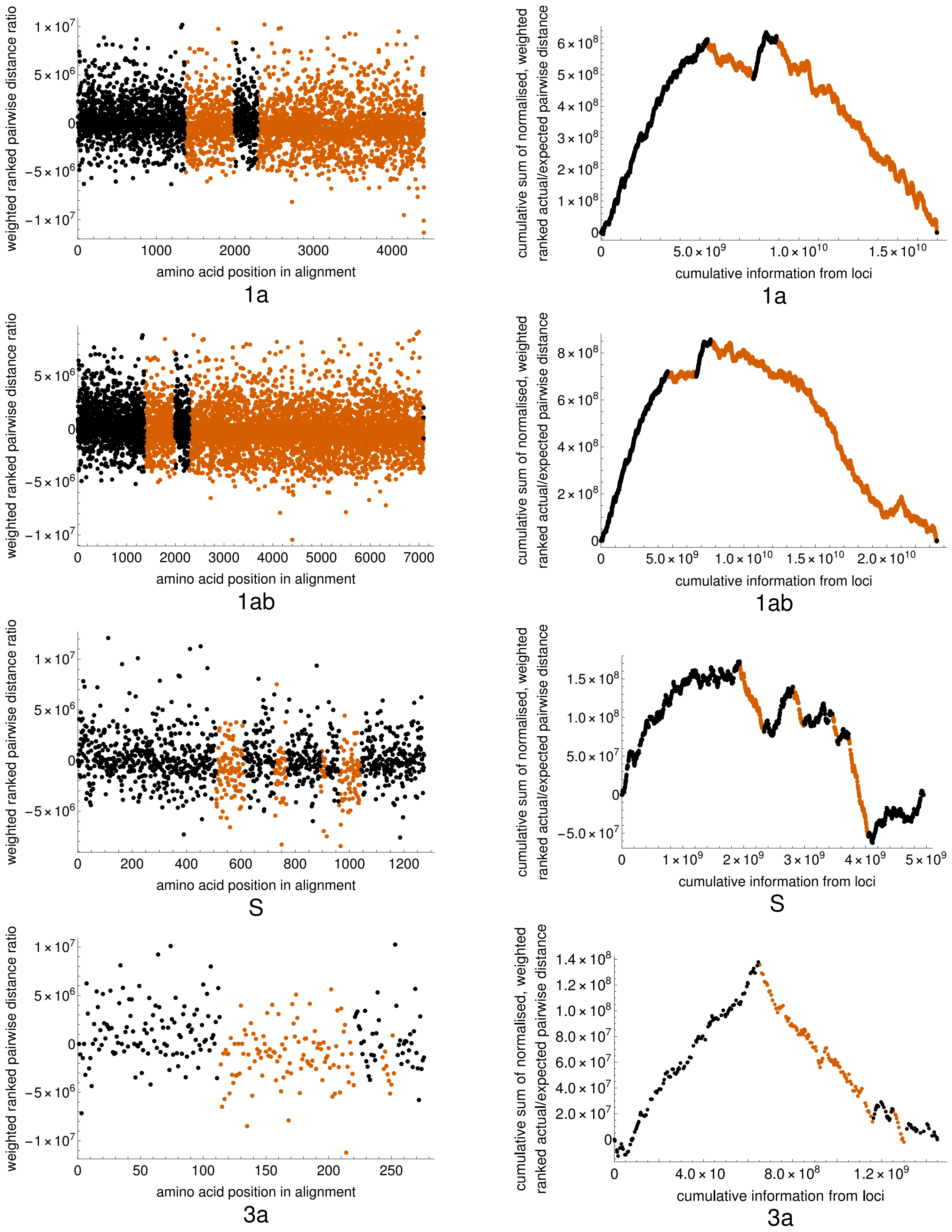

Fig. S21. Output from analyses of SARS-CoV-2 1a, 1ab, S and 3a genes, with raw analysis output on left and cumulative sums on right. Each dot represents one codon. Regions deemed significant by the algorithm are highlighted in orange and regions deemed significant after removal of a potentially interfering region are highlighted in blue. Codons contributing no information to the analysis are in a lighter colour and plotted with open triangles. Plots use weighted, ranked pairwise distance ratios. More detail of significant regions found is given in Table 1.

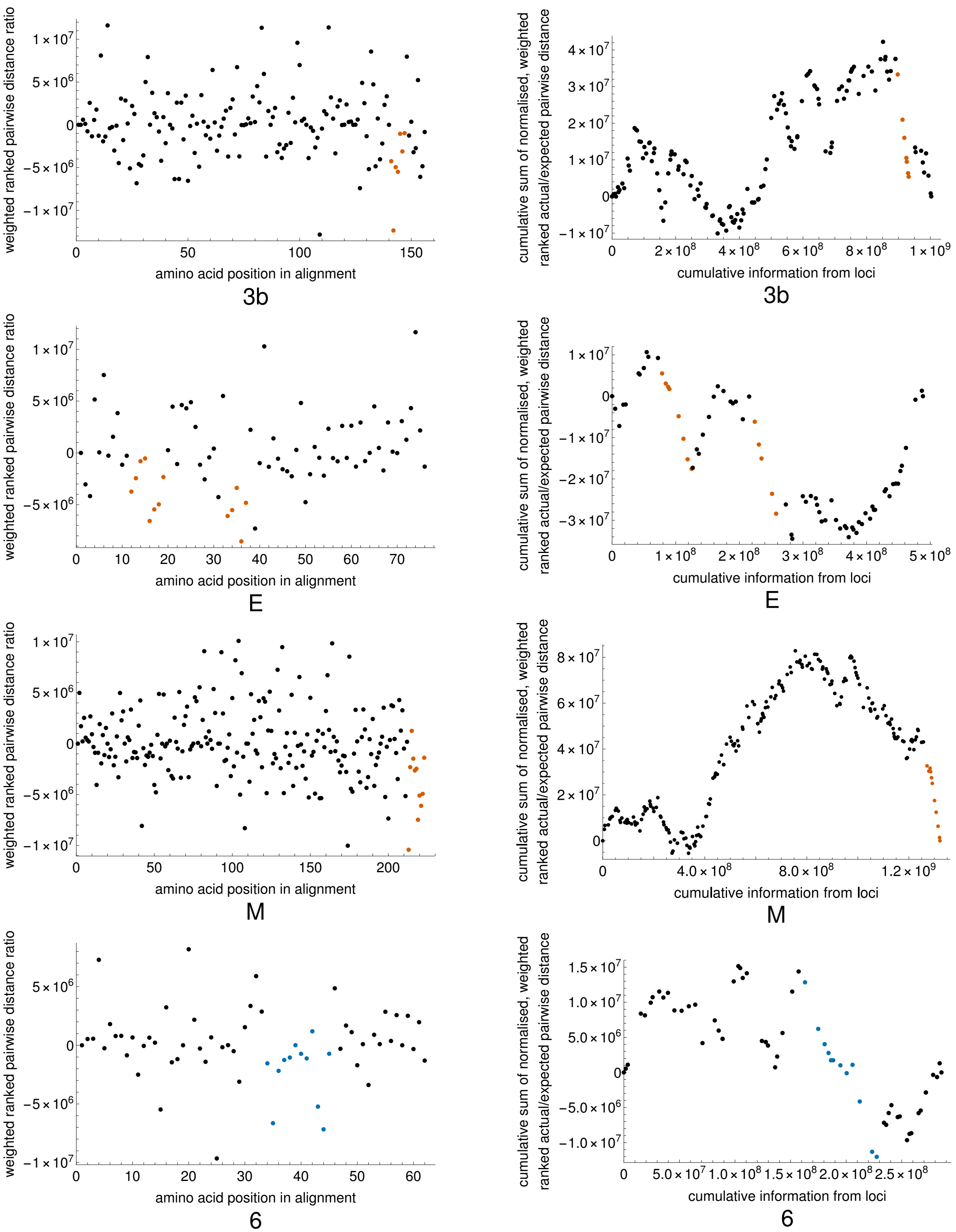

Fig. S22. Output from analyses of SARS-CoV-2 3b, E, M and 6 genes, with raw analysis output on left and cumulative sums on right. Each dot represents one codon. Regions deemed significant by the algorithm are highlighted in orange and regions deemed significant after removal of a potentially interfering region are highlighted in blue. Codons contributing no information to the analysis are in a lighter colour and plotted with open triangles. Plots use weighted, ranked pairwise distance ratios. More detail of significant regions found is given in Table 1.

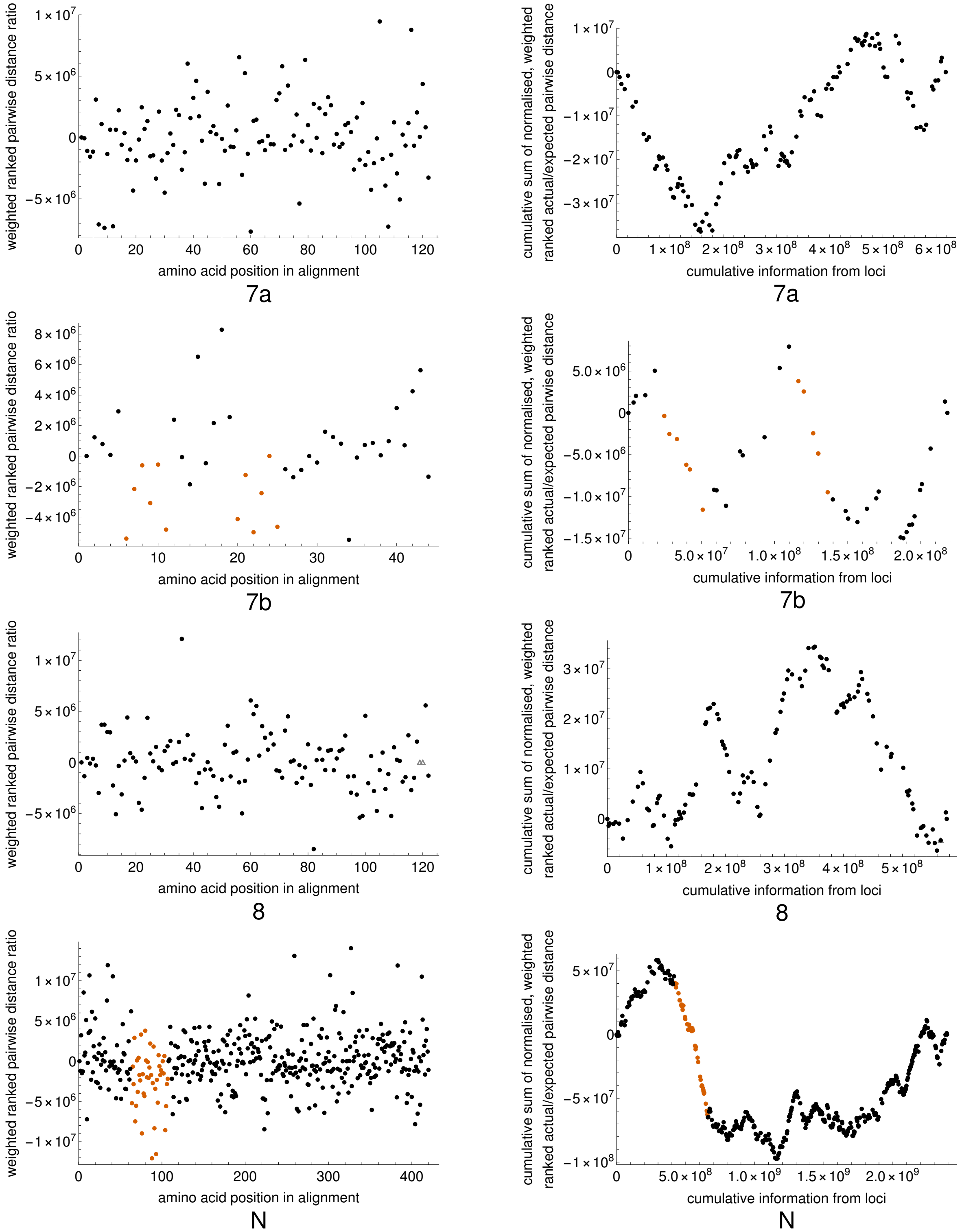

Fig. S23. Output from analyses of SARS-CoV-2 7a, 7b,8 and N genes, with raw analysis output on left and cumulative sums on right. Each dot represents one codon. Regions deemed significant by the algorithm are highlighted in orange and regions deemed significant after removal of a potentially interfering region are highlighted in blue. Codons contributing no information to the analysis are in a lighter colour and plotted with open triangles. Plots use weighted, ranked pairwise distance ratios. More detail of significant regions found is given in Table 1.

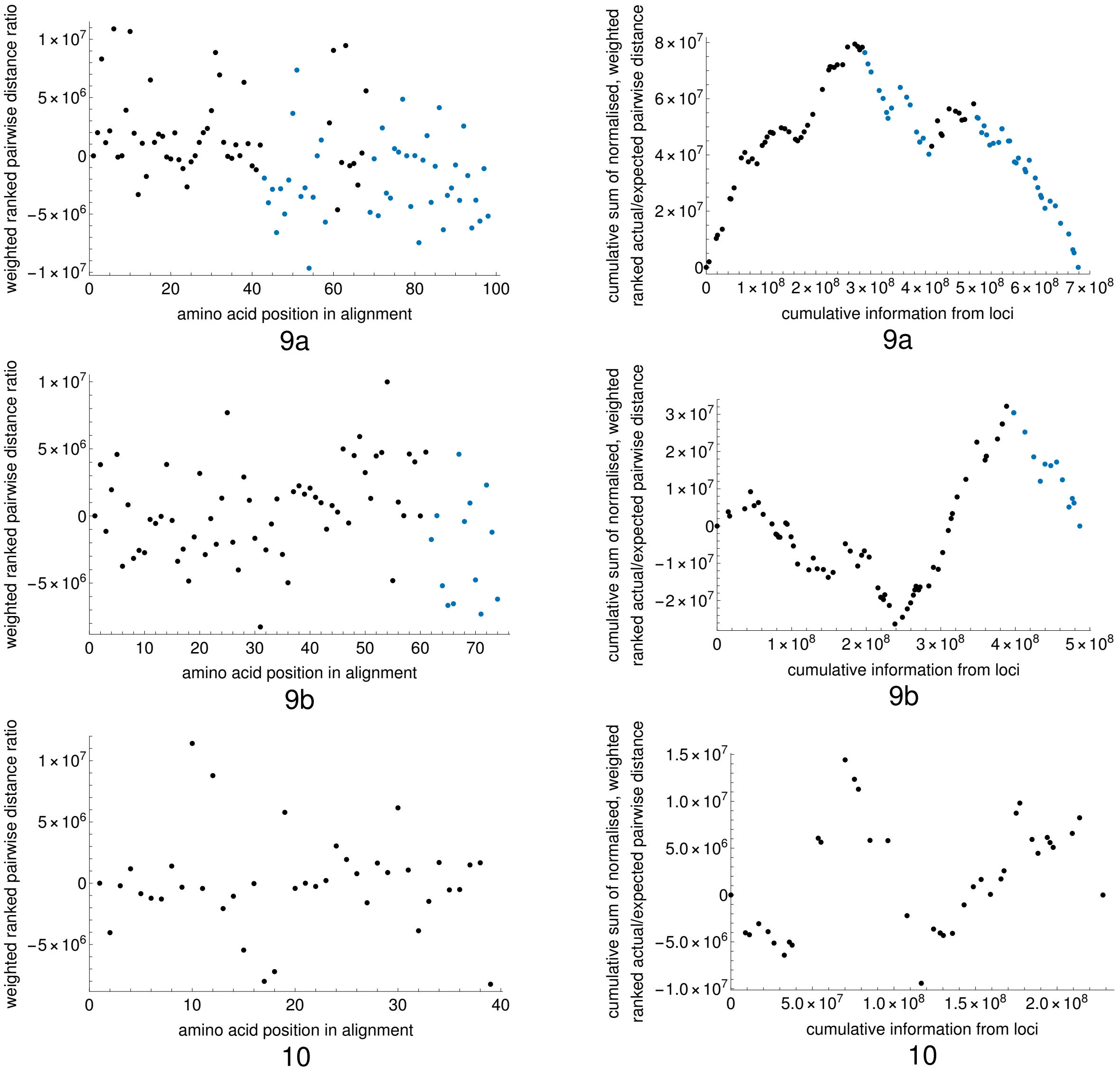

Fig. S24. Output from analyses of SARS-CoV-2 9a, 9b, and 10 genes, with raw analysis output on left and cumulative sums on right. Each dot represents one codon. Regions deemed significant by the algorithm are highlighted in orange and regions deemed significant after removal of a potentially interfering region are highlighted in blue. Codons contributing no information to the analysis are in a lighter colour and plotted with open triangles. Plots use weighted, ranked pairwise distance ratios. More detail of significant regions found is given in Table 1.

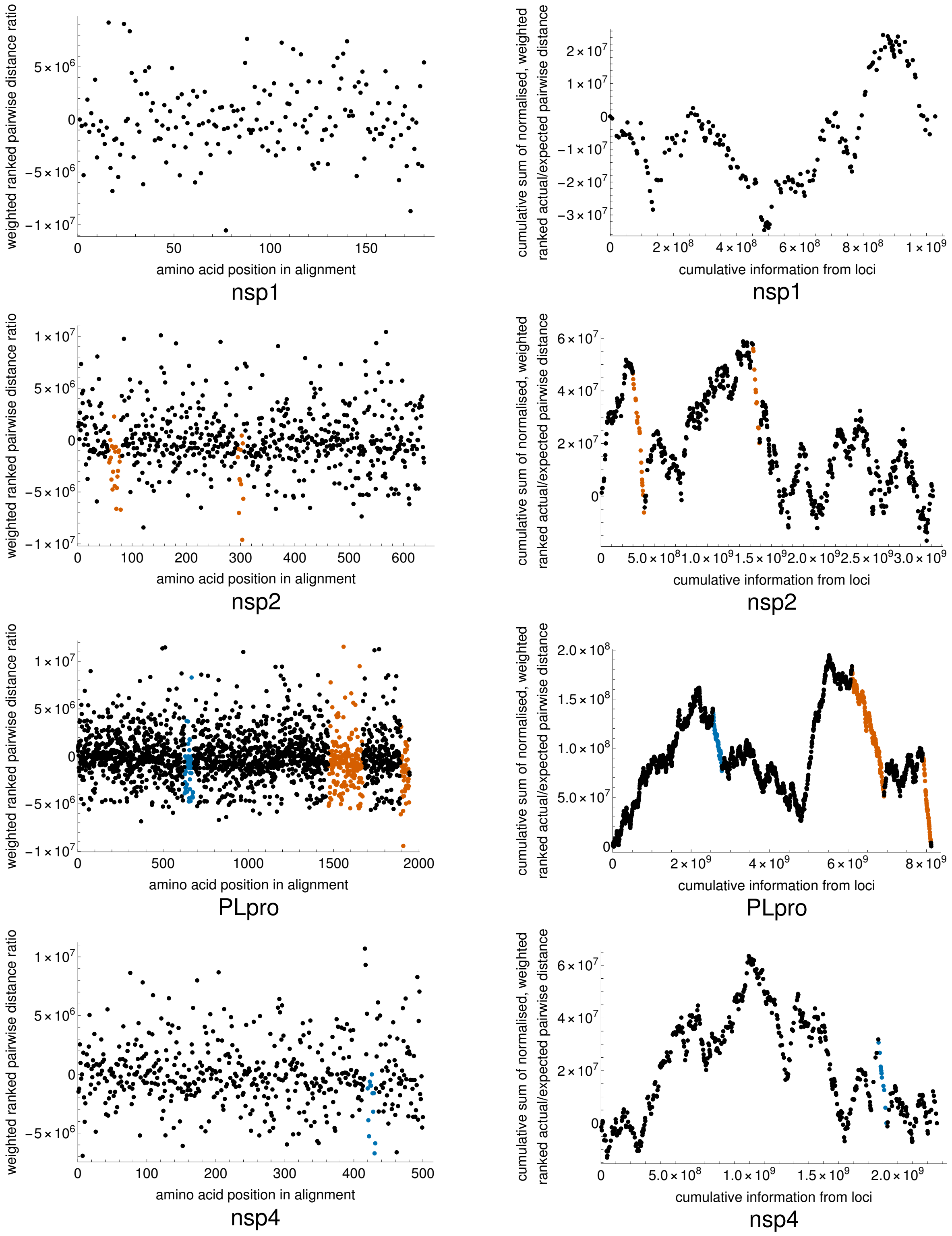

Fig. S25. Output from analyses of SARS-CoV-2 nsp1, nsp2, nsp3 and nsp4 regions, with raw analysis output on left and cumulative sums on right. Each dot represents one codon. Regions deemed significant by the algorithm are highlighted in orange and regions deemed significant after removal of a potentially interfering region are highlighted in blue. Codons contributing no information to the analysis are in a lighter colour and plotted with open triangles. Plots use weighted, ranked pairwise distance ratios. More detail of significant regions found is given in Table 2.

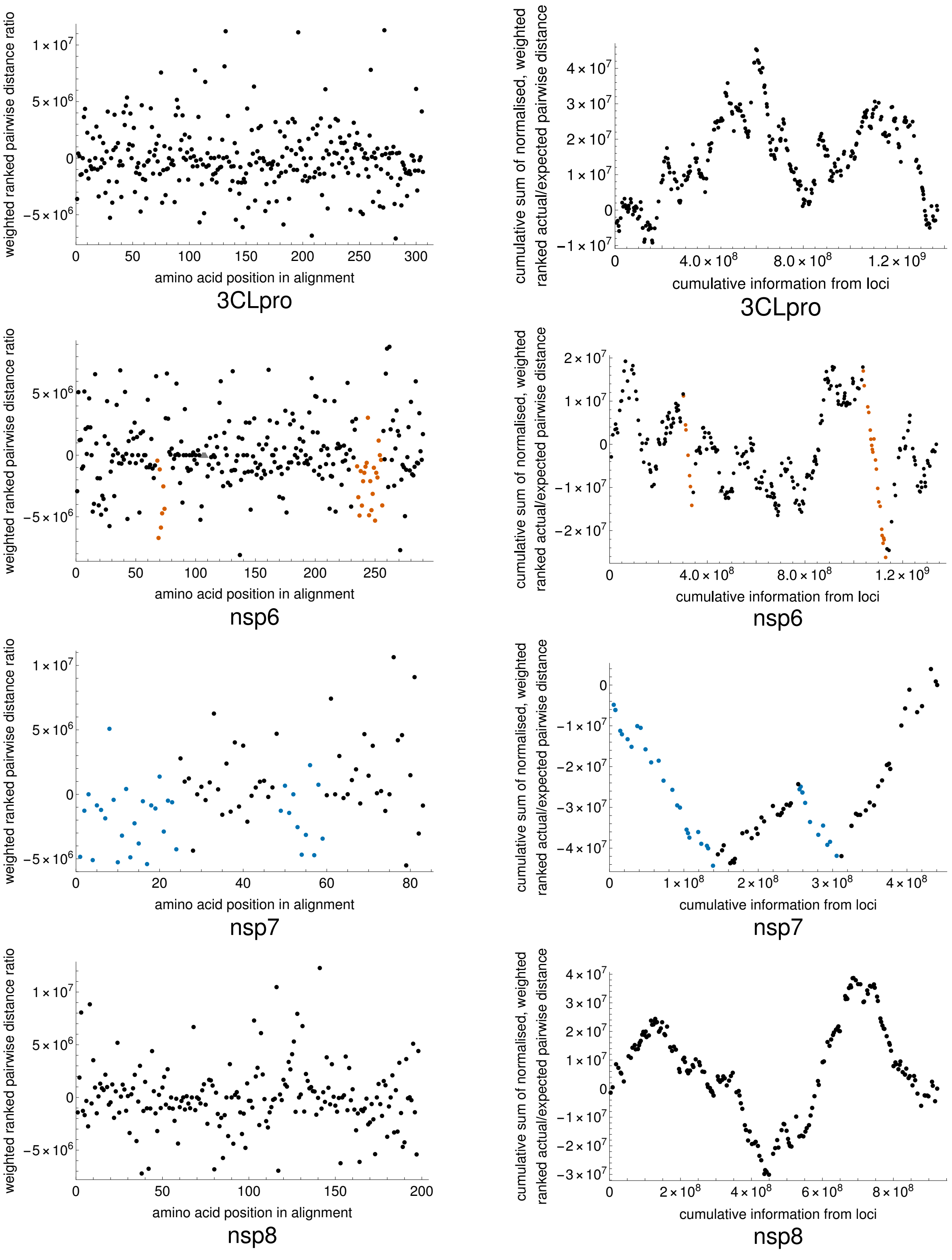

Fig. S26. Output from analyses of SARS-CoV-2 nsp5, nsp6, nsp7 and nsp8 regions, with raw analysis output on left and cumulative sums on right. Each dot represents one codon. Regions deemed significant by the algorithm are highlighted in orange and regions deemed significant after removal of a potentially interfering region are highlighted in blue. Codons contributing no information to the analysis are in a lighter colour and plotted with open triangles. Plots use weighted, ranked pairwise distance ratios. More detail of significant regions found is given in Table 2.

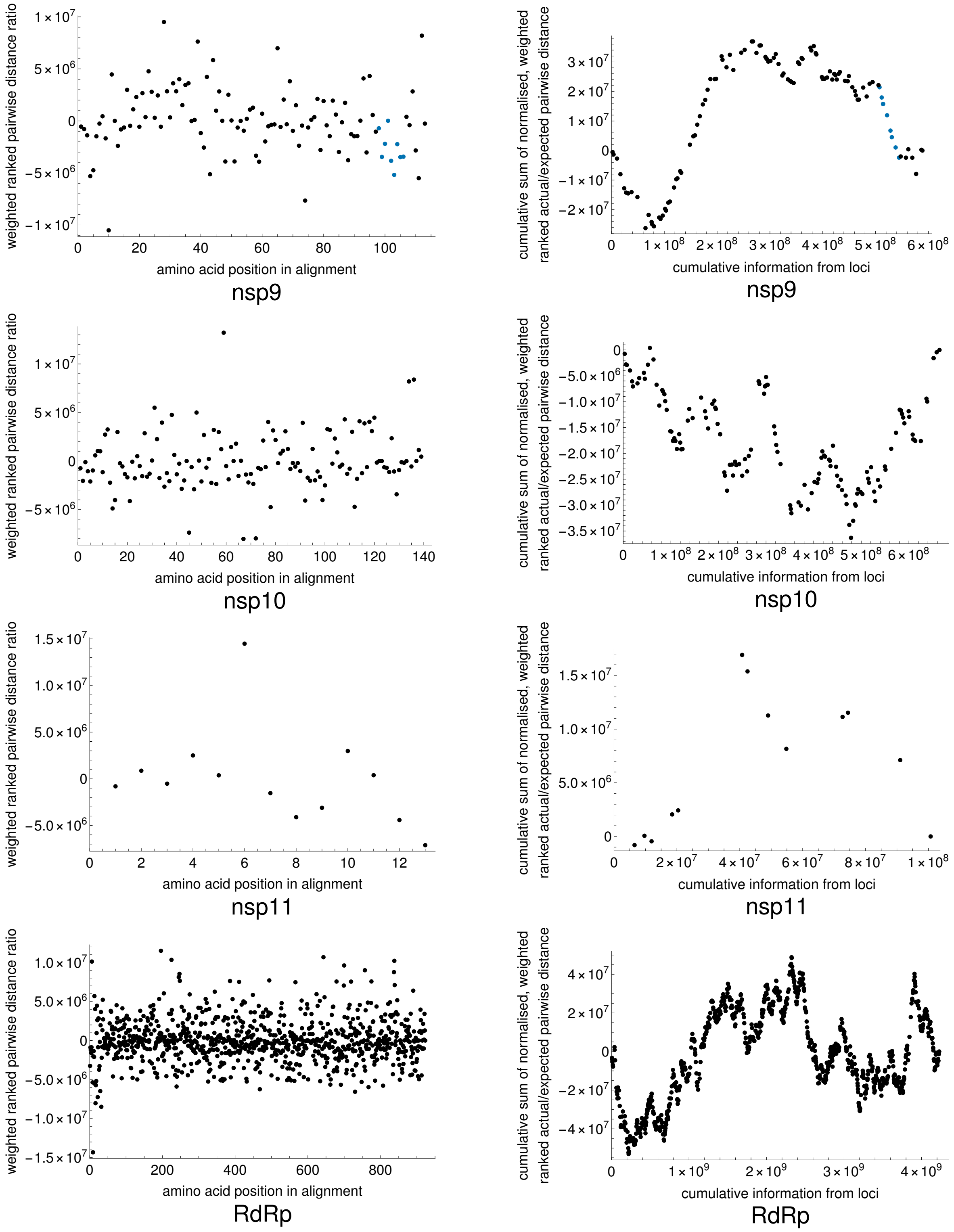

Fig. S27. Output from analyses of SARS-CoV-2 nsp9, nsp10, nsp11 and nsp12 regions, with raw analysis output on left and cumulative sums on right. Each dot represents one codon. Regions deemed significant by the algorithm are highlighted in orange and regions deemed significant after removal of a potentially interfering region are highlighted in blue. Codons contributing no information to the analysis are in a lighter colour and plotted with open triangles. Plots use weighted, ranked pairwise distance ratios. More detail of significant regions found is given in Table 2.

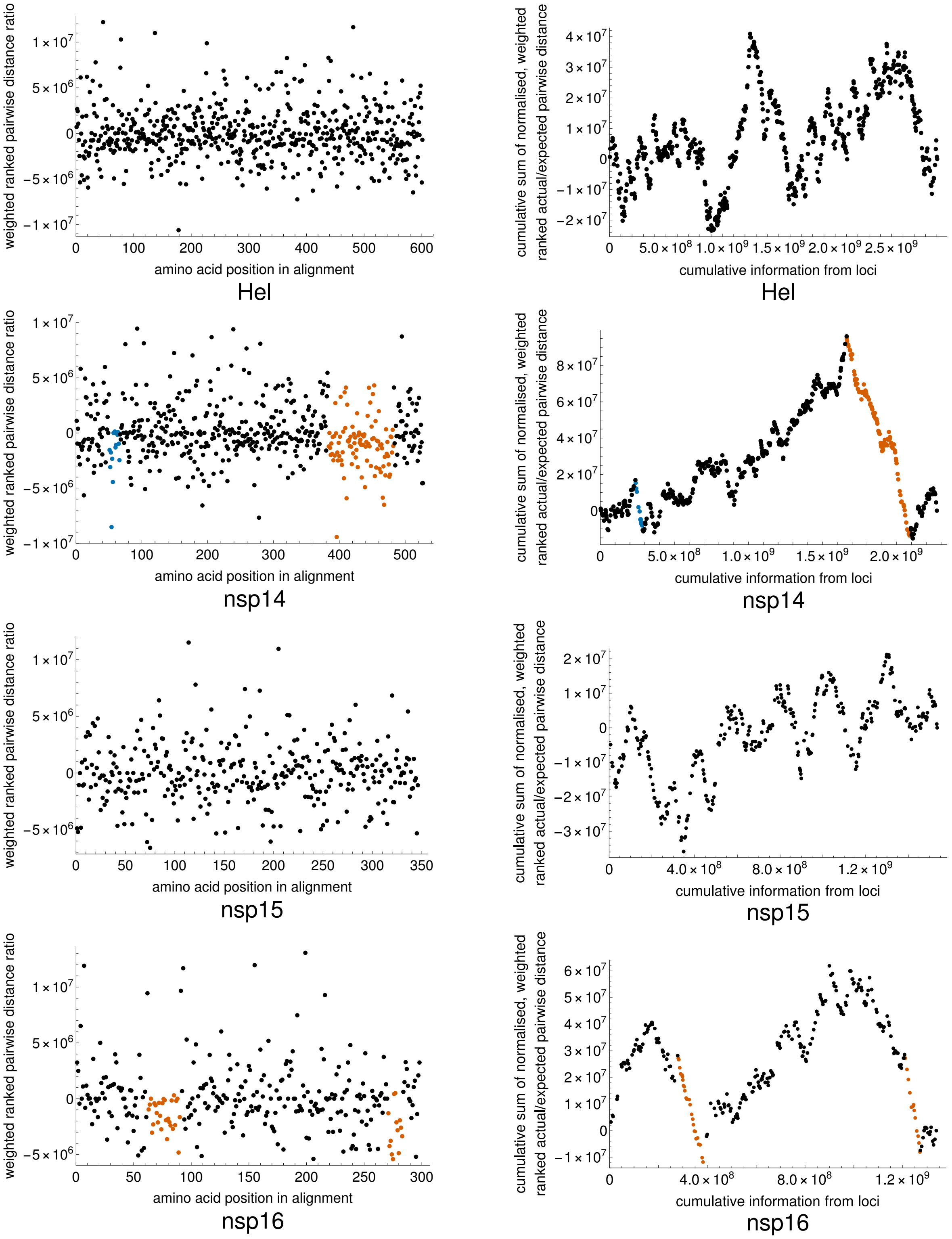

Fig. S28. Output from analyses of SARS-CoV-2 nsp13, nsp14, nsp15 and nsp16 regions, with raw analysis output on left and cumulative sums on right. Each dot represents one codon. Regions deemed significant by the algorithm are highlighted in orange and regions deemed significant after removal of a potentially interfering region are highlighted in blue. Codons contributing no information to the analysis are in a lighter colour and plotted with open triangles. Plots use weighted, ranked pairwise distance ratios. More detail of significant regions found is given in Table 2.

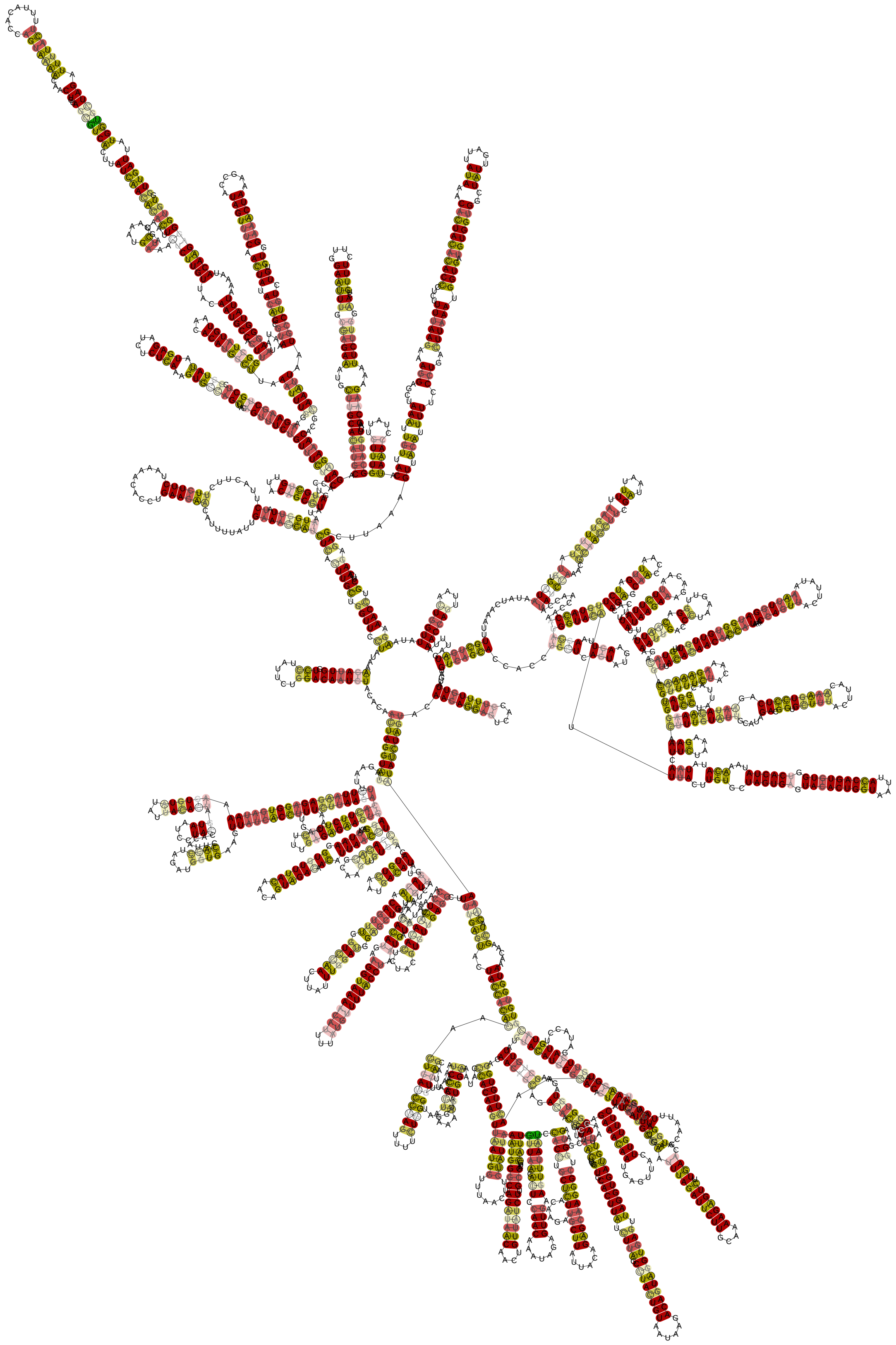

Fig. S29. Output of RNAalifold analysis of the conserved region found 2nd in analysis of the 1a region and 3rd in analysis of the 1ab region of SARS-CoV-2, NC_045512.2 nucleotide location 4335–6247. Prior to folding, aligned sequences used in the main analysis were deduplicated so that one example of each observed primary structure for the conserved region was used as input to the algorithm. (Results are displayed using sequences from the 1a analysis, from which fewer sequences were excluded owing to ambiguous nucleotides etc.) Base pairs are highlighted in deep/mid/light red as all/all but one/all but two sequences are capable of forming the pairs shown. Base pairs are highlighted in deep/mid/light yellow as all/all but one/all but two sequences are capable of forming the pair shown or one other base pair (including GU pairs). Base pairs are highlighted in deep/mid/light green as all/all but one/all but two sequences are capable of forming the pair shown or either of another two base pairs (including GU pairs). RNAalifold was used with input options disallowing lonely pairs, allowing G-quadruplexes, and with the ribosum scoring matrix enabled.

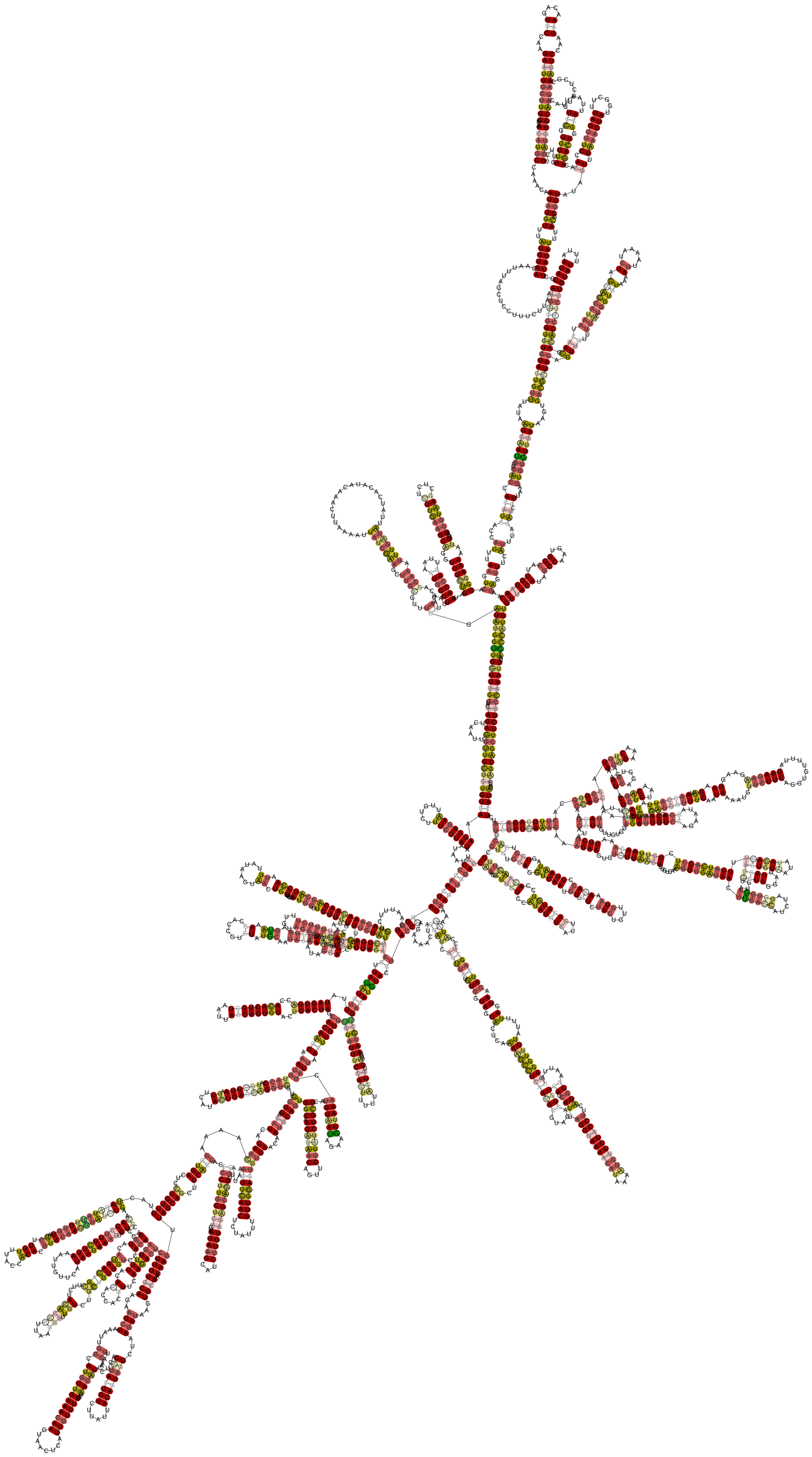

Fig. S30. Output of RNAalifold analysis of the reverse complement of the conserved region found 2nd in analysis of the 1a region and 3rd in analysis of the 1ab region of SARS-CoV-2, NC_045512.2 nucleotide location 4335–6247. Prior to folding, aligned sequences used in the main analysis were deduplicated so that one example of each observed primary structure for the conserved region was used as input to the algorithm. (Results are displayed using sequences from the 1a analysis, from which fewer sequences were excluded owing to ambiguous nucleotides etc.) Base pairs are highlighted in deep/mid/light red as all/all but one/all but two sequences are capable of forming the pairs shown. Base pairs are highlighted in deep/mid/light yellow as all/all but one/all but two sequences are capable of forming the pair shown or one other base pair (including GU pairs). Base pairs are highlighted in deep/mid/light green as all/all but one/all but two sequences are capable of forming the pair shown or either of another two base pairs (including GU pairs). RNAalifold was used with input options disallowing lonely pairs, allowing G-quadruplexes, and with the ribosum scoring matrix enabled.

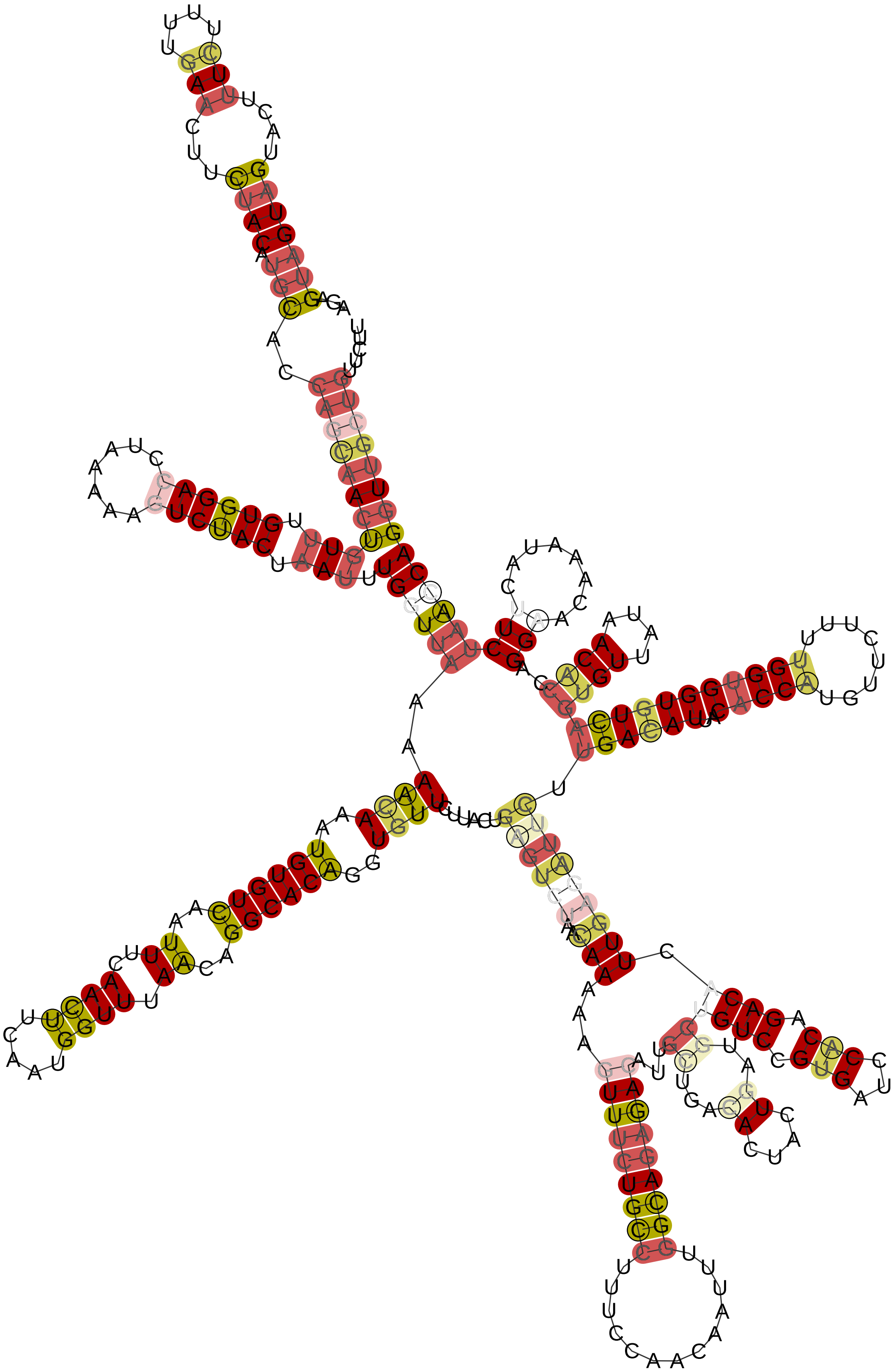

Fig. S31. Output of RNAalifold analysis of the conserved region found 2nd in analysis of the S region of SARS-CoV-2, NC_045512.2 nucleotide location 23087–23395. Prior to folding, aligned sequences used in the main analysis were deduplicated so that one example of each observed primary structure for the conserved region was used as input to the algorithm. Base pairs are highlighted in deep/mid/light red as all/all but one/all but two sequences are capable of forming the pairs shown. Base pairs are highlighted in deep/mid/light yellow as all/all but one/all but two sequences are capable of forming the pair shown or one other base pair (including GU pairs). Base pairs are highlighted in deep/mid/light green as all/all but one/all but two sequences are capable of forming the pair shown or either of another two base pairs (including GU pairs). RNAalifold was used with input options disallowing lonely pairs, allowing G-quadruplexes, and with the ribosum scoring matrix enabled.

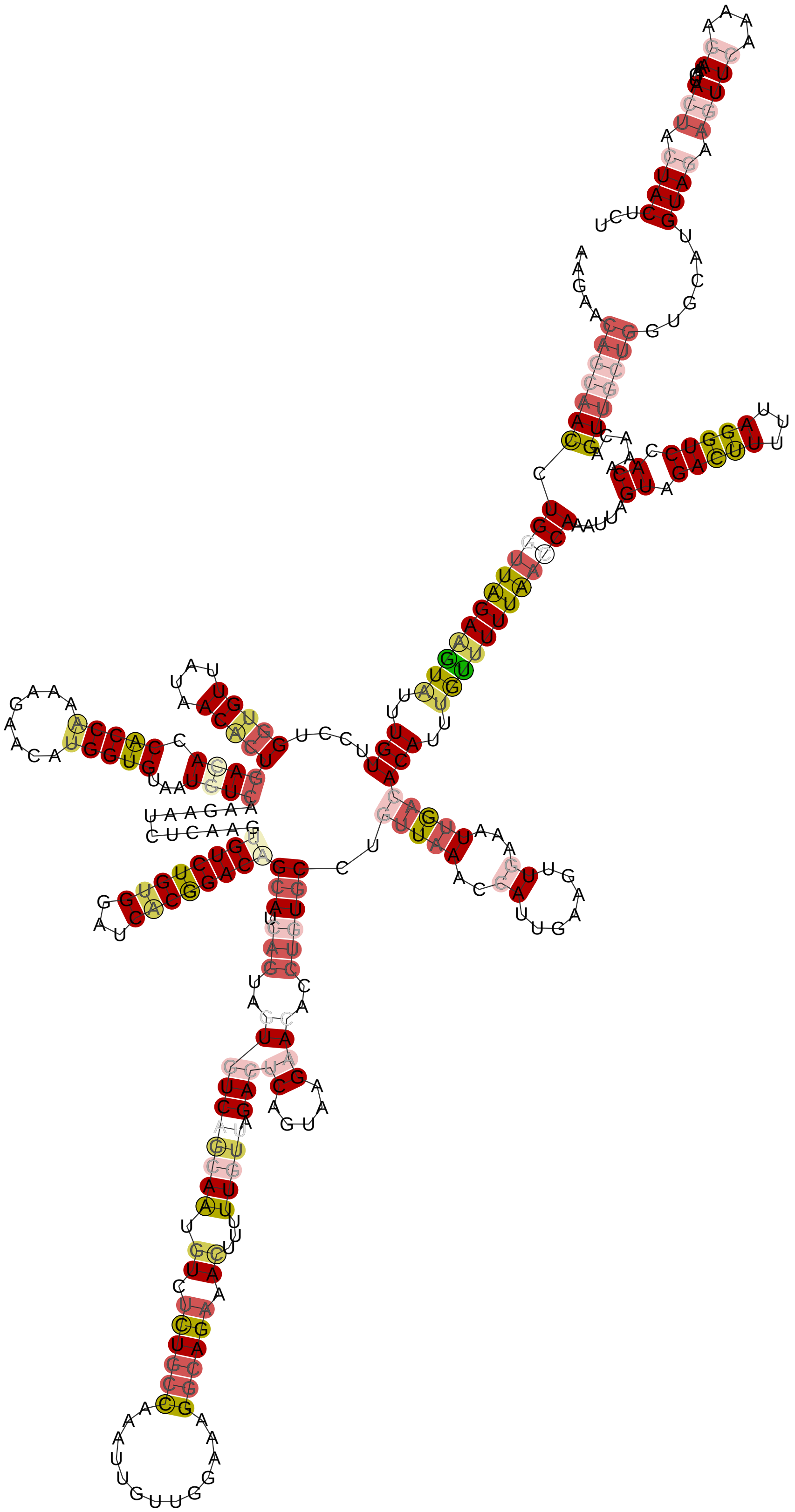

Fig. S32. Output of RNAalifold analysis of the reverse complement of the conserved region found 2nd in analysis of the S region of SARS-CoV-2, NC_045512.2 nucleotide location 23087–23395. Prior to folding, aligned sequences used in the main analysis were deduplicated so that one example of each observed primary structure for the conserved region was used as input to the algorithm. Base pairs are highlighted in deep/mid/light red as all/all but one/all but two sequences are capable of forming the pairs shown. Base pairs are highlighted in deep/mid/light yellow as all/all but one/all but two sequences are capable of forming the pair shown or one other base pair (including GU pairs). Base pairs are highlighted in deep/mid/light green as all/all but one/all but two sequences are capable of forming the pair shown or either of another two base pairs (including GU pairs). RNAalifold was used with input options disallowing lonely pairs, allowing G-quadruplexes, and with the ribosum scoring matrix enabled.

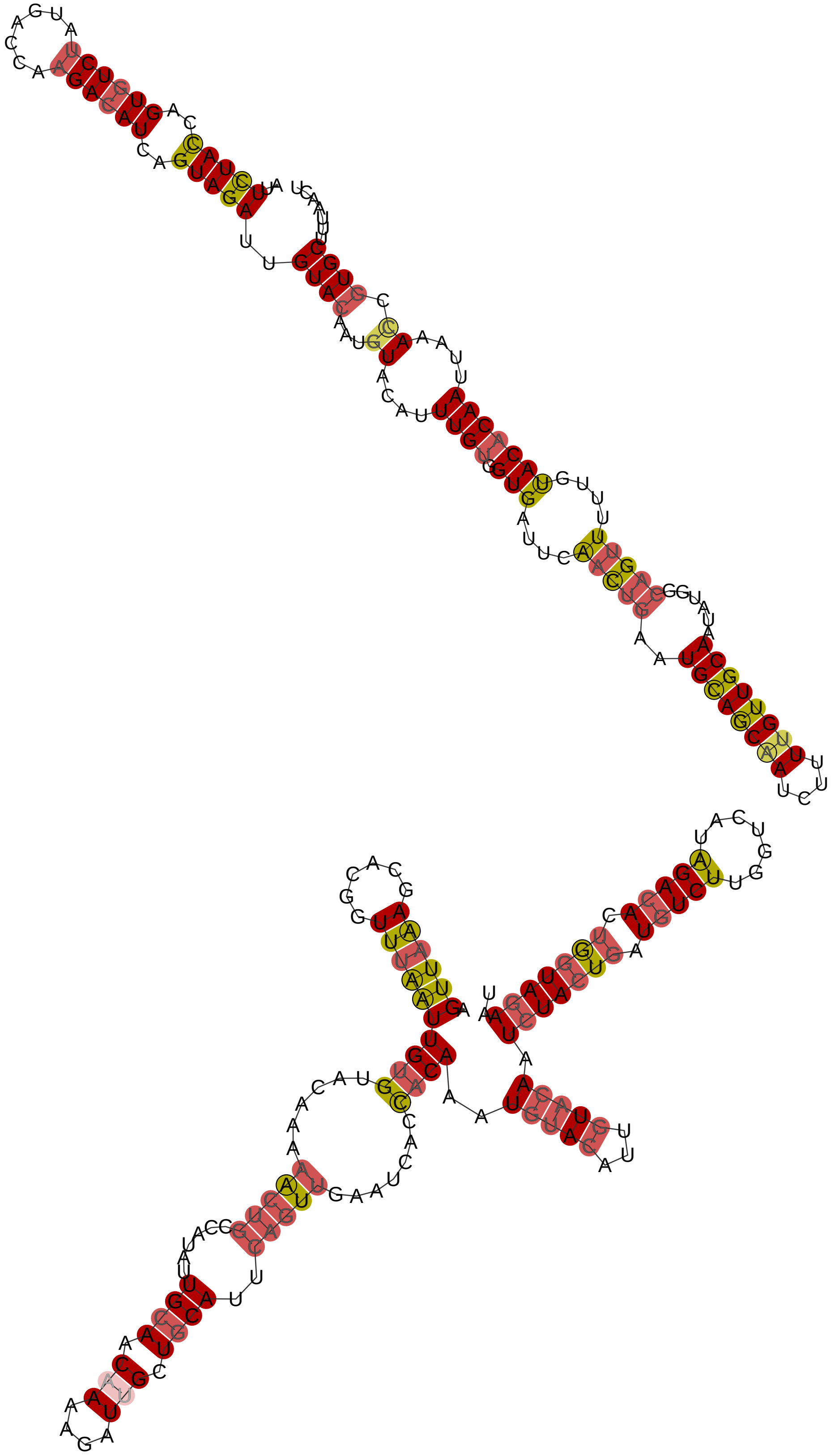

Fig. S33. Output of RNAalifold analysis of the conserved region found 3rd in analysis of the S region of SARS-CoV-2, NC_045512.2 nucleotide location 23735–26052 (top: forward sense; bottom: reverse complement). Prior to folding, aligned sequences used in the main analysis were deduplicated so that one example of each observed primary structure for the conserved region was used as input to the algorithm. Base pairs are highlighted in deep/mid/light red as all/all but one/all but two sequences are capable of forming the pairs shown. Base pairs are highlighted in deep/mid/light yellow as all/all but one/all but two sequences are capable of forming the pair shown or one other base pair (including GU pairs). Base pairs are highlighted in deep/mid/light green as all/all but one/all but two sequences are capable of forming the pair shown or either of another two base pairs (including GU pairs). RNAalifold was used with input options disallowing lonely pairs, allowing G-quadruplexes, and with the ribosum scoring matrix enabled.

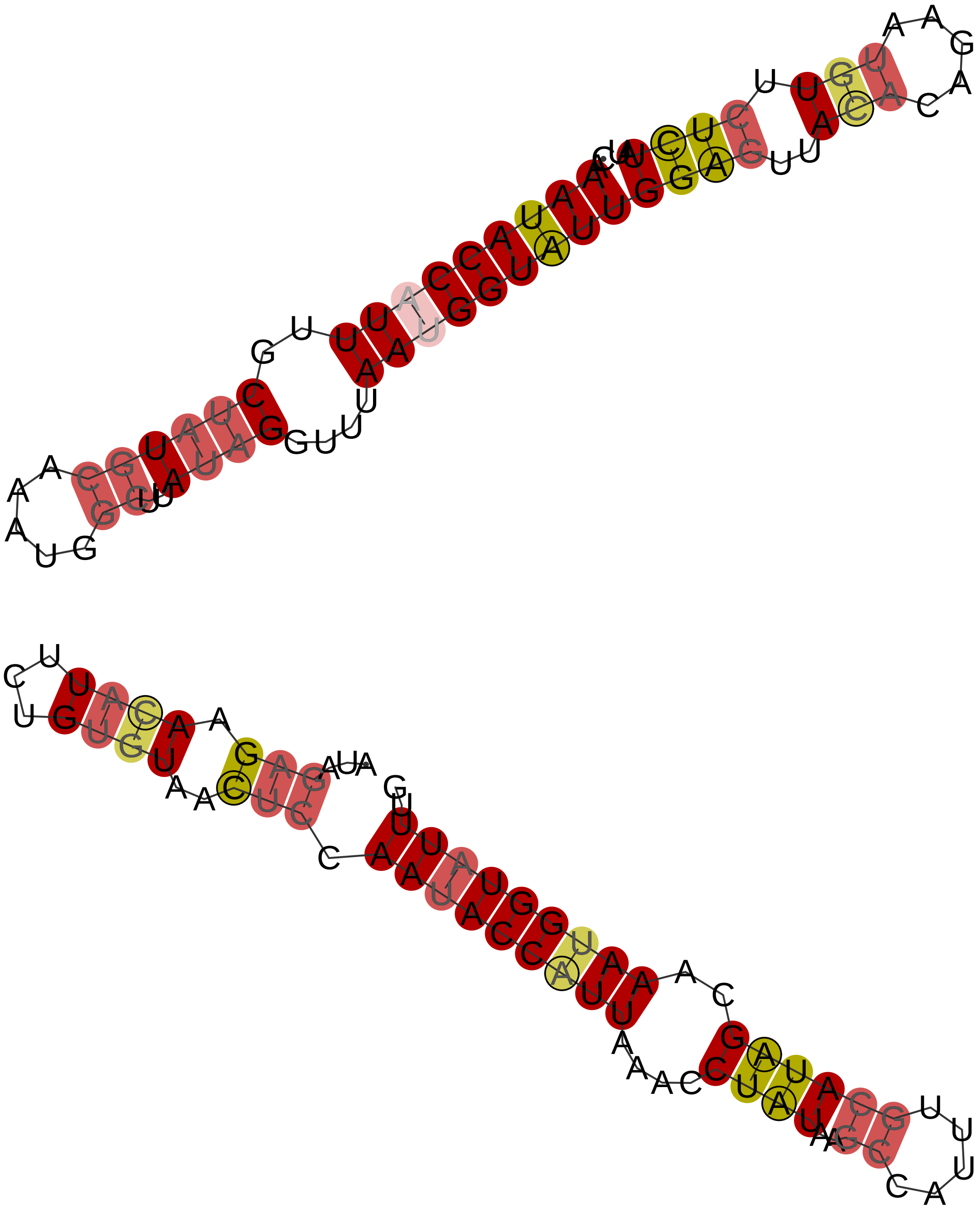

Fig. S34. Output of RNAalifold analysis of the conserved region found 4th in analysis of the S region of SARS-CoV-2, NC_045512.2 nucleotide location 24242–24313 (top: forward sense; bottom: reverse complement). Prior to folding, aligned sequences used in the main analysis were deduplicated so that one example of each observed primary structure for the conserved region was used as input to the algorithm. Base pairs are highlighted in deep/mid/light red as all/all but one/all but two sequences are capable of forming the pairs shown. Base pairs are highlighted in deep/mid/light yellow as all/all but one/all but two sequences are capable of forming the pair shown or one other base pair (including GU pairs). Base pairs are highlighted in deep/mid/light green as all/all but one/all but two sequences are capable of forming the pair shown or either of another two base pairs (including GU pairs). RNAalifold was used with input options disallowing lonely pairs, allowing G-quadruplexes, and with the ribosum scoring matrix enabled.

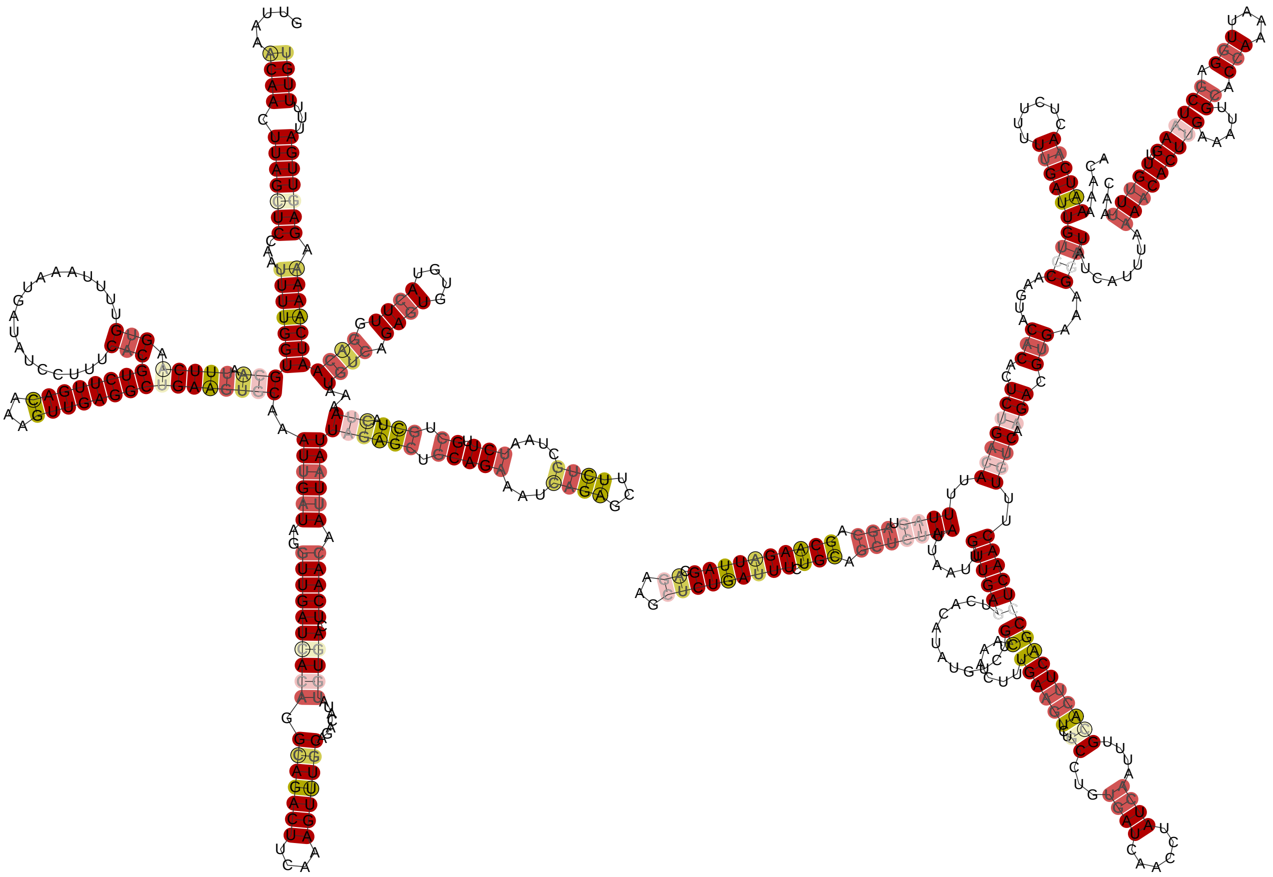

Fig. S35. Output of RNAalifold analysis of the conserved region found 1st in analysis of the S region of SARS-CoV-2, NC_045512.2 nucleotide location 24446–24690 (left: forward sense; right: reverse complement). Prior to folding, aligned sequences used in the main analysis were deduplicated so that one example of each observed primary structure for the conserved region was used as input to the algorithm. Base pairs are highlighted in deep/mid/light red as all/all but one/all but two sequences are capable of forming the pairs shown. Base pairs are highlighted in deep/mid/light yellow as all/all but one/all but two sequences are capable of forming the pair shown or one other base pair (including GU pairs). Base pairs are highlighted in deep/mid/light green as all/all but one/all but two sequences are capable of forming the pair shown or either of another two base pairs (including GU pairs). RNAalifold was used with input options disallowing lonely pairs, allowing G-quadruplexes, and with the ribosum scoring matrix enabled.

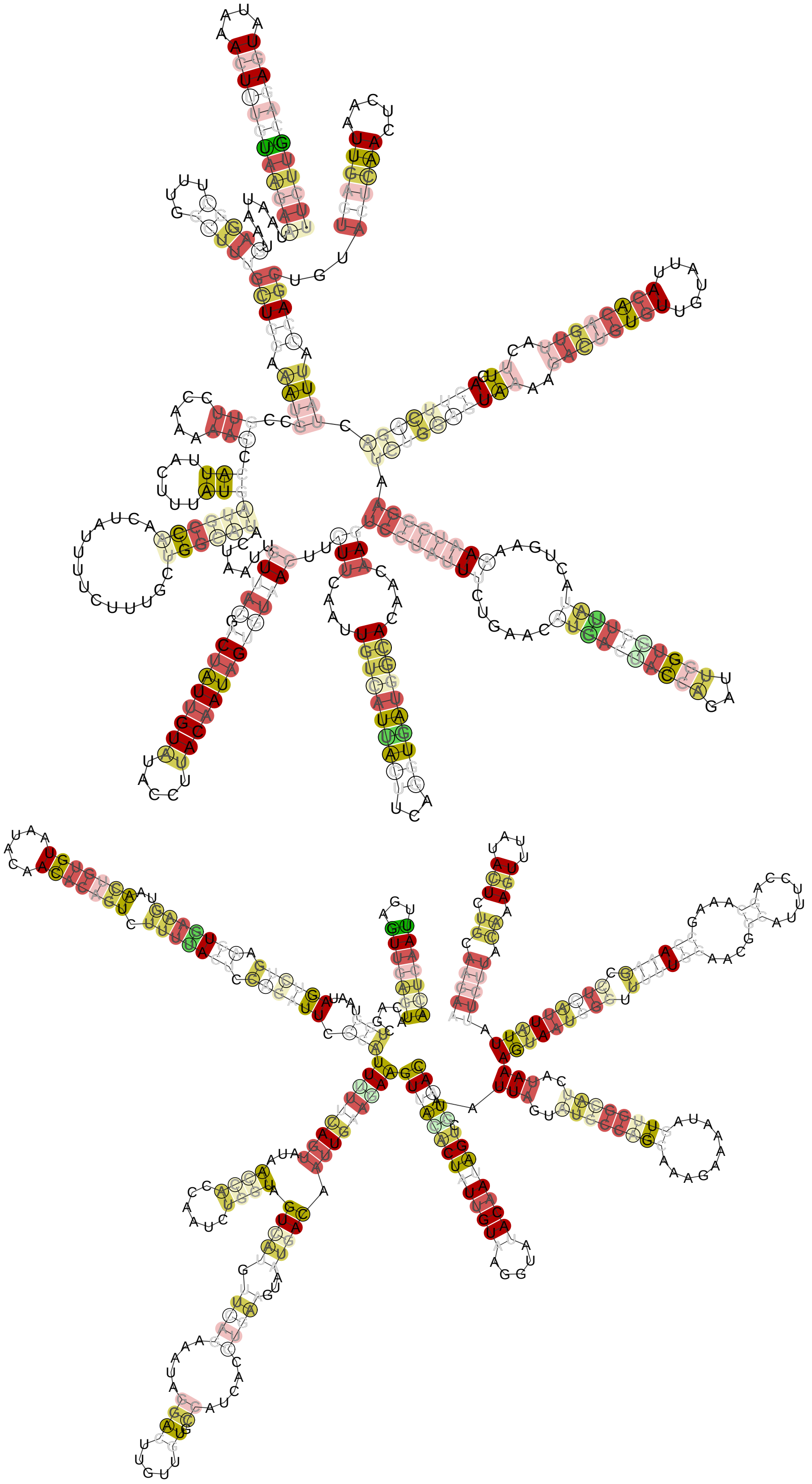

Fig. S36. Output of RNAalifold analysis of the conserved region found 1st in analysis of the 3a region of SARS-CoV-2, NC_045512.2 nucleotide location 25732–26052 (top: forward sense; bottom: reverse complement). Prior to folding, aligned sequences used in the main analysis were deduplicated so that one example of each observed primary structure for the conserved region was used as input to the algorithm. Base pairs are highlighted in deep/mid/light red as all/all but one/all but two sequences are capable of forming the pairs shown. Base pairs are highlighted in deep/mid/light yellow as all/all but one/all but two sequences are capable of forming the pair shown or one other base pair (including GU pairs). Base pairs are highlighted in deep/mid/light green as all/all but one/all but two sequences are capable of forming the pair shown or either of another two base pairs (including GU pairs). RNAalifold was used with input options disallowing lonely pairs, allowing G-quadruplexes, and with the ribosum scoring matrix enabled.

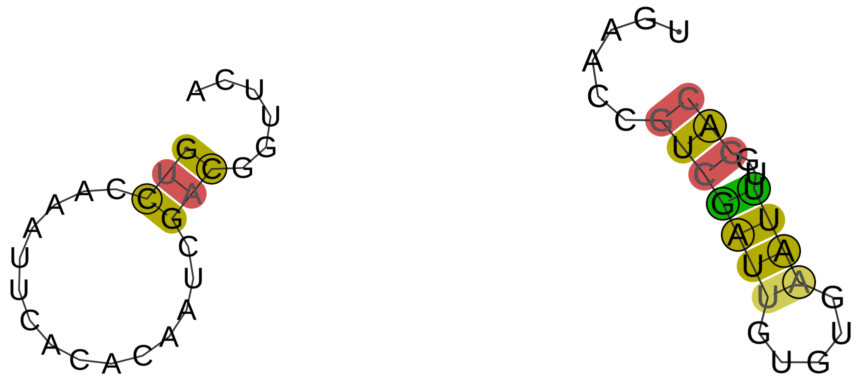

Fig. S37. Output of RNAalifold analysis of the conserved region found 2nd in analysis of the 3a region of SARS-CoV-2, NC_045512.2 nucleotide location 26119–26148 (left: forward sense; right: reverse complement). Prior to folding, aligned sequences used in the main analysis were deduplicated so that one example of each observed primary structure for the conserved region was used as input to the algorithm. Base pairs are highlighted in deep/mid/light red as all/all but one/all but two sequences are capable of forming the pairs shown. Base pairs are highlighted in deep/mid/light yellow as all/all but one/all but two sequences are capable of forming the pair shown or one other base pair (including GU pairs). Base pairs are highlighted in deep/mid/light green as all/all but one/all but two sequences are capable of forming the pair shown or either of another two base pairs (including GU pairs). RNAalifold was used with input options disallowing lonely pairs, allowing G-quadruplexes, and with the ribosum scoring matrix enabled.

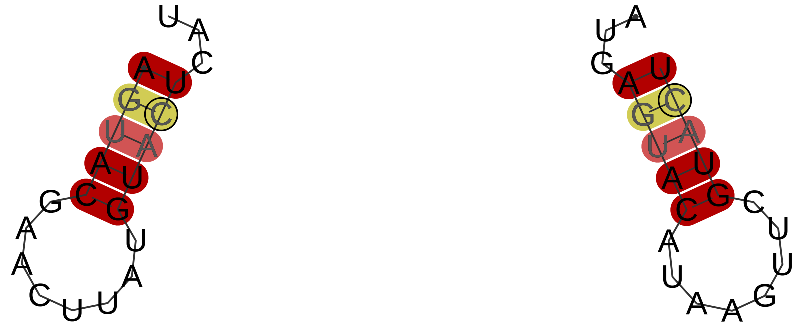

Fig. S38. Output of RNAalifold analysis of the conserved region found 1st in analysis of the 3b region of SARS-CoV-2, NC_045512.2 nucleotide location 26234–26254 (left: forward sense; right: reverse complement). Prior to folding, aligned sequences used in the main analysis were deduplicated so that one example of each observed primary structure for the conserved region was used as input to the algorithm. Base pairs are highlighted in deep/mid/light red as all/all but one/all but two sequences are capable of forming the pairs shown. Base pairs are highlighted in deep/mid/light yellow as all/all but one/all but two sequences are capable of forming the pair shown or one other base pair (including GU pairs). Base pairs are highlighted in deep/mid/light green as all/all but one/all but two sequences are capable of forming the pair shown or either of another two base pairs (including GU pairs). RNAalifold was used with input options disallowing lonely pairs, allowing G-quadruplexes, and with the ribosum scoring matrix enabled.

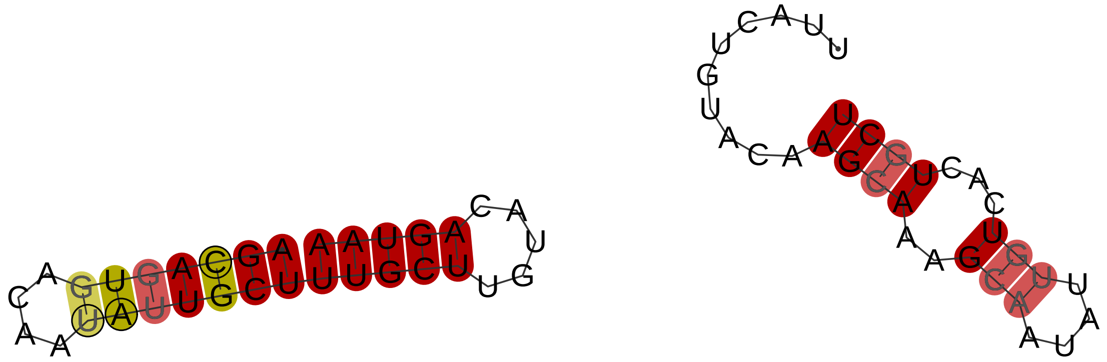

Fig. S39. Output of RNAalifold analysis of the conserved region found 1st in analysis of the M region of SARS-CoV-2, NC_045512.2 nucleotide location 27159–27191 (left: forward sense; right: reverse complement). Prior to folding, aligned sequences used in the main analysis were deduplicated so that one example of each observed primary structure for the conserved region was used as input to the algorithm. Base pairs are highlighted in deep/mid/light red as all/all but one/all but two sequences are capable of forming the pairs shown. Base pairs are highlighted in deep/mid/light yellow as all/all but one/all but two sequences are capable of forming the pair shown or one other base pair (including GU pairs). Base pairs are highlighted in deep/mid/light green as all/all but one/all but two sequences are capable of forming the pair shown or either of another two base pairs (including GU pairs). RNAalifold was used with input options disallowing lonely pairs, allowing G-quadruplexes, and with the ribosum scoring matrix enabled.

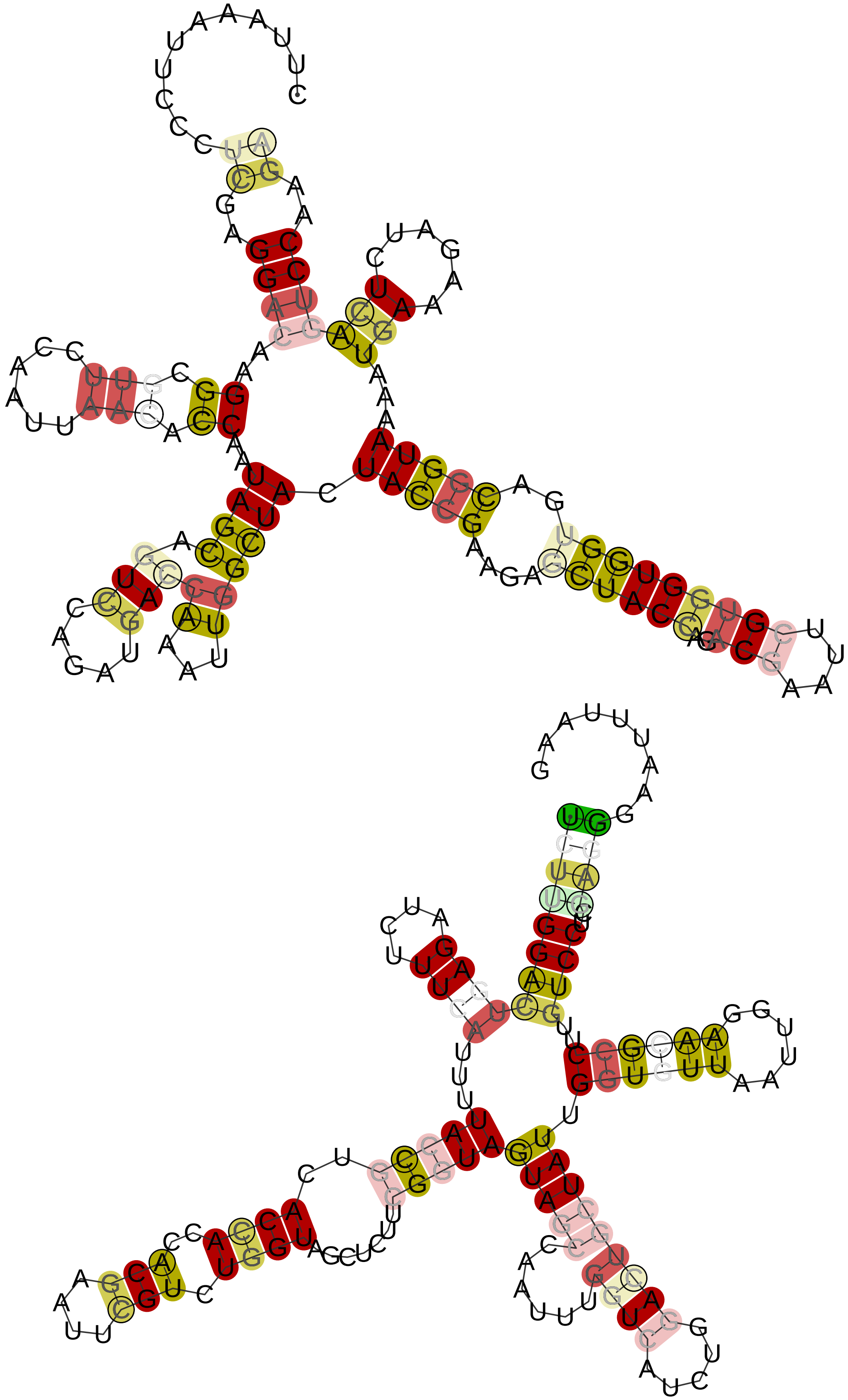

Fig. S40. Output of RNAalifold analysis of the conserved region found 1st in analysis of the N region of SARS-CoV-2, NC_045512.2 nucleotide location 28463–28594 (top: forward sense; bottom: reverse complement). Prior to folding, aligned sequences used in the main analysis were deduplicated so that one example of each observed primary structure for the conserved region was used as input to the algorithm. Base pairs are highlighted in deep/mid/light red as all/all but one/all but two sequences are capable of forming the pairs shown. Base pairs are highlighted in deep/mid/light yellow as all/all but one/all but two sequences are capable of forming the pair shown or one other base pair (including GU pairs). Base pairs are highlighted in deep/mid/light green as all/all but one/all but two sequences are capable of forming the pair shown or either of another two base pairs (including GU pairs). RNAalifold was used with input options disallowing lonely pairs, allowing G-quadruplexes, and with the ribosum scoring matrix enabled.

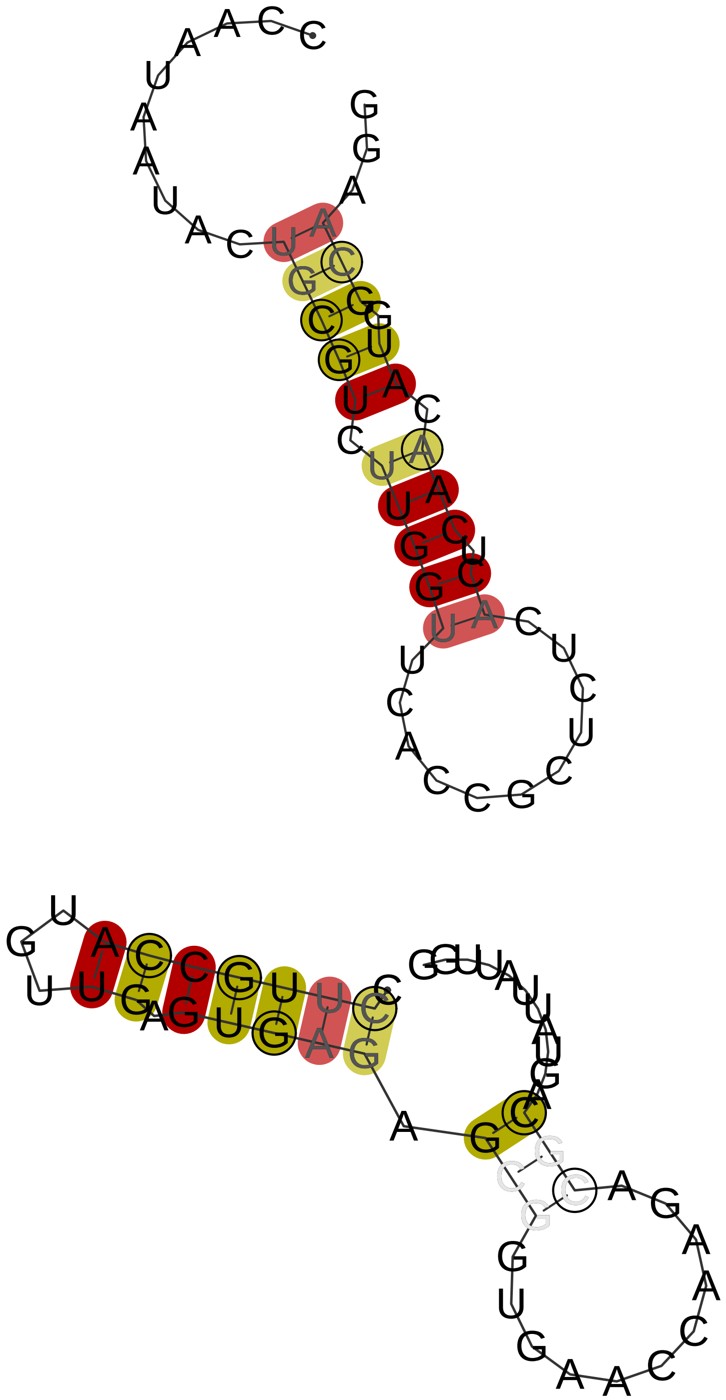

Fig. S41. Output of RNAalifold analysis of the conserved region found 2nd in analysis of the 9a region of SARS-CoV-2, NC_045512.2 nucleotide location 28410–28457 (top: forward sense; bottom: reverse complement). Prior to folding, aligned sequences used in the main analysis were deduplicated so that one example of each observed primary structure for the conserved region was used as input to the algorithm. Base pairs are highlighted in deep/mid/light red as all/all but one/all but two sequences are capable of forming the pairs shown. Base pairs are highlighted in deep/mid/light yellow as all/all but one/all but two sequences are capable of forming the pair shown or one other base pair (including GU pairs). Base pairs are highlighted in deep/mid/light green as all/all but one/all but two sequences are capable of forming the pair shown or either of another two base pairs (including GU pairs). RNAalifold was used with input options disallowing lonely pairs, allowing G-quadruplexes, and with the ribosum scoring matrix enabled.

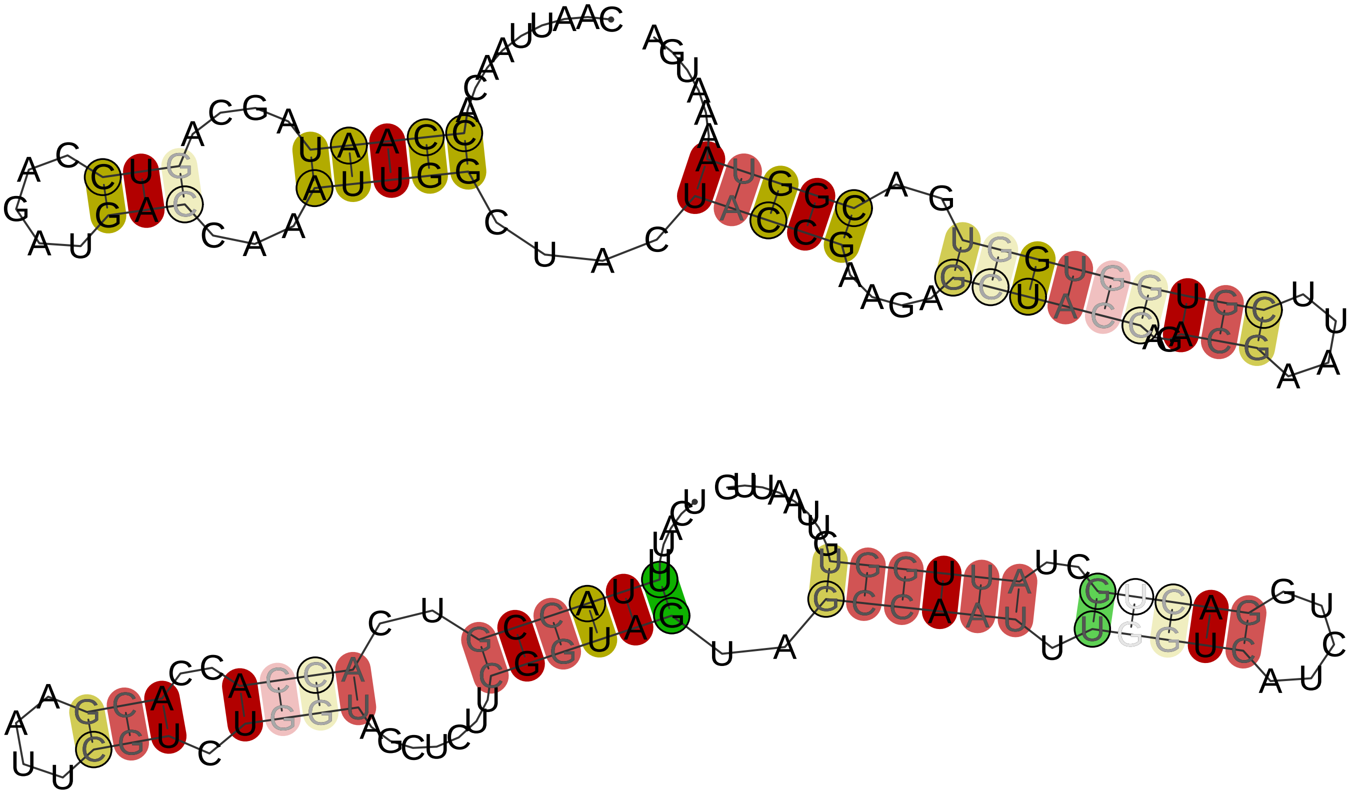

Fig. S42. Output of RNAalifold analysis of the conserved region found 1st in analysis of the 9a region of SARS-CoV-2, NC_045512.2 nucleotide location 28488–28577 (top: forward sense; bottom: reverse complement). Prior to folding, aligned sequences used in the main analysis were deduplicated so that one example of each observed primary structure for the conserved region was used as input to the algorithm. Base pairs are highlighted in deep/mid/light red as all/all but one/all but two sequences are capable of forming the pairs shown. Base pairs are highlighted in deep/mid/light yellow as all/all but one/all but two sequences are capable of forming the pair shown or one other base pair (including GU pairs). Base pairs are highlighted in deep/mid/light green as all/all but one/all but two sequences are capable of forming the pair shown or either of another two base pairs (including GU pairs). RNAalifold was used with input options disallowing lonely pairs, allowing G-quadruplexes, and with the ribosum scoring matrix enabled.

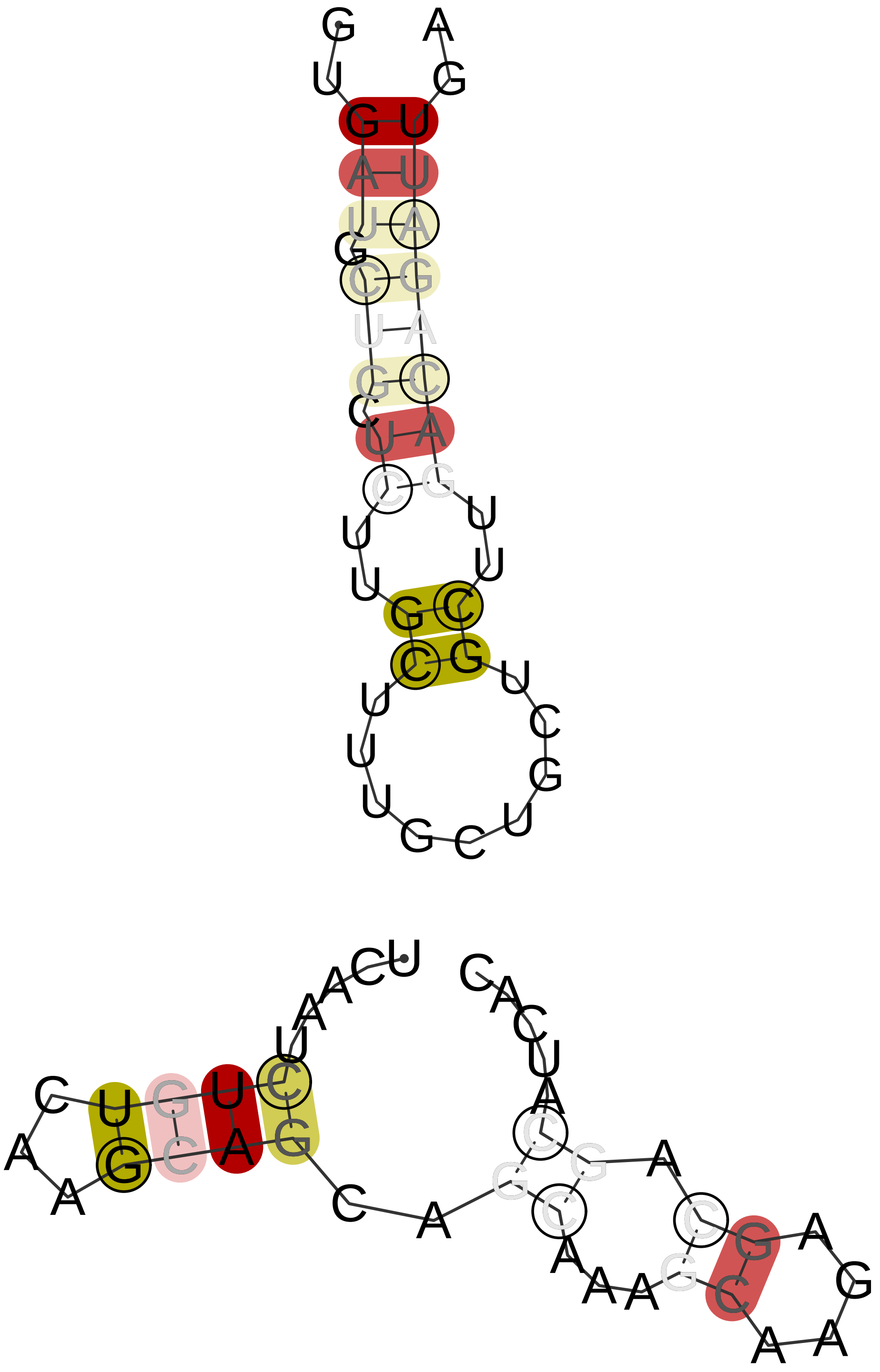

Fig. S43. Output of RNAalifold analysis of the conserved region found 1st in analysis of the 9b region of SARS-CoV-2, NC_045512.2 nucleotide location 28917–28955 (top: forward sense; bottom: reverse complement). Prior to folding, aligned sequences used in the main analysis were deduplicated so that one example of each observed primary structure for the conserved region was used as input to the algorithm. Base pairs are highlighted in deep/mid/light red as all/all but one/all but two sequences are capable of forming the pairs shown. Base pairs are highlighted in deep/mid/light yellow as all/all but one/all but two sequences are capable of forming the pair shown or one other base pair (including GU pairs). Base pairs are highlighted in deep/mid/light green as all/all but one/all but two sequences are capable of forming the pair shown or either of another two base pairs (including GU pairs). RNAalifold was used with input options disallowing lonely pairs, allowing G-quadruplexes, and with the ribosum scoring matrix enabled.

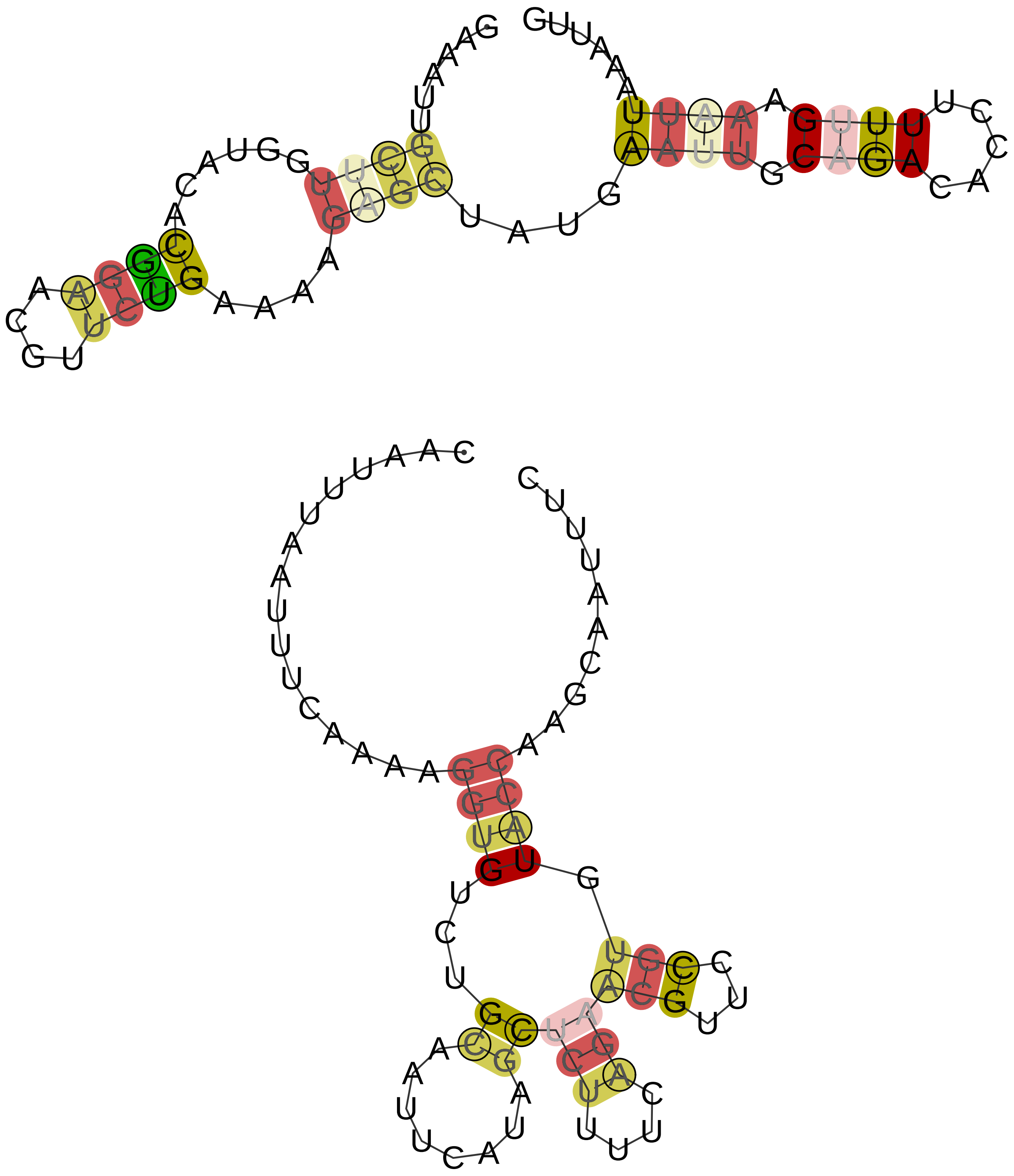

Fig. S44. Output of RNAalifold analysis of the conserved region found 1st in analysis of the nsp2 region of SARS-CoV-2, NC_045512.2 nucleotide location 974–1042 (top: forward sense; bottom: reverse complement). Prior to folding, aligned sequences used in the main analysis were deduplicated so that one example of each observed primary structure for the conserved region was used as input to the algorithm. Base pairs are highlighted in deep/mid/light red as all/all but one/all but two sequences are capable of forming the pairs shown. Base pairs are highlighted in deep/mid/light yellow as all/all but one/all but two sequences are capable of forming the pair shown or one other base pair (including GU pairs). Base pairs are highlighted in deep/mid/light green as all/all but one/all but two sequences are capable of forming the pair shown or either of another two base pairs (including GU pairs). RNAalifold was used with input options disallowing lonely pairs, allowing G-quadruplexes, and with the ribosum scoring matrix enabled.

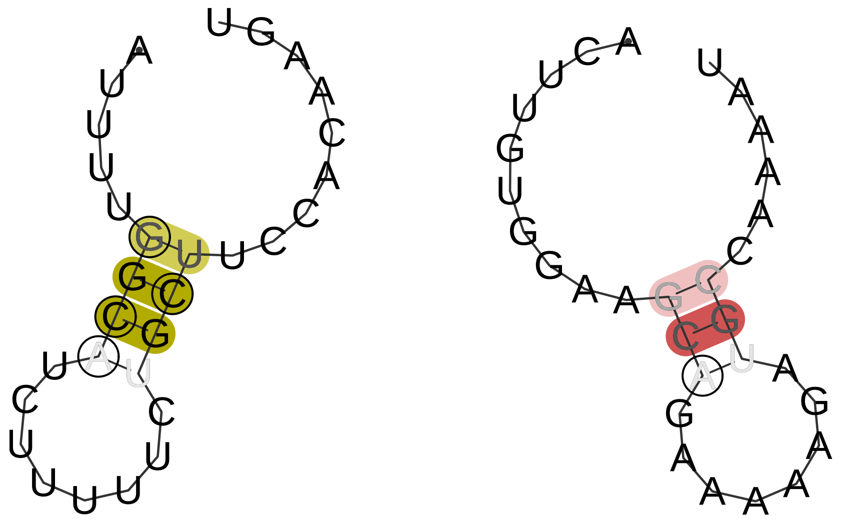

Fig. S45. Output of RNAalifold analysis of the conserved region found 2nd in analysis of the nsp2 region of SARS-CoV-2, NC_045512.2 nucleotide location 1688–1720 (left: forward sense; right: reverse complement). Prior to folding, aligned sequences used in the main analysis were deduplicated so that one example of each observed primary structure for the conserved region was used as input to the algorithm. Base pairs are highlighted in deep/mid/light red as all/all but one/all but two sequences are capable of forming the pairs shown. Base pairs are highlighted in deep/mid/light yellow as all/all but one/all but two sequences are capable of forming the pair shown or one other base pair (including GU pairs). Base pairs are highlighted in deep/mid/light green as all/all but one/all but two sequences are capable of forming the pair shown or either of another two base pairs (including GU pairs). RNAalifold was used with input options disallowing lonely pairs, allowing G-quadruplexes, and with the ribosum scoring matrix enabled.

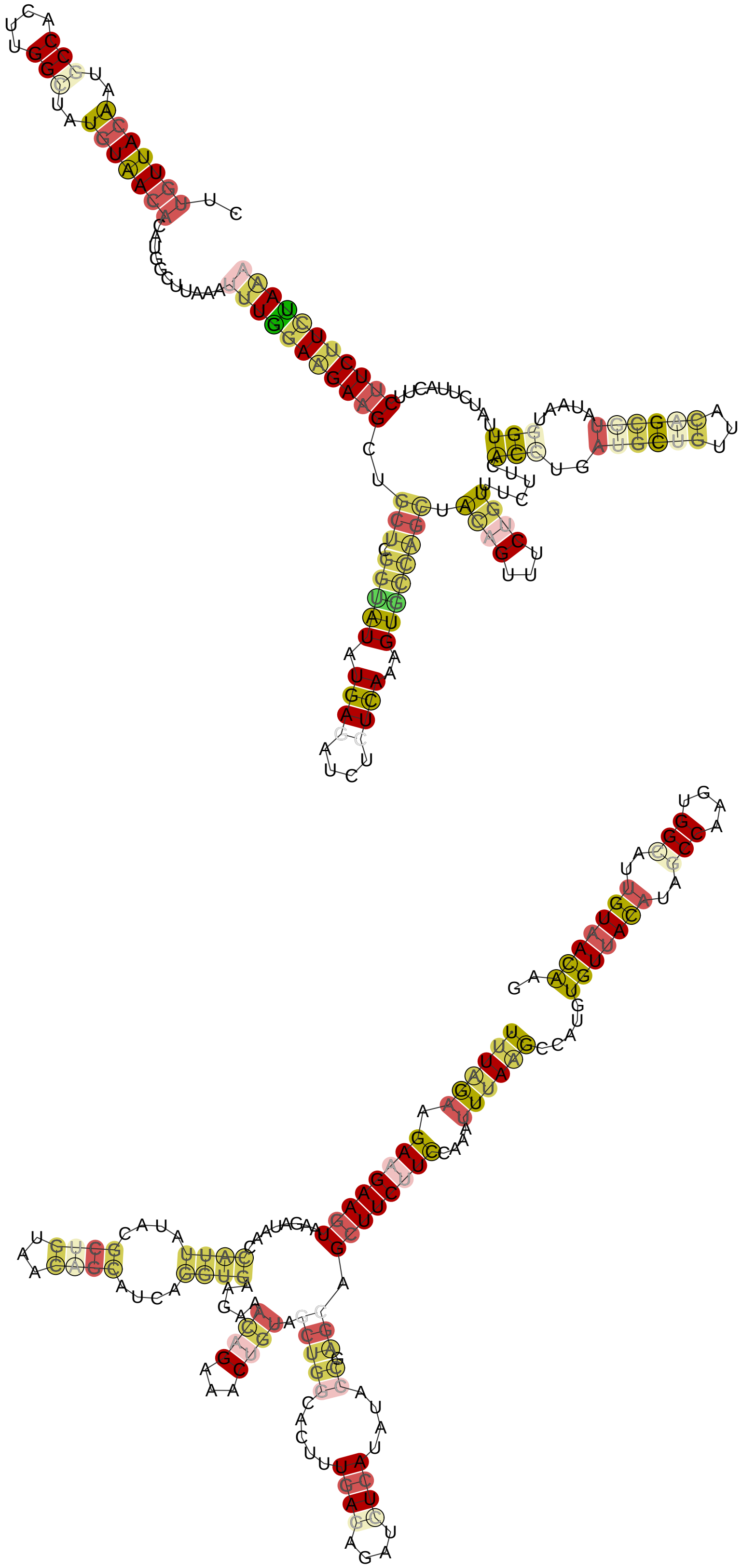

Fig. S46. Output of RNAalifold analysis of the conserved region found 3rd in analysis of the nsp3/PLpro region of SARS-CoV-2, NC_045512.2 nucleotide location 4598–4750 (top: forward sense; bottom: reverse complement). Prior to folding, aligned sequences used in the main analysis were deduplicated so that one example of each observed primary structure for the conserved region was used as input to the algorithm. Base pairs are highlighted in deep/mid/light red as all/all but one/all but two sequences are capable of forming the pairs shown. Base pairs are highlighted in deep/mid/light yellow as all/all but one/all but two sequences are capable of forming the pair shown or one other base pair (including GU pairs). Base pairs are highlighted in deep/mid/light green as all/all but one/all but two sequences are capable of forming the pair shown or either of another two base pairs (including GU pairs). RNAalifold was used with input options disallowing lonely pairs, allowing G-quadruplexes, and with the ribosum scoring matrix enabled.

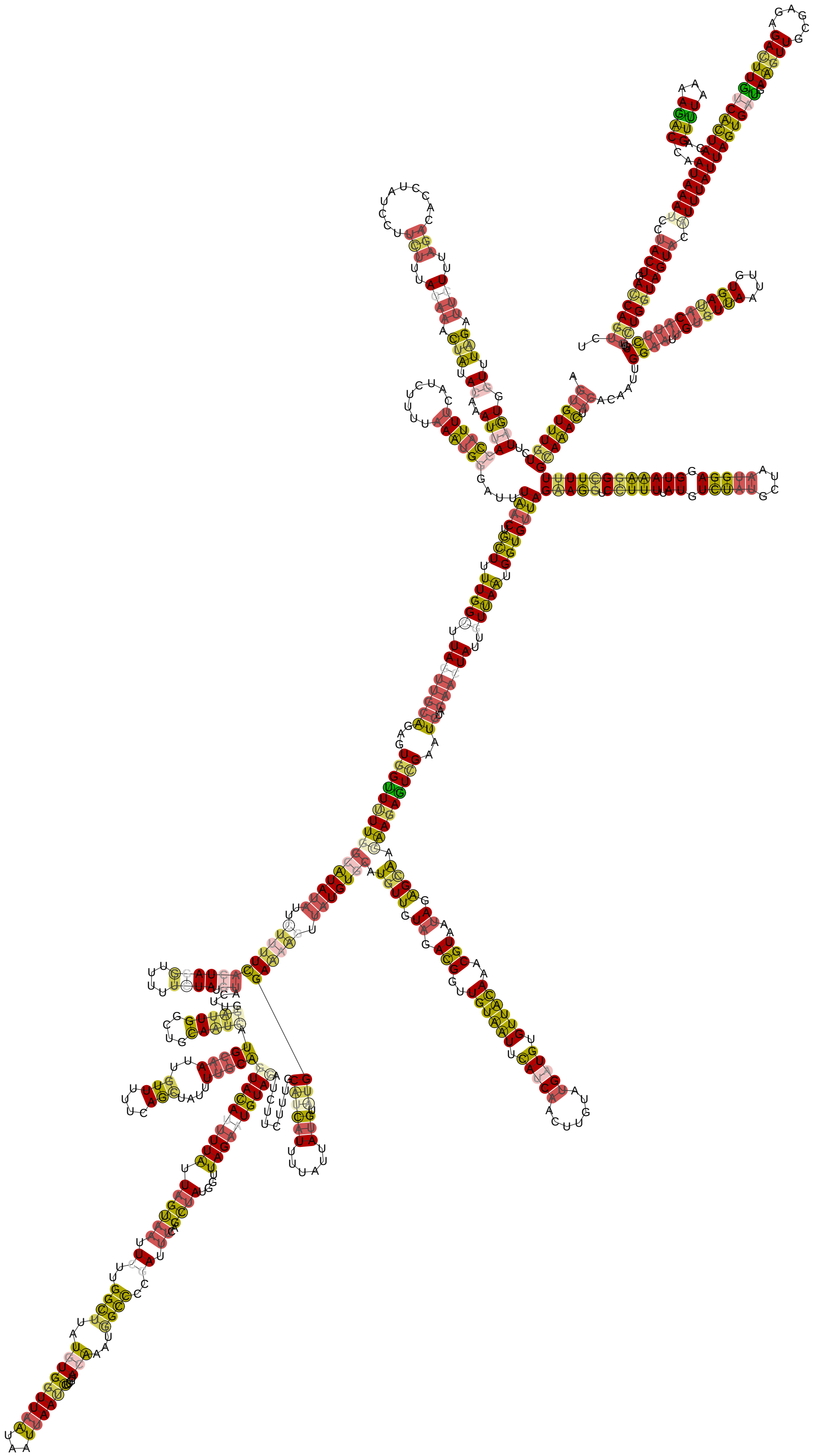

Fig. S47. Output of RNAalifold analysis of the conserved region found 2nd in analysis of the nsp3/PLpro region of SARS-CoV-2, NC_045512.2 nucleotide location 7127–7726. Prior to folding, aligned sequences used in the main analysis were deduplicated so that one example of each observed primary structure for the conserved region was used as input to the algorithm. Base pairs are highlighted in deep/mid/light red as all/all but one/all but two sequences are capable of forming the pairs shown. Base pairs are highlighted in deep/mid/light yellow as all/all but one/all but two sequences are capable of forming the pair shown or one other base pair (including GU pairs). Base pairs are highlighted in deep/mid/light green as all/all but one/all but two sequences are capable of forming the pair shown or either of another two base pairs (including GU pairs). RNAalifold was used with input options disallowing lonely pairs, allowing G-quadruplexes, and with the ribosum scoring matrix enabled.

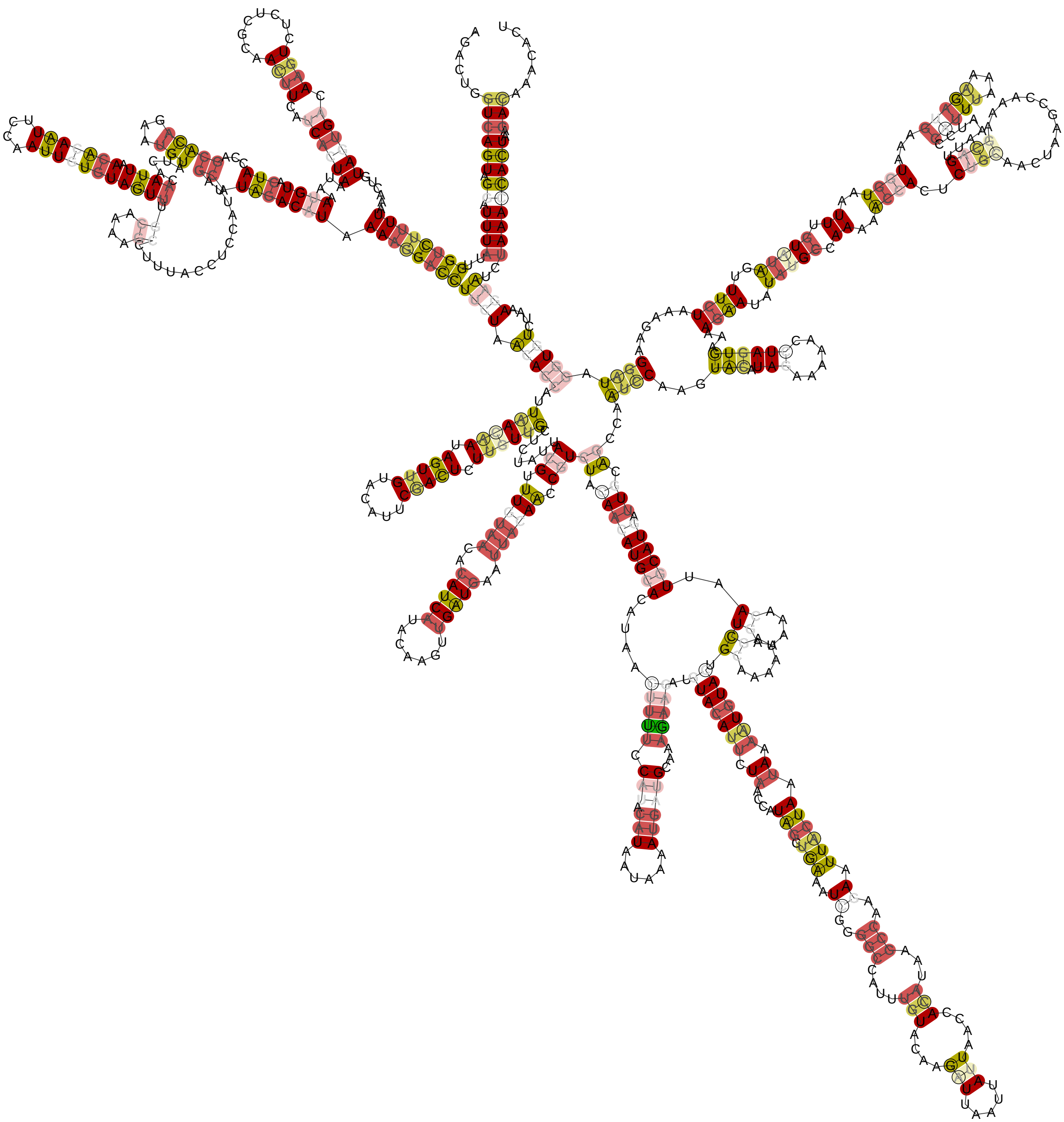

Fig. S48. Output of RNAalifold analysis of the reverse complement of the conserved region found 2nd in analysis of the nsp3/PLpro region of SARS-CoV-2, NC_045512.2 nucleotide location 7127–7726. Prior to folding, aligned sequences used in the main analysis were deduplicated so that one example of each observed primary structure for the conserved region was used as input to the algorithm. Base pairs are highlighted in deep/mid/light red as all/all but one/all but two sequences are capable of forming the pairs shown. Base pairs are highlighted in deep/mid/light yellow as all/all but one/all but two sequences are capable of forming the pair shown or one other base pair (including GU pairs). Base pairs are highlighted in deep/mid/light green as all/all but one/all but two sequences are capable of forming the pair shown or either of another two base pairs (including GU pairs). RNAalifold was used with input options disallowing lonely pairs, allowing G-quadruplexes, and with the ribosum scoring matrix enabled.

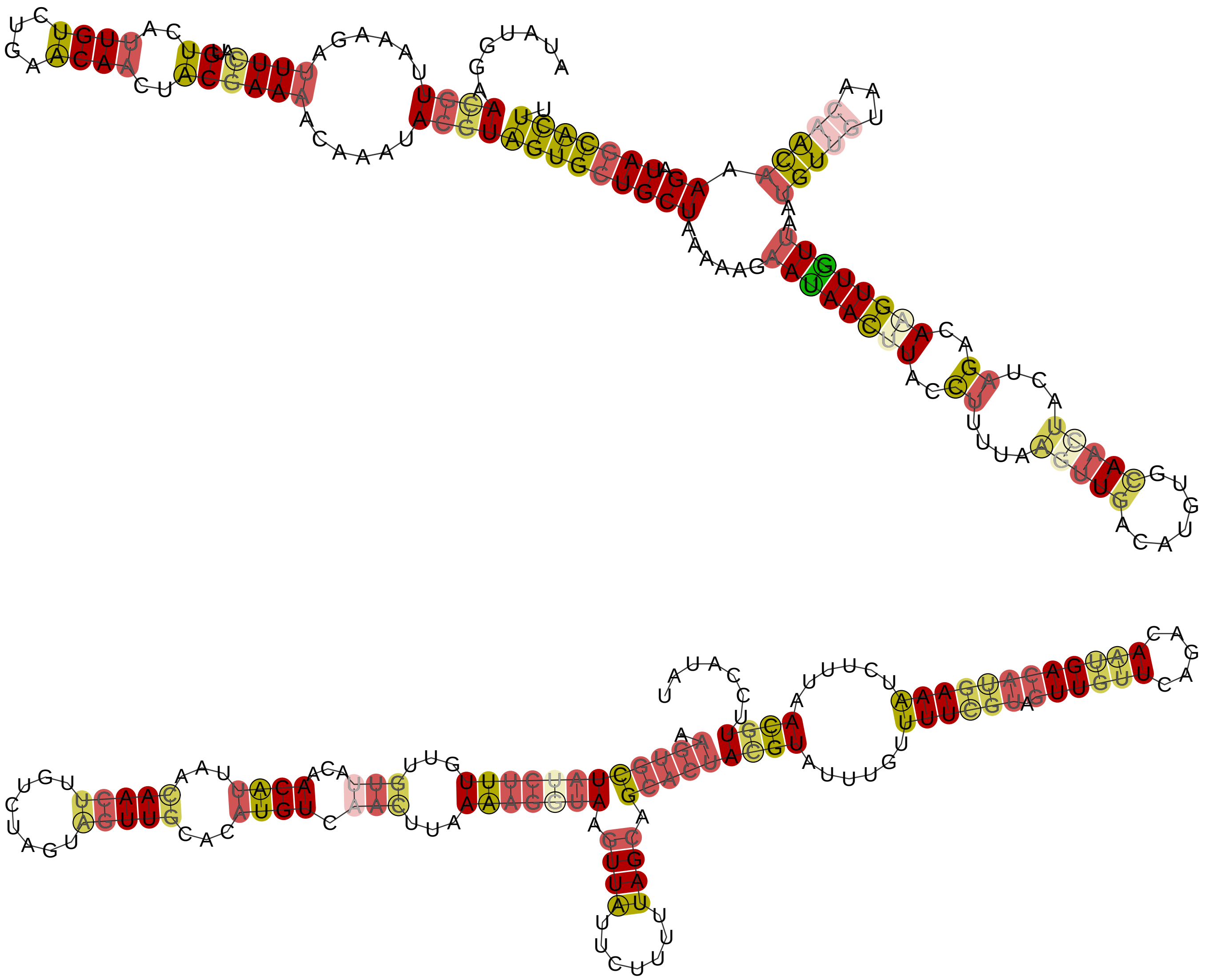

Fig. S49. Output of RNAalifold analysis of the conserved region found 1st in analysis of the nsp3/PLpro region of SARS-CoV-2, NC_045512.2 nucleotide location 8396–8545 (top: forward sense; bottom: reverse complement). Prior to folding, aligned sequences used in the main analysis were deduplicated so that one example of each observed primary structure for the conserved region was used as input to the algorithm. Base pairs are highlighted in deep/mid/light red as all/all but one/all but two sequences are capable of forming the pairs shown. Base pairs are highlighted in deep/mid/light yellow as all/all but one/all but two sequences are capable of forming the pair shown or one other base pair (including GU pairs). Base pairs are highlighted in deep/mid/light green as all/all but one/all but two sequences are capable of forming the pair shown or either of another two base pairs (including GU pairs). RNAalifold was used with input options disallowing lonely pairs, allowing G-quadruplexes, and with the ribosum scoring matrix enabled.

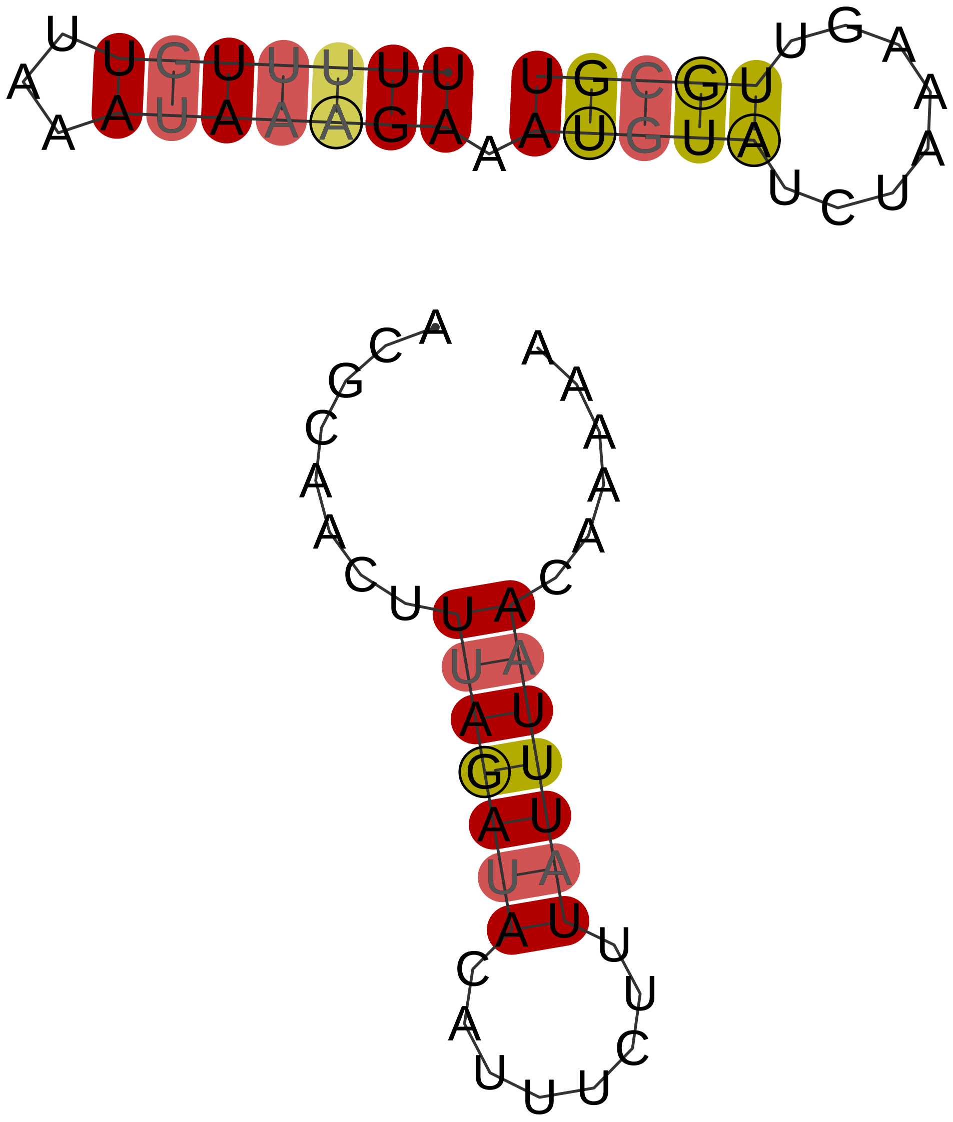

Fig. S50. Output of RNAalifold analysis of the conserved region found 1st in analysis of the nsp4 region of SARS-CoV-2, NC_045512.2 nucleotide location 9812–9847 (top: forward sense; bottom: reverse complement). Prior to folding, aligned sequences used in the main analysis were deduplicated so that one example of each observed primary structure for the conserved region was used as input to the algorithm. Base pairs are highlighted in deep/mid/light red as all/all but one/all but two sequences are capable of forming the pairs shown. Base pairs are highlighted in deep/mid/light yellow as all/all but one/all but two sequences are capable of forming the pair shown or one other base pair (including GU pairs). Base pairs are highlighted in deep/mid/light green as all/all but one/all but two sequences are capable of forming the pair shown or either of another two base pairs (including GU pairs). RNAalifold was used with input options disallowing lonely pairs, allowing G-quadruplexes, and with the ribosum scoring matrix enabled.

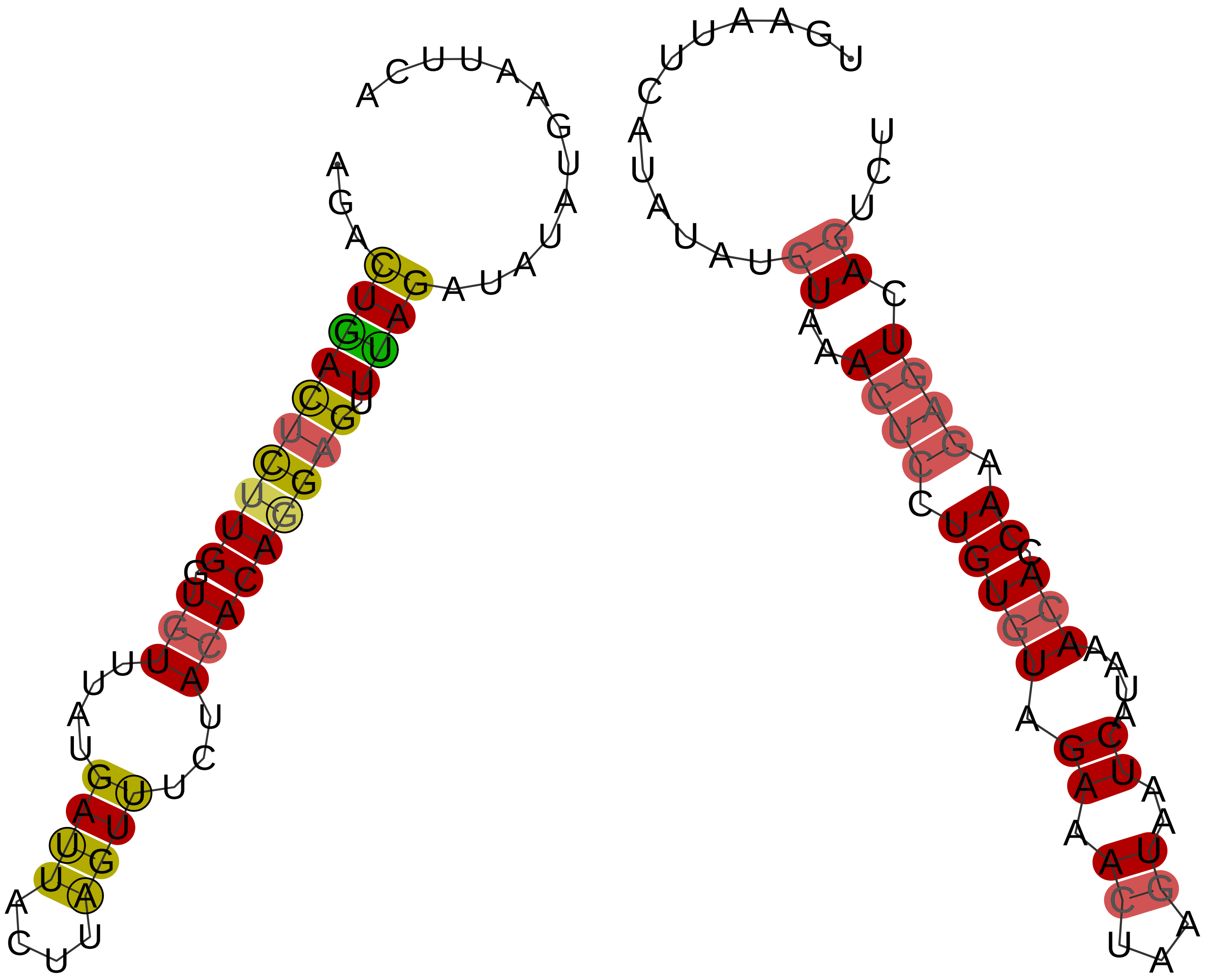

Fig. S51. Output of RNAalifold analysis of the conserved region found 1st in analysis of the nsp6 region of SARS-CoV-2, NC_045512.2 nucleotide location 11675–11740 (left: forward sense; right: reverse complement). Prior to folding, aligned sequences used in the main analysis were deduplicated so that one example of each observed primary structure for the conserved region was used as input to the algorithm. Base pairs are highlighted in deep/mid/light red as all/all but one/all but two sequences are capable of forming the pairs shown. Base pairs are highlighted in deep/mid/light yellow as all/all but one/all but two sequences are capable of forming the pair shown or one other base pair (including GU pairs). Base pairs are highlighted in deep/mid/light green as all/all but one/all but two sequences are capable of forming the pair shown or either of another two base pairs (including GU pairs). RNAalifold was used with input options disallowing lonely pairs, allowing G-quadruplexes, and with the ribosum scoring matrix enabled.

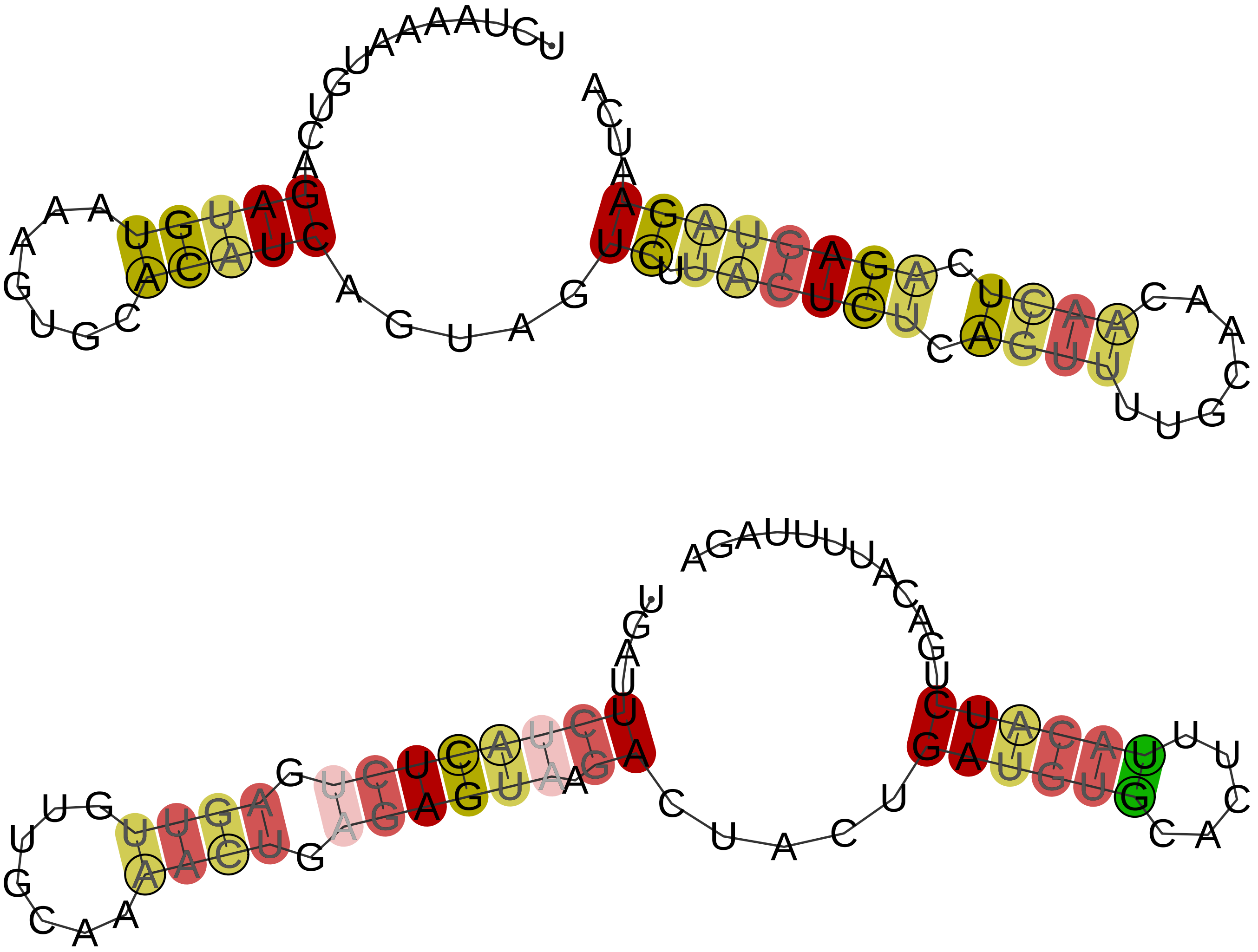

Fig. S52. Output of RNAalifold analysis of the conserved region found 1st in analysis of the nsp7 region of SARS-CoV-2, NC_045512.2 nucleotide location 11843–11914 (top: forward sense; bottom: reverse complement). Prior to folding, aligned sequences used in the main analysis were deduplicated so that one example of each observed primary structure for the conserved region was used as input to the algorithm. Base pairs are highlighted in deep/mid/light red as all/all but one/all but two sequences are capable of forming the pairs shown. Base pairs are highlighted in deep/mid/light yellow as all/all but one/all but two sequences are capable of forming the pair shown or one other base pair (including GU pairs). Base pairs are highlighted in deep/mid/light green as all/all but one/all but two sequences are capable of forming the pair shown or either of another two base pairs (including GU pairs). RNAalifold was used with input options disallowing lonely pairs, allowing G-quadruplexes, and with the ribosum scoring matrix enabled.

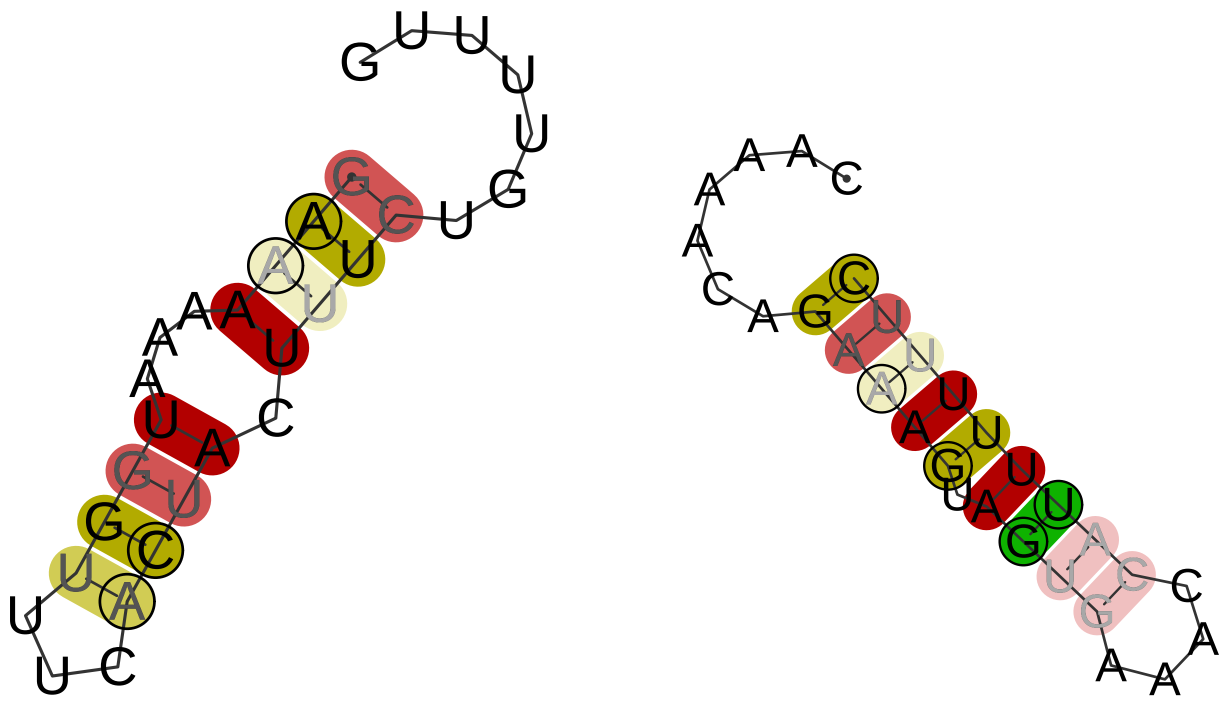

Fig. S53. Output of RNAalifold analysis of the conserved region found 2nd in analysis of the nsp7 region of SARS-CoV-2, NC_045512.2 nucleotide location 11987–12019 (left: forward sense; right: reverse complement). Prior to folding, aligned sequences used in the main analysis were deduplicated so that one example of each observed primary structure for the conserved region was used as input to the algorithm. Base pairs are highlighted in deep/mid/light red as all/all but one/all but two sequences are capable of forming the pairs shown. Base pairs are highlighted in deep/mid/light yellow as all/all but one/all but two sequences are capable of forming the pair shown or one other base pair (including GU pairs). Base pairs are highlighted in deep/mid/light green as all/all but one/all but two sequences are capable of forming the pair shown or either of another two base pairs (including GU pairs). RNAalifold was used with input options disallowing lonely pairs, allowing G-quadruplexes, and with the ribosum scoring matrix enabled.

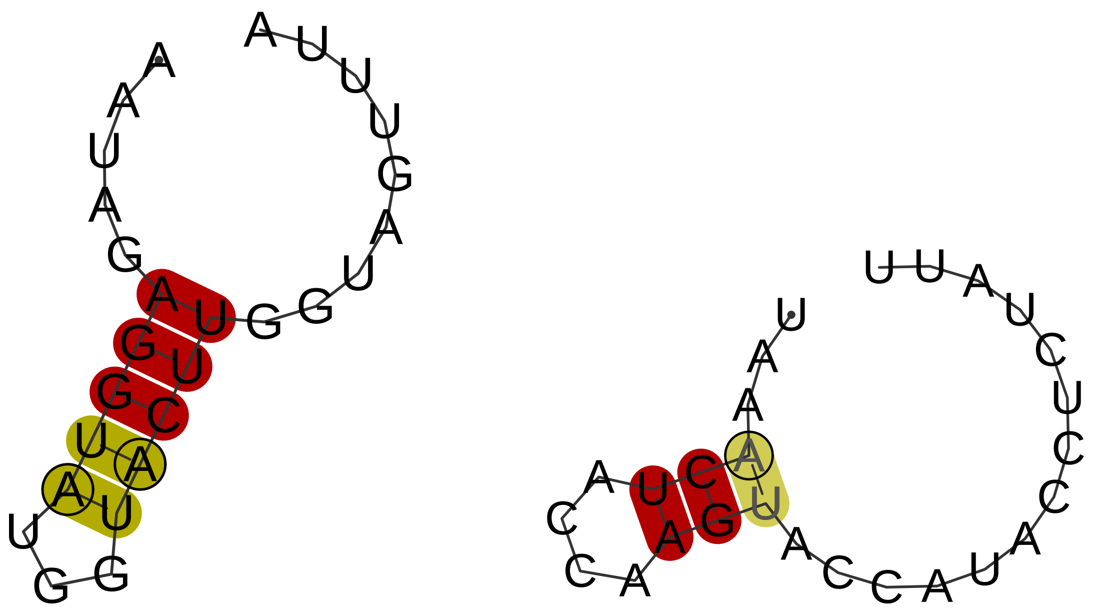

Fig. S54. Output of RNAalifold analysis of the conserved region found 1st in analysis of the nsp9 region of SARS-CoV-2, NC_045512.2 nucleotide location 12977–13003 (left: forward sense; right: reverse complement). Prior to folding, aligned sequences used in the main analysis were deduplicated so that one example of each observed primary structure for the conserved region was used as input to the algorithm. Base pairs are highlighted in deep/mid/light red as all/all but one/all but two sequences are capable of forming the pairs shown. Base pairs are highlighted in deep/mid/light yellow as all/all but one/all but two sequences are capable of forming the pair shown or one other base pair (including GU pairs). Base pairs are highlighted in deep/mid/light green as all/all but one/all but two sequences are capable of forming the pair shown or either of another two base pairs (including GU pairs). RNAalifold was used with input options disallowing lonely pairs, allowing G-quadruplexes, and with the ribosum scoring matrix enabled.

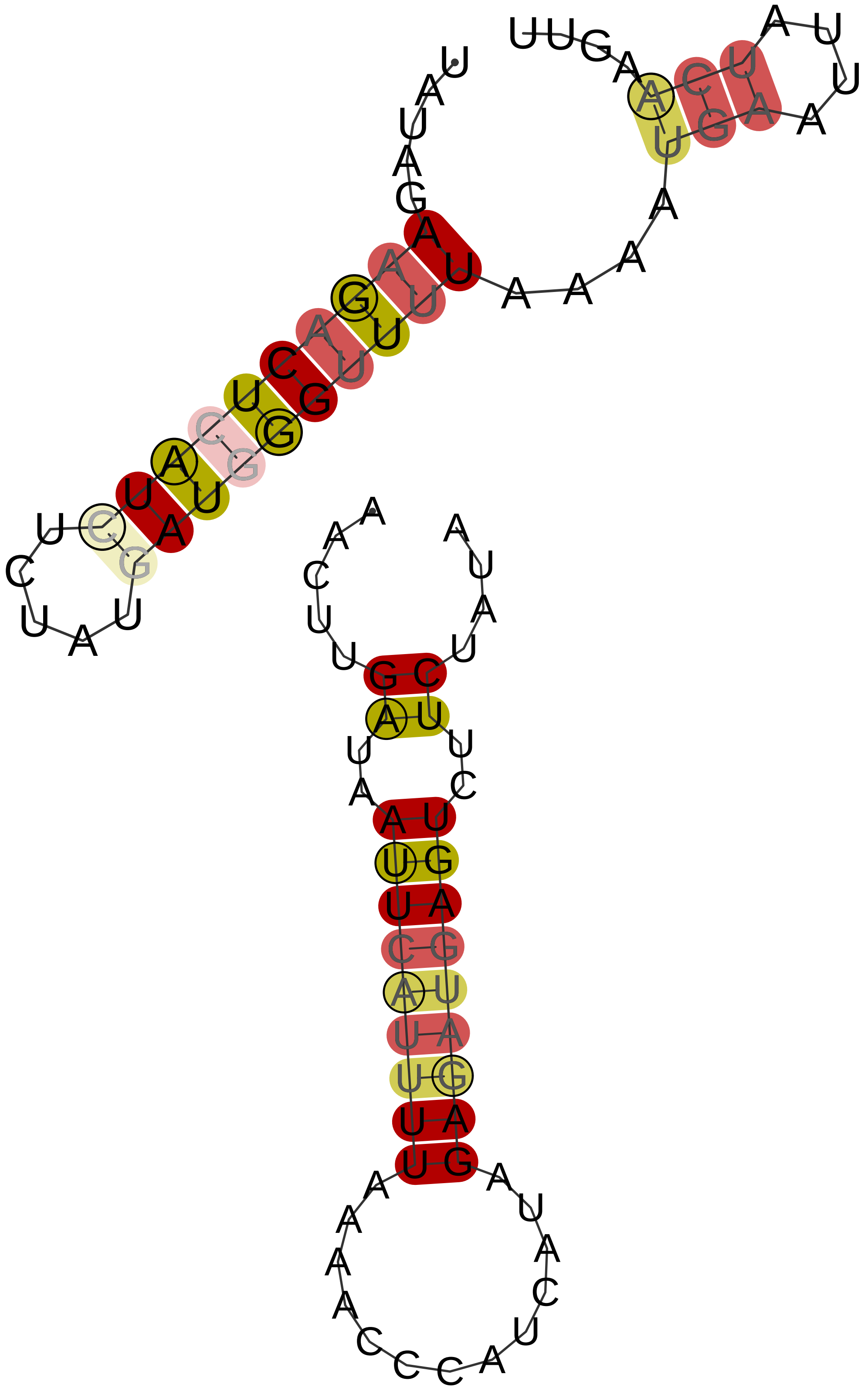

Fig. S55. Output of RNAalifold analysis of the conserved region found 2nd in analysis of the nsp14 region of SARS-CoV-2, NC_045512.2 nucleotide location 18190–18237 (top: forward sense; bottom: reverse complement). Prior to folding, aligned sequences used in the main analysis were deduplicated so that one example of each observed primary structure for the conserved region was used as input to the algorithm. Base pairs are highlighted in deep/mid/light red as all/all but one/all but two sequences are capable of forming the pairs shown. Base pairs are highlighted in deep/mid/light yellow as all/all but one/all but two sequences are capable of forming the pair shown or one other base pair (including GU pairs). Base pairs are highlighted in deep/mid/light green as all/all but one/all but two sequences are capable of forming the pair shown or either of another two base pairs (including GU pairs). RNAalifold was used with input options disallowing lonely pairs, allowing G-quadruplexes, and with the ribosum scoring matrix enabled.

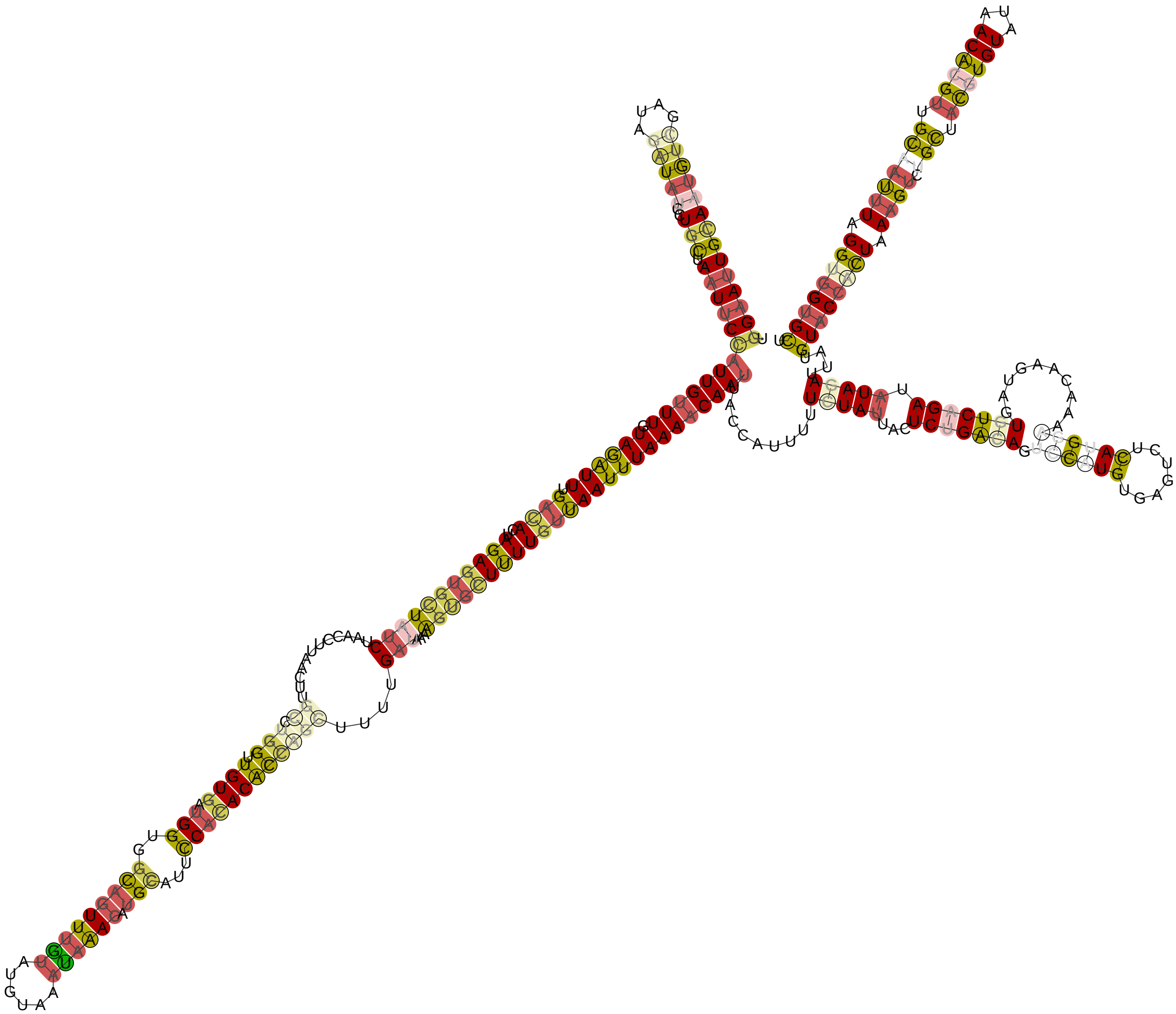

Fig. S56. Output of RNAalifold analysis of the conserved region found 1st in analysis of the nsp14 region of SARS-CoV-2, NC_045512.2 nucleotide location 19189–19485. Prior to folding, aligned sequences used in the main analysis were deduplicated so that one example of each observed primary structure for the conserved region was used as input to the algorithm. Base pairs are highlighted in deep/mid/light red as all/all but one/all but two sequences are capable of forming the pairs shown. Base pairs are highlighted in deep/mid/light yellow as all/all but one/all but two sequences are capable of forming the pair shown or one other base pair (including GU pairs). Base pairs are highlighted in deep/mid/light green as all/all but one/all but two sequences are capable of forming the pair shown or either of another two base pairs (including GU pairs). RNAalifold was used with input options disallowing lonely pairs, allowing G-quadruplexes, and with the ribosum scoring matrix enabled.

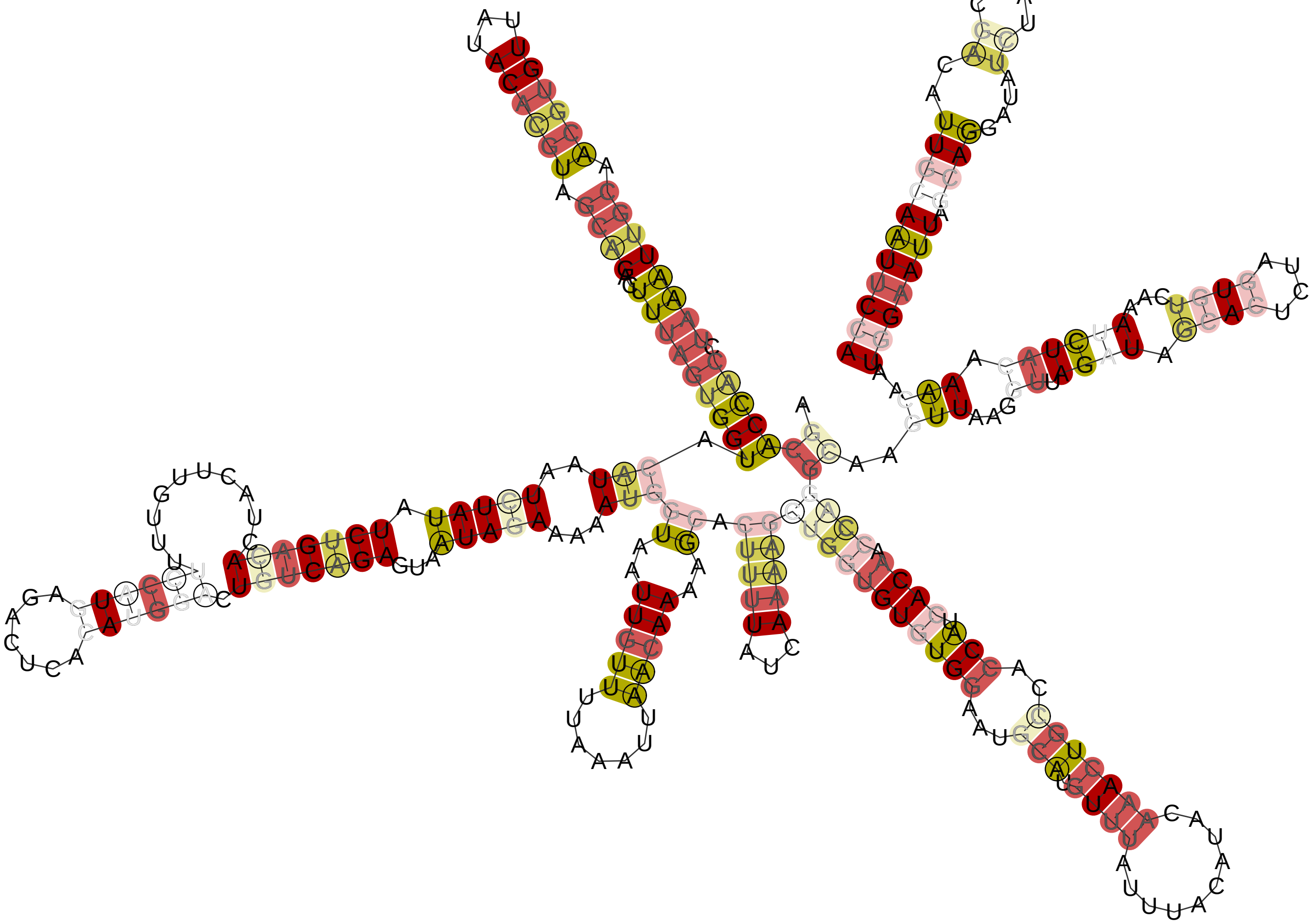

Fig. S57. Output of RNAalifold analysis of the reverse complement of the conserved region found 1st in analysis of the nsp14 region of SARS-CoV-2, NC_045512.2 nucleotide location 19189–19485. Prior to folding, aligned sequences used in the main analysis were deduplicated so that one example of each observed primary structure for the conserved region was used as input to the algorithm. Base pairs are highlighted in deep/mid/light red as all/all but one/all but two sequences are capable of forming the pairs shown. Base pairs are highlighted in deep/mid/light yellow as all/all but one/all but two sequences are capable of forming the pair shown or one other base pair (including GU pairs). Base pairs are highlighted in deep/mid/light green as all/all but one/all but two sequences are capable of forming the pair shown or either of another two base pairs (including GU pairs). RNAalifold was used with input options disallowing lonely pairs, allowing G-quadruplexes, and with the ribosum scoring matrix enabled.

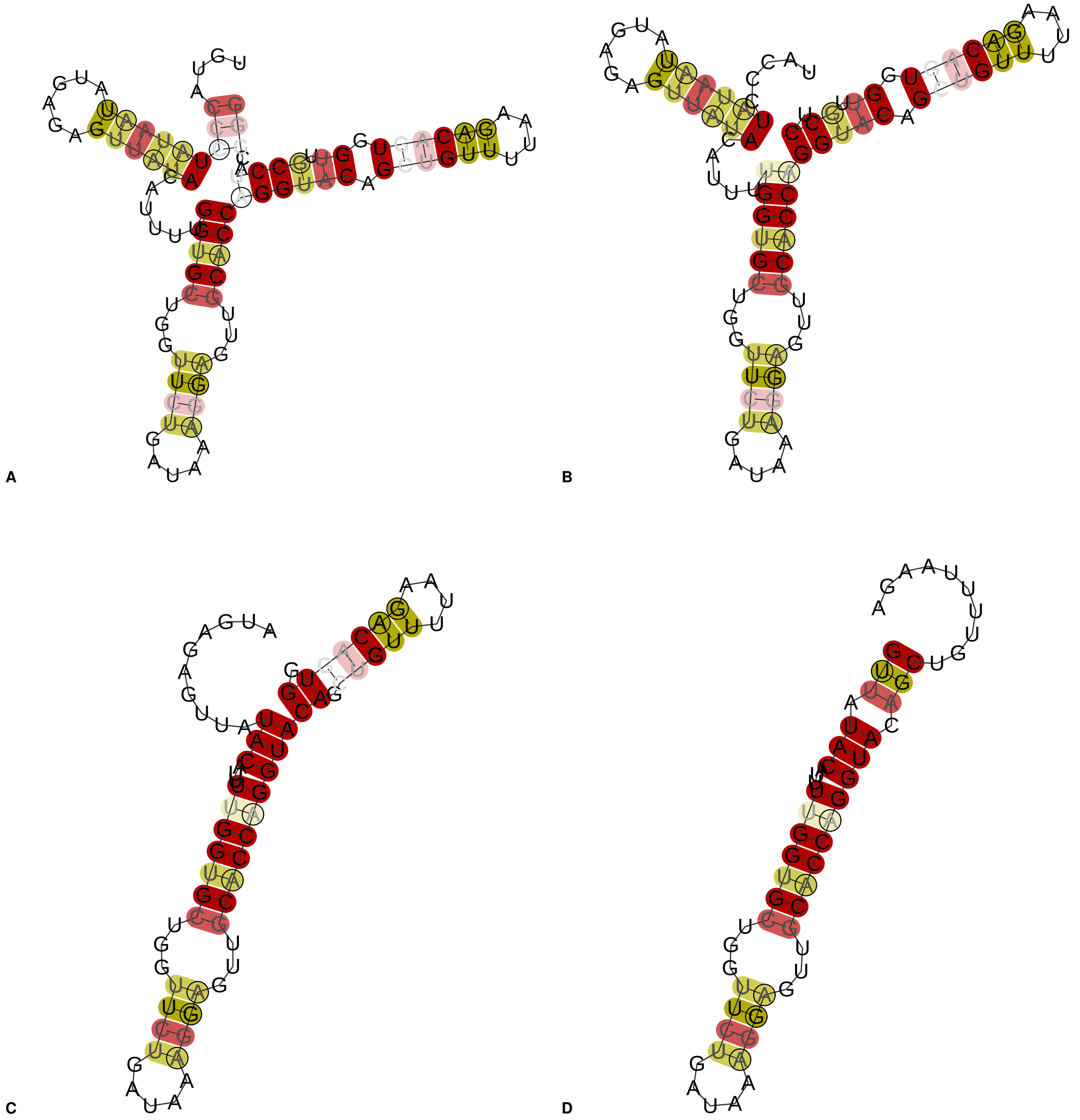

Fig. S58. Sensitivity analysis of predicted structures from the conserved region found 2nd in analysis of the nsp16 region of SARS-CoV-2, NC_045512.2 nucleotide location 20845–20928. (Note that the fold of the region is shown in Fig. 9 in the main text.) Prior to folding, aligned sequences used in the main analysis were deduplicated so that one example of each observed primary structure for the conserved region was used as input to the algorithm. Base pairs are highlighted in deep/mid/light red as all/all but one/all but two sequences are capable of forming the pairs shown. Base pairs are highlighted in deep/mid/light yellow as all/all but one/all but two sequences are capable of forming the pair shown or one other base pair (including GU pairs). Base pairs are highlighted in deep/mid/light green as all/all but one/all but two sequences are capable of forming the pair shown or either of another two base pairs (including GU pairs). RNAalifold was used with input options disallowing lonely pairs, allowing G-quadruplexes, and with the ribosum scoring matrix enabled. (**A**) Region 20838–20933. (**B**) Region 20840–20928. (**C**) Region 20851–20922. (**D**) Region 20857–20916. Addition of small numbers of nucleotides $5'$ and $3'$ of the conserved region does not disrupt the predicted structures; removal of nucleotides only disrupts the predicted structures when nucleotides in the structures themselves are removed, and the predicted structure that is left remains stable.

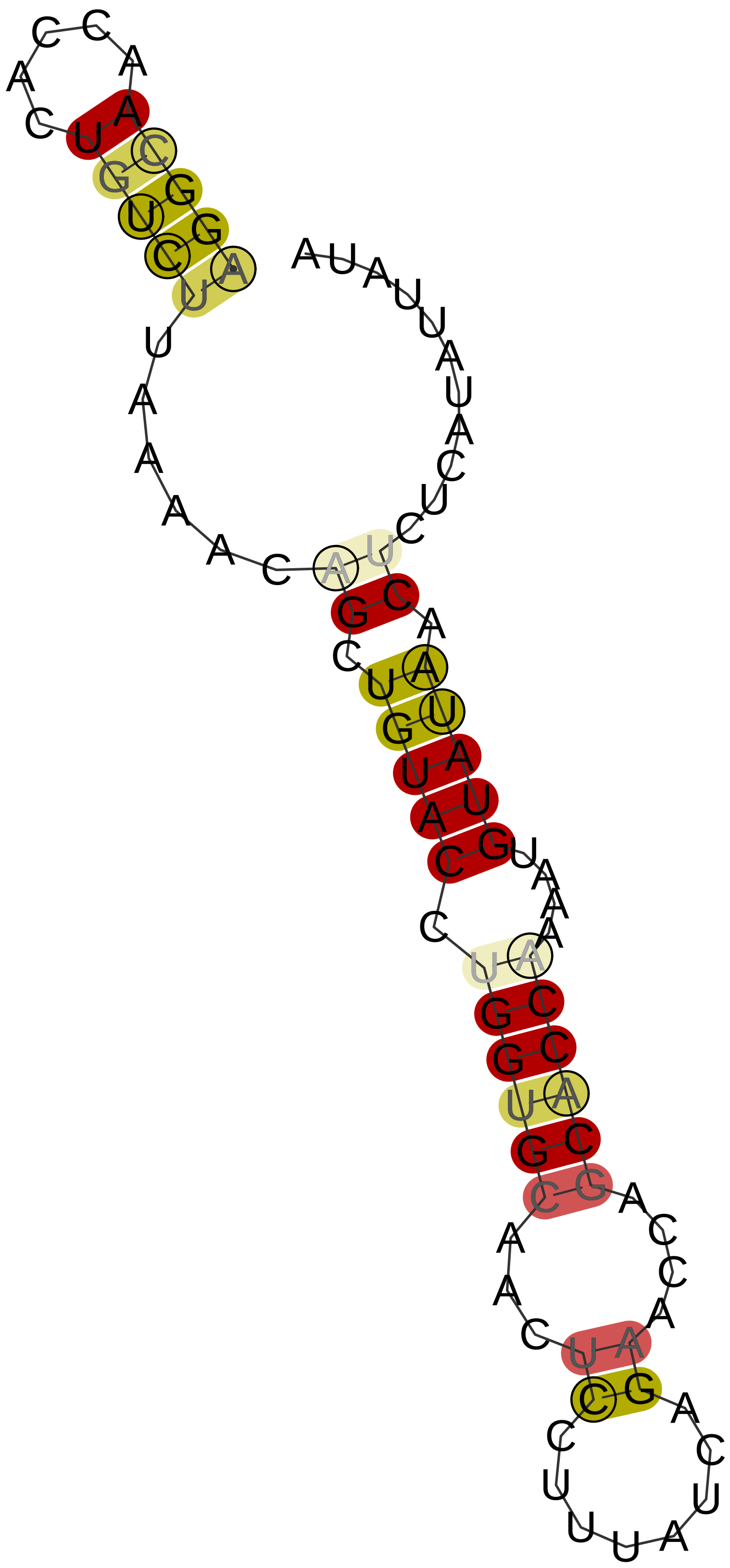

Fig. S59. Output of RNAalifold analysis of the reverse complement of the conserved region found 2nd in analysis of the nsp16 region of SARS-CoV-2, NC_045512.2 nucleotide location 20845–20928. (Note that the fold of the forward sense RNA is shown in Fig. 9 in the main text.) Prior to folding, aligned sequences used in the main analysis were deduplicated so that one example of each observed primary structure for the conserved region was used as input to the algorithm. Base pairs are highlighted in deep/mid/light red as all/all but one/all but two sequences are capable of forming the pairs shown. Base pairs are highlighted in deep/mid/light yellow as all/all but one/all but two sequences are capable of forming the pair shown or one other base pair (including GU pairs). Base pairs are highlighted in deep/mid/light green as all/all but one/all but two sequences are capable of forming the pair shown or either of another two base pairs (including GU pairs). RNAalifold was used with input options disallowing lonely pairs, allowing G-quadruplexes, and with the ribosum scoring matrix enabled.

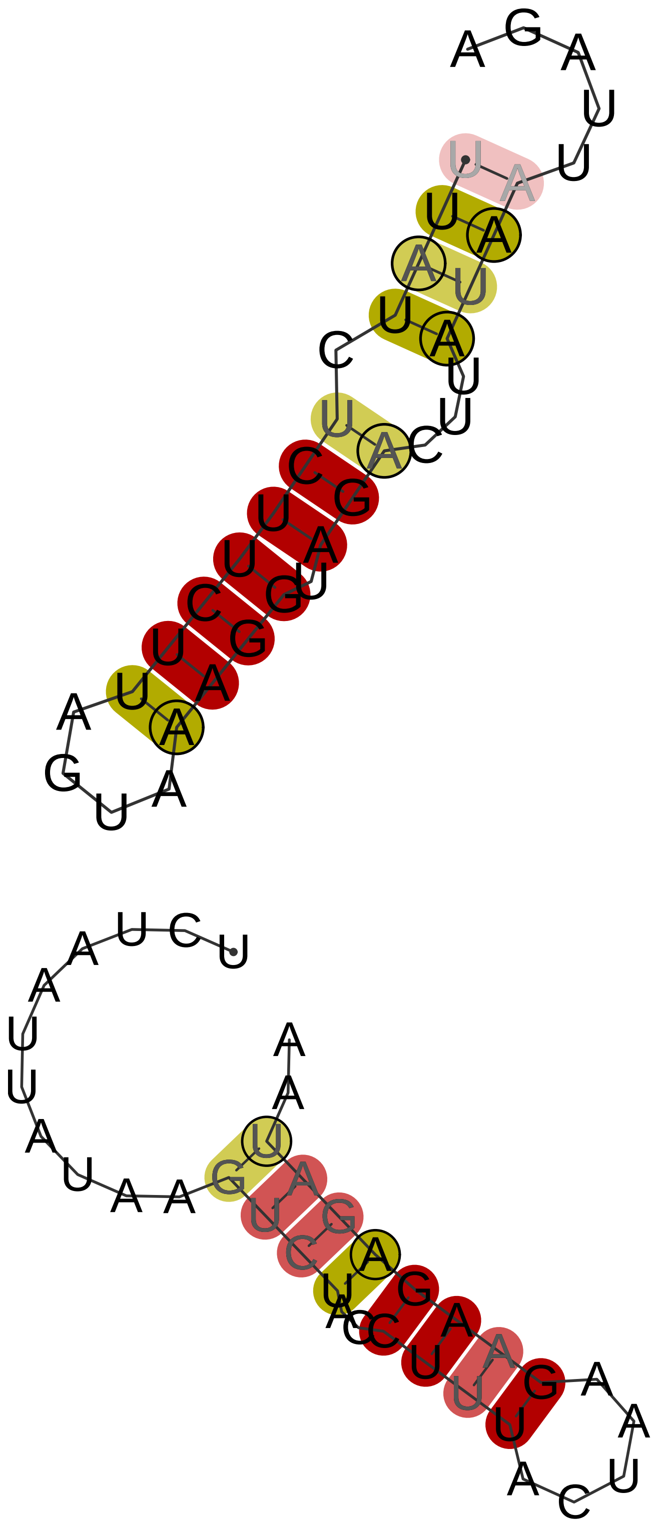

Fig. S60. Output of RNAalifold analysis of the conserved region found 1st in analysis of the nsp14 region of SARS-CoV-2, NC_045512.2 nucleotide location 21469–21507 (top: forward sense; bottom: reverse complement). Prior to folding, aligned sequences used in the main analysis were deduplicated so that one example of each observed primary structure for the conserved region was used as input to the algorithm. Base pairs are highlighted in deep/mid/light red as all/all but one/all but two sequences are capable of forming the pairs shown. Base pairs are highlighted in deep/mid/light yellow as all/all but one/all but two sequences are capable of forming the pair shown or one other base pair (including GU pairs). Base pairs are highlighted in deep/mid/light green as all/all but one/all but two sequences are capable of forming the pair shown or either of another two base pairs (including GU pairs). RNAalifold was used with input options disallowing lonely pairs, allowing G-quadruplexes, and with the ribosum scoring matrix enabled.

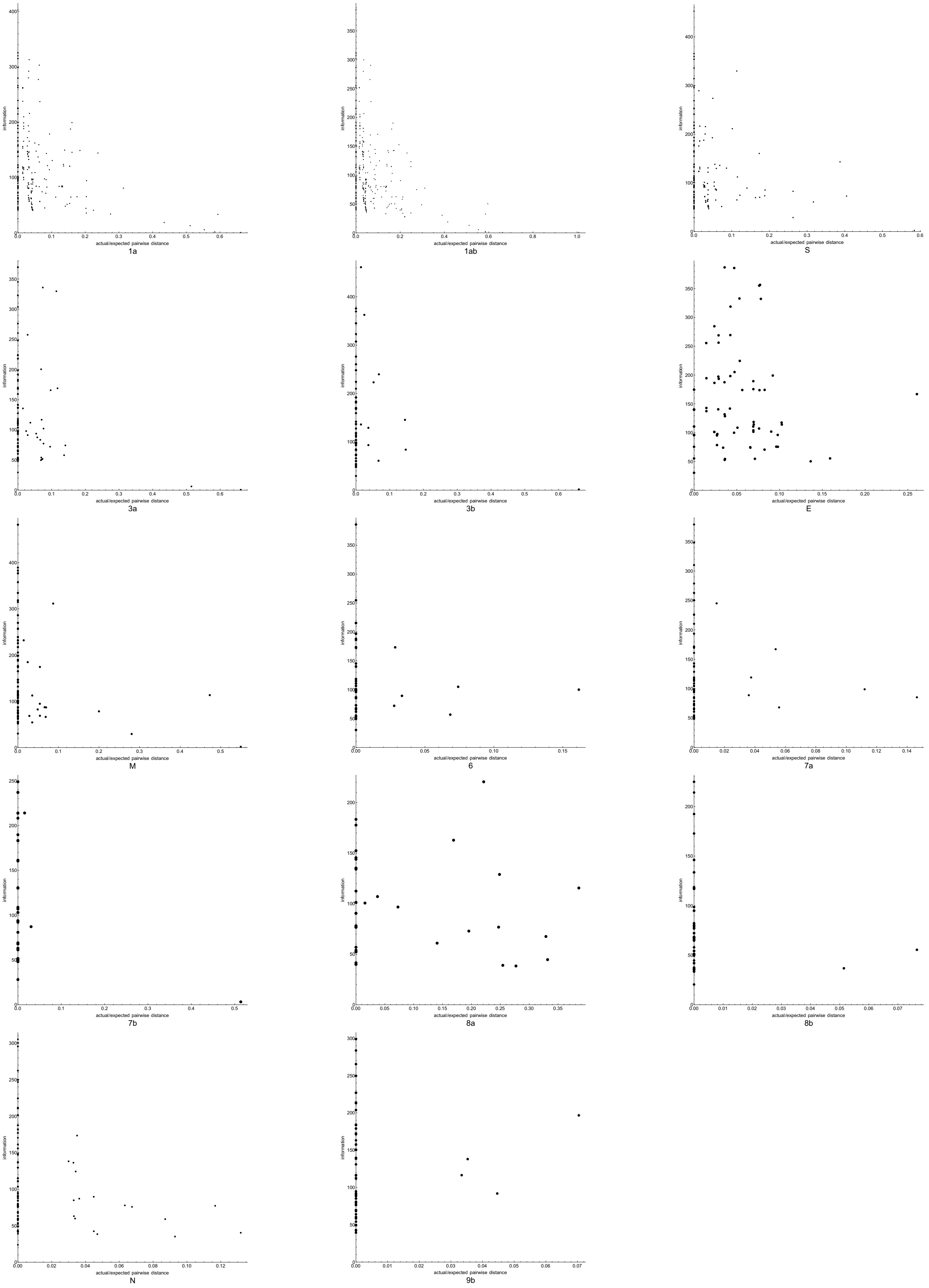

Fig. S61. Locus information versus variability for genes of SARS-CoV.

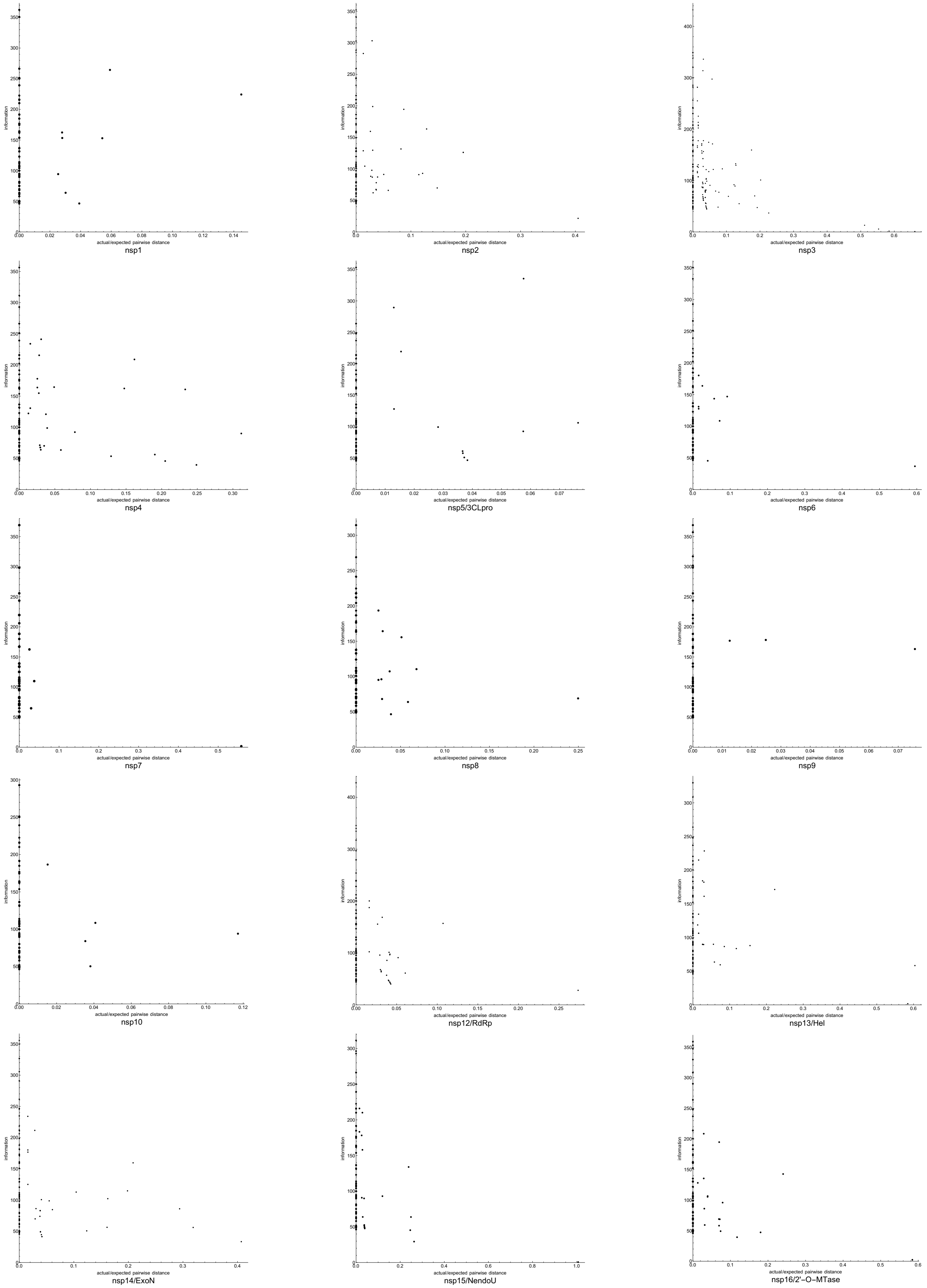

Fig. S62. Locus information versus variability for individual non-structural protein regions in the SARS-CoV 1ab region. The nsp11 plot is omitted as all loci evaluated had zero variability.

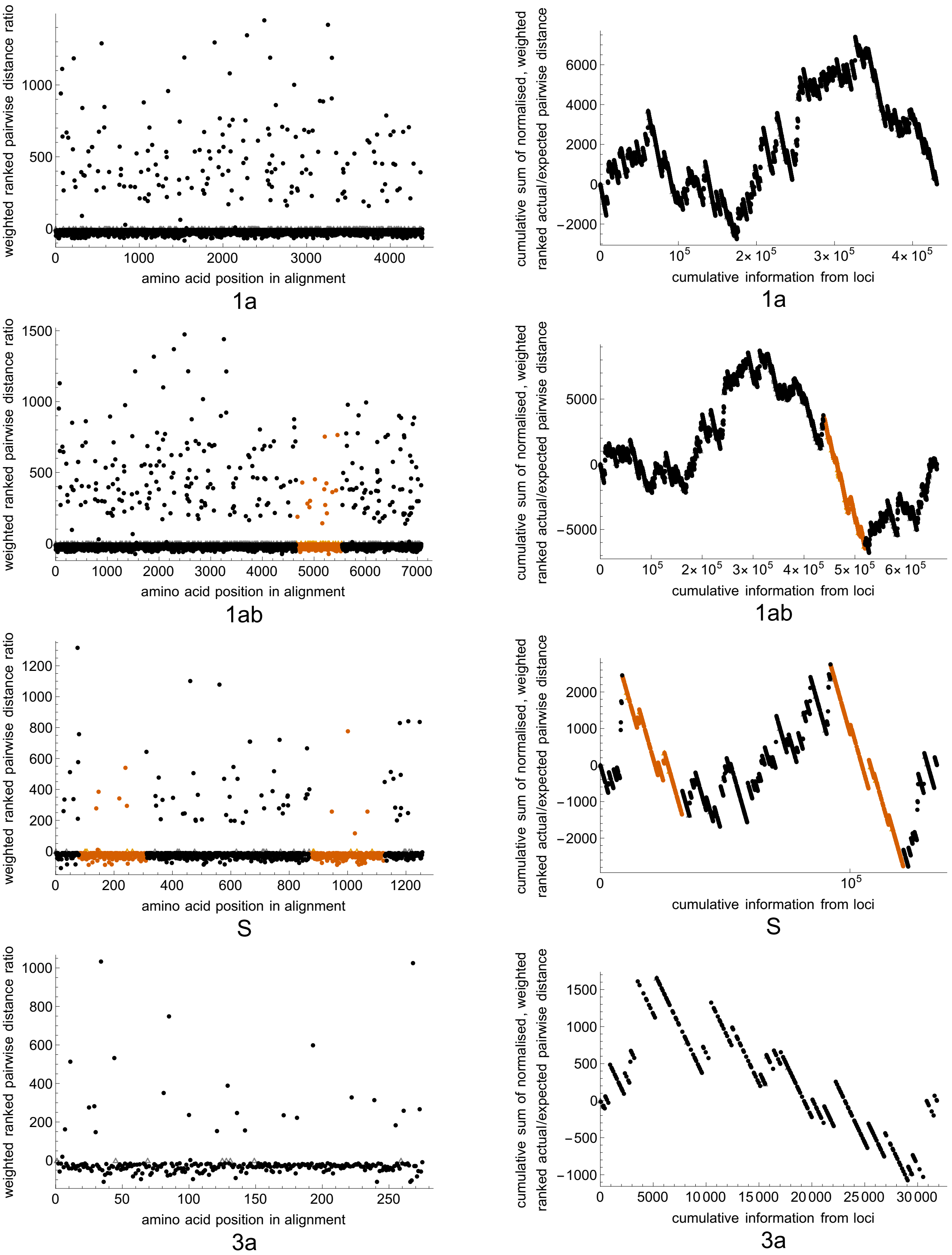

Fig. S63. Output from analyses of SARS-CoV 1a, 1ab, S and 3a genes, with raw analysis output on left and cumulative sums on right. Each dot represents one codon. Regions deemed significant by the algorithm are highlighted in orange and regions deemed significant after removal of a potentially interfering region are highlighted in blue. Codons contributing no information to the analysis are in a lighter colour and plotted with open triangles. Plots use weighted, ranked pairwise distance ratios. More detail of significant regions found is given in Table S2.

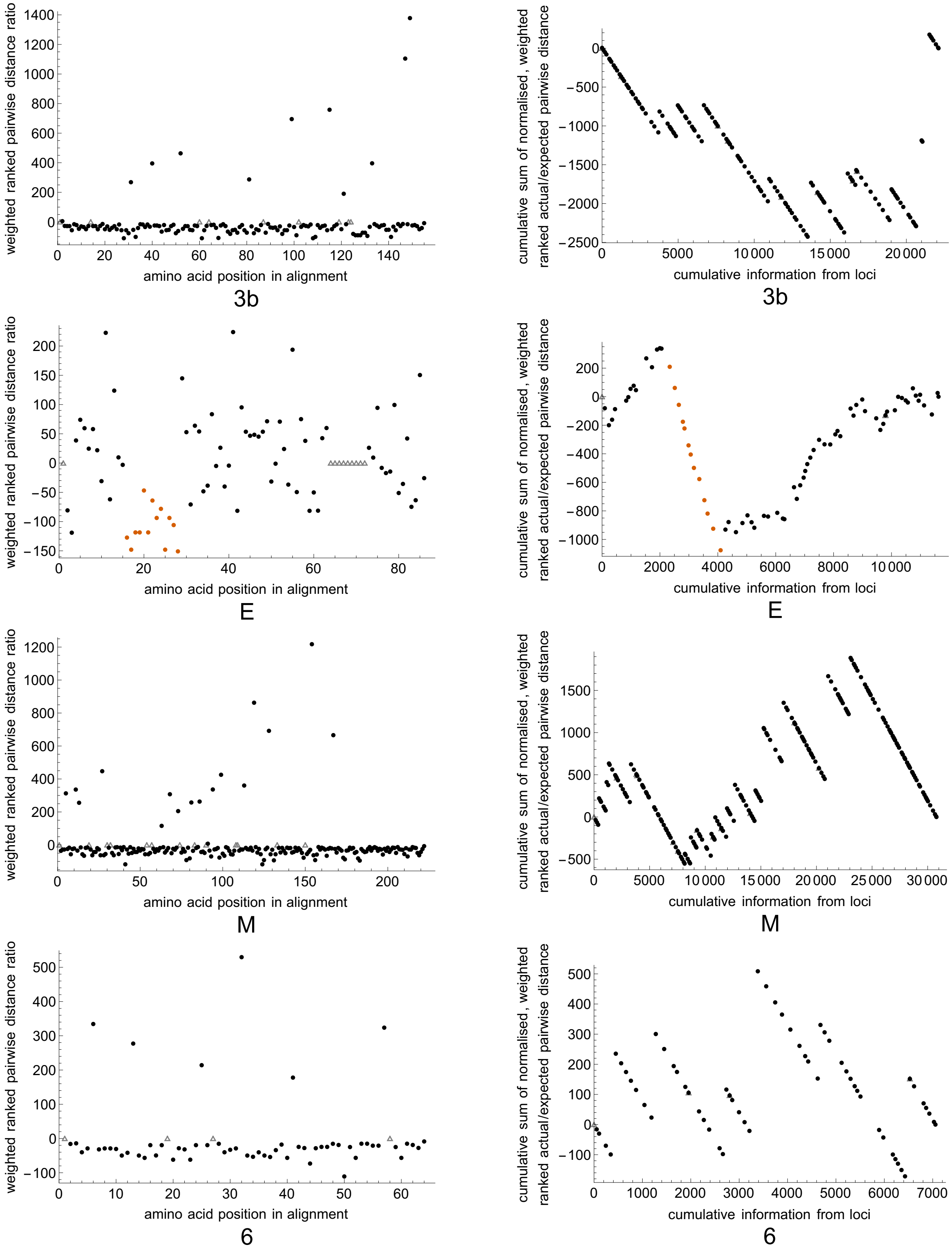

Fig. S64. Output from analyses of SARS-CoV 3b, E, M and 6 genes, with raw analysis output on left and cumulative sums on right. Each dot represents one codon. Regions deemed significant by the algorithm are highlighted in orange and regions deemed significant after removal of a potentially interfering region are highlighted in blue. Codons contributing no information to the analysis are in a lighter colour and plotted with open triangles. Plots use weighted, ranked pairwise distance ratios. More detail of significant regions found is given in Table S2.

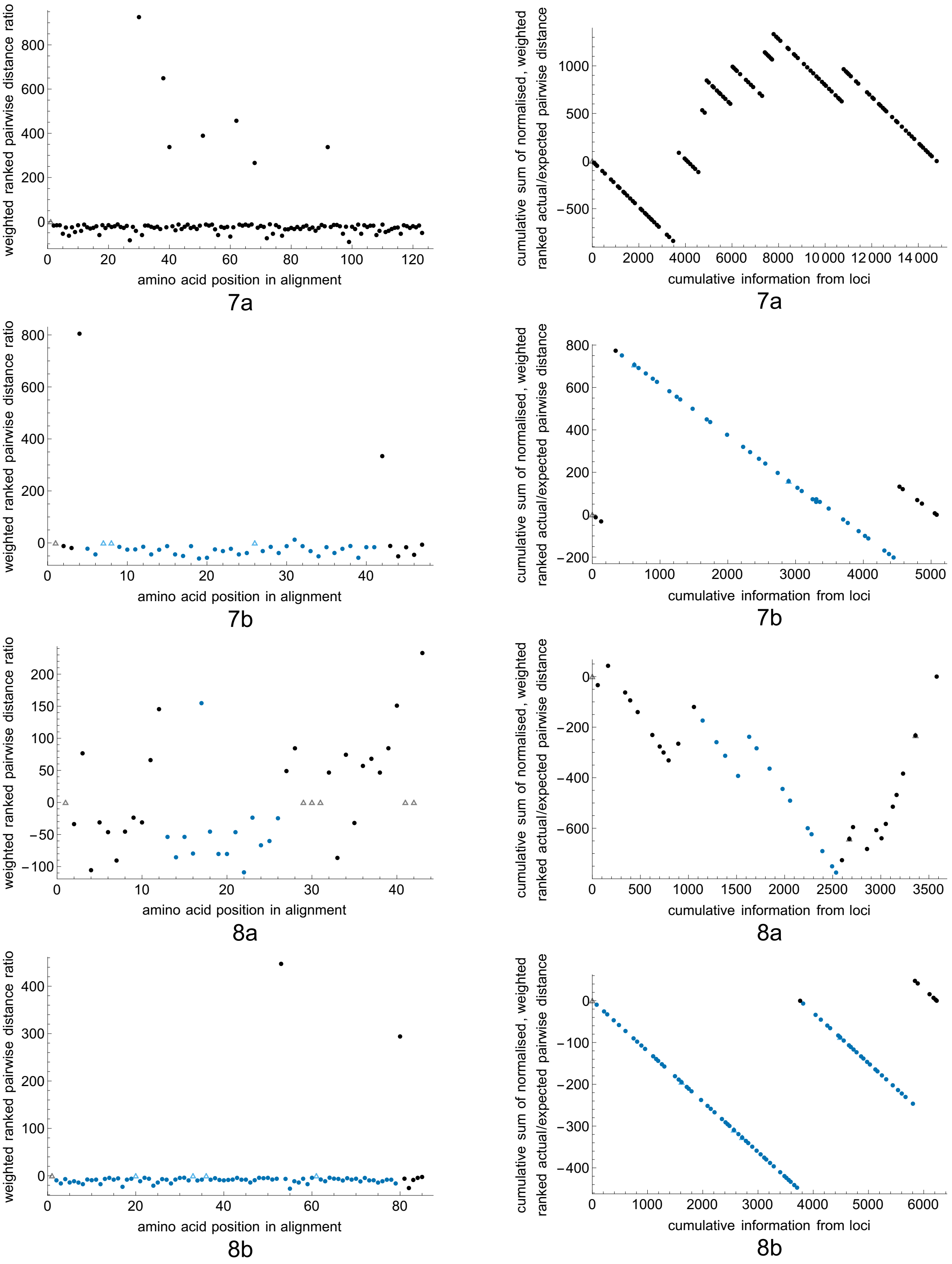

Fig. S65. Output from analyses of SARS-CoV 7a, 7b, 8a and 8b genes, with raw analysis output on left and cumulative sums on right. Each dot represents one codon. Regions deemed significant by the algorithm are highlighted in orange and regions deemed significant after removal of a potentially interfering region are highlighted in blue. Codons contributing no information to the analysis are in a lighter colour and plotted with open triangles. Plots use weighted, ranked pairwise distance ratios. More detail of significant regions found is given in Table S2.

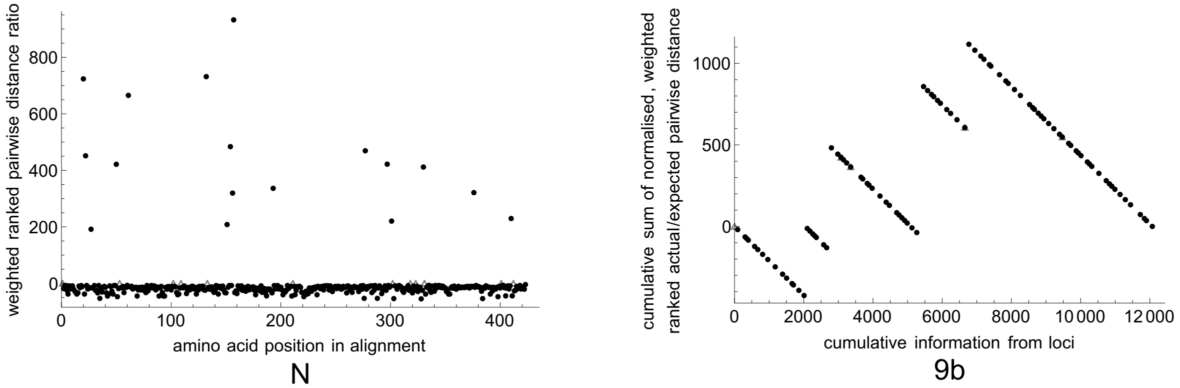

Fig. S66. Output from analyses of SARS-CoV N and 9b genes, with raw analysis output on left and cumulative sums on right. Each dot represents one codon. Regions deemed significant by the algorithm are highlighted in orange and regions deemed significant after removal of a potentially interfering region are highlighted in blue. Codons contributing no information to the analysis are in a lighter colour and plotted with open triangles. Plots use weighted, ranked pairwise distance ratios. More detail of significant regions found is given in Table S2.

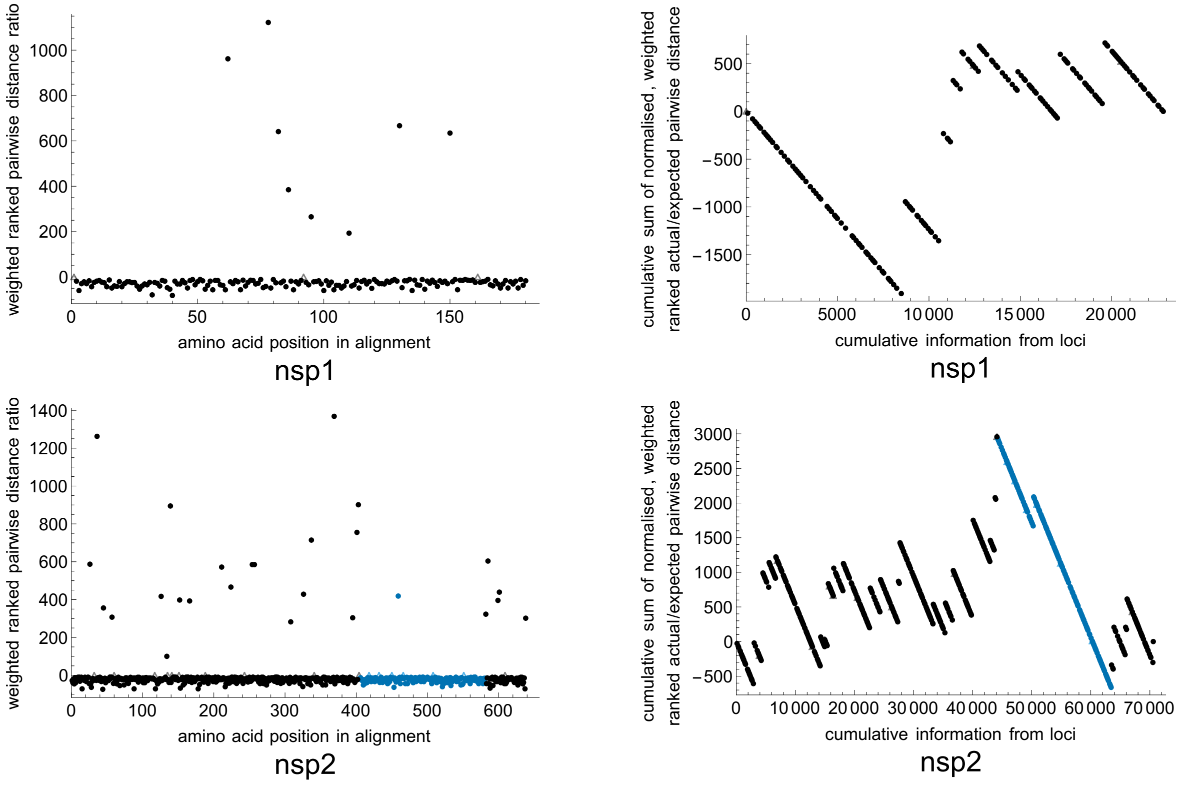

Fig. S67. Output from analyses of SARS-CoV nsp1 and nsp2 regions, with raw analysis output on left and cumulative sums on right. Each dot represents one codon. Regions deemed significant by the algorithm are highlighted in orange and regions deemed significant after removal of a potentially interfering region are highlighted in blue. Codons contributing no information to the analysis are in a lighter colour and plotted with open triangles. Plots use weighted, ranked pairwise distance ratios. More detail of significant regions found is given in Table S3.

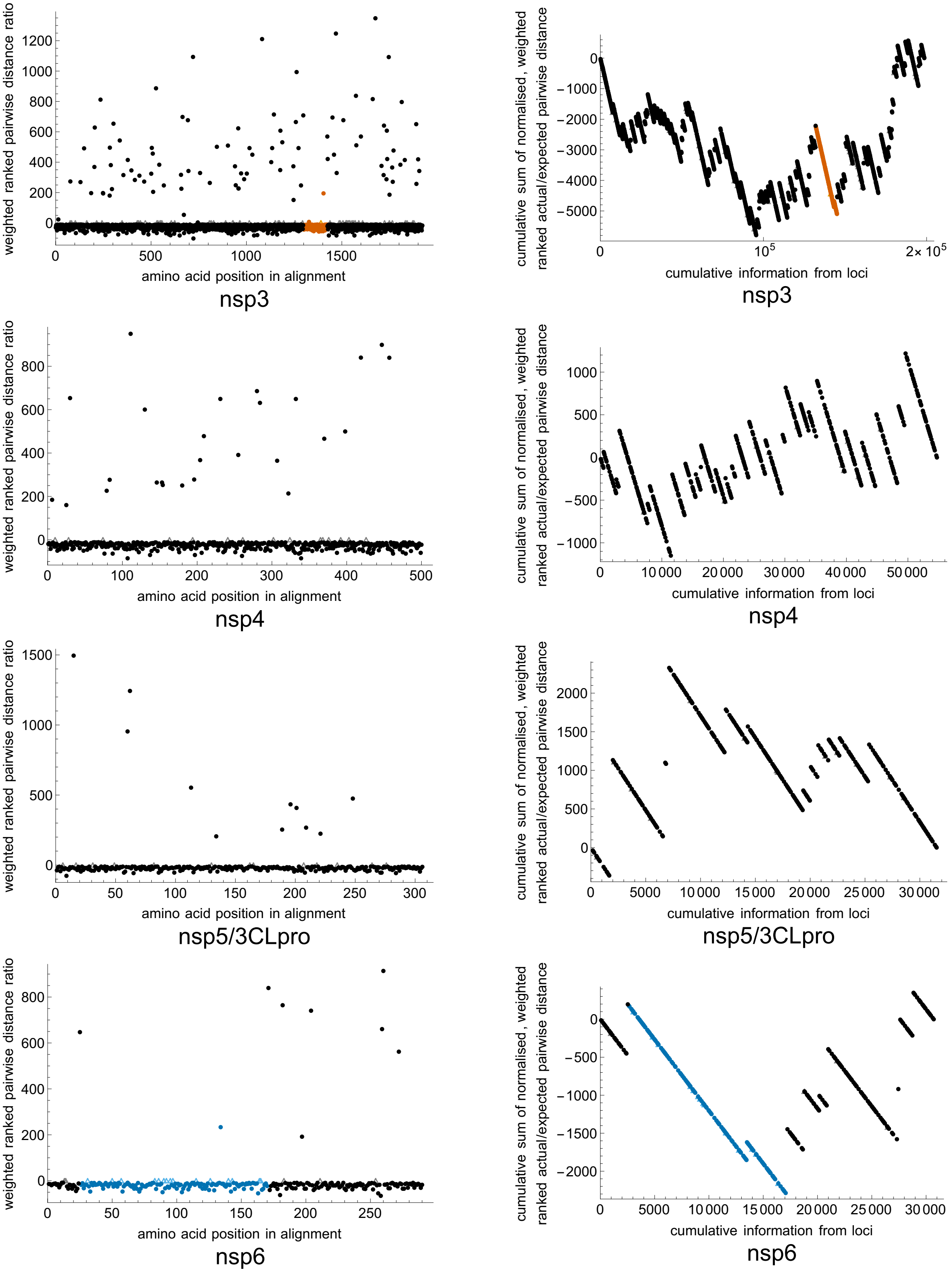

Fig. S68. Output from analyses of SARS-CoV nsp3, nsp4, nsp5 and nsp6 regions, with raw analysis output on left and cumulative sums on right. Each dot represents one codon. Regions deemed significant by the algorithm are highlighted in orange and regions deemed significant after removal of a potentially interfering region are highlighted in blue. Codons contributing no information to the analysis are in a lighter colour and plotted with open triangles. Plots use weighted, ranked pairwise distance ratios. More detail of significant regions found is given in Table S3.

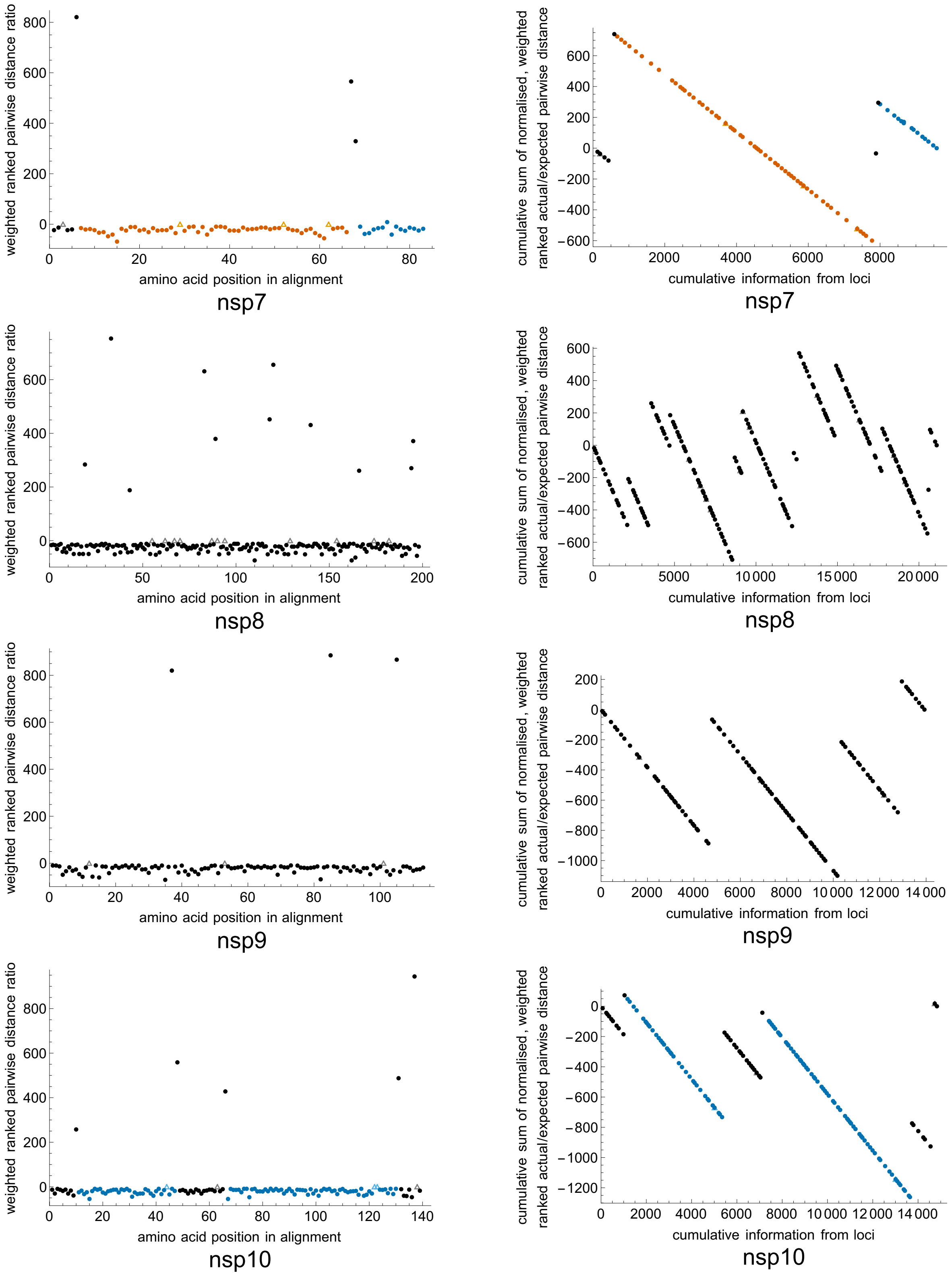

Fig. S69. Output from analyses of SARS-CoV nsp7, nsp8, nsp9 and nsp10 regions, with raw analysis output on left and cumulative sums on right. Each dot represents one codon. Regions deemed significant by the algorithm are highlighted in orange and regions deemed significant after removal of a potentially interfering region are highlighted in blue. Codons contributing no information to the analysis are in a lighter colour and plotted with open triangles. Plots use weighted, ranked pairwise distance ratios. More detail of significant regions found is given in Table S3.

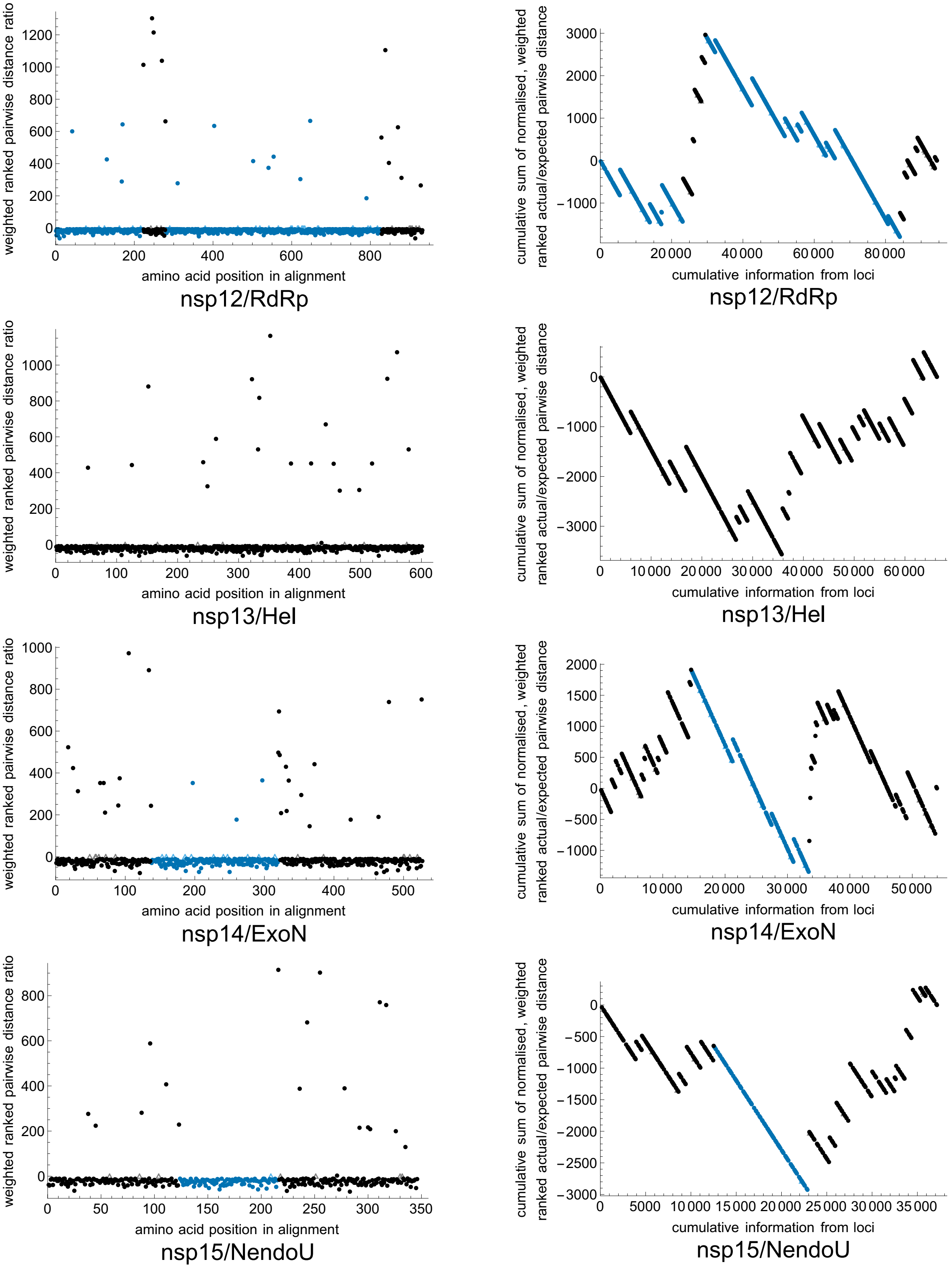

Fig. S70. Output from analyses of SARS-CoV nsp12, nsp13, nsp14 and nsp15 regions, with raw analysis output on left and cumulative sums on right. Each dot represents one codon. Regions deemed significant by the algorithm are highlighted in orange and regions deemed significant after removal of a potentially interfering region are highlighted in blue. Codons contributing no information to the analysis are in a lighter colour and plotted with open triangles. Plots use weighted, ranked pairwise distance ratios. More detail of significant regions found is given in Table S3. Note that output from analysis of nsp11 is not included in the Supplementary Information because no variability is seen in any codon locus in the region.

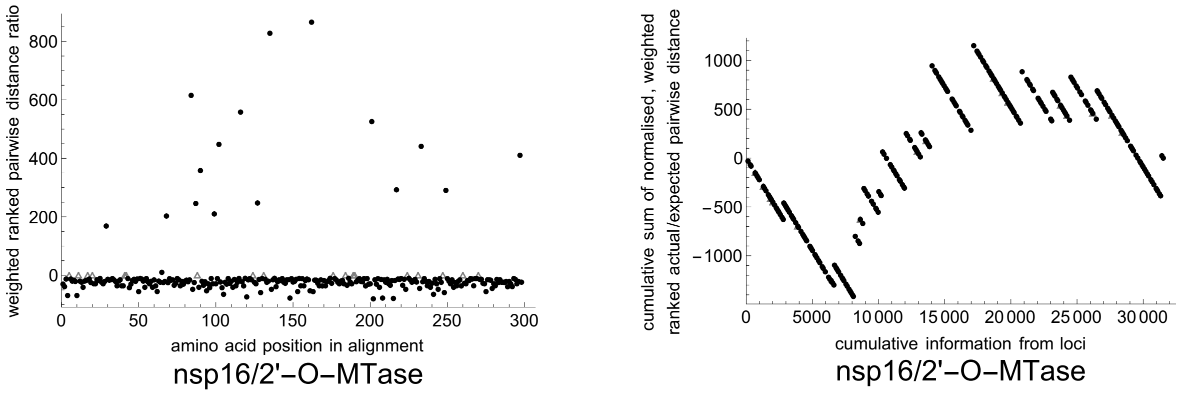

Fig. S71. Output from analyses of SARS-CoV nsp16 region, with raw analysis output on left and cumulative sums on right. Each dot represents one codon. Regions deemed significant by the algorithm are highlighted in orange and regions deemed significant after removal of a potentially interfering region are highlighted in blue. Codons contributing no information to the analysis are in a lighter colour and plotted with open triangles. Plots use weighted, ranked pairwise distance ratios. More detail of significant regions found is given in Table S3.

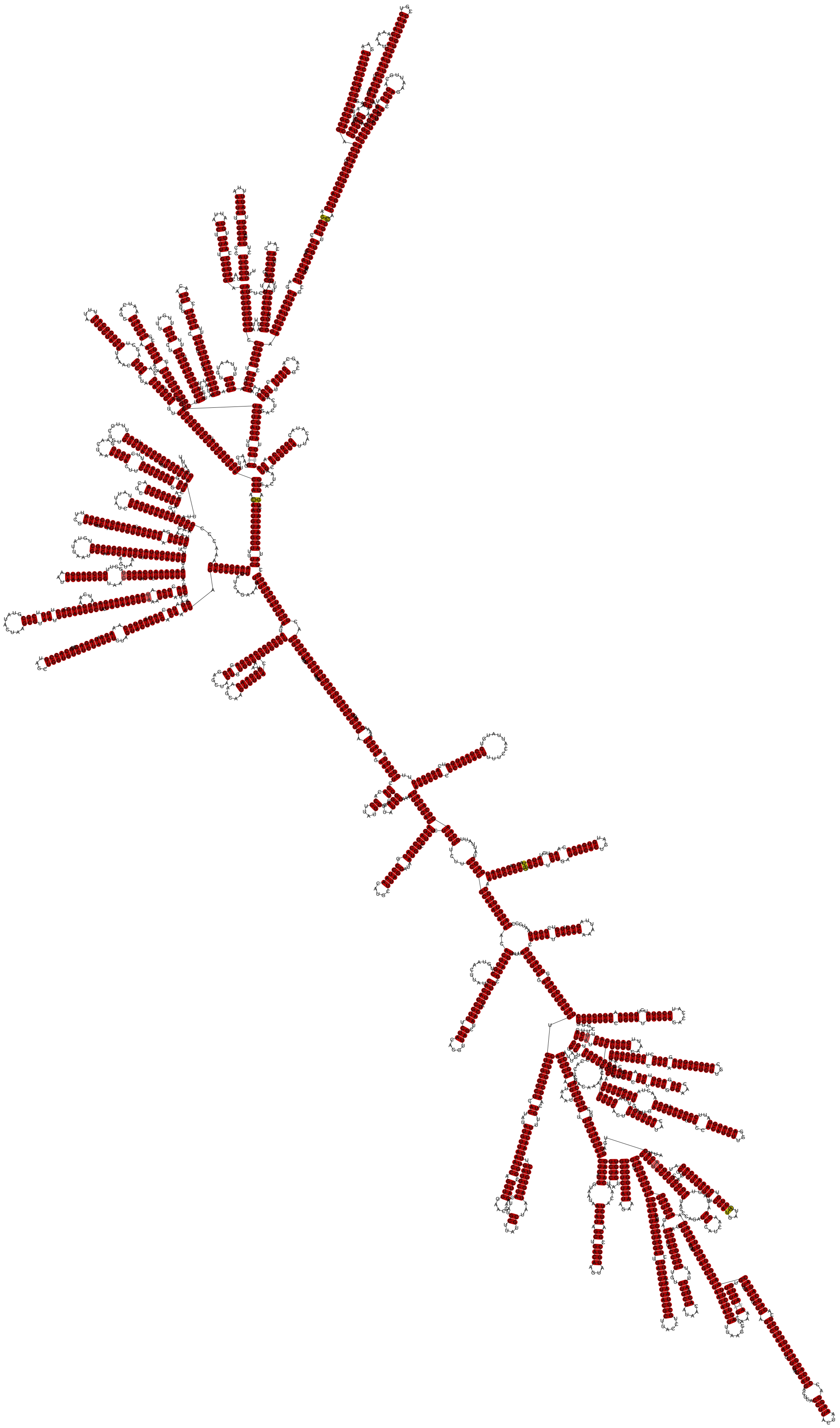

Fig. S72. Output of RNAalifold analysis of the conserved region found 1st in analysis of the 1ab region of SARS-CoV, AY274119.3 nucleotide location 14211–16889. All sequences used in conservation analysis of the region are included in the RNAalifold analysis (differently from the SARS-CoV-2 RNAalifold analysis, because of the much smaller number of SARS-CoV sequences). Base pairs are highlighted in deep/mid/light red as all/all but one/all but two sequences are capable of forming the pairs shown. Base pairs are highlighted in deep/mid/light yellow as all/all but one/all but two sequences are capable of forming the pair shown or one other base pair (including GU pairs). Base pairs are highlighted in deep/mid/light green as all/all but one/all but two sequences are capable of forming the pair shown or either of another two base pairs (including GU pairs). RNAalifold was used with input options disallowing lonely pairs, allowing G-quadruplexes, and with the ribosum scoring matrix enabled.

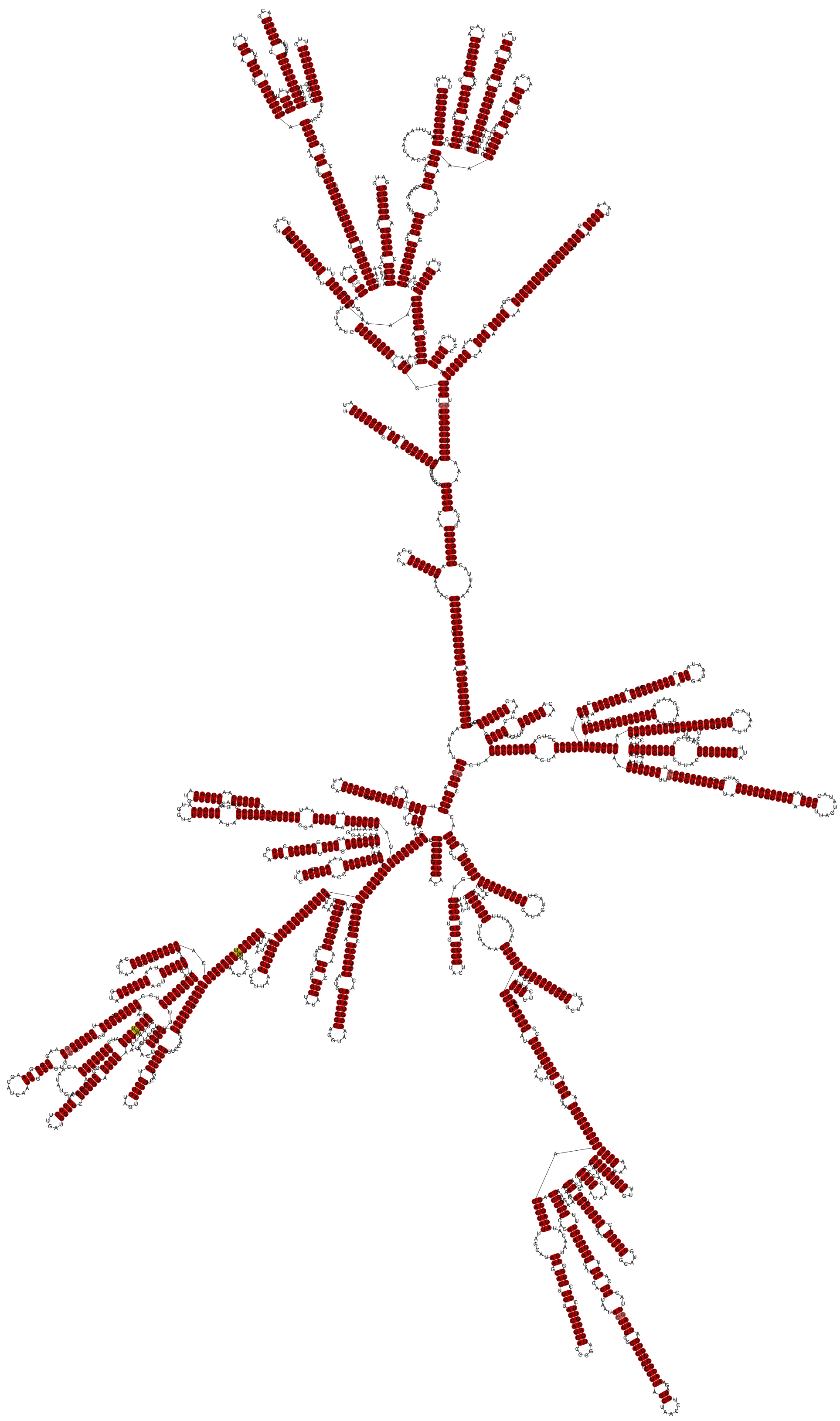

Fig. S73. Output of RNAalifold analysis of the reverse complement of the conserved region found 1st in analysis of the 1ab region of SARS-CoV, AY274119.3 nucleotide location 14211–16889. All sequences used in conservation analysis of the region are included in the RNAalifold analysis (differently from the SARS-CoV-2 RNAalifold analysis, because of the much smaller number of SARS-CoV sequences). Base pairs are highlighted in deep/mid/light red as all/all but one/all but two sequences are capable of forming the pairs shown. Base pairs are highlighted in deep/mid/light yellow as all/all but one/all but two sequences are capable of forming the pair shown or one other base pair (including GU pairs). Base pairs are highlighted in deep/mid/light green as all/all but one/all but two sequences are capable of forming the pair shown or either of another two base pairs (including GU pairs). RNAalifold was used with input options disallowing lonely pairs, allowing G-quadruplexes, and with the ribosum scoring matrix enabled.

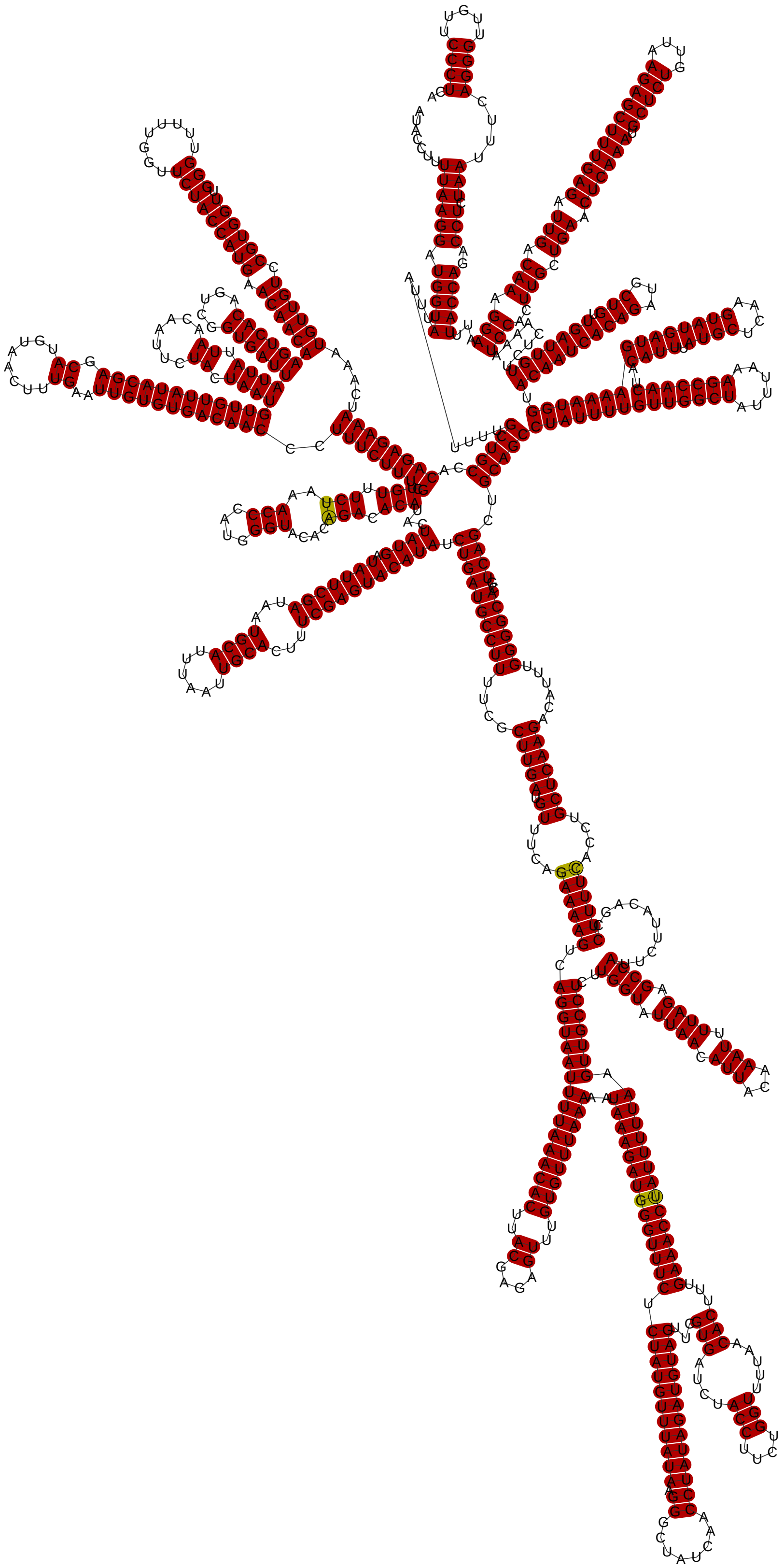

Fig. S74. Output of RNAalifold analysis of the conserved region found 2nd in analysis of the S region of SARS-CoV, AY274119.3 nucleotide location 21732–22421. All sequences used in conservation analysis of the region are included in the RNAalifold analysis (differently from the SARS-CoV-2 RNAalifold analysis, because of the much smaller number of SARS-CoV sequences). Base pairs are highlighted in deep/mid/light red as all/all but one/all but two sequences are capable of forming the pairs shown. Base pairs are highlighted in deep/mid/light yellow as all/all but one/all but two sequences are capable of forming the pair shown or one other base pair (including GU pairs). Base pairs are highlighted in deep/mid/light green as all/all but one/all but two sequences are capable of forming the pair shown or either of another two base pairs (including GU pairs). RNAalifold was used with input options disallowing lonely pairs, allowing G-quadruplexes, and with the ribosum scoring matrix enabled.

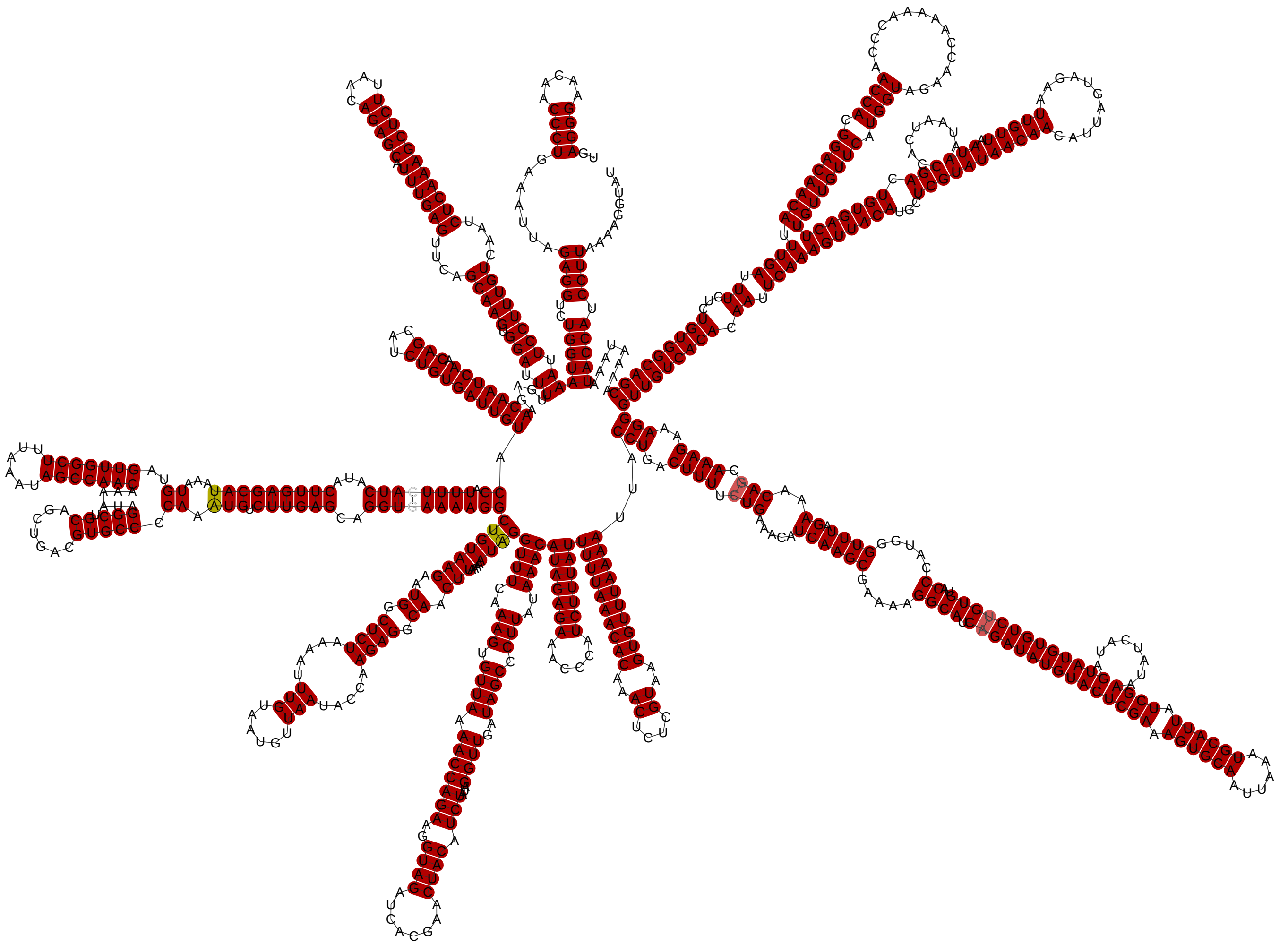

Fig. S75. Output of RNAalifold analysis of the reverse complement of the conserved region found 2nd in analysis of the S region of SARS-CoV, AY274119.3 nucleotide location 21732–22421. All sequences used in conservation analysis of the region are included in the RNAalifold analysis (differently from the SARS-CoV-2 RNAalifold analysis, because of the much smaller number of SARS-CoV sequences). Base pairs are highlighted in deep/mid/light red as all/all but one/all but two sequences are capable of forming the pairs shown. Base pairs are highlighted in deep/mid/light yellow as all/all but one/all but two sequences are capable of forming the pair shown or one other base pair (including GU pairs). Base pairs are highlighted in deep/mid/light green as all/all but one/all but two sequences are capable of forming the pair shown or either of another two base pairs (including GU pairs). RNAalifold was used with input options disallowing lonely pairs, allowing G-quadruplexes, and with the ribosum scoring matrix enabled.

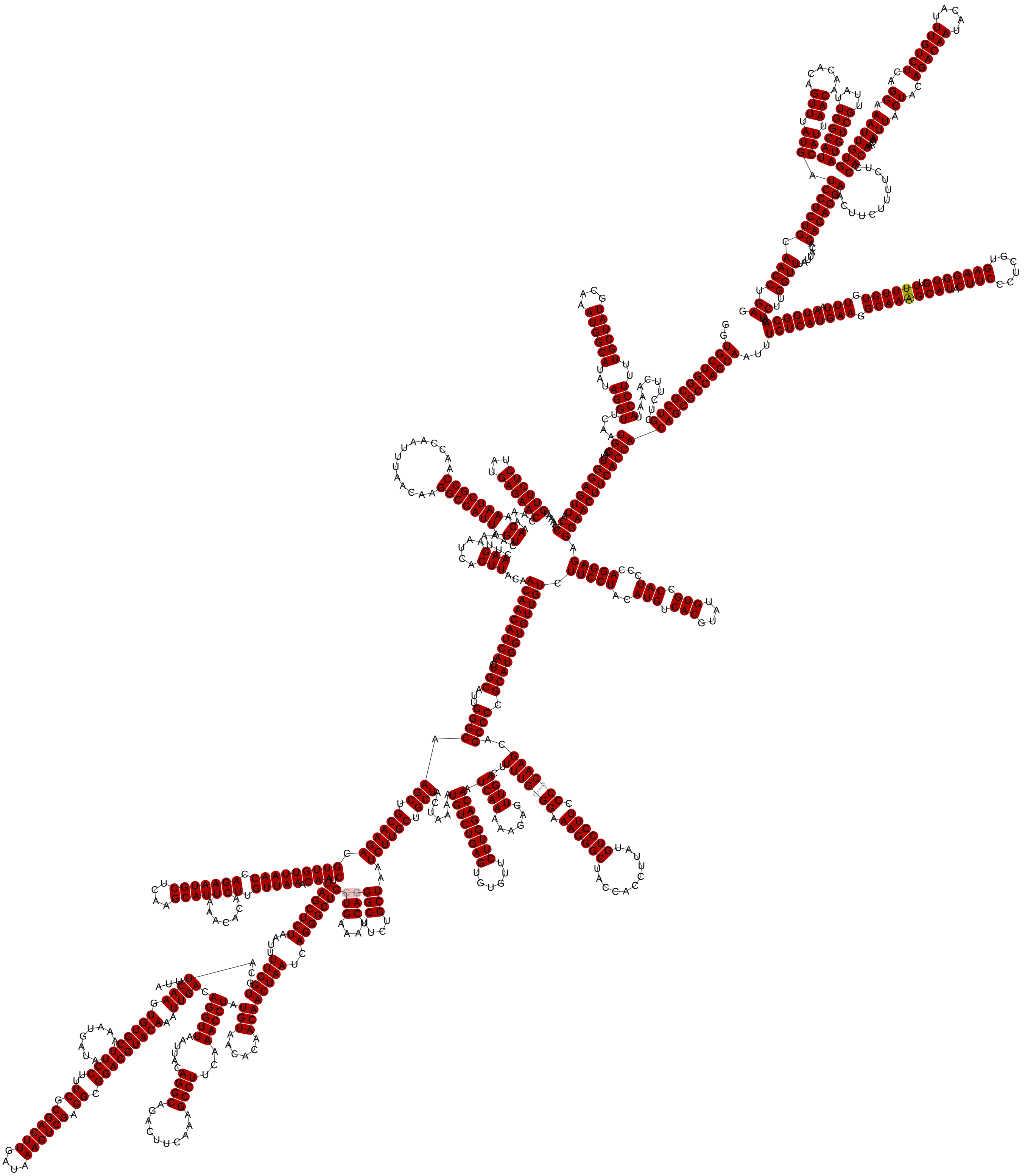

Fig. S76. Output of RNAalifold analysis of the conserved region found 1st in analysis of the S region of SARS-CoV, AY274119.3 nucleotide location 24099–24869. All sequences used in conservation analysis of the region are included in the RNAalifold analysis (differently from the SARS-CoV-2 RNAalifold analysis, because of the much smaller number of SARS-CoV sequences). Base pairs are highlighted in deep/mid/light red as all/all but one/all but two sequences are capable of forming the pairs shown. Base pairs are highlighted in deep/mid/light yellow as all/all but one/all but two sequences are capable of forming the pair shown or one other base pair (including GU pairs). Base pairs are highlighted in deep/mid/light green as all/all but one/all but two sequences are capable of forming the pair shown or either of another two base pairs (including GU pairs). RNAalifold was used with input options disallowing lonely pairs, allowing G-quadruplexes, and with the ribosum scoring matrix enabled.

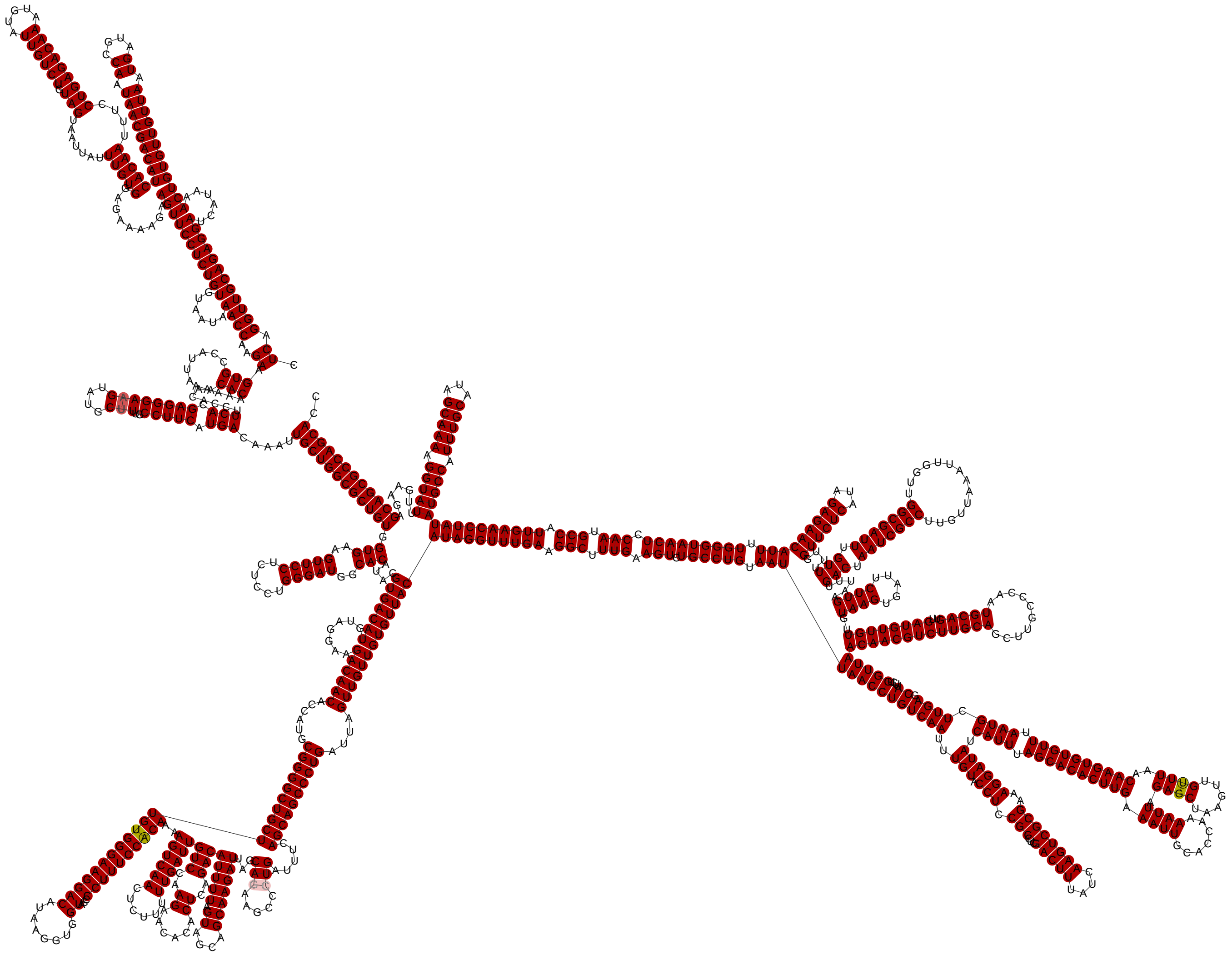

Fig. S77. Output of RNAalifold analysis of the reverse complement of the conserved region found 1st in analysis of the S region of SARS-CoV, AY274119.3 nucleotide location 24099–24869. All sequences used in conservation analysis of the region are included in the RNAalifold analysis (differently from the SARS-CoV-2 RNAalifold analysis, because of the much smaller number of SARS-CoV sequences). Base pairs are highlighted in deep/mid/light red as all/all but one/all but two sequences are capable of forming the pairs shown. Base pairs are highlighted in deep/mid/light yellow as all/all but one/all but two sequences are capable of forming the pair shown or one other base pair (including GU pairs). Base pairs are highlighted in deep/mid/light green as all/all but one/all but two sequences are capable of forming the pair shown or either of another two base pairs (including GU pairs). RNAalifold was used with input options disallowing lonely pairs, allowing G-quadruplexes, and with the ribosum scoring matrix enabled.

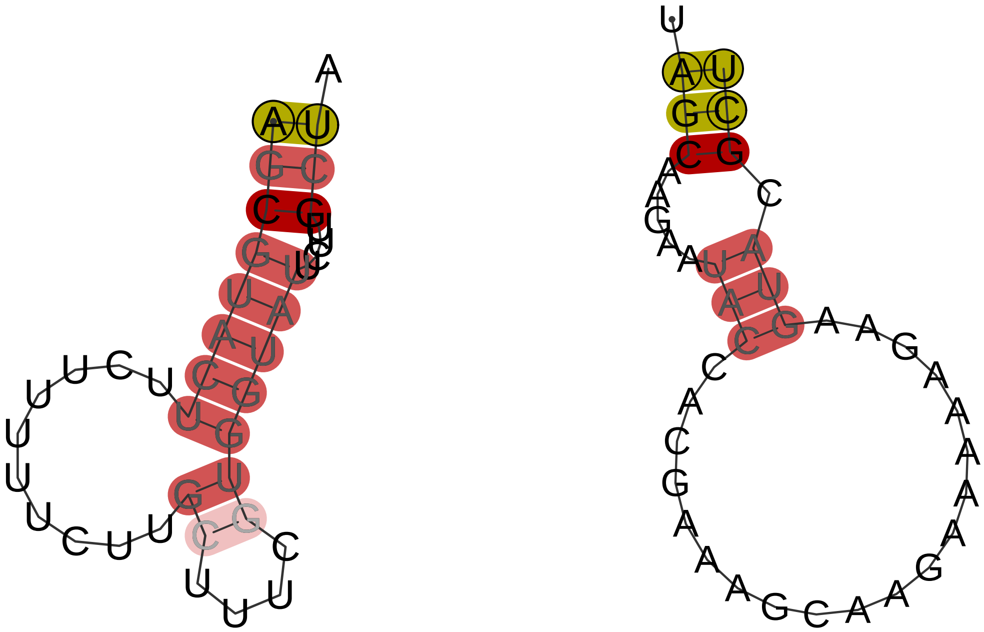

Fig. S78. Output of RNAalifold analysis of the conserved region found 1st in analysis of the E region of SARS-CoV, AY274119.3 nucleotide location 24099–24869 (left: forward sense; right: reverse complement). All sequences used in conservation analysis of the region are included in the RNAalifold analysis (differently from the SARS-CoV-2 RNAalifold analysis, because of the much smaller number of SARS-CoV sequences). Base pairs are highlighted in deep/mid/light red as all/all but one/all but two sequences are capable of forming the pairs shown. Base pairs are highlighted in deep/mid/light yellow as all/all but one/all but two sequences are capable of forming the pair shown or one other base pair (including GU pairs). Base pairs are highlighted in deep/mid/light green as all/all but one/all but two sequences are capable of forming the pair shown or either of another two base pairs (including GU pairs). RNAalifold was used with input options disallowing lonely pairs, allowing G-quadruplexes, and with the ribosum scoring matrix enabled.

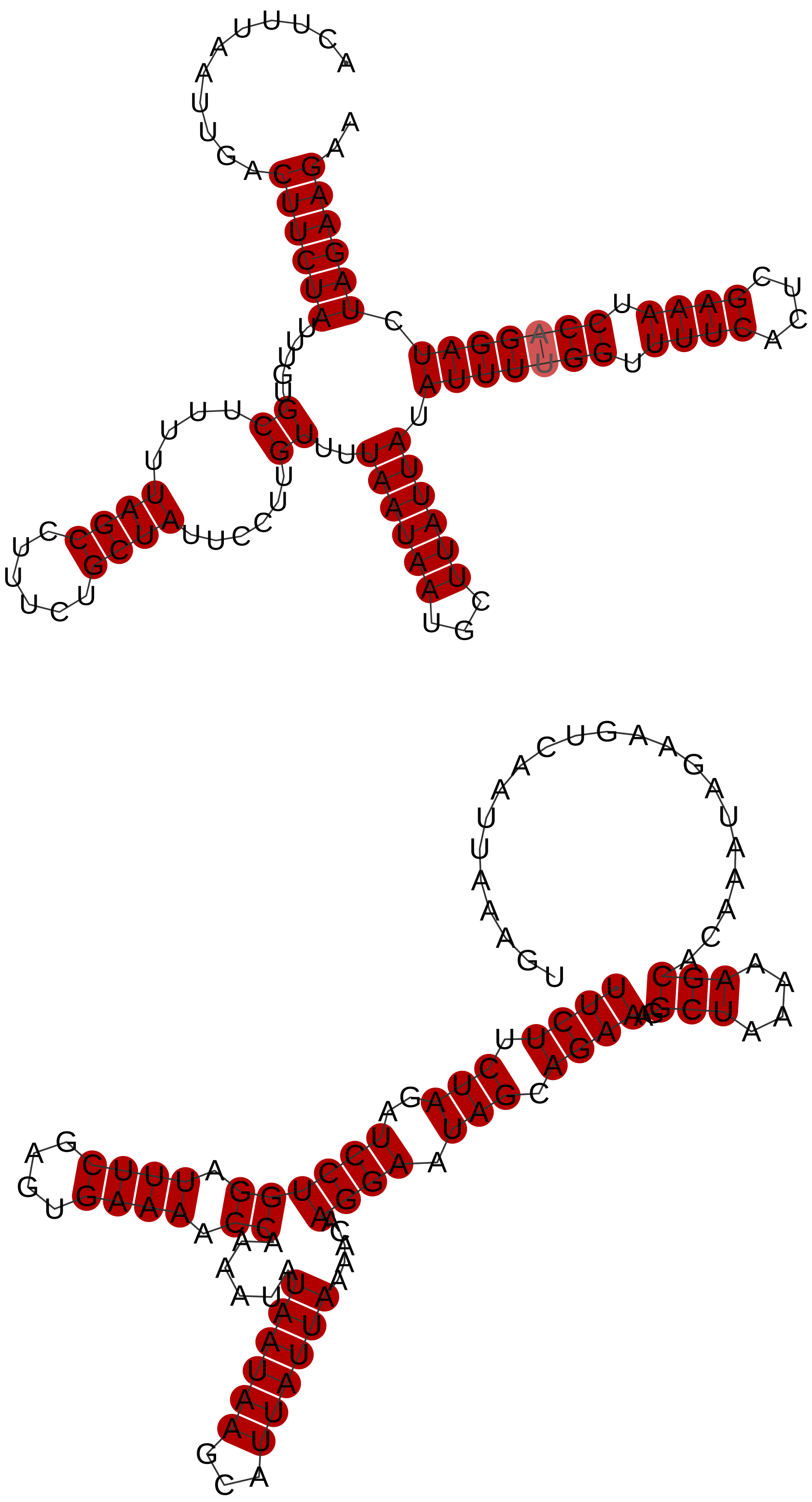

Fig. S79. Output of RNAalifold analysis of the conserved region found 1st in analysis of the 7b region of SARS-CoV, AY274119.3 nucleotide location 27650–27760 (top: forward sense; bottom: reverse complement). All sequences used in conservation analysis of the region are included in the RNAalifold analysis (differently from the SARS-CoV-2 RNAalifold analysis, because of the much smaller number of SARS-CoV sequences). Base pairs are highlighted in deep/mid/light red as all/all but one/all but two sequences are capable of forming the pairs shown. Base pairs are highlighted in deep/mid/light yellow as all/all but one/all but two sequences are capable of forming the pair shown or one other base pair (including GU pairs). Base pairs are highlighted in deep/mid/light green as all/all but one/all but two sequences are capable of forming the pair shown or either of another two base pairs (including GU pairs). RNAalifold was used with input options disallowing lonely pairs, allowing G-quadruplexes, and with the ribosum scoring matrix enabled.

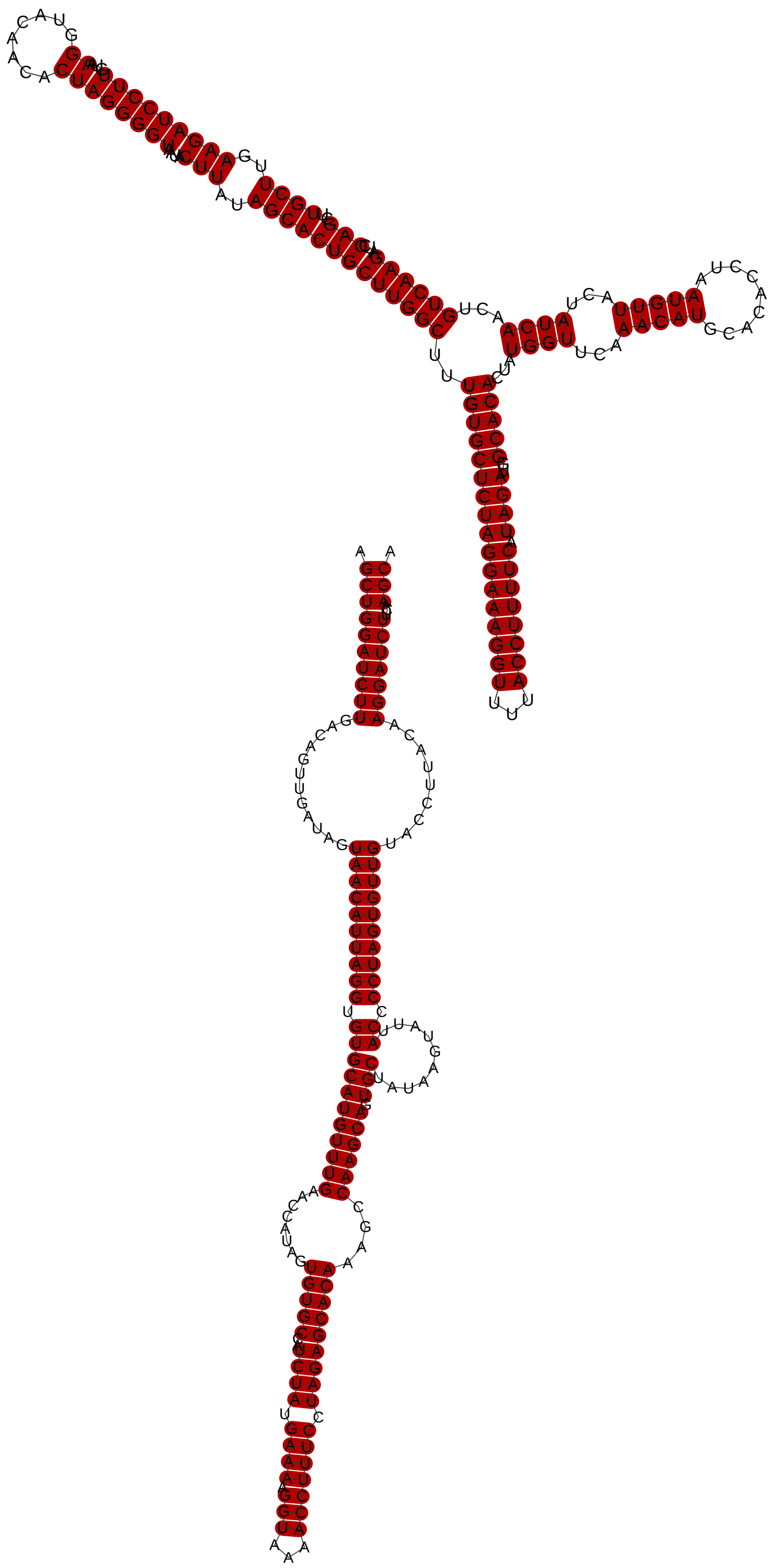

Fig. S80. Output of RNAalifold analysis of the conserved region found 1st in analysis of the 8b region of SARS-CoV, AY274119.3 nucleotide location 27867–28019 (top: forward sense; bottom: reverse complement). All sequences used in conservation analysis of the region are included in the RNAalifold analysis (differently from the SARS-CoV-2 RNAalifold analysis, because of the much smaller number of SARS-CoV sequences). Base pairs are highlighted in deep/mid/light red as all/all but one/all but two sequences are capable of forming the pairs shown. Base pairs are highlighted in deep/mid/light yellow as all/all but one/all but two sequences are capable of forming the pair shown or one other base pair (including GU pairs). Base pairs are highlighted in deep/mid/light green as all/all but one/all but two sequences are capable of forming the pair shown or either of another two base pairs (including GU pairs). RNAalifold was used with input options disallowing lonely pairs, allowing G-quadruplexes, and with the ribosum scoring matrix enabled.

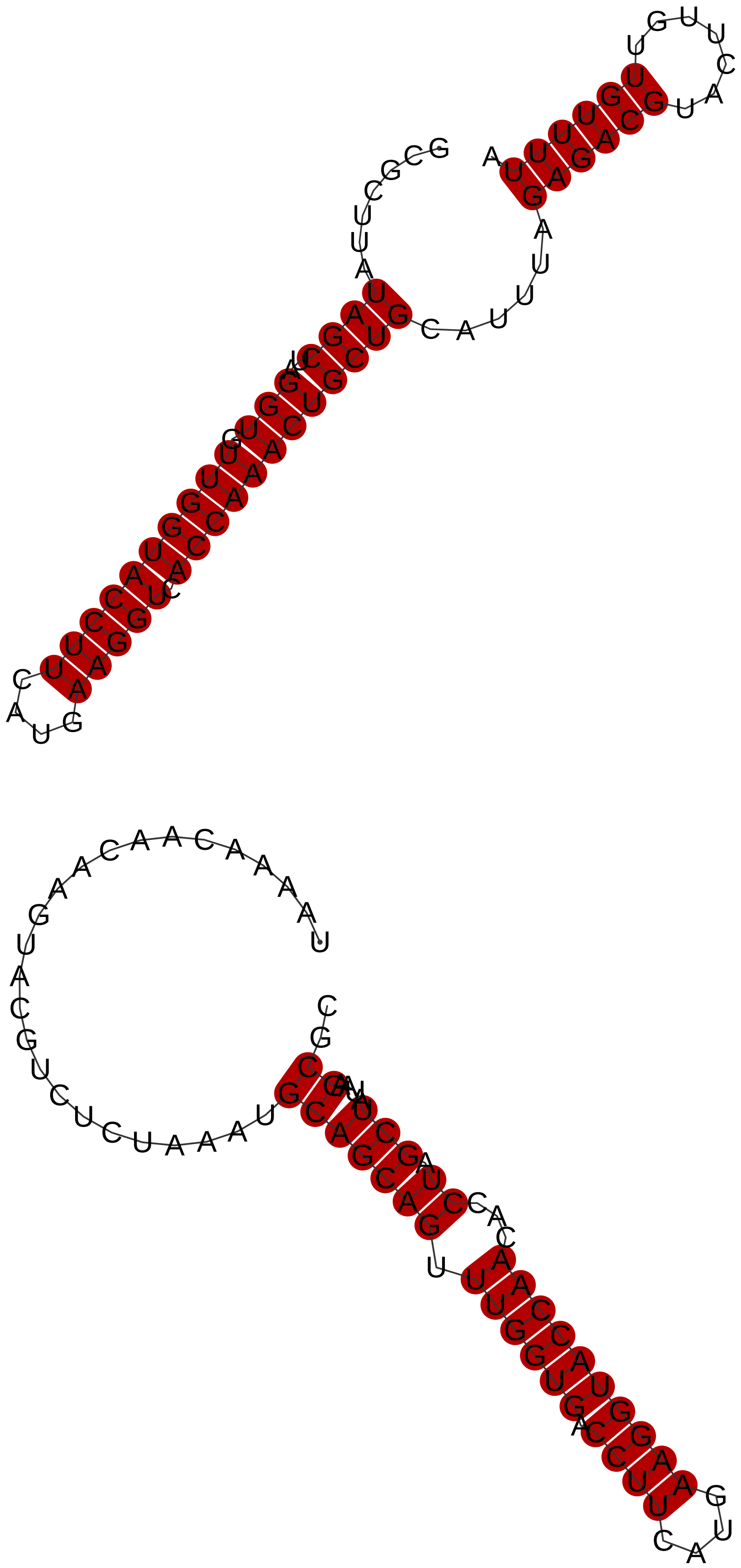

Fig. S81. Output of RNAalifold analysis of the conserved region found 2nd in analysis of the 8b region of SARS-CoV, AY274119.3 nucleotide location 28023–28100 (top: forward sense; bottom: reverse complement). All sequences used in conservation analysis of the region are included in the RNAalifold analysis (differently from the SARS-CoV-2 RNAalifold analysis, because of the much smaller number of SARS-CoV sequences). Base pairs are highlighted in deep/mid/light red as all/all but one/all but two sequences are capable of forming the pairs shown. Base pairs are highlighted in deep/mid/light yellow as all/all but one/all but two sequences are capable of forming the pair shown or one other base pair (including GU pairs). Base pairs are highlighted in deep/mid/light green as all/all but one/all but two sequences are capable of forming the pair shown or either of another two base pairs (including GU pairs). RNAalifold was used with input options disallowing lonely pairs, allowing G-quadruplexes, and with the ribosum scoring matrix enabled.

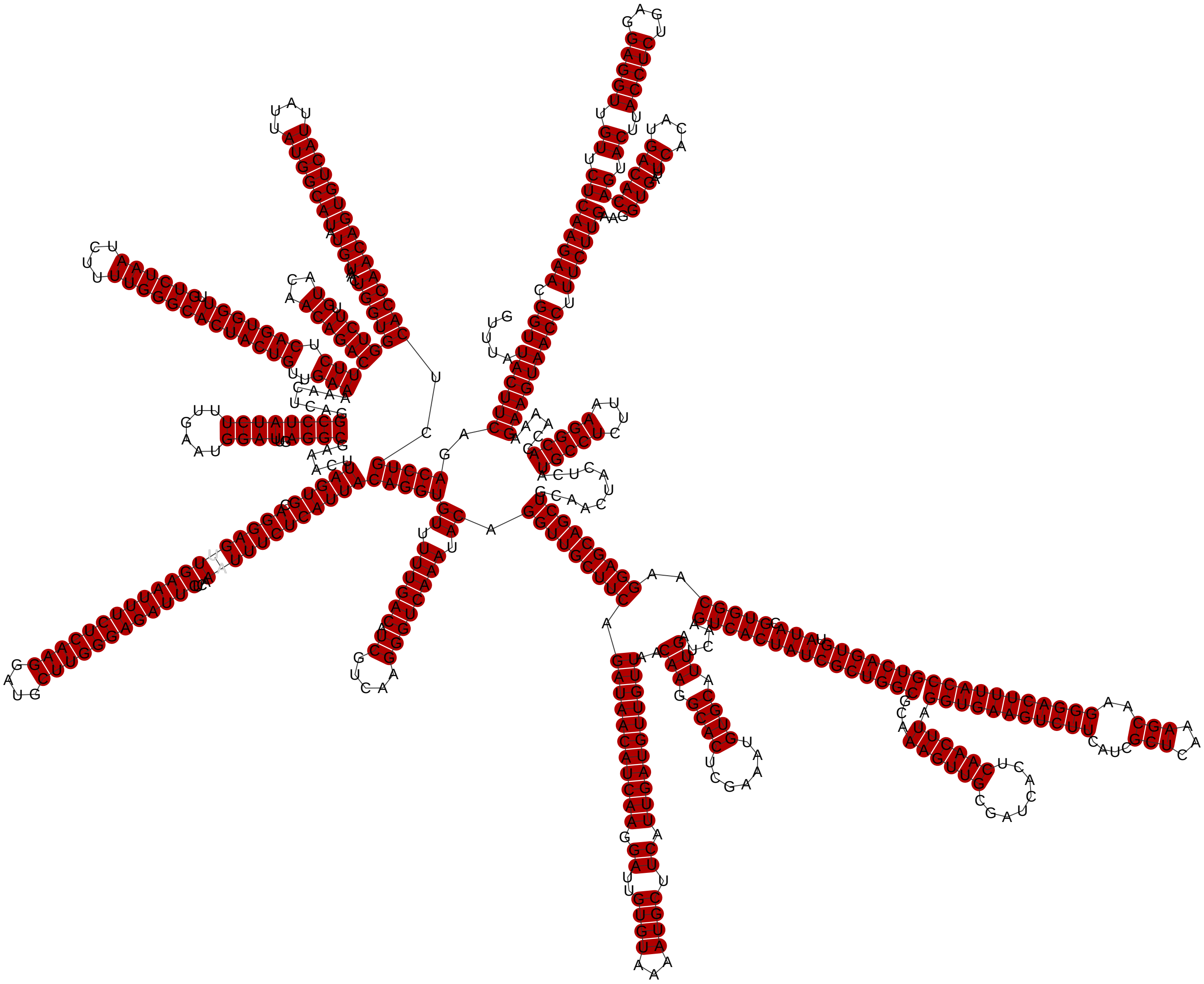

Fig. S82. Output of RNAalifold analysis of the conserved region found 1st in analysis of the nsp2 region of SARS-CoV, AY274119.3 nucleotide location 2017–2547. All sequences used in conservation analysis of the region are included in the RNAalifold analysis (differently from the SARS-CoV-2 RNAalifold analysis, because of the much smaller number of SARS-CoV sequences). Base pairs are highlighted in deep/mid/light red as all/all but one/all but two sequences are capable of forming the pairs shown. Base pairs are highlighted in deep/mid/light yellow as all/all but one/all but two sequences are capable of forming the pair shown or one other base pair (including GU pairs). Base pairs are highlighted in deep/mid/light green as all/all but one/all but two sequences are capable of forming the pair shown or either of another two base pairs (including GU pairs). RNAalifold was used with input options disallowing lonely pairs, allowing G-quadruplexes, and with the ribosum scoring matrix enabled.

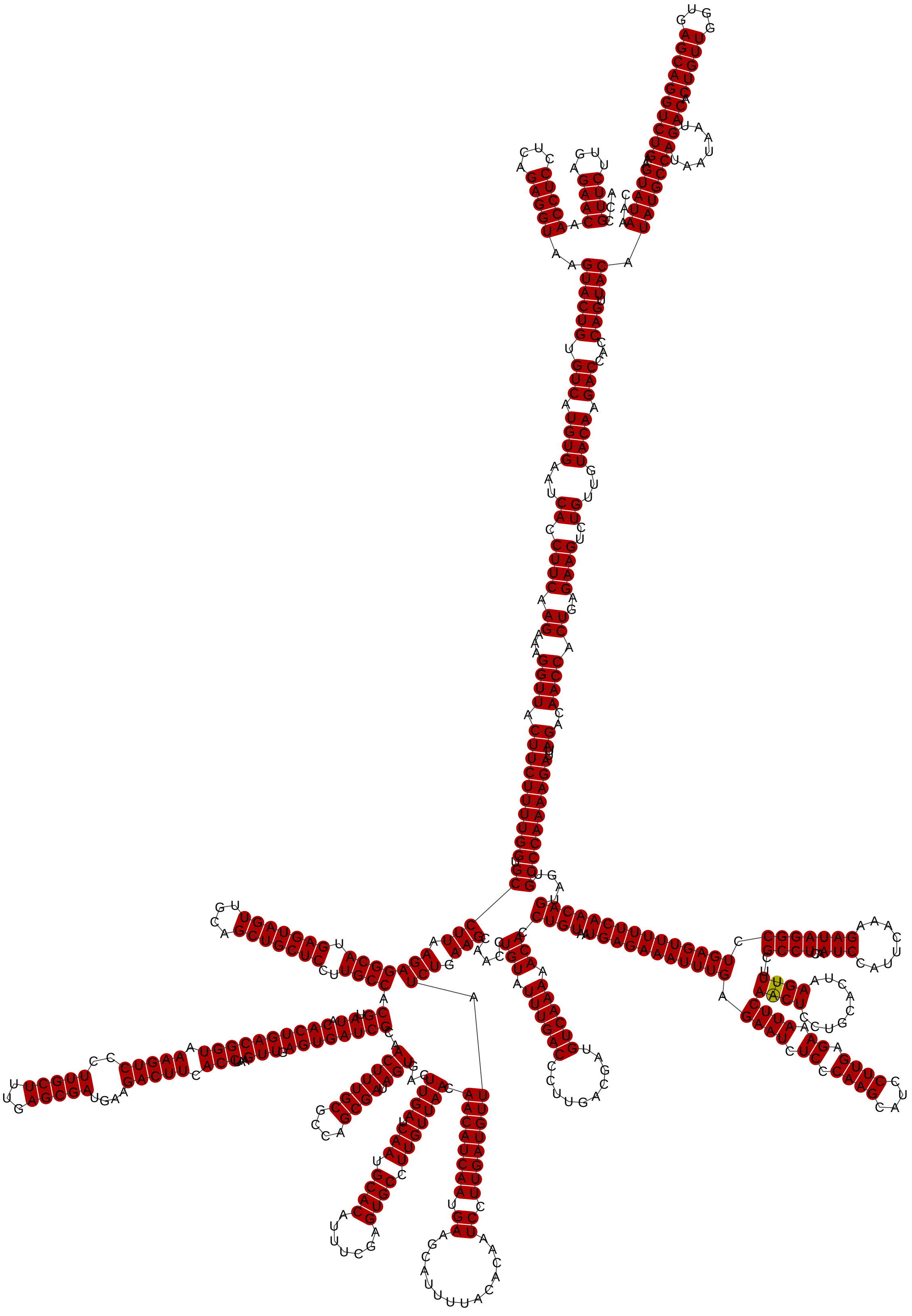

Fig. S83. Output of RNAalifold analysis of the reverse complement conserved region found 1st in analysis of the nsp2 region of SARS-CoV, AY274119.3 nucleotide location 2017–2547. All sequences used in conservation analysis of the region are included in the RNAalifold analysis (differently from the SARS-CoV-2 RNAalifold analysis, because of the much smaller number of SARS-CoV sequences). Base pairs are highlighted in deep/mid/light red as all/all but one/all but two sequences are capable of forming the pairs shown. Base pairs are highlighted in deep/mid/light yellow as all/all but one/all but two sequences are capable of forming the pair shown or one other base pair (including GU pairs). Base pairs are highlighted in deep/mid/light green as all/all but one/all but two sequences are capable of forming the pair shown or either of another two base pairs (including GU pairs). RNAalifold was used with input options disallowing lonely pairs, allowing G-quadruplexes, and with the ribosum scoring matrix enabled.

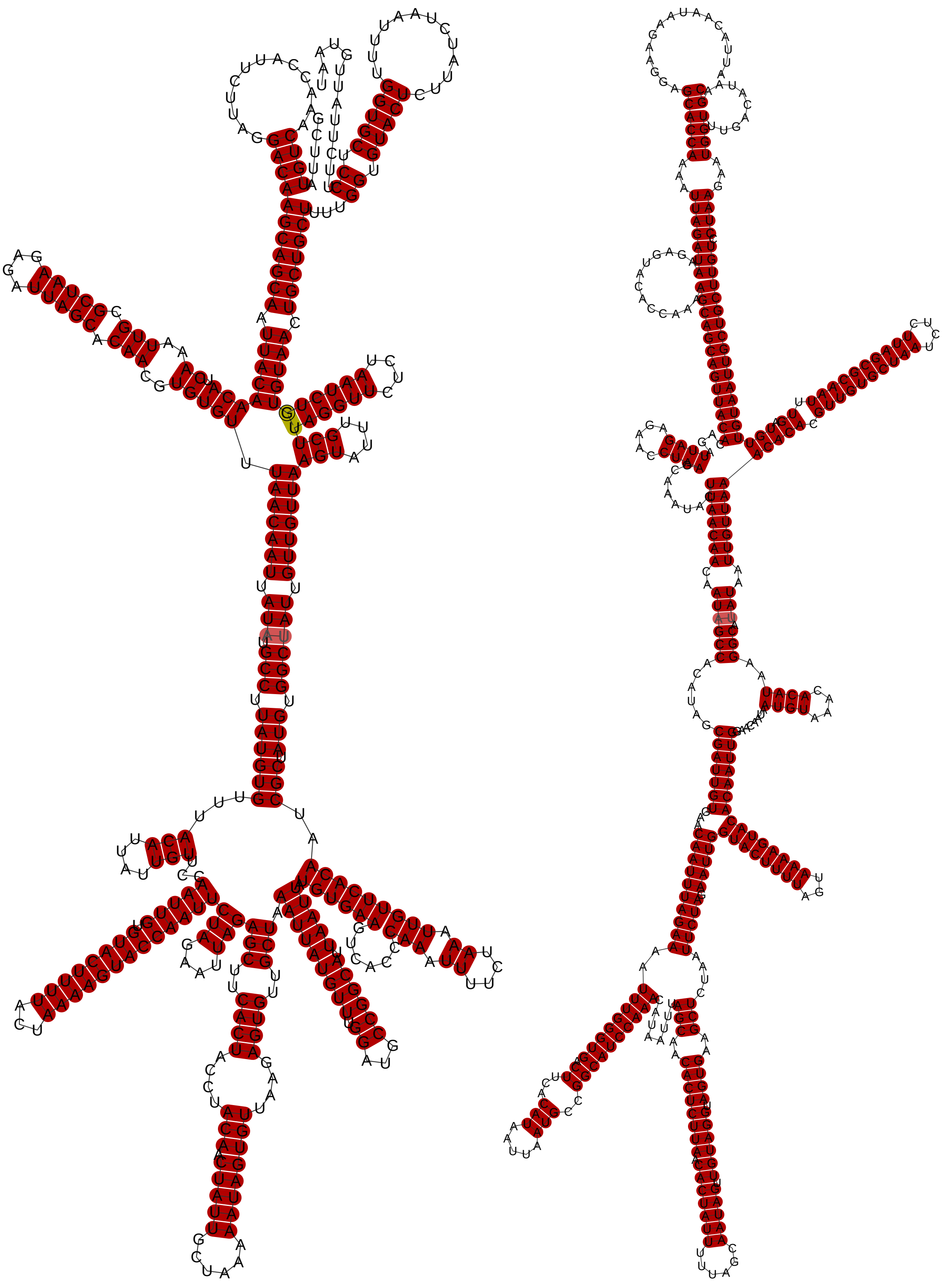

Fig. S84. Output of RNAalifold analysis of the conserved region found 1st in analysis of the nsp3 region of SARS-CoV, AY274119.3 nucleotide location 6613–6987 (left: forward sense; right: reverse complement). All sequences used in conservation analysis of the region are included in the RNAalifold analysis (differently from the SARS-CoV-2 RNAalifold analysis, because of the much smaller number of SARS-CoV sequences). Base pairs are highlighted in deep/mid/light red as all/all but one/all but two sequences are capable of forming the pairs shown. Base pairs are highlighted in deep/mid/light yellow as all/all but one/all but two sequences are capable of forming the pair shown or one other base pair (including GU pairs). Base pairs are highlighted in deep/mid/light green as all/all but one/all but two sequences are capable of forming the pair shown or either of another two base pairs (including GU pairs). RNAalifold was used with input options disallowing lonely pairs, allowing G-quadruplexes, and with the ribosum scoring matrix enabled.

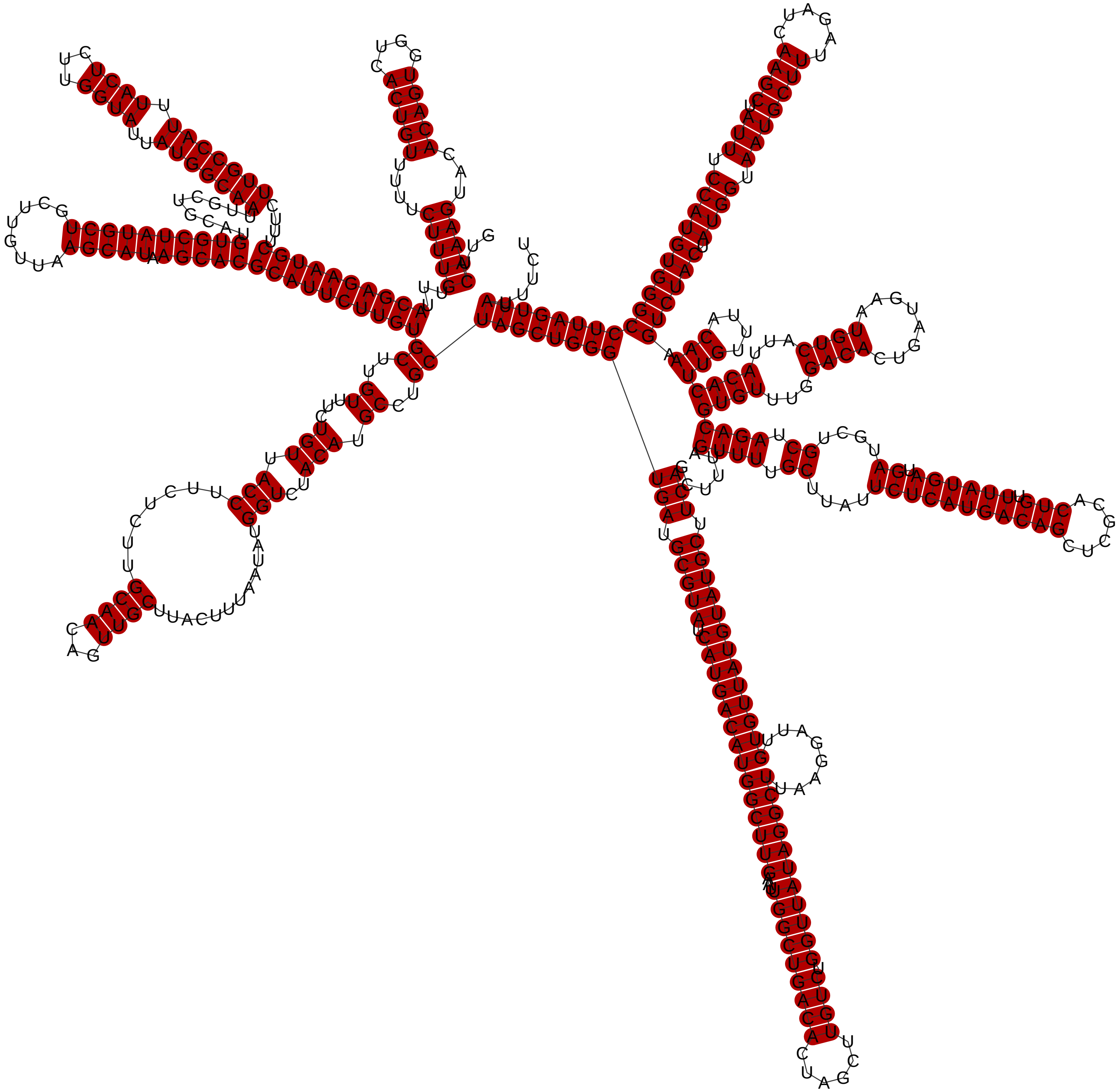

Fig. S85. Output of RNAalifold analysis of the conserved region found 1st in analysis of the nsp6 region of SARS-CoV, AY274119.3 nucleotide location 10978–11412. All sequences used in conservation analysis of the region are included in the RNAalifold analysis (differently from the SARS-CoV-2 RNAalifold analysis, because of the much smaller number of SARS-CoV sequences). Base pairs are highlighted in deep/mid/light red as all/all but one/all but two sequences are capable of forming the pairs shown. Base pairs are highlighted in deep/mid/light yellow as all/all but one/all but two sequences are capable of forming the pair shown or one other base pair (including GU pairs). Base pairs are highlighted in deep/mid/light green as all/all but one/all but two sequences are capable of forming the pair shown or either of another two base pairs (including GU pairs). RNAalifold was used with input options disallowing lonely pairs, allowing G-quadruplexes, and with the ribosum scoring matrix enabled.

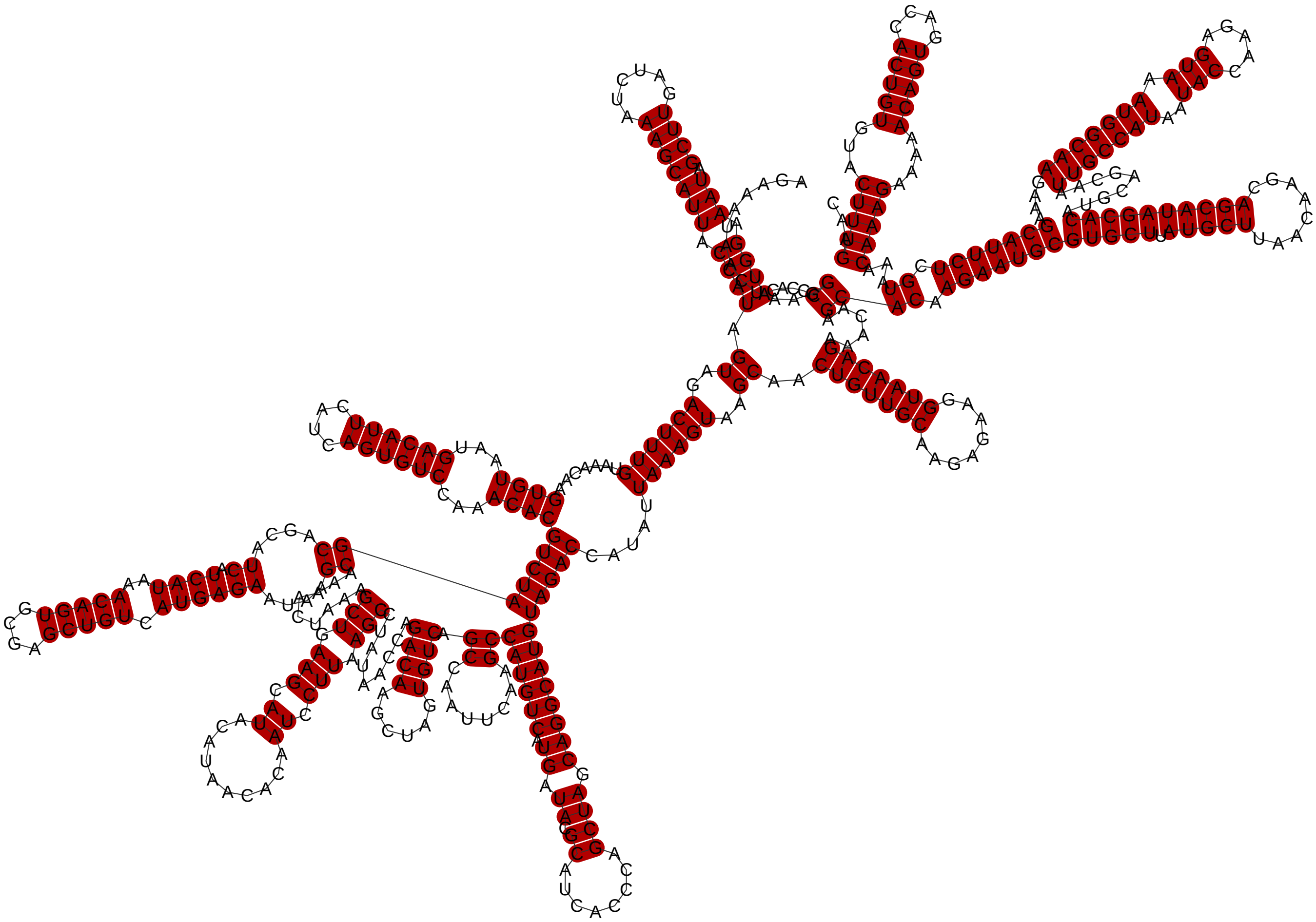

Fig. S86. Output of RNAalifold analysis of the reverse complement of the conserved region found 1st in analysis of the nsp6 region of SARS-CoV, AY274119.3 nucleotide location 10978–11412. All sequences used in conservation analysis of the region are included in the RNAalifold analysis (differently from the SARS-CoV-2 RNAalifold analysis, because of the much smaller number of SARS-CoV sequences). Base pairs are highlighted in deep/mid/light red as all/all but one/all but two sequences are capable of forming the pairs shown. Base pairs are highlighted in deep/mid/light yellow as all/all but one/all but two sequences are capable of forming the pair shown or one other base pair (including GU pairs). Base pairs are highlighted in deep/mid/light green as all/all but one/all but two sequences are capable of forming the pair shown or either of another two base pairs (including GU pairs). RNAalifold was used with input options disallowing lonely pairs, allowing G-quadruplexes, and with the ribosum scoring matrix enabled.

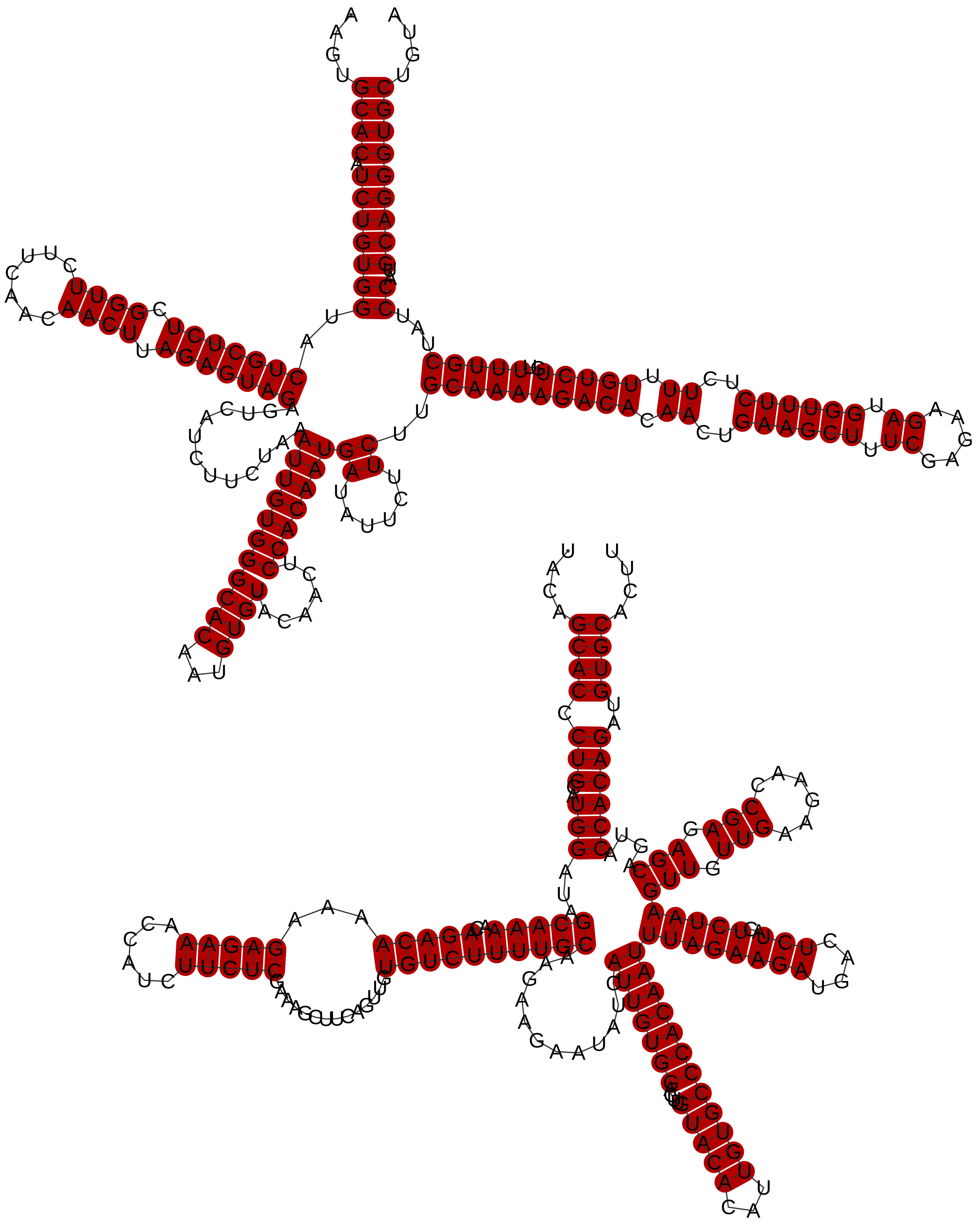

Fig. S87. Output of RNAalifold analysis of the conserved region found 1st in analysis of the nsp7 region of SARS-CoV, AY274119.3 nucleotide location 11791–11970 (top: forward sense; bottom: reverse complement). All sequences used in conservation analysis of the region are included in the RNAalifold analysis (differently from the SARS-CoV-2 RNAalifold analysis, because of the much smaller number of SARS-CoV sequences). Base pairs are highlighted in deep/mid/light red as all/all but one/all but two sequences are capable of forming the pairs shown. Base pairs are highlighted in deep/mid/light yellow as all/all but one/all but two sequences are capable of forming the pair shown or one other base pair (including GU pairs). Base pairs are highlighted in deep/mid/light green as all/all but one/all but two sequences are capable of forming the pair shown or either of another two base pairs (including GU pairs). RNAalifold was used with input options disallowing lonely pairs, allowing G-quadruplexes, and with the ribosum scoring matrix enabled.

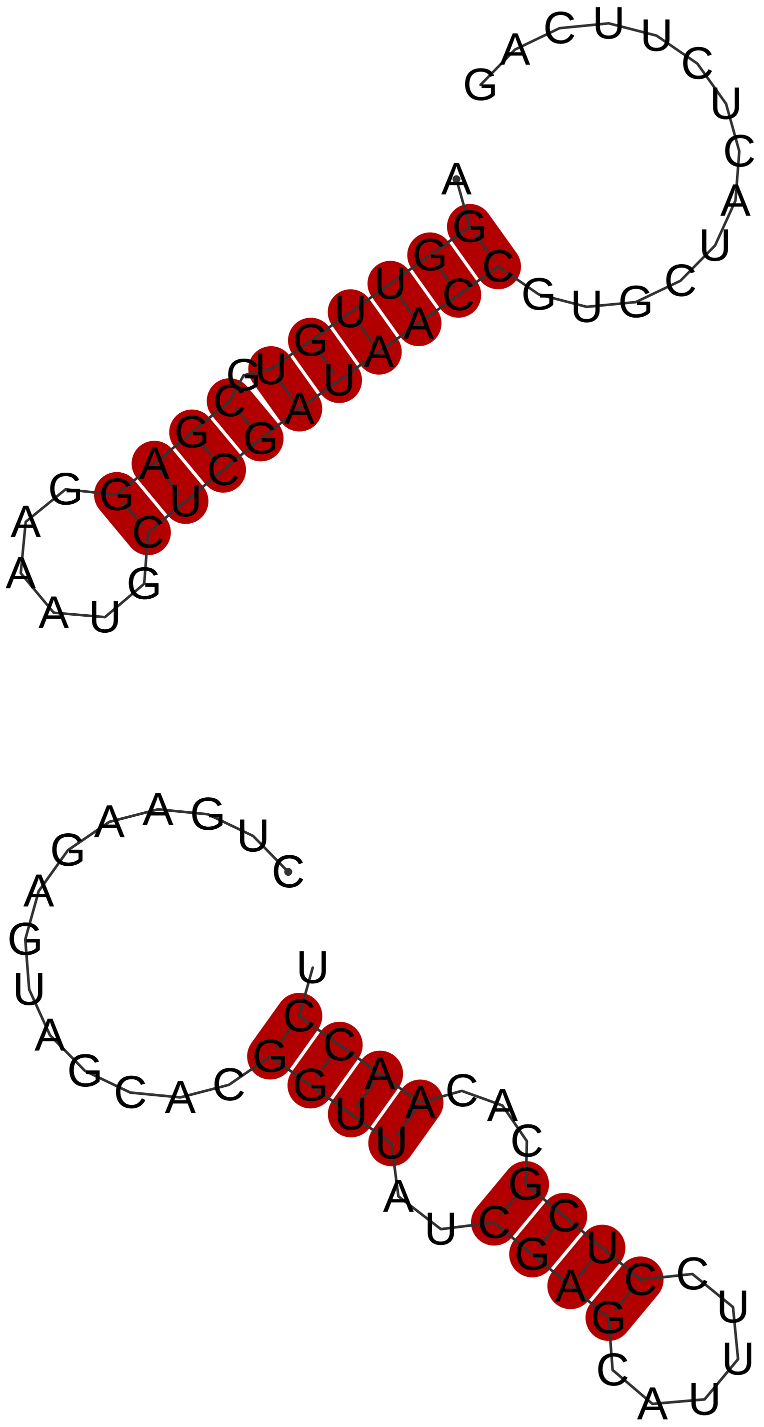

Fig. S88. Output of RNAalifold analysis of the conserved region found 2nd in analysis of the nsp7 region of SARS-CoV, AY274119.3 nucleotide location 11977–12021 (top: forward sense; bottom: reverse complement). All sequences used in conservation analysis of the region are included in the RNAalifold analysis (differently from the SARS-CoV-2 RNAalifold analysis, because of the much smaller number of SARS-CoV sequences). Base pairs are highlighted in deep/mid/light red as all/all but one/all but two sequences are capable of forming the pairs shown. Base pairs are highlighted in deep/mid/light yellow as all/all but one/all but two sequences are capable of forming the pair shown or one other base pair (including GU pairs). Base pairs are highlighted in deep/mid/light green as all/all but one/all but two sequences are capable of forming the pair shown or either of another two base pairs (including GU pairs). RNAalifold was used with input options disallowing lonely pairs, allowing G-quadruplexes, and with the ribosum scoring matrix enabled.

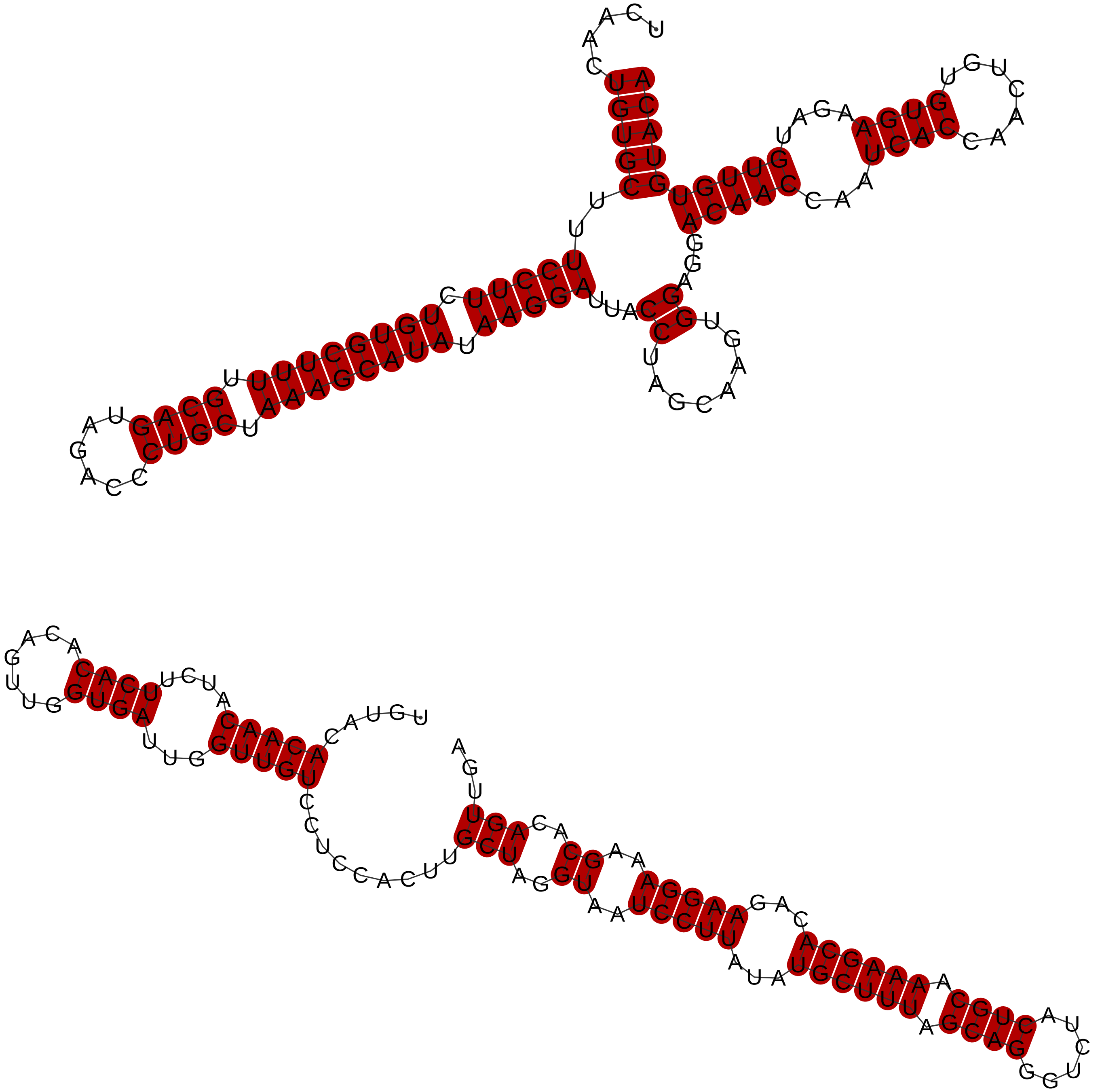

Fig. S89. Output of RNAalifold analysis of the conserved region found 2nd in analysis of the nsp10 region of SARS-CoV, AY274119.3 nucleotide location 12985–13095 (top: forward sense; bottom: reverse complement). All sequences used in conservation analysis of the region are included in the RNAalifold analysis (differently from the SARS-CoV-2 RNAalifold analysis, because of the much smaller number of SARS-CoV sequences). Base pairs are highlighted in deep/mid/light red as all/all but one/all but two sequences are capable of forming the pairs shown. Base pairs are highlighted in deep/mid/light yellow as all/all but one/all but two sequences are capable of forming the pair shown or one other base pair (including GU pairs). Base pairs are highlighted in deep/mid/light green as all/all but one/all but two sequences are capable of forming the pair shown or either of another two base pairs (including GU pairs). RNAalifold was used with input options disallowing lonely pairs, allowing G-quadruplexes, and with the ribosum scoring matrix enabled.

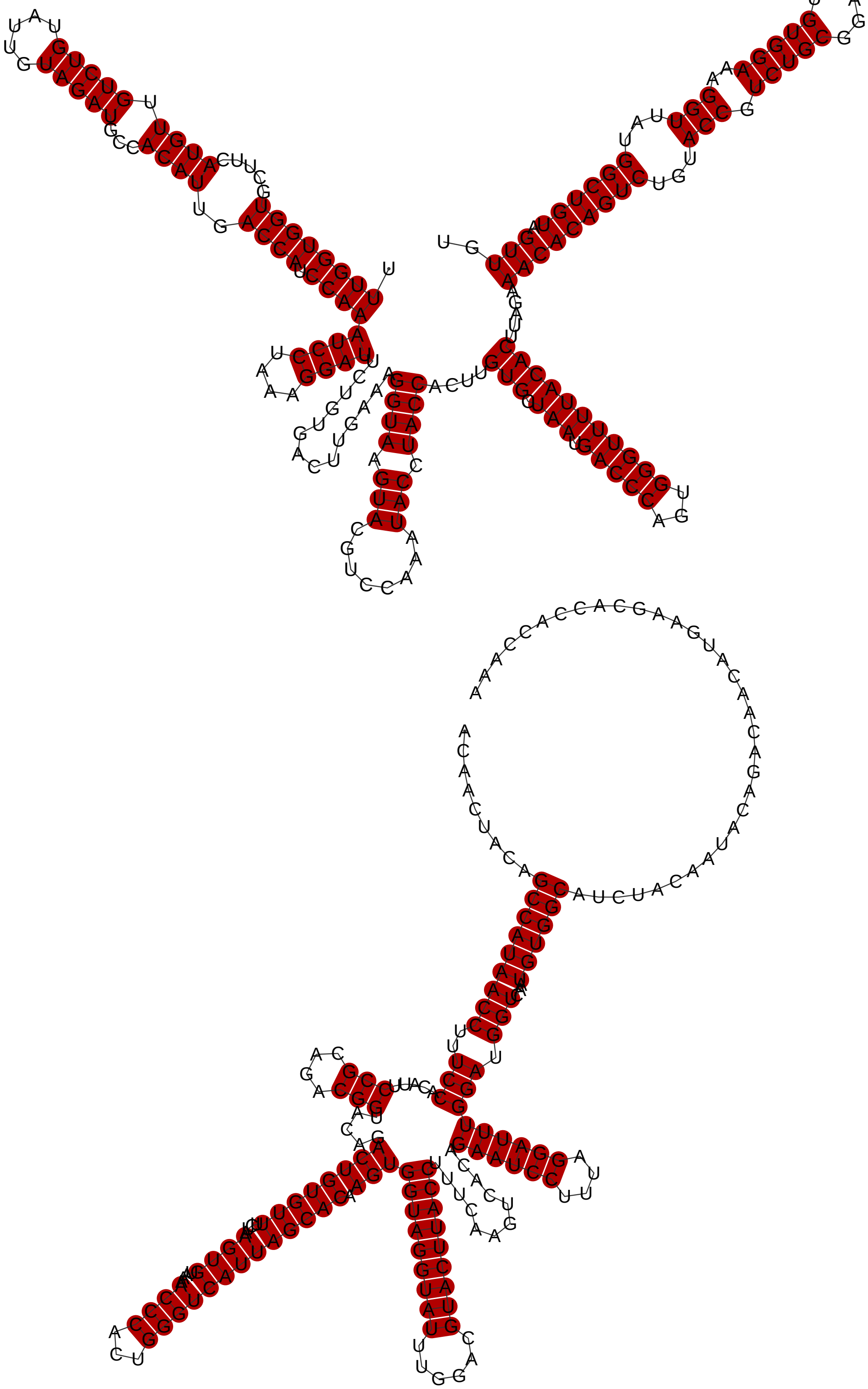

Fig. S90. Output of RNAalifold analysis of the conserved region found 1st in analysis of the nsp10 region of SARS-CoV, AY274119.3 nucleotide location 13153–13344 (top: forward sense; bottom: reverse complement). All sequences used in conservation analysis of the region are included in the RNAalifold analysis (differently from the SARS-CoV-2 RNAalifold analysis, because of the much smaller number of SARS-CoV sequences). Base pairs are highlighted in deep/mid/light red as all/all but one/all but two sequences are capable of forming the pairs shown. Base pairs are highlighted in deep/mid/light yellow as all/all but one/all but two sequences are capable of forming the pair shown or one other base pair (including GU pairs). Base pairs are highlighted in deep/mid/light green as all/all but one/all but two sequences are capable of forming the pair shown or either of another two base pairs (including GU pairs). RNAalifold was used with input options disallowing lonely pairs, allowing G-quadruplexes, and with the ribosum scoring matrix enabled.

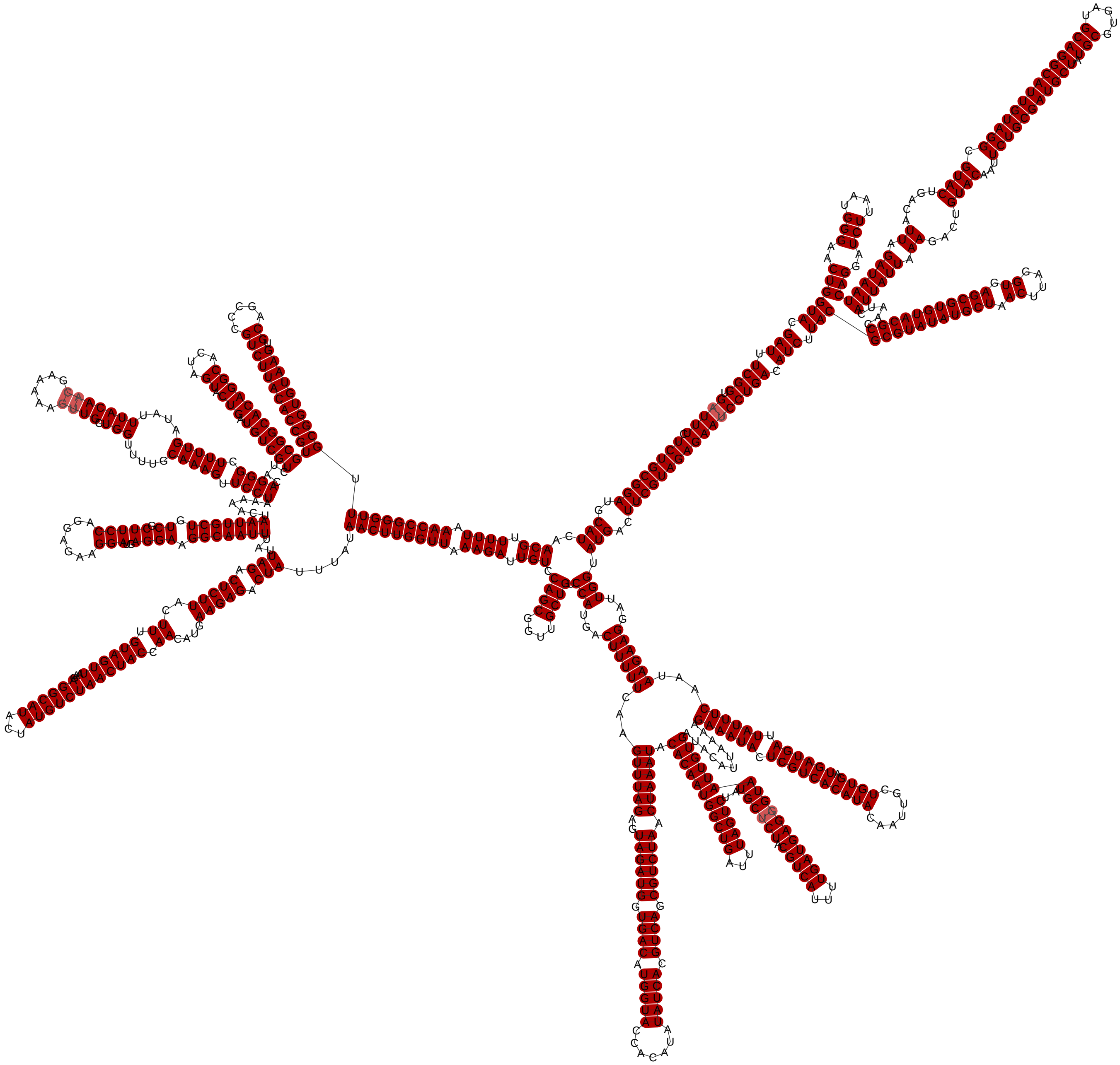

Fig. S91. Output of RNAalifold analysis of the conserved region found 2nd in analysis of the nsp12 region of SARS-CoV, AY274119.3 nucleotide location 13372–14037. All sequences used in conservation analysis of the region are included in the RNAalifold analysis (differently from the SARS-CoV-2 RNAalifold analysis, because of the much smaller number of SARS-CoV sequences). Base pairs are highlighted in deep/mid/light red as all/all but one/all but two sequences are capable of forming the pairs shown. Base pairs are highlighted in deep/mid/light yellow as all/all but one/all but two sequences are capable of forming the pair shown or one other base pair (including GU pairs). Base pairs are highlighted in deep/mid/light green as all/all but one/all but two sequences are capable of forming the pair shown or either of another two base pairs (including GU pairs). RNAalifold was used with input options disallowing lonely pairs, allowing G-quadruplexes, and with the ribosum scoring matrix enabled.

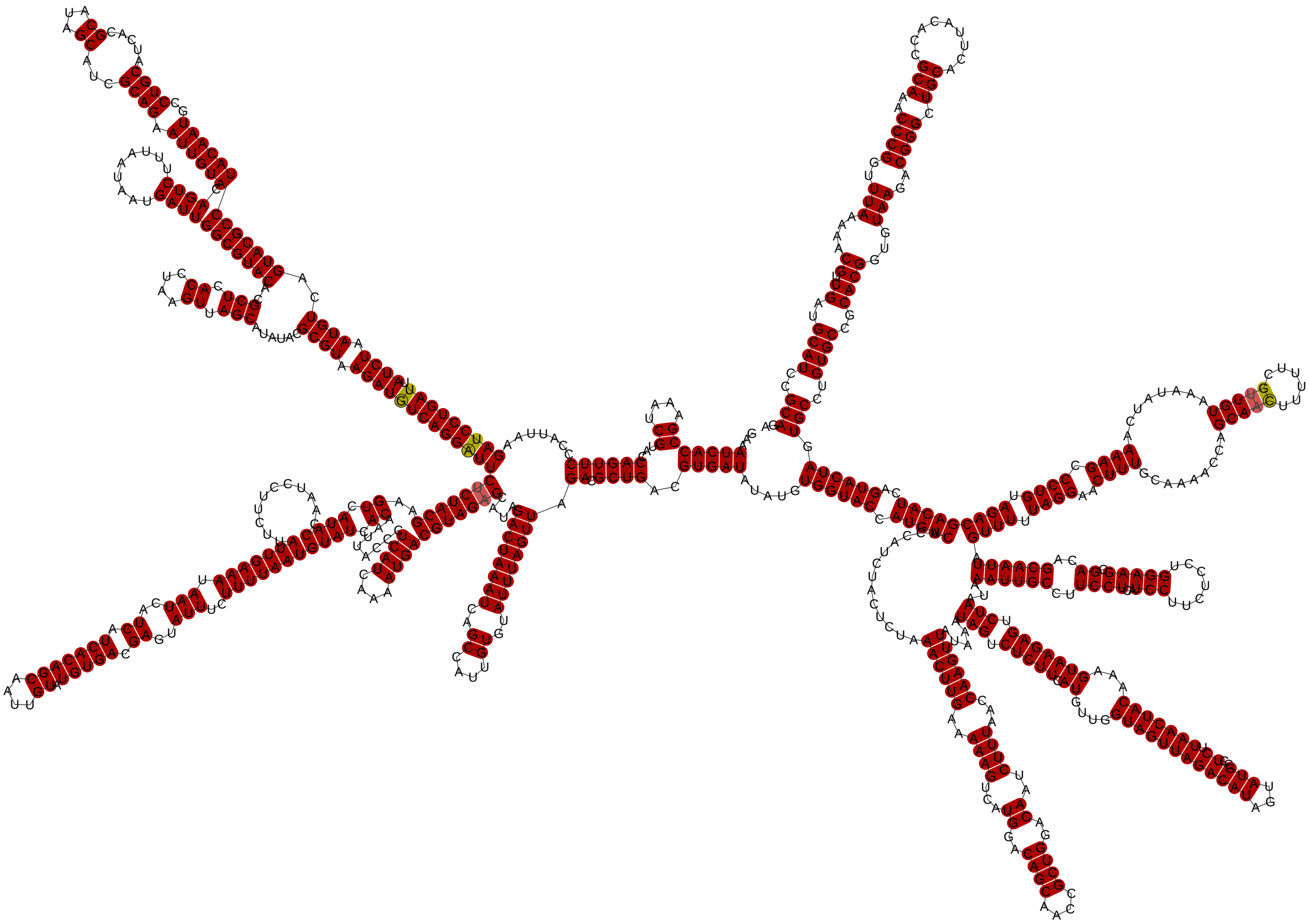

Fig. S92. Output of RNAalifold analysis of the reverse complement of the conserved region found 2nd in analysis of the nsp12 region of SARS-CoV, AY274119.3 nucleotide location 13372–14037. All sequences used in conservation analysis of the region are included in the RNAalifold analysis (differently from the SARS-CoV-2 RNAalifold analysis, because of the much smaller number of SARS-CoV sequences). Base pairs are highlighted in deep/mid/light red as all/all but one/all but two sequences are capable of forming the pairs shown. Base pairs are highlighted in deep/mid/light yellow as all/all but one/all but two sequences are capable of forming the pair shown or one other base pair (including GU pairs). Base pairs are highlighted in deep/mid/light green as all/all but one/all but two sequences are capable of forming the pair shown or either of another two base pairs (including GU pairs). RNAalifold was used with input options disallowing lonely pairs, allowing G-quadruplexes, and with the ribosum scoring matrix enabled.

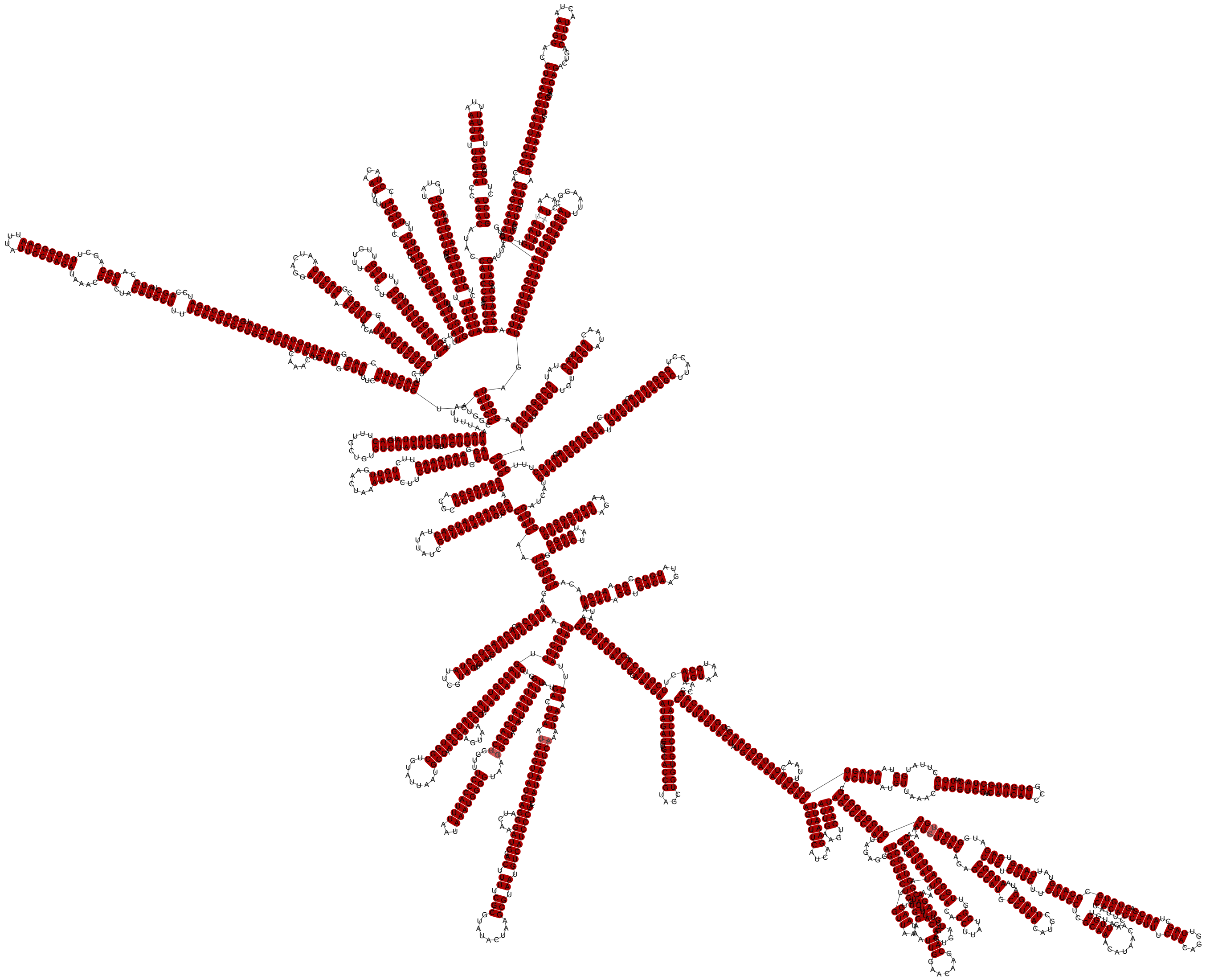

Fig. S93. Output of RNAalifold analysis of the conserved region found 1st in analysis of the nsp12 region of SARS-CoV, AY274119.3 nucleotide location 14208–15852. All sequences used in conservation analysis of the region are included in the RNAalifold analysis (differently from the SARS-CoV-2 RNAalifold analysis, because of the much smaller number of SARS-CoV sequences). Base pairs are highlighted in deep/mid/light red as all/all but one/all but two sequences are capable of forming the pairs shown. Base pairs are highlighted in deep/mid/light yellow as all/all but one/all but two sequences are capable of forming the pair shown or one other base pair (including GU pairs). Base pairs are highlighted in deep/mid/light green as all/all but one/all but two sequences are capable of forming the pair shown or either of another two base pairs (including GU pairs). RNAalifold was used with input options disallowing lonely pairs, allowing G-quadruplexes, and with the ribosum scoring matrix enabled.

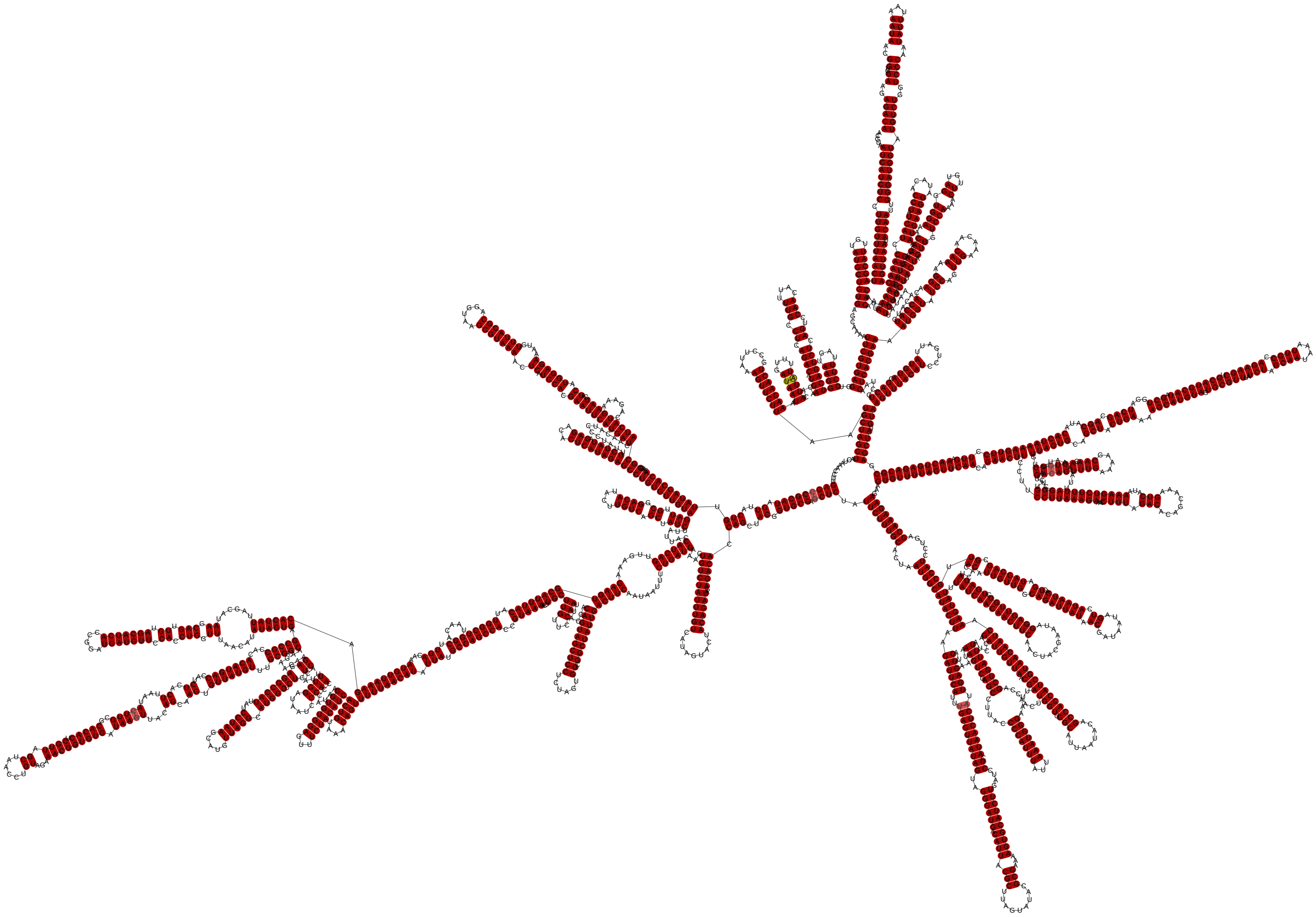

Fig. S94. Output of RNAalifold analysis of the reverse complement of the conserved region found 1st in analysis of the nsp12 region of SARS-CoV, AY274119.3 nucleotide location 14208–15852. All sequences used in conservation analysis of the region are included in the RNAalifold analysis (differently from the SARS-CoV-2 RNAalifold analysis, because of the much smaller number of SARS-CoV sequences). Base pairs are highlighted in deep/mid/light red as all/all but one/all but two sequences are capable of forming the pairs shown. Base pairs are highlighted in deep/mid/light yellow as all/all but one/all but two sequences are capable of forming the pair shown or one other base pair (including GU pairs). Base pairs are highlighted in deep/mid/light green as all/all but one/all but two sequences are capable of forming the pair shown or either of another two base pairs (including GU pairs). RNAalifold was used with input options disallowing lonely pairs, allowing G-quadruplexes, and with the ribosum scoring matrix enabled.

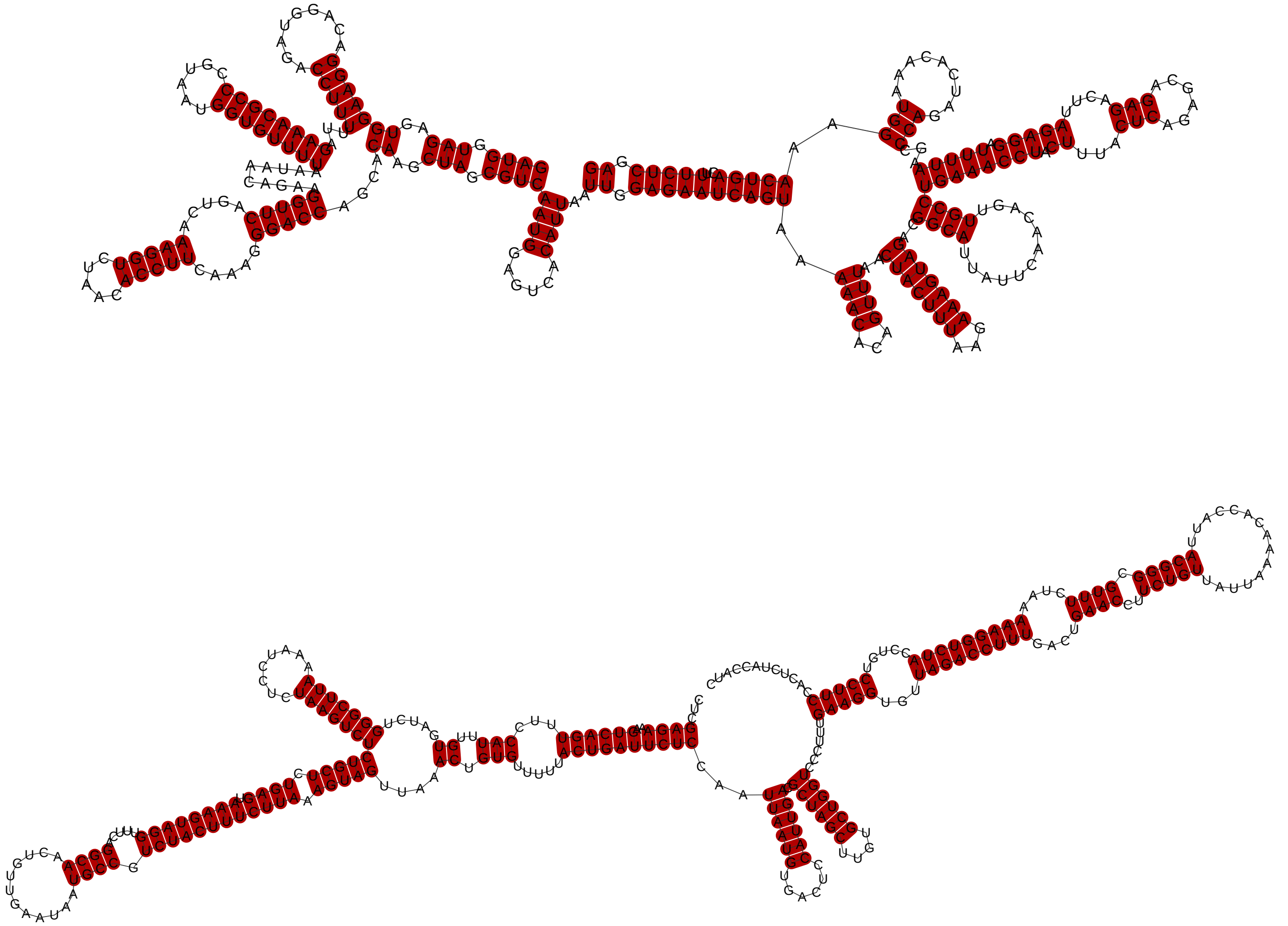

Fig. S95. Output of RNAalifold analysis of the conserved region found 1st in analysis of the nsp15 region of SARS-CoV, AY274119.3 nucleotide location 19920–20195 (top: forward sense; bottom: reverse complement). All sequences used in conservation analysis of the region are included in the RNAalifold analysis (differently from the SARS-CoV-2 RNAalifold analysis, because of the much smaller number of SARS-CoV sequences). Base pairs are highlighted in deep/mid/light red as all/all but one/all but two sequences are capable of forming the pairs shown. Base pairs are highlighted in deep/mid/light yellow as all/all but one/all but two sequences are capable of forming the pair shown or one other base pair (including GU pairs). Base pairs are highlighted in deep/mid/light green as all/all but one/all but two sequences are capable of forming the pair shown or either of another two base pairs (including GU pairs). RNAalifold was used with input options disallowing lonely pairs, allowing G-quadruplexes, and with the ribosum scoring matrix enabled.

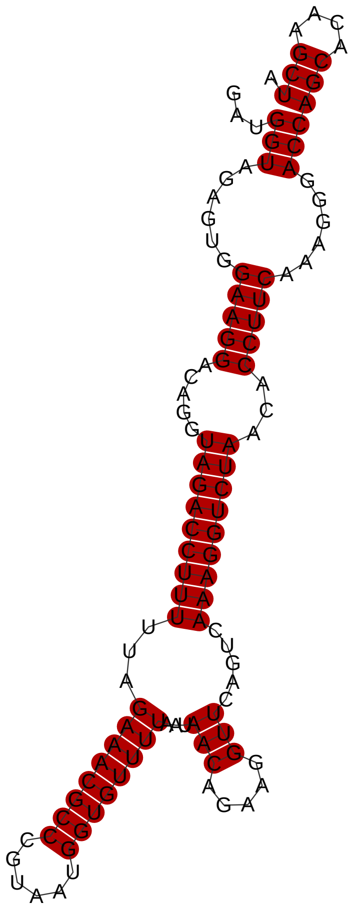

Fig. S96. Output of RNAalifold analysis if a fold is attempted if a positive-sense truncation of the conserved region of Fig. S95, AY274119.3 nucleotide location 19920–20031, is attempted. The central stem-loop at the left of the upper image in the preceding figure is maintained, but the two predicted stem-loops adjacent to it are lost. The change in prediction reflects both low confidence in the exact identification of the appropriate region to fold, because of lower granularity in determining the boundaries of conserved regions when there are few sequences to analyse, and the folding algorithm not being able to use data on covarying base pairs to make better predictions, because no covarying base pairs are seen in the alignment of the region. All sequences used in conservation analysis of the region are included in the RNAalifold analysis (differently from the SARS-CoV-2 RNAalifold analysis, because of the much smaller number of SARS-CoV sequences). Base pairs are highlighted in deep/mid/light red as all/all but one/all but two sequences are capable of forming the pairs shown. Base pairs are highlighted in deep/mid/light yellow as all/all but one/all but two sequences are capable of forming the pair shown or one other base pair (including GU pairs). Base pairs are highlighted in deep/mid/light green as all/all but one/all but two sequences are capable of forming the pair shown or either of another two base pairs (including GU pairs). RNAalifold was used with input options disallowing lonely pairs, allowing G-quadruplexes, and with the ribosum scoring matrix enabled.
